# Supplementary material for: Factors affecting the implementation of calcium supplementation strategies during pregnancy to prevent pre-eclampsia: a mixed-methods systematic review
Source: BMJ Open. 2023 Dec 22;13(12):e070677. doi: 10.1136/bmjopen-2022-070677 (PMC10749021; doi:10.1136/bmjopen-2022-070677)
Supplement: Supplementary data [file bmjopen-2022-070677supp003.pdf]

Appendix 2. Enhancing transparency in reporting the synthesis of qualitative research: ENTREQ Checklist (Tong, et al., 2012)

Item No.	Guide and Description	Report Location
1. Aim	State the research question the synthesis addresses	Introduction
2. Synthesis methodology	Identify the synthesis methodology or theoretical framework which underpins the synthesis, and describe the rationale for choice of methodology (e.g. meta-ethnography, thematic synthesis, critical interpretive synthesis, grounded theory synthesis, realist synthesis, meta-aggregation, meta-study, framework synthesis)	Methods – _ Data extraction and assessing the methodological limitations
3. Approach to searching	Indicate whether the search was pre-planned (comprehensive search strategies to seek all available studies) or iterative (to seek all available concepts until they theoretical saturation is achieved)	Methods – Search methods for identification of studies
4. Inclusion criteria	Specify the inclusion/exclusion criteria (e.g. in terms of population, language, year limits, type of publication, study type)	Methods – Selection of studies
5. Data sources	Describe the information sources used (e.g. electronic databases (MEDLINE, EMBASE, CINAHL, psycINFO), grey literature databases (digital thesis, policy reports), relevant organisational websites, experts, information specialists, generic web searches (Google Scholar) hand searching, reference lists) and when the searches conducted; provide the rationale for using the data sources	Methods - Search methods for identification of studies
6. Electronic Search strategy	Describe the literature search (e.g. provide electronic search strategies with population terms, clinical or health topic terms, experiential or social phenomena related terms, filters for qualitative research, and search limits)	Appendix 3
7. Study screening methods	Describe the process of study screening and sifting (e.g. title, abstract and full text review, number of independent reviewers who screened studies)	Methods - Search methods for identification of studies
8. Study characteristics	Present the characteristics of the included studies (e.g. year of publication, country, population, number of participants, data collection, methodology, analysis, research questions)	Table 1 - Characteristics of included studies
9. Study selection results	Identify the number of studies screened and provide reasons for study exclusion (e.g. for comprehensive searching, provide numbers of studies screened and reasons for exclusion indicated in a figure/flowchart; for iterative searching describe reasons for study exclusion	Fig 1 - PRISMA flow diagram

	and inclusion based on modifications to the research question and/or contribution to theory development)	
10. Rationale for appraisal	Describe the rationale and approach used to appraise the included studies or selected findings (e.g. assessment of conduct (validity and robustness), assessment of reporting (transparency), assessment of content and utility of the findings)	Methods - Quality appraisal, analysis and assessing confidence and Appendix 4
11. Appraisal items	State the tools, frameworks and criteria used to appraise the studies or selected findings (e.g. Existing tools: CASP, QARI, COREQ, Mays and Pope [25]; reviewer developed tools; describe the domains assessed: research team, study design, data analysis and interpretations, reporting)	Methods - Data extraction and assessing the methodological limitations
12. Appraisal process	Indicate whether the appraisal was conducted independently by more than one reviewer and if consensus was required	Methods - Data extraction and assessing the methodological limitations
13. Appraisal results	Present results of the quality assessment and indicate which articles, if any, were weighted/excluded based on the assessment and give the rationale	Appendix 5
14. Data extraction	Indicate which sections of the primary studies were analysed and how were the data extracted from the primary studies? (e.g. all text under the headings "results /conclusions" were extracted electronically and entered into a computer software)	Methods- Data extraction and assessing the methodological limitations
15. Software	State the computer software used, if any	None
16. Number of reviewers	Identify who was involved in coding and analysis	Methods - Data extraction and assessing the methodological limitations
17. Coding	Describe the process for coding of data (e.g. line by line coding to search for concepts)	Methods - Quality appraisal, analysis and assessing confidence
18. Study comparison	Describe how were comparisons made within and across studies (e.g. subsequent studies were coded into pre-existing concepts, and new concepts were created when deemed necessary)	Methods - Quality appraisal, analysis and assessing confidence

19. Derivation of themes	Explain whether the process of deriving the themes or constructs was inductive or deductive	Methods - Quality appraisal, analysis and assessing confidence
20. Quotations	Provide quotations from the primary studies to illustrate themes/constructs, and identify whether the quotations were participant quotations of the author's interpretation	Results
21. Synthesis output	Present rich, compelling and useful results that go beyond a summary of the primary studies (e.g. new interpretation, models of evidence, conceptual models, analytical framework, development of a new theory or construct)	Discussion
