# Supplementary material for: Factors affecting the implementation of calcium supplementation strategies during pregnancy to prevent pre-eclampsia: a mixed-methods systematic review
Source: BMJ Open. 2023 Dec 22;13(12):e070677. doi: 10.1136/bmjopen-2022-070677 (PMC10749021; doi:10.1136/bmjopen-2022-070677)
Supplement: Supplementary data [file bmjopen-2022-070677supp004.pdf]

Appendix 3. Search Strategy

Embase (inception to 2021 March 22)

1 exp Calcium/ or Calcium Carbonate/ (305629)
2 (calcium adj3 supplement*).mp. (9456)
3 1 or 2 (307747)
4 Pregnant Women/ or Prenatal Care/ (109074)
5 (pregnan* or prenatal).mp. (1086333)
6 4 or 5 (1086333)
7 3 and 6 (8440)
8 limit 7 to humans (5885)
9 limit 8 to (amphibia or ape or bird or cat or cattle or chicken or dog or "ducks and geese" or fish or "frogs and toads" or goat or guinea pig or "hamsters and gerbils" or horse or monkey or mouse or "pigeons and doves" or "rabbits and hares" or rat or reptile or sheep or swine) (277)
10 8 not 9 (5608)
11 limit 10 to (conference abstract or conference paper or "conference review" or editorial or erratum or letter or note or "review") (2567)
12 10 not 11 (**3041**)

Embase (inception to 2022 August 16) – Search update

1 exp Calcium/ or Calcium Carbonate/ (323807)
2 (calcium adj3 supplement*).mp. (10017)
3 1 or 2 (326035)
4 Pregnant Women/ or Prenatal Care/ (125455)
5 (pregnan* or prenatal).mp. (1161704)
6 4 or 5 (1161704)
7 3 and 6 (9061)
8 limit 7 to humans (6413)
9 limit 8 to (amphibia or ape or bird or cat or cattle or chicken or dog or "ducks and geese" or fish or "frogs and toads" or goat or guinea pig or "hamsters and gerbils" or horse or monkey or mouse or "pigeons and doves" or "rabbits and hares" or rat or reptile or sheep or swine) (303)
10 8 not 9 (6110)
11 limit 10 to (conference abstract or conference paper or "conference review" or editorial or erratum or letter or note or "review") (2744)
12 10 not 11 (3366)
13 limit 12 to yr="2022 - 2023" (**142**)

MEDLINE (1946 to March Week 2 2021)

1 exp Calcium/ or Calcium Carbonate/ (277850)
2 (calcium adj3 supplement*).mp. (5560)

3 1 or 2 (280742)
4 Pregnant Women/ or Prenatal Care/ (36709)
5 (pregnan* or prenatal).mp. (999134)
6 4 or 5 (999134)
7 3 and 6 (6417)
8 limit 7 to humans (3417)
9 limit 8 to animals (618)
10 8 not 9 (2799)
11 limit 10 to ("review articles" and case reports) (17)
12 10 not 11 (**2782 results**)

MEDLINE (inception to August Week 1, 2022) – Search update

1 exp Calcium/ or Calcium Carbonate/ (286238)
2 (calcium adj3 supplement*).mp. (5967)
3 1 or 2 (289314)
4 Pregnant Women/ or Prenatal Care/ (42373)
5 (pregnan* or prenatal).mp. (1068999)
6 4 or 5 (1068999)
7 3 and 6 6605
8 limit 7 to humans (3545)
9 limit 8 to animals (634)
10 8 not 9 (2911)
11 limit 10 to ("review articles" and case reports) (18)
12 10 not 11 (2893)
13 limit 12 to yr="2021 - 2022" (**89**)

CINAHL

CINAHL (inception to March 2021)

(calcium and supplement*) AND (pregnan* or prenatal) AND (preeclamp* or pre-eclamp* or eclamp* or "gestational hypertension" or "maternal hypertension") (**132**)

CINAHL (March 2021 to August 2022) – Search update

(calcium and supplement*) AND (pregnan* or prenatal) AND (preeclamp* or pre-eclamp* or eclamp* or "gestational hypertension" or "maternal hypertension") (**1**)

GLOBAL HEALTH

GLOBAL HEALTH (inception to March 2021)

(calcium and supplement*) AND (pregnan* or prenatal) AND (preeclamp* or pre-eclamp* or eclamp* or "gestational hypertension" or "maternal hypertension") (**158 results**)

GLOBAL HEALTH (2021 to August 2022) – Search update

(calcium and supplement*) AND (pregnan* or prenatal) AND (preeclamp* or pre-eclamp* or eclamp* or "gestational hypertension" or "maternal hypertension") (**158 results**)
