# Supplementary material for: Factors affecting the implementation of calcium supplementation strategies during pregnancy to prevent pre-eclampsia: a mixed-methods systematic review
Source: BMJ Open. 2023 Dec 22;13(12):e070677. doi: 10.1136/bmjopen-2022-070677 (PMC10749021; doi:10.1136/bmjopen-2022-070677)
Supplement: Supplementary data [file bmjopen-2022-070677supp005.pdf]

Appendix 4. Critical Appraisal Table

Qualitative studies

STUDY DETAIL	SCREENING QUESTIONS		1. QUALITATIVE STUDIES							
First author	S1. Are there clear research questions?	S2. Do the collected data allow to address the research questions?	1.1. Is the qualitative approach appropriate to answer the research question? (Aim, appropriateness of a qualitative approach)	1.2. Are the qualitative data collection methods adequate to address the research question? (recruitment, data collection)	1.3. Are the findings adequately derived from the data? (rigor in analysis)	1.4. Is the interpretation of results sufficiently substantiated by data? (link from data to findings)	1.5. Is there coherence between qualitative data sources, collection, analysis and interpretation? (overall design from start to finish)	1.6. Have ethical issues been taken into consideration? (consent, confidentiality, ethics approval)	1.7. Is relationship between researcher and participants adequately considered? (interaction and reflection on how research team influences design & implementation)	MMAT RATING
**Vestering 2019**	Yes	Yes	Yes	Yes	Yes	Yes	Yes	Partial	Partial	"Moderate" (minor issues impacting credibility/validity)
**Birhanu 2018**	Yes	Yes	Yes	Yes	Yes	Yes	Yes	Yes	Unclear	"Moderate" (minor issues impacting credibility/validity)
**Martin 2017a**	Yes	Yes	Yes	Yes	Yes	Yes	Yes	Yes	Unclear	"Moderate" (minor issues impacting credibility/validity)
**Martin 2018**	Yes	Yes	Yes	Yes	Yes	Yes	Yes	Yes	Partial	"Moderate" (minor issues impacting credibility/validity)

Quantitative studies

STUDY DETAIL	SCREENING QUESTIONS		3. NON-RANDOMIZED STUDIES					4. QUANTITATIVE DESCRIPTIVE STUDIES					MMAT RATING
First author	S1. Are there clear research questions?	S2. Do the collected data allow to address the research questions?	3.1. Are the participants representative of the target population?	3.2. Are measurements appropriate regarding both the outcome and intervention (or exposure)?	3.3. Are there complete outcome data?	3.4. Are the confounders accounted for in the design and analysis?	3.5. During the study period, is the intervention administered (or exposure occurred) as intended?	4.1. Is the sampling strategy relevant to address the research question?	4.2. Is the sample representative of the target population?	4.3. Are the measurements appropriate?	4.4. Is the risk of nonresponse bias low?	4.5. Is the statistical analysis appropriate to answer the research question?	
Baxter 2014	Yes	Yes						Yes	Unclear	Yes	No	Yes	“Low” (some issues likely to impact credibility/validity)
Thapa 2016	Yes	Yes	Yes	Yes	Yes	Yes	Yes						"High” (no or very minor significant issues)
Nguyen 2019	Yes	Yes						Yes	Yes	Yes	Unclear	Yes	“Moderate” (minor issues impacting credibility/validity)
Omotayo 2018a & Martin 2017b	Yes	Yes	Yes	Yes	Partial	Yes	Yes						“Moderate” (minor issues impacting credibility/validity)
Nguyen 2017	Yes	Yes						Yes	Yes	Yes	Yes	Yes	"High” (no or very minor significant issues)
Nguyen 2018	Yes	Yes						Yes	Yes	Yes	Yes	Yes	"High” (no or very minor significant issues)

Liu 2019	Yes	Yes						Yes	Yes	Partial	No	Yes	“Low” (some issues likely to impact credibility/validity)
Shakya Shrestha 2020	Yes	Yes						Partial	Unclear	Partial	Yes	Partial	“Very low” (significant issues impacting credibility/validity)
Ghosh-Jerath 2015	Yes	Yes						Yes	Yes	Partial	No	Yes	“Low” (some issues likely to impact credibility/validity)

*Grey shades or empty cells refer to not applicable.

Mixed methods studies

STUDY DETAIL	First author	Omotayo 2018b & Martin 2017c	Kachwaha 2022
SCREENING QUESTIONS	S1. Are there clear research questions?	Yes	Yes
	S2. Do the collected data allow to address the research questions?	Yes	Yes
1. QUALITATIVE STUDIES	1.1. Is the qualitative approach appropriate to answer the research question? (Aim, appropriateness of a qualitative approach)	Yes	Yes
	1.2. Are the qualitative data collection methods adequate to address the research question? (recruitment, data collection)	Yes	Yes
	1.3. Are the findings adequately derived from the data? (rigor in analysis)	Yes	Yes
	1.4. Is the interpretation of results sufficiently substantiated by data? (link from data to findings)	Yes	Yes
	1.5. Is there coherence between qualitative data sources, collection, analysis and interpretation? (overall design from start to finish)	Yes	Yes
	1.6. Have ethical issues been taken into consideration? (consent, confidentiality, ethics approval)	Yes	Yes
	1.7. Is relationship between researcher and participants adequately considered? (interaction and reflection on how research team influences design & implementation)	Unclear	Unclear
	4.1. Is the sampling strategy relevant to address the research question?	Yes	Yes

**4. QUANTITATIVE DESCRIPTIVE STUDIES**	**4.2. Is the sample representative of the target population?**	Yes	Yes
	**4.3. Are the measurements appropriate?**	Yes	Yes
	**4.4. Is the risk of nonresponse bias low?**	Yes	Partial
	**4.5. Is the statistical analysis appropriate to answer the research question?**	Yes	Yes
**5. MIXED METHODS STUDIES**	**5.1. Is there an adequate rationale for using a mixed methods design to address the research question?**	Yes	Yes
	**5.2. Are the different components of the study effectively integrated to answer the research question?**	Yes	Yes
	**5.3. Are the outputs of the integration of qualitative and quantitative components adequately interpreted?**	Yes	Yes
	**5.4. Are divergences and inconsistencies between quantitative and qualitative results adequately addressed?**	Yes	Yes
	**5.5. Do the different components of the study adhere to the quality criteria of each tradition of the methods involved?**	Yes	Partial
**MMAT RATING**		"High" (no or very minor significant issues)	"Moderate" (minor issues impacting credibility/validity)
