# Supplementary material for: Factors affecting the implementation of calcium supplementation strategies during pregnancy to prevent pre-eclampsia: a mixed-methods systematic review
Source: BMJ Open. 2023 Dec 22;13(12):e070677. doi: 10.1136/bmjopen-2022-070677 (PMC10749021; doi:10.1136/bmjopen-2022-070677)
Supplement: Supplementary data [file bmjopen-2022-070677supp001.pdf]

# Appendix 5. Evidence profile

| No | Summary of qualitative review findings | Contributing qualitative studies | Methodological limitations assessment | Coherence assessment | Relevance assessment | Adequacy assessment | Overall CERQual assessment | Explanation of overall assessment |
|---|---|---|---|---|---|---|---|---|
| **Women's factors** | | | | | | | | |
| **1. Women's knowledge and learning** | | | | | | | | |
| **1.1** | **Women's knowledge about pre-eclampsia or eclampsia** Most women had limited knowledge about pre-eclampsia or eclampsia, and these conditions were not typically considered by women to be a serious problem. Symptoms of pre-eclampsia such as swollen feet, severe headache, blurred vision and vomiting were considered normal signs and symptoms in pregnancy, while seizures were associated with evil attacks or nutritional deficiencies. | (Martin 2017b, Birhanu 2018, Vestering 2019) | **Minor concerns:** Three studies with minor issues (reflexivity and ethics). | **No or very minor concerns** | **Moderate concerns:** One study is indirectly relevant to review aim and represented 3 countries (Kenya, Ethiopia, Netherlands), including 1 low-income, 1 lower middle income and 1 high income country. All perspectives came from health providers and women. | **Minor concerns:** 3 out of 6 studies contributed to review finding (2 thick data, 1 thin data). | **High confidence** | Due to no or very minor concerns on coherence, minor concerns on methodological limitations (reflexivity and ethics), moderate concerns on relevance (1 out of 3 studies are indirectly relevant to our review aim and small number of countries), and minor concerns of adequacy (3 out of 6 contributed with 2 thick and 1 thin data). |
| **1.2** | **Information provision to women** Women felt they did not receive adequate information during pregnancy from healthcare providers about pre-eclampsia or eclampsia and calcium supplements. They described wanting to be given more information, regardless of their pre-eclampsia risk status. Women believed that having this essential information could help them to make | (Vestering 2019, Martin 2018) | **Minor concerns:** Two studies with minor issues (ethics and reflexivity). | **Moderate concerns:** No good understanding why some providers worry in generating anxiety to women | **Moderate concerns:** All studies are directly relevant to review aim and represented 2 countries (Kenya, Netherlands), including 1 lower middle income and 1 high income country. All perspectives came from health providers and women. | **Serious concerns:** 2 out of 6 studies contributed to review finding (1 thick data, 1 thin data). | **Low confidence** | Minor concerns on methodological limitations (ethics and reflexivity), moderate concerns on coherence (no clear understanding on why some providers worry in generating anxiety to women while the others are not), moderate concerns on relevance (small number of countries), and |

| No | Summary of qualitative review findings | Contributing qualitative studies | Methodological limitations assessment | Coherence assessment | Relevance assessment | Adequacy assessment | Overall CERQual assessment | Explanation of overall assessment |
|---|---|---|---|---|---|---|---|---|
| | informed choices and actively participate in their care. There were, however, mixed opinions from healthcare providers - some feared that more information could generate anxiety for women, while others were more supportive of providing information. | | | while others are not. | | | | serious concerns on adequacy (2 out of 6 contributed with 1 thick and 1 thin data). |
| **1.3** | **Learning about calcium supplements** Women typically learned about dietary calcium, including pre-eclampsia and eclampsia symptoms, from healthcare providers. They considered healthcare providers to be the most trusted and reliable source of information and reported feeling confident about taking calcium-containing supplements after receiving adequate information from them. Women also appreciated receiving information on calcium supplements and pre-eclampsia or eclampsia via information, education, and communication (IEC) materials like videos, media, and trusted websites. | (Vestering 2019, Martin 2018, Birhanu 2018) | **Minor concerns:** Three studies with minor issues (reflexivity and ethics). | **No or very minor concerns** | **Minor concerns:** All studies are directly relevant to review aim and represented 3 countries (Kenya, Ethiopia, Netherlands), including 1 low-income, 1 lower middle income and 1 high income country. All perspectives came from health providers and women. | **Minor concerns:** 3 out of 6 studies contributed to findings (2 with moderate-thick data and 1 with thin data. | **High confidence** | Due to no or very minor concerns on coherence, minor concerns on relevance (small number of countries), minor concerns on methodological limitations (reflexivity and ethics), and minor concerns on adequacy (3 out of 6 contributed, 1 moderate thick and 2 thin data). |

| No | Summary of qualitative review findings | Contributing qualitative studies | Methodological limitations assessment | Coherence assessment | Relevance assessment | Adequacy assessment | Overall CERQual assessment | Explanation of overall assessment |
|---|---|---|---|---|---|---|---|---|
| **2. Women's beliefs about calcium supplements in pregnancy** | | | | | | | | |
| **2.1** | **Fears about side-effects as a barrier to calcium supplements uptake** Women's fears about the side effects of calcium supplements affected their adherence. Women highlighted that assurance of safe use of calcium supplement is a key facilitator to consistent use. However, some women felt safety was not assured by healthcare providers, especially when calcium supplements were perceived as "experimental". Women had also received messages from their families or communities that any pills consumed during pregnancy could be harmful. | (Omotayo 2017, Birhanu 2018, Vestering 2019, Martin 2018) | **Minor concerns:** Three out of four studies have minor issues (ethics and reflexivity) and one study with no or very minor issues. | **No or very minor concerns** | **Minor concerns:** All studies are directly relevant to review aim and represented 3 countries (Kenya, Ethiopia, Netherlands), including 1 low-income, 1 lower middle income and 1 high income country. All perspectives came from women. | **Minor concerns** Overall, small number of studies contributing to the qualitative evidence synthesis. | **High Confidence** | Due to no or very minor concerns on coherence, minor concerns on relevance (small number of countries), minor concerns on adequacy (small number of studies contributing to the qualitative evidence synthesis), and minor concerns on methodological limitations (reflexivity and ethics). |
| **2.2** | **Women's experiences of side-effects** Some women reported experiencing side effects after taking calcium and iron-folic acid supplements, such as dizziness, vomiting, nausea, stomach aches, loss of appetite, tiredness, diarrhoea, bloating, and burping, yet noted that side effects subsided with time. Women also reported that they continued consuming calcium despite | (Omotayo 2017, Martin 2018, Birhanu 2018) | **Minor concerns:** Two out of three studies have minor issues (ethics and reflexivity) and one study with no or very minor issues | **No or very minor concerns** | **Moderate concerns:** All studies are directly relevant to review aim and represented 2 countries (Kenya, Ethiopia), including 1 low-income and 1 lower middle-income country. All perspectives came from health providers and women. | **Minor concerns:** 3 out of 6 studies contributed to the findings (2 thin and 1 with thick data). | **High Confidence** | Due to no or very minor concerns on coherence, minor concerns on methodological limitations (ethics and reflexivity), moderate concerns on relevance (only low and lower middle-income country and small number of countries) and minor concerns on adequacy (3 out of 6 studies |

| No | Summary of qualitative review findings | Contributing qualitative studies | Methodological limitations assessment | Coherence assessment | Relevance assessment | Adequacy assessment | Overall CERQual assessment | Explanation of overall assessment |
|---|---|---|---|---|---|---|---|---|
| | these side effects. | | | | | | | contributed with 2 thick and 1 with thin data). |
| 2.3 | **Concerns about being stigmatized as HIV patients** Women expressed concerns that if they ingested calcium supplements, they could be stigmatized as HIV patients, which was a reported barrier to use. Some women were afraid of being stigmatized as their community often associated nutritional supplement consumption and accompanying reminder posters with HIV. | (Martin 2018, Vestering 2019) | **No or very minor concerns** | **No or very minor concerns** | **Moderate concerns:** All studies are directly relevant to review aim and represented 2 countries (Kenya, Netherlands), including 1 lower middle income and 1 high income country. All perspectives came from health providers and women. | **Serious concerns:** 2 out of 6 studies contributed to review finding (1 thick data and 1 thin data). | **Moderate Confidence** | Due to no or very minor concerns on methodological limitations, no or very minor concerns on coherence, minor concerns on relevance (small number of countries), and serious concerns on adequacy (2 out of 6 study contributed). |
| 2.4 | **Positive perceptions of calcium supplements** Women reported that both their perceptions about expected benefits and previous experiences of taking calcium and iron-folic acid supplements were facilitators of use. Women believed that consuming pills could compensate for sub-optimal nutrition during pregnancy, and that supplements during pregnancy would help keep their baby safe. | (Vestering 2019, Martin 2018, Martin 2017b, Birhanu 2018, Omotayo 2017) | **Minor concerns:** Four out of five studies have minor issues (ethics and reflexivity) and one study with no or very minor issues. | **No or very minor concerns** | **Minor concerns:** One study is indirectly relevant to review aim and represented 3 countries (Kenya, Ethiopia, Netherlands), including 1 low-income, 1 lower middle income and 1 high income country. All perspectives came from health providers and women. | **Minor concerns** Overall, small number of studies contributing to the qualitative evidence synthesis. | **High Confidence** | Due to no or very minor concerns on coherence, minor concerns on methodological limitations (ethics and reflexivity), minor concerns on relevance (1 study indirectly relevant to review aim and small number of countries) and adequacy (small number of studies contributing to the qualitative evidence synthesis). |
| **3. Calcium supplement characteristics and regimens** | | | | | | | | |

| No | Summary of qualitative review findings | Contributing qualitative studies | Methodological limitations assessment | Coherence assessment | Relevance assessment | Adequacy assessment | Overall CERQual assessment | Explanation of overall assessment |
|---|---|---|---|---|---|---|---|---|
| **3.1** | **Varying preferences about characteristics of calcium tablets** Positive perceptions about the characteristics of the calcium tablet played a role in motivating women to take it. Some women preferred the chewable, sweet-tasting tablets that could be swallowed without water, while others preferred the hard tablets which were smaller in size, had no smell, and needed to be taken with water. Based on individual preference, the taste, smell, size, and convenience affected calcium supplement use. | (Omotayo 2017, Birhanu 2018) | **Minor concerns:** One study has minor issues (reflexivity) and one study has no or very minor issues. | **No or very minor concerns** | **Moderate concerns:** All studies are directly relevant to review aim and represented 2 countries (Kenya, Ethiopia), including 1 low-income and 1 lower middle-income country. All perspectives came from women. | **Moderate concerns:** 2 out of 6 studies contributed to findings and both had moderate to thick data. | **Moderate confidence** | Due to no or very minor concerns on coherence, minor concerns on methodological limitations (reflexivity), moderate concerns on relevance (all low or lower middle-income countries and small number of countries) and moderate concerns on adequacy (2 out of 6 studies contributed with moderate to thick data). |
| **3.2** | **Supplement regimen as a barrier to use** Women described that they feel overwhelmed with the number of calcium tablets they had to take each day, especially women with other comorbidities who needed to take additional medications for their health conditions. Women felt that 3-4 pills per day at multiple times was overly onerous and preferred if they were combined into one pill. | (Martin 2018, Birhanu 2018, Omotayo 2017) | **Minor concerns:** Two studies have minor issues (reflexivity) and one study has no or very minor issues. | **No or very minor concerns** | **Moderate concerns:** All studies are directly relevant to review aim and represented 2 countries (Kenya, Ethiopia), including 1 low-income and 1 lower middle-income country. All perspectives came from health providers and women. | **Minor concerns:** 3 out of 6 studies contributing to findings and all have moderate to thick data. | **High Confidence** | No or very minor concerns on coherence, minor concerns on methodological limitations (reflexivity), minor concerns on adequacy (3 out of 6 studies contributed with moderate to thick data), and moderate concerns on relevance (small number of countries). |
| **4. Daily routines and food insecurity** | | | | | | | | |

| No | Summary of qualitative review findings | Contributing qualitative studies | Methodological limitations assessment | Coherence assessment | Relevance assessment | Adequacy assessment | Overall CERQual assessment | Explanation of overall assessment |
|---|---|---|---|---|---|---|---|---|
| **4.1** | **Adherence challenges due to routines** Adherence to calcium supplements consumption was challenging for some women because of conflicting activities in their daily routine, such as consuming other medications, traveling, being away from home, and household chores, which can lead them to forget to take calcium. | (Martin 2018, Birhanu 2018, Omotayo 2017) | **Minor concerns:** Two out of three studies have minor issues (reflexivity) and one study with no or very minor issues. | **No or very minor concerns** | **Moderate concerns:** All studies are directly relevant to review aim and represented 2 countries (Kenya, Ethiopia), including 1 low-income and 1 lower middle-income country. All perspectives came from health providers and women. | **Minor concerns:** Overall, small number of studies contributing to qualitative evidence synthesis. | **High Confidence** | Due to no or very minor concerns with coherence, minor concerns on methodological limitations (reflexivity), moderate concerns on relevance (small number of countries) and moderate concerns on adequacy (small number of studies contributing to qualitative evidence synthesis). |
| **4.2** | **Food insecurity as a barrier to calcium uptake** Women believed that adequate food was necessary to be able to take the supplements, to avoid nausea. They perceived it as normal to eat before consuming any medication. However, women reported that food insecurity was a critical barrier to calcium uptake. | (Birhanu 2018, Martin 2018) | **Minor concerns:** Two studies with minor issues (reflexivity). | **No or very minor concerns** | **Moderate concerns:** All studies are directly relevant to review aim and represented 2 countries (Kenya, Ethiopia), including 1 low-income and 1 lower middle-income country. All perspectives came from health providers and women. | **Moderate concerns:** 2 out of 6 studies contributed to findings with 1 thick and 1 thin data. | **Moderate Confidence** | Due to no or very minor concern with coherence, minor concerns on methodological limitations (reflexivity), moderate concerns on adequacy (2 out 6 studies contributed with 1 thick and 1 thin data), and moderate concerns on relevance (small number of countries). |
| **5. Strategies to improve the use of calcium supplements** | | | | | | | | |
| **5.1** | **Implementation of reminders to promote adherence** Women and healthcare providers perceived reminders as beneficial in promoting women's adherence to consuming calcium supplements. | (Martin 2017a, Martin 2018, Birhanu 2018, | **No or very minor concerns** | **No or very minor concerns** | **Moderate concerns:** One study indirectly relevant to review aim and represented 2 countries (Kenya, Ethiopia), including 1 | **Minor concerns:** Overall, small numbers of studies | **High Confidence** | Due to no or very minor concerns on methodological limitations, no or very minor concerns on coherence, minor concerns on adequacy (small number of studies contributing |

| No | Summary of qualitative review findings | Contributing qualitative studies | Methodological limitations assessment | Coherence assessment | Relevance assessment | Adequacy assessment | Overall CERQual assessment | Explanation of overall assessment |
|---|---|---|---|---|---|---|---|---|
| | Several reminder strategies were described as useful by women and healthcare providers, such as home-based posters, calendars with illustrations and daily reminders, and integrating supplement consumption into women's daily routine, such as mealtimes. | Omotayo 2017) | | | low-income and 1 lower middle-income country. All perspectives came from health providers and women. | contributing to qualitative evidence synthesis. | | to qualitative evidence synthesis), moderate concerns on relevance (due to all studies coming from low income or lower middle-income country and small number of countries). |
| **5.2** | **Importance of family support and 'adherence partner' implementation** Having family support was instrumental to pregnant women adhering to calcium supplement use. This could be leveraged by notifying family members on the importance of calcium and appointing someone to be an "adherence partner" or "pill buddy" to help remind her to take it. Both women and healthcare providers were positive about adherence partners in providing support in terms of encouraging them to take the supplements, providing food, helping them around the house, providing emotional support, improving family relationships, and | (Martin 2018, Martin 2017b, Birhanu 2018, Martin 2017a, Omotayo 2017) | **No or very minor concerns** | **No or very minor concerns** | **Moderate concerns:** Two out of five studies indirectly relevant to review aim and represented 2 countries (Kenya, Ethiopia), including 1 low-income and 1 lower middle-income country. All perspectives came from health providers, partner and women. | **Minor concerns:** Overall, small number of studies contributing to qualitative evidence synthesis. | **High Confidence** | No or very minor concerns on methodological limitations, no or very minor concerns on coherence, minor concerns on adequacy (small number of studies contributing to qualitative evidence synthesis), and moderate concerns on relevance (due to all studies coming from low income or lower middle-income country only, 2 of 5 studies has indirectly relevant aim, and small number of countries). |

| No | Summary of qualitative review findings | Contributing qualitative studies | Methodological limitations assessment | Coherence assessment | Relevance assessment | Adequacy assessment | Overall CERQual assessment | Explanation of overall assessment |
|---|---|---|---|---|---|---|---|---|
| | thereby increasing partner or husband involvement in pregnancy. | | | | | | | |
| **5.3** | **Counselling facilitates calcium supplements uptake** Both women and healthcare providers acknowledged that counselling women on the benefits of calcium was a motivator to calcium supplements uptake. Women valued the discussion they have with healthcare providers and felt more confident to take calcium supplements when they received counselling and information and pre-eclampsia or eclampsia and the benefits of calcium from their healthcare providers. | (Birhanu 2018, Martin 2018) | **No or very minor concerns** | **No or very minor concerns** | **Moderate concerns:** All studies are directly relevant to review aim and represented 2 countries (Kenya, Ethiopia), including 1 low-income and 1 lower middle-income country. All perspectives came from health providers and women. | **Moderate concerns:** 2 out of 6 studies contributing to findings with all thick data. | **Moderate Confidence** | No or very minor concerns on methodological limitations, no or very minor concerns on coherence, moderate concerns on relevance (due to all studies coming from low or lower middle-income country and small number of countries) and moderate concerns on adequacy (2 out 6 studies contributed with thick data). |
| **Health care providers' factors** | | | | | | | | |
| **6. Healthcare provider knowledge and training** | | | | | | | | |
| **6.1** | **Varied knowledge about pre-eclampsia or eclampsia among healthcare providers** Healthcare providers' knowledge about pre-eclampsia and eclampsia was varied. Some felt that pre-eclampsia and eclampsia is not a priority health concern in their area | (Birhanu 2018, Martin 2017a, Kachwaha 2022) | **No or very minor concerns** | **No or very minor concerns** | **Moderate concerns:** One study is indirectly relevant to review aim and represented 2 countries (Kenya, Ethiopia), including 1 low-income and 1 lower middle-income country. | **Moderate concerns:** 2 out of 6 studies contributed to findings with thick data. | **Moderate Confidence** | Due to no or very minor concerns on methodological limitations and coherence, moderate concerns on relevance (due to all studies coming from low or lower middle-income countries and small number of studies) and |

| No | Summary of qualitative review findings | Contributing qualitative studies | Methodological limitations assessment | Coherence assessment | Relevance assessment | Adequacy assessment | Overall CERQual assessment | Explanation of overall assessment |
|---|---|---|---|---|---|---|---|---|
| | and reported never having encountered any case. | | | | All perspectives came from health providers. | | | moderate concerns on adequacy (2 out 6 studies contributed with thick data). |
| 6.2 | **Inadequate training to diagnose and treat pre-eclampsia and eclampsia** While some healthcare providers mentioned that training about pre-eclampsia or eclampsia and calcium supplements was adequate, others reported that their training lacked depth and continuity, and thus felt unprepared to diagnose it and offer information these conditions and the use of calcium for prevention. Healthcare providers expressed the need to have more and continuous training on managing pre-eclampsia, as well as time to address concerns or resistance from the community. | (Martin 2018, Birhanu 2018, Martin 2017a) | **No or very minor concerns** | **No or very minor concerns** | **Moderate concerns:** One study is indirectly relevant to review aim and represented 2 countries (Kenya, Ethiopia), including 1 low-income and 1 lower middle-income country. All perspectives came from health providers. | **Minor concerns:** 3 out of 6 studies contributed with 2 thick and 1 thin data. | **High Confidence** | No or very minor concerns on methodological limitations, coherence, moderate concerns on relevance (due to all studies coming from low income or lower middle-income country and small number of countries) and minor concerns on adequacy (3 out of 6 studies contributed with 2 thick and 1 thin data). |
| **7. Beliefs about the intervention** | | | | | | | | |
| 7.1 | **Perceived overmedicalization when prescribing calcium supplements** Both healthcare providers and women perceived that prescribing more tablets to “low-risk” women during pregnancy was a form of over- | (Vestering 2019) | **Minor concerns:** One study with minor issues (reflexivity and ethics). | **No or very minor concerns** | **Serious concerns:** One study is directly relevant to review aim and represented 1 country (Netherlands), which is high income country. All perspectives | **Serious concerns:** 1 out of 6 study contributed to findings | **Low confidence** | No or very minor concerns on coherence, minor concerns on methodological limitations (reflexivity and ethics), serious concerns on relevance (evidence coming from high income country only), serious |

| No | Summary of qualitative review findings | Contributing qualitative studies | Methodological limitations assessment | Coherence assessment | Relevance assessment | Adequacy assessment | Overall CERQual assessment | Explanation of overall assessment |
|---|---|---|---|---|---|---|---|---|
| | medicalization of pregnancy. However, some healthcare providers felt that calcium supplements were a way to prevent further medicalization due to pre-eclampsia-related complications. | | | | came from health providers and women. | with thick data. | | concerns on adequacy (1 out of 6 study contributed). |
| **7.2** | **Beliefs about calcium supplements** Healthcare providers generally had positive beliefs about calcium supplements and there was optimism that calcium could be delivered through antenatal care healthcare providers. Some facilitators motivating healthcare providers to prescribe calcium supplements included their beliefs in its prevention value and expected benefits, that women liked the calcium supplements and experienced benefits from it, and a perceived lack of knowledge on how to treat pre-eclampsia which motivated healthcare providers to side towards prevention. | (Martin 2018, Vestering 2019, Birhanu 2018) | **Minor concerns:** Two studies with minor issues (ethics and reflexivity). | **No or very minor concerns** | **Minor concerns:** All studies are directly relevant to review aim and represented 3 countries (Kenya, Ethiopia, Netherlands), including 1 low-income, 1 lower middle income and 1 high income country. All perspectives came from health providers. | **Minor concerns:** 3 out of 6 studies contributed with 2 thick and 1 thin data. | **High Confidence** | No or very minor concerns on coherence, minor concerns on relevance (small number of countries), minor concerns on methodological limitations (ethics and reflexivity), and minor concerns on adequacy (3 out of 6 studies contributed with 2 thick and 1 thin data). |
| **8. Structural factors** | | | | | | | | |

| No | Summary of qualitative review findings | Contributing qualitative studies | Methodological limitations assessment | Coherence assessment | Relevance assessment | Adequacy assessment | Overall CERQual assessment | Explanation of overall assessment |
|---|---|---|---|---|---|---|---|---|
| **8.1** | **High workload, inadequate staffing, stock out, and lack of equipment** In general, healthcare providers felt that their workload increased by including calcium supplements in antenatal care for pregnant women. Healthcare providers reported existing inadequate staffing, yet they needed to provide additional counselling and prescription to women, especially pregnant women with comorbidities. | (Martin 2018, Birhanu 2018) | **No or very minor concerns** | **No or very minor concerns** | **Moderate concerns:** All studies are directly relevant to review aim and represented 2 countries (Kenya, Ethiopia), including 1 low-income and 1 lower middle-income country. All perspectives came from health providers. | **Serious concerns:** 2 out 6 studies contributed to findings (1 thick, 1 thin data). | **Moderate Confidence** | No or very minor concerns on methodological limitations, no or very minor concerns on coherence, minor concerns on relevance (due to all studies coming from low income or lower middle-income countries and small number of countries) and serious concerns on adequacy (2 out 6 studies contributed). |

**References**

Birhanu Z, Chapleau GM, Ortolano SE, Mamo G, Martin SL, Dickin KL. Ethiopian women's perspectives on antenatal care and iron-folic acid supplementation: Insights for translating global antenatal calcium guidelines into practice. Matern Child Nutr [Internet]. 2018 Feb;14:e12424. Available from: https://onlinelibrary.wiley.com/doi/10.1111/mcn.12424

Kachwaha S, Nguyen PH, Tran LM, Avula R, Young MF, Ghosh S, et al. Specificity Matters: Unpacking Impact Pathways of Individual Interventions within Bundled Packages Helps Interpret the Limited Impacts of a Maternal Nutrition Intervention in India. J Nutr. 2022 Feb;152(2):612–29.

Martin SL, Seim GL, Wawire S, Chapleau GM, Young SL, Dickin KL. Translating formative research findings into a behaviour change strategy to promote antenatal calcium and iron and folic acid supplementation in western Kenya. Matern Child Nutr [Internet]. 2017 Jan 22;13(1):mcn.12233. Available from: https://onlinelibrary.wiley.com/doi/10.1111/mcn.12233 Martin 2017a 38

Martin SL, Omotayo MO, Chapleau GM, Stoltzfus RJ, Birhanu Z, Ortolano SE, et al. Adherence partners are an acceptable behaviour change strategy to support calcium and iron-folic acid supplementation among pregnant women in Ethiopia and Kenya. Matern Child Nutr [Internet]. 2017 Jul;13(3):e12331. Available from: https://onlinelibrary.wiley.com/doi/10.1111/mcn.12331 Martin 2017b 41

Martin SL, Wawire V, Ombunda H, Li T, Sklar K, Tzehaie H, et al. Integrating Calcium Supplementation into Facility-Based Antenatal Care Services in Western Kenya: A Qualitative Process Evaluation to Identify Implementation Barriers and Facilitators. Curr Dev Nutr [Internet]. 2018 Nov 1;2(11). Available from: https://academic.oup.com/cdn/article/doi/10.1093/cdn/nzy068/5078341 39

Omotayo MO, Martin SL, Stoltzfus RJ, Ortolano SE, Mwanga E, Dickin KL. With adaptation, the WHO guidelines on calcium supplementation for prevention of pre-eclampsia are adopted by pregnant women. Matern Child Nutr [Internet]. 2018 Apr;14(2):e12521. Available from: http://doi.wiley.com/10.1111/mcn.12521

Vestering A, Bekker MN, Grobbee DE, van der Graaf R, Franx A, Crombag NMT, et al. Views and preferences of medical professionals and pregnant women about a novel primary prevention intervention for hypertensive disorders of pregnancy: a qualitative study. Reprod Health [Internet]. 2019 May 2;16(1):46. Available from: http://www.ncbi.nlm.nih.gov/pubmed/31046778
