# Supplementary material for: Factors affecting the implementation of calcium supplementation strategies during pregnancy to prevent pre-eclampsia: a mixed-methods systematic review
Source: BMJ Open. 2023 Dec 22;13(12):e070677. doi: 10.1136/bmjopen-2022-070677 (PMC10749021; doi:10.1136/bmjopen-2022-070677)
Supplement: Supplementary data [file bmjopen-2022-070677supp006.pdf]

Appendix 6. Summary of Quantitative Findings

#	Summary of Quantitative review findings	Contributing quantitative studies	Quality ratings
**Women's factors**			
**1**	**Knowledge and learning**		
1.1	**Women's knowledge about pre-eclampsia or eclampsia**<br>No data supporting this theme from quantitative evidence.	No data supporting this theme from quantitative evidence.	No data supporting this theme from quantitative evidence.
1.2	**Information provision to women**<br>No data supporting this theme from quantitative evidence.	No data supporting this theme from quantitative evidence.	No data supporting this theme from quantitative evidence.
1.3	**Learning about calcium supplementation**<br>Quantitative evidence supported qualitative findings regarding women's learning on calcium supplements. In the context of an intervention implementing training to healthcare providers to reinforce calcium-related messages to women, women reported that they would take calcium in a future pregnancy and that they would recommend calcium to other pregnant women. Women were more likely to consume calcium supplements if they had higher knowledge of calcium benefits (Odds Ratio (OR) 11.7, 95% Confidence Interval (CI): 5.97-22.86) and higher general education (OR 2.59, 95% CI: 2.21-3.05). Higher adherence was also reported in those with higher education (OR 1.45, 95% CI: CI 1.31 to 1.60).(Liu et al., 2019) Higher nutrition knowledge was also associated with taking 6 times more calcium tablets. One paper, evaluating calcium supplement intake before and after nutritional interventions, showed an association between maternal knowledge of nutrition and higher calcium supplement intake (β ~31.9, 95% CI: 20.9, 43.0), however the paper also highlighted there were still large gaps between knowledge and practices, as the intake of calcium supplement tablets during 6 month was low, 82 ±66 out of the recommended 180 tablets.	(Liu et al., 2019; Nguyen et al., 2019, 2017; Thapa et al., 2016)	Four studies. 1 high quality, 1 low quality and 2 moderate quality studies.

**2**	**Believe about the intervention**		
2.1	**Fears about side-effects as barriers to calcium uptake among women**<br>Quantitative evidence supported qualitative findings as few women reported being discouraged to take the supplements by friends (4%) and elder women (3%).	(Martin et al., 2017)	1 Low quality study.
2.2	**Experiences of side effects**<br>Quantitative evidence supported the qualitative findings as some women reported experiencing side effects, usually related to gastrointestinal symptoms. The reported side-effects rates were usually low, 4% of women mentioning nausea or vomiting or constipation in one paper while in another paper 14.9% of women reported that side effects was the reason for missing a dose of IFA or calcium. These could cause supplement discontinuation or erratic supplement intake for one to 10 days after side effects were felt in around 5% of women.	(Baxter et al., 2014; Ghosh-Jerath et al., 2015; Shakya Shrestha et al., n.d.; Thapa et al., 2016)	Four studies. 1 high quality, 2 low quality and 1 very low quality study.
2.3	**Concerns of being stigmatized as HIV patient**<br>No data supporting this theme from quantitative evidence.	No data supporting this theme from quantitative evidence.	No data supporting this theme from quantitative evidence.
2.4	**Positive perceptions about calcium**<br>Quantitative evidence supported the qualitative findings that women's beliefs about the importance of calcium supplements to both the woman's and baby's health and on being able to consume it daily are associated with consuming calcium supplements. Positive beliefs about calcium supplements and self-efficacy were associated with taking calcium supplements (OR 4.6, 95% CI: 2.0 to 10.5) and with taking a higher number of supplements (OR 2.77, 95% CI: 1.68 to 4.57).	(Nguyen et al., 2019)	1 Moderate quality study.
**3**	**Calcium supplement characteristics and regimens**		
3.1	**Varying preferences about characteristics of calcium tablets**<br>Quantitative evidence supported qualitative findings regarding varying preferences calcium supplement's organoleptic properties. One paper which evaluate the impact of a program to implement calcium supplementation showed that most women (77%) reported preferences for conventional tablets that were easier to take and swallow, while the least preferred vehicle was unflavoured powder, as women dislike the taste.(Baxter et al., 2014) Another paper reported that conventional tablets had an acceptable taste (83.9% of women). Chewable tablets	(Baxter et al., 2014; Omotayo et al., 2018b; Thapa et al., 2016)	Three studies. 1 Low quality and 2 high quality studies.

	were preferred by most women (74%) in another paper. Some characteristics that women considered while taking the supplements include tablet's flavour, chewable or swallow, taken with water or not, smell, and size.		
3.2	**Adherence challenges due to routines**<br>In two quantitative studies, women preferred to take fewer tablets per day. However, in one study, women who were allocated to the study arm that used more frequent doses took more calcium overall.	(Baxter et al., 2014; Omotayo et al., 2018b, 2018a)	Three studies. 1 High quality, 1 moderate and 1 low quality studies.
**4**	**Daily routines and food insecurity**		
4.1	**Adherence challenges due to routines**<br>In quantitative evidence, women described busy work schedules and not being at home as also contributing to forgetting to consume their calcium supplements.	(Baxter et al., 2014; Shakya Shrestha et al., n.d.; Thapa et al., 2016)	Three studies. 1 High quality, 1 low and 1 very low-quality studies.
4.2	**Food insecurity as a barrier to calcium uptake**<br>Quantitative evidence supported qualitative findings that women with food security, high socio-economic status and living in urban areas are more likely to consume calcium supplements as compared to their counterparts. Food security was associated with consumption of 6 more calcium tablets.	(Liu et al., 2019; Nguyen et al., 2017)	Two studies. 1 High quality and 1 low quality studies.
**5**	**Strategies to improve use**		
5.1	**Implementation of reminders to promote adherence**<br>Quantitative evidence supported the qualitative findings that distribution of behaviour change materials to women, such as pill-taking calendars, were associated with increased adherence	(Omotayo et al., 2018b)	1 High quality study.
5.2	**Importance of family support and adherence partner implementation**<br>Quantitative evidence supported the qualitative findings that social support is important in encouraging women to consume calcium supplements. Women considered involving a husband, partner, or family in education sessions or appointing someone as the "adherence partner" to be an acceptable strategy for promoting adherence. Women often chose their husband (52%), older female relative (23%), children (14%), cousins or other relatives as adherence partners (8%), and were satisfied with the reminders and support received from their adherence partner. However, a randomised trial assessing adherence partners in improving calcium supplement intake showed that high social support, instead of	(Martin et al., 2017; Nguyen et al., 2019, 2018, 2017; Omotayo et al., 2018b)	Five studies. 3 high quality studies, 1 moderate and 1 low quality studies.

	adherence partners alone, was associated with higher adherence to calcium supplement (OR: 2.10; 95% CI: 1.32, 3.34). Women with high family support reported higher intake of calcium supplements (OR = 2.1).		
5.3	**Counselling facilitates calcium uptake**<br>Quantitative evidence extended qualitative findings where not only counselling, but also starting antenatal contacts at early gestational age, higher number of antenatal contacts, and receiving free calcium supplements were associated with higher calcium intake by women. One paper reports that women were 59 times more likely to consume calcium supplements if they had received them for free.	(Liu et al., 2019; Nguyen et al., 2019, 2017; Thapa et al., 2016)	Four studies. 2 High quality studies, 1 moderate and 1 low quality studies.
**Health care providers' factors**			
**6**	**Healthcare provider knowledge and training**		
6.1	**Varied knowledge about preeclampsia/eclampsia among providers**<br>No data supporting this theme from quantitative evidence.	No data supporting this theme from quantitative evidence.	No data supporting this theme from quantitative evidence.
6.2	**Inadequate training**<br>No data supporting this theme from quantitative evidence.	No data supporting this theme from quantitative evidence.	No data supporting this theme from quantitative evidence.
**7**	**Beliefs on the intervention**		
7.1	**Perceived overmedicalization when prescribing calcium supplements**<br>No data supporting this theme from quantitative evidence.	No data supporting this theme from quantitative evidence.	No data supporting this theme from quantitative evidence.
7.2	**Beliefs about calcium**<br>No data supporting this theme from quantitative evidence.	No data supporting this theme from quantitative evidence.	No data supporting this theme from quantitative evidence.
**8**	**Structural factors**		
8.1	**High workload, inadequate staffing, stock out, and lack of equipment**<br>Quantitative evidence extended qualitative findings by showing that in the context of a comprehensive integrated program including the implementation of job aids, training, guidelines, monitoring, and feedback session for healthcare providers, could overcome barriers in prescribing women with calcium supplements.	(Omotayo et al., 2018a)	1 Moderate study

References

Baxter, J.-A.B., Roth, D.E., Al Mahmud, A., Ahmed, T., Islam, M., Zlotkin, S.H., 2014. Tablets Are Preferred and More Acceptable Than Powdered Prenatal Calcium Supplements among Pregnant Women in Dhaka, Bangladesh. J. Nutr. 144, 1106–1112. https://doi.org/10.3945/jn.113.188524

Ghosh-Jerath, S., Devasenapathy, N., Singh, A., Shankar, A., Zodpey, S., 2015. Ante natal care (ANC) utilization, dietary practices and nutritional outcomes in pregnant and recently delivered women in urban slums of Delhi, India: an exploratory cross-sectional study. Reprod. Health 12, 20. https://doi.org/10.1186/s12978-015-0008-9

Liu, D., Cheng, Y., Dang, S., Wang, D., Zhao, Y., Li, C., Li, S., Lei, F., Qu, P., Mi, B., Zhang, R., Li, J., Zeng, L., Yan, H., 2019. Maternal adherence to micronutrient supplementation before and during pregnancy in Northwest China: a large-scale population-based cross-sectional survey. BMJ Open 9, e028843. https://doi.org/10.1136/bmjopen-2018-028843

Martin, S.L., Omotayo, M.O., Pelto, G.H., Chapleau, G.M., Stoltzfus, R.J., Dickin, K.L., 2017. Adherence-specific social support enhances adherence to calcium supplementation regimens among pregnant women. J. Nutr. 147. https://doi.org/10.3945/jn.116.242503

Nguyen, P.H., Frongillo, E.A., Sanghvi, T., Wable, G., Mahmud, Z., Tran, L.M., Aktar, B., Afsana, K., Alayon, S., Ruel, M.T., Menon, P., 2018. Engagement of Husbands in a Maternal Nutrition Program Substantially Contributed to Greater Intake of Micronutrient Supplements and Dietary Diversity during Pregnancy: Results of a Cluster-Randomized Program Evaluation in Bangladesh. J. Nutr. 148, 1352–1363. https://doi.org/10.1093/jn/nxy090

Nguyen, P.H., Kachwaha, S., Avula, R., Young, M., Tran, L.M., Ghosh, S., Agrawal, R., Escobar-Alegria, J., Patil, S., Menon, P., 2019. Maternal nutrition practices in Uttar Pradesh, India: Role of key influential demand and supply factors. Matern. Child Nutr. 15. https://doi.org/10.1111/mcn.12839

Nguyen, P.H., Sanghvi, T., Kim, S.S., Tran, L.M., Afsana, K., Mahmud, Z., Aktar, B., Menon, P., 2017. Factors influencing maternal nutrition practices in a large scale maternal, newborn and child health program in Bangladesh. PLoS One 12, e0179873. https://doi.org/10.1371/journal.pone.0179873

Omotayo, M.O., Dickin, K.L., Pelletier, D.L., Martin, S.L., Kung'u, J.K., Stoltzfus, R.J., 2018a. Feasibility of integrating calcium and iron-folate supplementation to prevent preeclampsia and anemia in pregnancy in primary healthcare facilities in Kenya. Matern. Child Nutr. 14, e12437. https://doi.org/10.1111/mcn.12437

Omotayo, M.O., Martin, S.L., Stoltzfus, R.J., Ortolano, S.E., Mwanga, E., Dickin, K.L., 2018b. With adaptation, the WHO guidelines on calcium supplementation for prevention of pre-eclampsia are adopted by pregnant women. Matern. Child Nutr. 14, e12521. https://doi.org/10.1111/mcn.12521

Shakya Shrestha, S., Adhikari, R., Tamrakar, S., Shrestha, R., Shrestha, A., n.d. Adherence to Iron, Folic Acid and Calcium Supplement and Factors Affecting it among the Antenatal Care Attending Women in a Tertiary Care Hospital: A Cross Sectional Study. Kathmandu Univ. Med. J. (KUMJ) 18, 186–192.

Thapa, K., Sanghvi, H., Rawlins, B., Karki, Y.B., Regmi, K., Aryal, S., Aryal, Y., Murakami, P., Bhattarai, J., Suhowatsky, S., K., T., H., S., B., R., Y.B., K., K., R., S., A., Y., A., P., M., J., B., S., S., 2016. Coverage, compliance, acceptability and feasibility of a program to prevent pre-eclampsia and eclampsia through calcium supplementation for pregnant women: An operations research study in one district of Nepal. BMC Pregnancy Childbirth. https://doi.org/10.1186/s12884-016-1033-6
