# Supplementary material for: Factors affecting the implementation of calcium supplementation strategies during pregnancy to prevent pre-eclampsia: a mixed-methods systematic review
Source: BMJ Open. 2023 Dec 22;13(12):e070677. doi: 10.1136/bmjopen-2022-070677 (PMC10749021; doi:10.1136/bmjopen-2022-070677)
Supplement: Supplementary data [file bmjopen-2022-070677supp007.pdf]

Appendix 7. COM-B Mapping Table

Behavior aimed: calcium supplementation use by women and health providers						
**No**	**COM-B Domain**	**TDF Domain***	**List of factors affecting calcium use**	**Stakeholders affected (women, health providers, partner)**	**Evidence Source (Quantitative/Qualitative)**	**Barriers or facilitators to calcium use**
**Women's factors**						
1	Capability	Know	Limited knowledge about preeclampsia/eclampsia	Women	Qualitative	
2	Capability	Know	Counselling on information about preeclampsia and calcium from their providers independent to woman risk	Women	Qualitative	
3	Capability	Know	High knowledge of calcium supplementation	Women	Quantitative	
4	Capability	Know	High education of women	Women	Quantitative	
5	Capability	Know	Symptoms were believed to be linked to evil attacks or nutritional deficiencies	Women	Qualitative	
6	Capability	Know	Inadequate information on preeclampsia/eclampsia and calcium from providers	Women	Qualitative	
7	Capability	Know	Receive adequate information about condition and calcium from their providers	Women	Qualitative	
8	Motivation	Bel Cons	Preeclampsia/eclampsia was not considered as a serious problem by women	Women	Qualitative	
9	Motivation	Bel Cons	Experiences and fears of side effects	Women	Qualitative and quantitative	
10	Motivation	Bel Cons	Assurance of calcium safety	Women	Qualitative	
11	Motivation	Bel Cons	Safety of calcium was not assured	Women	Qualitative	
12	Motivation	Bel Cons	Experienced and expected benefits from taking calcium and folic acid	Women	Qualitative and quantitative	
13	Motivation	Bel Cap	Belief that having essential information would help them making informed decisions	Women	Qualitative	
14	Motivation	Bel Cap	Belief that providers is a reliable source of information	Women	Qualitative	
15	Motivation	Bel Cap	Belief of being able to consume calcium daily	Women	Quantitative	
16	Motivation	Em	Valued information, education and communication (IEC) materials	Women	Qualitative	
17	Motivation	Em	Positive perceptions on calcium tablet characteristics (e.g. size, taste, smell)	Women	Qualitative and quantitative	
18	Motivation	Em	Women felt that 3-4 pills per day at multiple times were too many	Women	Quantitative and qualitative	
19	Motivation	Em	Inconvenience in taking pill daily	Women	Quantitative and qualitative	
20	Motivation	Em	Belief that one combined pill per day could ease burden	Women	Qualitative	
21	Motivation	Em	Feeling confident taking calcium after receiving adequate information from provider	Women	Quantitative	
22	Opportunity	Soc	Discouragement by family, neighbours and community in taking calcium	Women	Qualitative and quantitative	

23	Opportunity	Soc	Stigmatisation of having supplements and posters with HIV	Women	Qualitative	
24	Opportunity	Soc	Family support in consuming calcium	Women	Qualitative and quantitative	
25	Opportunity	Soc	Positive belief and experiences about 'adherence partners'	Women	Qualitative and quantitative	
26	Opportunity	Ev	Conflicting daily routine with taking calcium	Women	Qualitative and quantitative	
27	Opportunity	Ev	Food insecurity	Women	Qualitative and quantitative	
28	Opportunity	Ev	Early initiation and frequent antenatal visits	Women	Quantitative	
29	Opportunity	Ev	Receiving free calcium supplements	Women	Quantitative	
30	Opportunity	Ev	Reminder tools distribution, such as home based posters and pill-taking calendars	Women	Qualitative and Quantitative	
31	Opportunity	Ev	Universal free calcium distribution through antenatal care	Women	Quantitative	
**Providers factors**						
1	Capability	Know	Varied knowledge on pre-eclampsia/eclampsia	Providers	Qualitative	
3	Capability	Skills	Incontinuity and non-in-depth training about pre-eclampsia/eclampsia	Providers	Qualitative	
2	Capability	Skills	Felt inadequately trained	Providers	Qualitative	
4	Capability	Skills	Continuous training to manage the condition and address resistance from community	Providers	Qualitative	
5	Motivation	Bel Cons	Positive belief about calcium supplementation benefits	Providers	Qualitative	
6	Motivation	Bel Cons	Belief that preeclampsia/eclampsia was not a priority health concern	Providers	Qualitative	
7	Motivation	Bel Cons	Perceived over-medicalization to prescribe calcium to low risk women	Women and Providers	Qualitative	
8	Motivation	Bel Cons	Fears in generating anxiety on low-risk women	Providers	Qualitative	
9	Motivation	Bel Cons	Belief that women should get a chance to receive information regardless risk	Providers	Qualitative	
10	Opportunity	Ev	Providers felt that providing calcium would increase workload	Providers	Qualitative	
11	Opportunity	Ev	Inadequate number of staff providing care	Providers	Qualitative	
12	Opportunity	Ev	Comprehensive integrated program (job aids, training, guidelines, regular supplies)	Providers	Quantitative	

Barriers	
Facilitators	

* Know: Knowledge, Phys: Physical skills, Mem: Memory, attention and decision processes, Beh Reg: Behavioural regulation, Em: Emotion, Id: Social/professional role and identity, Bel Cons: Belief about consequences, Bel Cap: Belief about capabilities, Int: Intentions, Opt: Optimisms, Ev: Environmental context and resources, Soc: Social influences
