# Supplementary material for: Factors affecting the implementation of calcium supplementation strategies during pregnancy to prevent pre-eclampsia: a mixed-methods systematic review
Source: BMJ Open. 2023 Dec 22;13(12):e070677. doi: 10.1136/bmjopen-2022-070677 (PMC10749021; doi:10.1136/bmjopen-2022-070677)
Supplement: Author's manuscript [file bmjopen-2022-070677.draft_revisions.pdf]

BMJ Open

BMJ Open is committed to open peer review. As part of this commitment we make the peer review history of every article we publish publicly available.

When an article is published we post the peer reviewers' comments and the authors' responses online. We also post the versions of the paper that were used during peer review. These are the versions that the peer review comments apply to.

The versions of the paper that follow are the versions that were submitted during the peer review process. They are not the versions of record or the final published versions. They should not be cited or distributed as the published version of this manuscript.

BMJ Open is an open access journal and the full, final, typeset and author-corrected version of record of the manuscript is available on our site with no access controls, subscription charges or pay-per-view fees (http://bmjopen.bmj.com).

If you have any questions on BMJ Open's open peer review process please email info.bmjopen@bmj.com

BMJ Open

Factors affecting the implementation of calcium supplements strategies during pregnancy to prevent pre-eclampsia: a mixed methods systematic review

Journal:	*BMJ Open*
Manuscript ID	bmjopen-2022-070677
Article Type:	Original research
Date Submitted by the Author:	16-Dec-2022

Complete List of Authors:	Cormick, G; CONICET,<br>Moraa, Helen; University of Nairobi<br>Zahroh, Rana; University of Melbourne<br>Allotey, John; Univ Birmingham<br>Rocha, Thaís; University of Birmingham<br>Pena-Rosas, Juan-Pablo; Organisation mondiale de la Sante, Nutrition for Health and Development<br>Qureshi , Zahida ; University of Nairobi, Obstetrics and Gynaecology<br>Hofmeyr, G Justus ; University of the Witwatersrand Faculty of Health Sciences, Effective Care Research Unit<br>Mistry, Hema; University of Birmingham<br>Smits, Luc; Maastricht University<br>Vogel, J; Burnet Institute; World Health Organization, Reproductive Health and Research<br>Palacios , Alfredo ; Institute for Clinical Effectiveness and Health Policy<br>Gwako, George N. ; University of Nairobi, Obstetrics and Gynaecology ;<br>Abalos, E; Univ Western Australia<br>Larbi, Koiwah Koi; ACTION ON PREECLAMPSIA<br>Carroli, Guillermo; CREP<br>Riley, Richard; University of Birmingham<br>Snell, Kym IE; Keele University<br>Thorson, Anna; Human Reproduction Program/World Health Organization (Geneva)<br>Young, Taryn; Stellenbosch University, Centre for Evidence-based Health Care, Division Epidemiology and Biostatistics, Dept. of Global Health<br>Betran, Ana Pilar; World Hlth Org, Reproductive Health and Research<br>Thangaratinam, Shakila; Institute of Metabolism and Systems Research University of Birmingham<br>Bohren, Meghan ; University of Melbourne School of Population and Global Health, Centre for Health Equity

Keywords:	Hypertension < CARDIOLOGY, OBSTETRICS, PUBLIC HEALTH

SCHOLARONE™
Manuscripts

I, the Submitting Author has the right to grant and does grant on behalf of all authors of the Work (as defined in the below author licence), an exclusive licence and/or a non-exclusive licence for contributions from authors who are: i) UK Crown employees; ii) where BMJ has agreed a CC-BY licence shall apply, and/or iii) in accordance with the terms applicable for US Federal Government officers or employees acting as part of their official duties; on a worldwide, perpetual, irrevocable, royalty-free basis to BMJ Publishing Group Ltd ("BMJ") its licensees and where the relevant Journal is co-owned by BMJ to the co-owners of the Journal, to publish the Work in this journal and any other BMJ products and to exploit all rights, as set out in our licence.

The Submitting Author accepts and understands that any supply made under these terms is made by BMJ to the Submitting Author unless you are acting as an employee on behalf of your employer or a postgraduate student of an affiliated institution which is paying any applicable article publishing charge ("APC") for Open Access articles. Where the Submitting Author wishes to make the Work available on an Open Access basis (and intends to pay the relevant APC), the terms of reuse of such Open Access shall be governed by a Creative Commons licence – details of these licences and which Creative Commons licence will apply to this Work are set out in our licence referred to above.

Other than as permitted in any relevant BMJ Author's Self Archiving Policies, I confirm this Work has not been accepted for publication elsewhere, is not being considered for publication elsewhere and does not duplicate material already published. I confirm all authors consent to publication of this Work and authorise the granting of this licence.

For peer review only

Factors affecting the implementation of calcium supplements strategies during pregnancy to prevent pre-eclampsia: a mixed-methods systematic review

Gabriela Cormick [1,2,3], Helen Moraa [4], Rana Zahroh [5], John Allotey [6], Thais Rocha [6], Juan Pablo Peña-Rosas [7], Zahida Qureshi [8], G Justus Hofmeyr [9], Hema Mistry [10], Luc JM Smits [11], Joshua P. Vogel [12], Alfredo Palacios [13], Gwako George Nyakundi[4], Edgardo Abalos [14], Koiwah Koi-Larbi [15], Guillermo Carroli [16], Richard Riley [17], Kym Snell[17], Anna Thorson [18], Taryn Young [19], Ana Pilar Betran[18], Shakila Thangaratinam [6,17], Meghan A. Bohren[5] for I-CIP Collaborative Network

1. Centro de Investigaciones Epidemiológicas y Salud Pública (CIESP-IECS), CONICET, Buenos Aires, Argentina
2. Department of Mother and Child Health Research, Institute for Clinical Effectiveness and Health Policy (IECS-CONICET), Buenos Aires, Argentina
3. Departamento de Salud, Universidad Nacional de La Matanza (UNLAM), San Justo 1903, Argentina.
4. University of Nairobi
5. Gender and Women's Health Unit, Centre for Health Equity, School of Population and Global Health, University of Melbourne, Carlton, Victoria, Australia
6. WHO Collaborating Centre for Global Women's Health, Institute of Metabolism and Systems Research, University of Birmingham, Birmingham, UK
7. Department of Nutrition and Food Safety, World Health Organization, Geneva, Switzerland
8. Department of Obstetrics and Gynaecology, University of Nairobi, Nairobi Kenya.
9. Department of Obstetrics and Gynaecology, University of Botswana, Gaborone, Botswana; Effective Care Research Unit, University of the Witwatersrand and Walter Sisulu University, East London, South Africa.
10. Clinical Trials Unit, Warwick Medical School, University of Warwick, Coventry, CV4 7AL, United Kingdom; University Hospitals Coventry and Warwickshire NHS Trust, Coventry, CV2 2DX, United Kingdom
11. Department of Epidemiology, CAPHRI Care and Public Health Research Institute, Maastricht University, Maastricht, The Netherlands
12. Maternal, Child and Adolescent Health Program, Burnet Institute, 85 Commercial Road, Melbourne, 3000, Australia

For peer review only - http://bmjopen.bmj.com/site/about/guidelines.xhtml

13. Department of Health Technology Assessment and Health Economics, Institute for Clinical Effectiveness and Health Policy (IECS), Buenos Aires, Argentina; Centre for Health Economics (CHE), University of York, York, United Kingdom
14. Centro de estudios de Estado y Sociedad, Buenos Aires, Argentina
15. Action on preeclampsia Charity (APEC), Ghana
16. Centro Rosarino de Estudios Perinatales (CREP), Rosario, Argentina
17. Institute of Applied Health Research, University of Birmingham, Birmingham, UK
18. UNDP-UNFPA-UNICEF-WHO-World Bank Special Programme of Research Development and Research Training in Human Reproduction (HRP), Department of Sexual and Reproductive Health and Research, World Health Organization, Geneva, Switzerland.
19. Centre for Evidence-based Health Care, Division of Epidemiology and Biostatistics, Department of Global Health, Faculty of Medicine and Health Sciences, Stellenbosch University, Stellenbosch, South Africa
20. Birmingham Women's and Children's NHS Foundation Trust, Birmingham, UK

Corresponding author: Gabriela Cormick, email: gabmick@yahoo.co.uk

For peer review only

Abstract (300 words)

Objectives

Hypertensive disorders of pregnancy are among the leading causes of maternal and perinatal morbidity and mortality globally. The World Health Organization recommends daily calcium supplements from 20 weeks' gestation in populations with low dietary calcium intake to prevent pre-eclampsia. However, this recommendation is not implemented in many contexts. This systematic review aims to improve understanding of barriers and facilitators for consumption of calcium supplements during pregnancy to prevent pre-eclampsia, from the perspectives of women, community members, healthcare providers, and policymakers.

Methods

We conducted a mixed-method systematic review and searched MEDLINE, EMBASE, CINAHL, Global Health, and grey literature databases from database inception to 17 September 2022. We used the Theoretical Domains Framework to identify barriers and facilitators. We used the GRADE-CERQual (Confidence in the Evidence from Reviews of Qualitative Research) approach to assess confidence. Quantitative findings were thematically mapped to qualitative findings.

Results

Eighteen reports from nine studies are included in this review. From women's perspectives, barriers to using calcium supplements included knowledge about calcium-containing supplements and pre-eclampsia, fears and experiences of side effects, varying preferences for tablets, dosing, working schedules, being away from home and taking other supplements. Facilitating factors for women included receiving information regarding pre-eclampsia and safety of calcium supplement use from reliable sources, alternative dosing options, supplement reminders, and support from families and communities. Early initiation of antenatal care and free calcium supplements may help improve adherence. Facilitators of healthcare providers promoting calcium supplement use to pregnant women included, consistent messaging about benefits and risks of calcium, training, and ensuring adequate staffing and calcium supply is available.

Conclusion

Relevant stakeholders should consider the identified barriers and facilitators when formulating intervention and policies on calcium supplement use. This study findings can inform implementation, to ensure effective and equitable provision and scale-up of calcium interventions.

For peer review only - http://bmjopen.bmj.com/site/about/guidelines.xhtml

Key questions (summary box)

What is already known on this topic

- Existing research shows? that calcium-containing supplements during pregnancy for women living in areas of low dietary intake significantly reduces the risk of pre-eclampsia.
- The World Health Organization recommends calcium supplements of 1.5 to 2 grams of elemental calcium per day from 20 weeks' gestation for women living in populations with low dietary calcium intake.
- Despite these recommendations, translating WHO recommendations into national guidelines, policies and clinical practice has been slow due to a range of complex factors.

What this study adds

- We conducted a mixed-methods systematic review to understand what factors (e.g., barriers and facilitators) influence the use of calcium supplements during pregnancy.
- Women identified barriers to the use of calcium supplements as limited knowledge and insufficient information about safety of calcium supplements, symptoms, and risk of pre-eclampsia, fears and experiences of side effects, varying preferences on tablet characteristics and dosing, working schedules, being away from home and taking other supplements, food insecurity, stigma, and discouragement by family.
- Healthcare providers identified barriers to prescribing calcium to women during pregnancy as inadequate training to diagnose and treat pre-eclampsia, high workload and inadequate staffing, perceived overmedicalization, beliefs that pre-eclampsia is not a serious problem in their setting, and fear that prescribing calcium-containing supplements during pregnancy may generate anxiety for women.
- Potential solutions to these barriers include ensuring adequate information about pre-eclampsia and safe calcium use for women and providers, adequate staffing and stock, counselling with providers, early initiation and frequent antenatal contacts, options for daily doses, reminders, free supplements and support from families and communities.

How this study might affect research, practice, policy

- Findings from this study can inform implementation considerations to ensure effective and equitable scale-up of calcium supplements for women for a positive pregnancy experience.
- Prior to research or programme implementation, formative research should be conducted to ensure context-specific barriers and facilitators can be addressed and leveraged.

For peer review only - http://bmjopen.bmj.com/site/about/guidelines.xhtml

For peer review only

For peer review only - http://bmjopen.bmj.com/site/about/guidelines.xhtml

Background

Hypertensive disorders of pregnancy are among the leading causes of maternal and perinatal morbidity and mortality globally.(1) Pre-eclampsia is a hypertensive disorder of pregnancy characterised by hypertension developing after 20 weeks' gestation, combined with proteinuria or other new onset of maternal organ dysfunction, while eclampsia is a severe form of pre-eclampsia characterised by seizures.(2,3) Pre-eclampsia contribute to approximately 14% of the 300,000 maternal deaths worldwide annually.(4) Management of pre-eclampsia requires regular monitoring and evaluation of the woman and her baby to achieve an optimal timing of birth and prevent severe complications.(5) Preventive strategies are essential to reduce the burden of morbidity and mortality, especially in low- and middle-income countries (LMICs) where most complications occur.

The World Health Organization (WHO) recommends 1.5 to 2 grams a day should be offered from 20 weeks' gestation to women who are living in populations with low dietary calcium intake, especially those at high risk of developing pre-eclampsia (6). This is aligned with findings from a systematic review that reported calcium supplements during pregnancy compared to placebo may reduce the risk of pre-eclampsia by 55% (13 trials, 15,730 participants; Relative Risk (RR) 0.45, 95% CI: 0.31 to 0. 65).(7) Moreover, maternal death or severe morbidity was reduced by 20% with calcium supplements (four trials, 9,732 participants; RR 0.80, 95% CI 0.66 to 0.98). (7) The evidence base has since been updated multiple times, with WHO recommendation consistently revalidated up to 2018.(8)(9,10)

Despite the WHO recommendation, calcium supplement during pregnancy remains low in LMICs and rates of pre-eclampsia are not falling in regions where calcium supplementation is recommended.(10) Practical challenges to implementing WHO recommendations have been documented. For example, women need to take three, spaced tablets to achieve the requisite daily dose, and this needs be separated from timing of intake of other supplements (such as iron) to optimise calcium absorption.(11) In addition, antenatal care services need consistent supplies of calcium tablets, which can be hindered by logistical issues in supplement distribution and storage.(7) We conducted a mixed-methods systematic review aiming to improve understanding of the barriers and facilitators of calcium supplements during pregnancy to prevent pre-eclampsia, from the perspectives of women, families, community members, healthcare providers, and policymakers.

For peer review only

Methods

This mixed-methods review is reported according to Preferred Reporting Items for Systematic Reviews and Meta-Analyses (PRISMA) (Appendix 1) (12), Enhancing transparency in reporting the synthesis of qualitative research (ENTREQ) statement (Appendix 2) (13), and based on guidance from Cochrane Effective Practice and Organisation of Care.(14) The protocol was registered on PROSPERO (CRD42021239143).

Topic of interest and types of studies

We included studies that documented perspectives, perceptions and experiences of women who experienced or were at risk of pre-eclampsia and/or received calcium-containing supplements. We also included studies on the views of their partners or families, as well as studies on maternity healthcare providers (e.g., midwives, nurses, doctors) and other relevant stakeholders (e.g., facility managers, policymakers) involved in decisions on calcium supplements in pregnancy. There were no limitations imposed on geographical location or type of health facility. The timeframe for using calcium supplement is during pregnancy, independent of the gestational age.

We included primary qualitative, quantitative, and mixed-methods studies reporting implementation or use of calcium supplements in any presentations including powder, granule, chewable tablet, capsule, capsule with liquid filled, tablet, suspension, or powder for suspension. We did not include studies assessing the effects of calcium fortified foods or beverages. We excluded case reports or case series, letters, editorials, commentaries, reviews, study protocols, posters, and conference abstracts or other study sources that did not provide primary data.

Search methods for identification of studies

We searched MEDLINE, EMBASE, CINAHL, Global Health databases to identify eligible studies from inception to 17 September 2022. A search strategy was developed and adapted for each database (Appendix 3), using different terms for calcium and pregnancy. No limitations on publication date or language were imposed. Grey literature searches were also conducted using OpenGrey and Google. We checked reference lists of included studies to identify any relevant record not retrieved in the database search.

Selection of studies

After removing duplicates in EndNote, records were imported to Covidence for screening.(15) Two of the following authors (GC, GG, APB, RIZ, HM) independently assessed eligibility of each record by comparing titles and abstracts against the eligibility criteria. Full texts of potentially eligible reports were retrieved and assessed, with disagreements were resolved through discussion or consulting a third author. Reports emerging from the same study were collated and treated as one data source. Titles and abstracts of reports published in languages other English, French, and Spanish were translated through open-source software (Google Translate) to assess their eligibility. Had we identified any relevant titles or abstracts in

For peer review only - http://bmjopen.bmj.com/site/about/guidelines.xhtml

languages other than English, French and Spanish, we planned to seek formal translation of the full texts from a native speaker.

Data extraction and assessing the methodological limitations

Using a pre-designed form, two reviewers (GC, RIZ) independently extracted data from included studies on study characteristics (setting, sample size, characteristics of participants, objectives), design (data collection and analysis methods), qualitative data (themes, findings, and quotations) and quantitative data (data source, outcome measures, results, measures of compliance or uptake).

All included studies underwent quality appraisal by two authors (GC, HM, RIZ). As the review included quantitative, qualitative and mixed-methods studies, we used the Mixed Methods Appraisal Tool (MMAT), which produces a single quality rating on the basis of: aims, methodology, design, recruitment, data collection, blinding, data analysis, selective reporting, reflexivity, ethical considerations, results, research contribution, and other sources of bias.(16) Appraisal of study quality was used to inform data analysis, and not to exclude studies.

Quality appraisal, analysis and assessing confidence

We conducted a preliminary qualitative synthesis using a thematic analysis approach.(17) Thematic analysis is a valuable method in analysing qualitative data to examine perspectives, preferences, experiences, acceptability, feasibility, and other factors that can influence implementation.(17) The analysis begins with initial readings to build our familiarity with the data. Two reviewers (GC, HM) independently conducted line-by-line coding of findings of two qualitative studies.(18,19) From this we developed the qualitative codebook, which was then used to code all other included studies. Next, we generated analytical themes and interpretations to explore relationships within and across studies. This was achieved by organising codes into a hierarchy, and identifying barriers and facilitators between study characteristics and findings or exploring different findings across studies. Once qualitative themes were generated, a summary of qualitative findings was developed. Quantitative findings were then narratively mapped to qualitative themes to explore areas of convergence or divergence. ATLAS.ti was used to manage data analysis.

After the thematic analysis, we mapped the qualitative themes to the Theoretical Domains Framework (TDF) and Capability, Opportunity, and Motivation of Behaviour (COM-B) models.(20,21) TDF and COM-B are interrelated behaviour change models which can guide implementation research and intervention design in understanding barriers and facilitators of intended behaviours. We used TDF and COM-B to explore barriers and facilitators of healthcare providers and women to use calcium supplements during pregnancy using evidence-based behaviours to identify potential behaviour change intervention strategies.

We used the GRADE-CERQual (Confidence in the Evidence from Reviews of Qualitative research) approach to assess confidence in each qualitative finding (22). GRADE-CERQual assessed confidence based on four key components: methodological limitations (23),

For peer review only - http://bmjopen.bmj.com/site/about/guidelines.xhtml

coherence (24), adequacy (25), relevance (26). After assessing each of the four components, we assessed the overall confidence (22) as high, moderate, low, or very low.

Patient and Public Involvement

Patients were not directly involved in design or development of this systematic review.

Results

We included 18 reports from nine studies (Figure 1). Included reports were published in English between 2014 and 2022. Seven reports were the results of the Micronutrient Initiative-Cornell University Calcium (MICa) trial, which aimed to compare the effect of two calcium dosing strategies and adherence to related recommendations in Kenya and Ethiopia (19,27–32). Four reports (33–36) emerged from the Alive & Thrive initiative, while the remaining seven reports reported seven separate studies (18,37–42). Out of 18 reports, 11 were quantitative (27,32,42,34–41), four were qualitative (18,19,28,29) and three were mixed-methods (30,31,43). Detailed study characteristics can be found in Table 1.

For peer review only - http://bmjopen.bmj.com/site/about/guidelines.xhtml

Table 1. Characteristics of included studies

#	Authors	Year	Title	Country	Study designs	Sample Size	Participants	Project
	**Qualitative**							
**1**	Vestering	2019	Views and preferences of medical professionals and pregnant women about a novel primary prevention intervention for hypertensive disorders of pregnancy: a qualitative study	Netherlands	Phenomology	8 healthcare providers, 25 women	Healthcare providers and pregnant women	
**2**	Birhanu	2016	Ethiopian women's perspectives on antenatal care and iron-folic acid supplements: Insights for translating global antenatal calcium guidelines into practice	Ethiopia	Phenomology	20 women, 22 healthcare providers	Pregnant women and healthcare providers	Micronutrient Initiative-Cornell University Calcium (MICa) trial
**3**	Martin	2016	Translating formative research findings into a behaviour change strategy to promote antenatal calcium and iron and folic acid supplements in western Kenya	Kenya	Phenomology	22 women, 20 healthcare providers	Pregnant women and healthcare providers	Micronutrient Initiative-Cornell University Calcium (MICa) trial
**4**	Martin	2018	Integrating Calcium Supplements into Facility-Based Antenatal Care Services in Western Kenya: A Qualitative Process Evaluation to Identify Implementation Barriers and Facilitators	Kenya	Phenomology	7 healthcare providers, 32 pregnant women, 20 adherence partners.	Healthcare providers, pregnant women, adherence partners	Micronutrient Initiative-Cornell University Calcium (MICa) trial
	**Mixed methods**							
**1**	Omotayo	2017	With adaptation, the WHO guidelines on calcium supplements for prevention of pre-eclampsia are adopted by pregnant women	Kenya	Trials of improved practices	38 pregnant women	Pregnant women	Micronutrient Initiative-Cornell University Calcium (MICa) trial
**2**	Martin	2017	Adherence partners are an acceptable behaviour change strategy to support calcium and iron-folic acid supplements among pregnant women in Ethiopia and Kenya	Kenya and Ethiopia	Trials of improved practices	85 pregnant women, 50 in Ethiopia, 35 in Kenya	Pregnant women	Micronutrient Initiative-Cornell University Calcium (MICa) trial
**3**	Kachwaha	2022	Specificity Matters: Unpacking Impact Pathways of Individual Interventions within Bundled Packages Helps Interpret the Limited Impacts of a Maternal Nutrition Intervention in India	India	Mixed methods	~500 healthcare providers and supervisors, and 20 block level staffs	healthcare providers, supervisors and facility staffs	Alive & Thrive (A&T) trial (NCT03378141)
`	**Quantitative**							
**1**	Martin	2017	Adherence-specific social support enhances adherence to calcium supplements regimens among pregnant women	Kenya	RCT Secondary analysis	1036 pregnant women	Pregnant women	Micronutrient Initiative-Cornell University Calcium (MICa) trial
**2**	Baxter	2014	Tablets Are Preferred and More Acceptable Than Powdered Prenatal Calcium Supplements among Pregnant Women in Dhaka, Bangladesh	Bangladesh	Modified discrete-choice trial	132 pregnant women	Pregnant women	
**3**	Thapa	2016	Coverage, compliance, acceptability, and feasibility of a program to prevent pre-eclampsia and eclampsia through calcium supplements for pregnant women: an operations research study in one district of Nepal	Nepal	Prospective collection and secondary analysis of monitoring data captured by the MOHP	1240 women	Pregnant and postpartum women, health facilities, female community health volunteers	
**4**	Nguyen	2019	Maternal nutrition practices in Uttar Pradesh, India: Role of key influential demand and supply factors	India	Baseline household survey	667 pregnant women and 1835 recently delivered women	Pregnant women and recently delivered women	Alive & Thrive (A&T) trial (NCT03378141)
**5**	Omotayo	2018	Feasibility of integrating calcium and iron-folate supplements to prevent pre-	Kenya	Process evaluatio	990 women and 16	Pregnant women,	Micronutrient Initiative-Cornell

For peer review only

			eclampsia and anemia in pregnancy in primary healthcare facilities in Kenya		n study adopting program impact pathway	facilities (unclear how many healthcare providers participated)	healthcare providers, community healthcare providers	University Calcium (MICa) trial
**6**	Nguyen	2017	Factors influencing maternal nutrition practices in a large scale maternal, newborn and child health program in Bangladesh	Bangladesh	Cross-sectional study	600 pregnant women and 2000 recently delivered women	Pregnant women and recently delivered women	Alive & Thrive (A&T) trial (NCT02745249)
**7**	Nguyen	2018	Engagement of husbands in a maternal nutrition program substantially contributed to greater intake of micronutrient supplements and dietary diversity during pregnancy: Results of a cluster-randomized program evaluation in Bangladesh	Bangladesh	Cluster randomised control trial with cross-sectional household surveys	1000 women and 70% of their husbands	Women and husbands	Alive & Thrive (A&T) trial (NCT02745249)
**8**	Liu	2019	Maternal adherence to micronutrient supplements before and during pregnancy in Northwest China: A large-scale population-based cross-sectional survey	China	Cross-sectional study	30,027 women	Women aged from 16 to 49 years who were pregnant between 2010 and 2013 and had specific pregnancy outcomes before the survey	
**9**	Shakya Shrestha	2020	Adherence to iron, folic acid and calcium supplement and factors affecting it among the antenatal care attending women in a tertiary care hospital: A cross sectional study	Nepal	Cross-sectional study	191 pregnant women	Pregnant women	
**10**	Ghosh-Jareth	2015	Ante natal care (ANC) utilization, dietary practices and nutritional outcomes in pregnant and recently delivered women in urban slums of Delhi, India: an exploratory cross-sectional study	India	Cross-sectional study	184 pregnant women and 160 recently delivered women	Pregnant women and recently delivered women	ANCHUL (Ante Natal and Child Healthcare in Urban Slums) trial
**11**	Bora	2022	Coverage of antenatal iron-folic acid and calcium distribution during pregnancy and their contextual determinants in the northeastern region of India	India	Cross-sectional study	3,097,274 pregnant women	Pregnant women	Distribution of calcium under India National Health Mission

For peer review only

Three studies aimed to evaluate the implementation of calcium supplements in pregnancy (five reports) (29,30,37–39). Four studies evaluated the incorporation of calcium supplement recommendation to other recommended supplements taken during pregnancy, including aspirin (1 report) (18), and iron and folic acid supplements (7 reports) (19,27,28,31,32,41,43). Two studies focused on general nutritional practices during pregnancy (4 reports) (34–36,42) and one study on all types of micronutrient supplements used before and during pregnancy (1 report) (40).

The studies were conducted in seven different countries across four regions. One study (7 reports) was conducted in the African Region: Kenya and Ethiopia (19,27–32). Six studies in the Southeast Asia Region: one study in Bangladesh and India (five reports) (34–37,43), one study in Bangladesh (1 report) (38), two studies in India (two reports) (37,42) , and two studies

For peer review only - http://bmjopen.bmj.com/site/about/guidelines.xhtml

in Nepal (2 reports) (39,41). The remaining two studies were conducted in the Western Pacific Region: China (40), and European Region: Netherlands (18).

All four qualitative reports involved pregnant women and healthcare providers (3 reports) (18,19,28), while one report also included adherence partners to support and remind women to consume medication (1 report) (29). Two mixed-methods reports included pregnant women (30,31) and one included healthcare providers and facility staff (43). Among the ten quantitative reports, eight included pregnant women or women who had recently given birth (27,34,36–38,40–42), two included healthcare providers (32,39) and one included both women and their husbands (35).

Results of critical appraisal of included studies is available in Appendix 4. The main areas of concern for qualitative studies were an unclear or partial description of reflexivity and limited information regarding ethical considerations. For the quantitative studies, main concerns were regarding the appropriateness of measurement tools, lack of detail regarding nonresponse bias, and insufficient information regarding statistical analysis.

Qualitative and quantitative synthesis

We identified five themes related to factors affecting women's use of calcium supplements. This included: 1) women's existing knowledge and learning; 2) women's beliefs about calcium supplements; 3) calcium supplement characteristics and dose regimens; 4) challenges due to daily routines and food insecurity; and 5) strategies to improve the use of calcium. We also identified three themes related to factors affecting healthcare providers' prescription of calcium supplements: 1) health provider knowledge and training, 2) their beliefs about calcium supplements, 3) Adequacy of resources on site. Across all these themes there were 19 qualitative findings (Table 2: Summary of qualitative findings): 11 findings were high confidence, six were moderate confidence and two were low confidence (Appendix 5: Evidence profile).

For peer review only - http://bmjopen.bmj.com/site/about/guidelines.xhtml

Table 2. Summary of qualitative findings

No	Summary of qualitative review findings	Studies	CERQual assessment	Explanation of overall assessment
**1. Women's knowledge and learning**				
**1.1**	**Women's knowledge about pre-eclampsia or eclampsia**: Most women had limited knowledge about pre-eclampsia or eclampsia, and these conditions were not typically considered by women to be a serious problem. Symptoms of pre-eclampsia such as swollen feet, severe headache, blurred vision and vomiting were considered normal signs and symptoms in pregnancy, while seizures were associated with evil attacks or nutritional deficiencies.	(18,19,28,33–36,38,39,41,42)	**High confidence**	Due to no or very minor concerns on coherence, minor concerns on methodological limitations (reflexivity and ethics), moderate concerns on relevance (1 out of 3 studies are indirectly relevant to our review aim and small number of countries), and minor concerns of adequacy.
**1.2**	**Information provision to women:** Women felt they did not receive adequate information during pregnancy from healthcare providers about pre-eclampsia or eclampsia and calcium supplements. They described wanting to be given more information, regardless of their pre-eclampsia risk status. Women believed that having this essential information could help them to make informed choices and actively participate in their care. There were, however, mixed opinions from healthcare providers - some feared that more information could generate anxiety for women, while others were more supportive of providing information.	(18,29)	**Low confidence**	Minor concerns on methodological limitations (ethics and reflexivity), moderate concerns on coherence (no clear understanding on why some health providers worry in generating anxiety to women while the others are not), moderate concerns on relevance (small number of countries), and serious concerns on adequacy.
**1.3**	**Learning about calcium supplements**: Women typically learned about dietary calcium, including pre-eclampsia and eclampsia symptoms, from healthcare providers. They considered healthcare providers to be the most trusted and reliable source of information, and reported feeling confident about taking calcium-containing supplements after receiving adequate information from them. Women also appreciated receiving information on calcium supplements and pre-eclampsia or eclampsia via information, education, and communication (IEC) materials like videos, media, and trusted websites.	(18,19,29)	**High confidence**	Due to no or very minor concerns on coherence, minor concerns on relevance (small number of countries), minor concerns on methodological limitations (reflexivity and ethics), and minor concerns on adequacy (3 out of 6 contributed, 1 moderate thick and 2 thin data).
**2. Women's beliefs about calcium supplements in pregnancy**				
**2.1**	**Fears about side-effects as a barrier to calcium supplements uptake**: Women's fears about the side effects of calcium supplements affected their adherence. Women highlighted that assurance of safe use of calcium supplement is a key facilitator to consistent use. However, some women felt safety was not assured by healthcare providers, especially when calcium supplements were perceived as "experimental". Women had also received messages from their families or communities that any pills consumed during pregnancy could be harmful.	(18,19,29,30)	**High Confidence**	Due to no or very minor concerns on coherence, minor concerns on relevance (small number of countries), minor concerns on adequacy (small number of studies contributing to the qualitative evidence synthesis), and minor concerns on methodological limitations (reflexivity and ethics).
**2.2**	**Women's experiences of side-effects**: Some women reported experiencing side effects after taking calcium and iron-folic acid supplements, such as dizziness, vomiting, nausea, stomach aches, loss of appetite, tiredness, diarrhoea, bloating, and burping, yet noted that side effects subsided with time. Women also reported that they continued consuming calcium despite these side effects.	(19,29,30)	**High Confidence**	Due to no or very minor concerns on coherence, minor concerns on methodological limitations (ethics and reflexivity), moderate concerns on relevance (only low and lower middle-income country and small number of countries) and minor concerns on adequacy
**2.3**	**Concerns about being stigmatized as HIV patients**: Women expressed concerns that if they ingested calcium supplements, they could be stigmatized as HIV patients, which was a reported barrier to use. Some women were afraid of being stigmatized as their community often associated nutritional supplement consumption and accompanying reminder posters with HIV.	(18,29)	**Moderate Confidence**	Due to no or very minor concerns on methodological limitations or concerns on coherence, minor concerns on relevance (small number of countries), and serious concerns on adequacy.
**2.4**	**Positive perceptions of calcium supplements**: Women reported that both their perceptions about expected benefits and previous experiences of taking calcium and iron-folic acid supplements were facilitators of use. Women believed that consuming pills could compensate for sub-optimal nutrition during pregnancy, and that supplements during pregnancy would help keep their baby safe.	(18,19,29–31)	**High Confidence**	Due to no or very minor concerns on coherence, minor concerns on methodological limitations (ethics and reflexivity), minor concerns on relevance (1 study indirectly relevant to review aim and small number of countries) and adequacy (small number of studies contributing to the qualitative evidence synthesis).
**3. Calcium supplement characteristics and regimens**				
**3.1**	**Varying preferences about characteristics of calcium tablets**: Positive perceptions about the characteristics of the calcium tablet played a role in motivating women to take it. Some women preferred the chewable, sweet-tasting tablets that could be swallowed without water, while others preferred the hard tablets which were smaller in size, had no smell, and needed to be taken with water. Based on individual preference, the taste, smell, size, and convenience affected calcium supplement use.	(19,30)	**Moderate confidence**	Due to no or very minor concerns on coherence, minor concerns on methodological limitations (reflexivity), moderate concerns on relevance (all low or lower middle-income countries and small number of countries) and moderate concerns on adequacy
**3.2**	**Supplement regimen as a barrier to use:** Women described that they feel overwhelmed with the number of calcium tablets they had to take each day, especially women with other comorbidities who needed to take additional medications for their health condition s. Women felt that 3-4 pills per day at multiple times was overly onerous and preferred if they were combined into one pill.	(19,29,30)	**High Confidence**	No or very minor concerns on coherence, minor concerns on methodological limitations (reflexivity), minor concerns on adequacy (3 out of 6 studies contributed with moderate to thick data), and moderate concerns on relevance (small number of countries).
**4**	**Daily routines and food insecurity**			

For peer review only - http://bmjopen.bmj.com/site/about/guidelines.xhtml

4.1	**Adherence challenges due to routine**: Adherence to calcium supplements consumption was challenging for some women because of conflicting activities in their daily routine, such as consuming other medications, traveling, being away from home, and household chores, which can lead them to forget to take calcium.	(19,29,30)	**High Confidence**	Due to no or very minor concerns with coherence, minor concerns on methodological limitations (reflexivity), moderate concerns on relevance (small number of countries) and moderate concerns on adequacy.
4.2	**Food insecurity as a barrier to calcium uptake**: Women believed that adequate food was necessary to be able to take the supplements, to avoid nausea. They perceived it as normal to eat before consuming any medication. However, women reported that food insecurity was a critical barrier to calcium uptake.	(19,29)	**Moderate Confidence**	Due to no or very minor concern with coherence, minor concerns on methodological limitations (reflexivity), moderate concerns on adequacy (2 out 6 studies contributed with 1 thick and 1 thin data), and moderate concerns on relevance (small number of countries).
**5. Strategies to improve the use of calcium supplements**				
5.1	**Implementation of reminders to promote adherence**: Women and healthcare providers perceived reminders as beneficial in promoting women's adherence to consuming calcium supplements. Several reminder strategies were described as useful by women and healthcare providers, such as home-based posters, calendars with illustrations and daily reminders, and integrating supplement consumption into women's daily routine, such as mealtimes.	(19,28–30)	**High Confidence**	Due to no or very minor concerns on methodological limitations or coherence, minor concerns on adequacy (small number of studies contributing to qualitative evidence synthesis), moderate concerns on relevance (due to all studies coming from low income or lower middle-income country and small number of countries).
5.2	**Importance of family support and 'adherence partner' implementation:** Having family support was instrumental to pregnant women adhering to calcium supplement use. This could be leveraged by notifying family members on the importance of calcium, and appointing someone to be an "adherence partner" or "pill buddy" to help remind her to take it. Both women and healthcare providers were positive about adherence partners in providing support in terms of encouraging them to take the supplements, providing food, helping them around the house, providing emotional support, improving family relationships, and thereby increasing partner or husband involvement.	(28–31)	**High Confidence**	No or very minor concerns on methodological limitations or coherence, minor concerns on adequacy (small number of studies contributing to qualitative evidence synthesis), and moderate concerns on relevance (due to all studies coming from low income or lower middle-income country only, 2 of 5 studies has indirectly relevant aim, and small number of countries).
5.3	**Counselling facilitates calcium supplements uptake:** Both women and healthcare providers acknowledged that counselling women on the benefits of calcium was a motivator to calcium supplements uptake. Women valued the discussion they have with healthcare providers and felt more confident to take calcium supplements when they received counselling and information and pre-eclampsia or eclampsia and the benefits of calcium from their healthcare providers.	(19,29)	**Moderate Confidence**	No or very minor concerns on methodological limitations or coherence, moderate concerns on relevance (due to all studies coming from low or lower middle-income country and small number of countries) and moderate concerns on adequacy
**6. Healthcare provider knowledge and training**				
6.1	**Varied knowledge about pre-eclampsia or eclampsia among healthcare providers** : Healthcare providers' knowledge about pre-eclampsia and eclampsia was varied. Some felt that pre-eclampsia and eclampsia is not a priority health concern in their area and reported never having encountered any case.	(19,28)	**Moderate Confidence**	Due to no or very minor concerns on methodological limitations and coherence, moderate concerns on relevance (due to all studies coming from low or lower middle-income countries and small number of studies) and moderate concerns on adequacy
6.2	**Inadequate training to diagnose and treat pre-eclampsia and eclampsia:** While some healthcare providers mentioned that training about pre-eclampsia or eclampsia and calcium supplements was adequate, others reported that their training lacked depth and continuity, and thus felt unprepared to diagnose it and offer information these conditions and the use of calcium for prevention. Healthcare providers expressed the need to have more and continuous training on managing pre-eclampsia, as well as time to address concerns or resistance from the community.	(19,28,29)	**High Confidence**	No or very minor concerns on methodological limitations, coherence, moderate concerns on relevance (due to all studies coming from low income or lower middle-income country and small number of countries) and minor concerns on adequacy (3 out of 6 studies contributed with 2 thick and 1 thin data).
**7. Beliefs about the intervention**				
7.1	**Perceived overmedicalization when prescribing calcium supplements**: Both healthcare providers and women perceived that prescribing more tablets to "low-risk" women during pregnancy was a form of over-medicalization of pregnancy. However, some healthcare providers felt that calcium supplements were a way to prevent further medicalization due to pre-eclampsia-related complications.	(18)	**Low confidence**	No or very minor concerns on coherence, minor concerns on methodological limitations (reflexivity and ethics), serious concerns on relevance (evidence coming from high income country only), serious concerns on adequacy (1 out of 6 study contributed).
7.2	**Beliefs about calcium supplements**: Healthcare providers generally had positive beliefs about calcium supplements and there was optimism that calcium could be delivered through antenatal care healthcare providers. Some facilitators motivating healthcare providers to prescribe calcium supplements included their beliefs in its prevention value and expected benefits, that women liked the calcium supplements and experienced benefits from it, and a perceived lack of knowledge on how to treat pre-eclampsia which motivated healthcare providers to side towards prevention.	(18,19,29)	**High Confidence**	No or very minor concerns on coherence, minor concerns on relevance (small number of countries), minor concerns on methodological limitations (ethics and reflexivity), and minor concerns on adequacy (3 out of 6 studies contributed with 2 thick and 1 thin data).
**8. Structural factors**				
8.1	**High workload, inadequate staffing, stock out, and lack of equipment**: healthcare providers felt that their workload increased by including calcium supplements in antenatal care for pregnant women. Healthcare providers reported existing inadequate staffing, yet they needed to provide additional counselling and prescription to women, especially pregnant women with comorbidities.	(19,29)	**Moderate Confidence**	No or very minor concerns on methodological limitations or coherence, minor concerns on relevance (due to all studies coming from low income or lower middle-income countries and small number of countries) and serious concerns on adequacy (2 out 6 studies contributed).

For peer review only - http://bmjopen.bmj.com/site/about/guidelines.xhtml

Women's knowledge and learning

Women's knowledge about pre-eclampsia

Most women had limited knowledge about pre-eclampsia, and these conditions were not typically considered by women to be a serious problem. Symptoms of pre-eclampsia such as swollen feet, severe headache, blurred vision and vomiting were considered normal signs and symptoms in pregnancy, while seizures were associated with evil attacks or nutritional deficiencies (1.1 – High confidence) (18,19,28). Women and healthcare providers from Ethiopia and Kenya mentioned that there was no local language for pre-eclampsia, which made it difficult for healthcare providers to explain the condition to women, and served as a barrier in providing adequate knowledge to women and encouraging them to use calcium supplements. (19,28)

Quantitative evidence extended the understanding of qualitative findings, where higher knowledge of calcium benefits (Odds Ratio (OR) 11.7, 95% Confidence Interval (CI) 5.97-22.86) (34,36) and higher general education of women (OR 2.59, 95%CI 2.21-3.05) (34,36,40) were shown to be associated with higher calcium supplement intake. However, one study that evaluated calcium supplement intake before and after other nutritional interventions shows that there were large gaps between knowledge and practices (36).

Information provision to women

Women felt they did not receive adequate information during pregnancy from healthcare providers about pre-eclampsia and calcium supplements. They described wanting to be given more information, regardless of their pre-eclampsia risk status. Women believed that having this essential information could help them to make informed choices and actively participate in their care. There were, however, mixed opinions from healthcare providers - some feared that more information could generate anxiety for women, while others were more supportive of providing information (1.2 – *Low confidence*) (18,29). Healthcare providers were worried that sharing information about pre-eclampsia, especially with low-risk pregnant women, could lead to anxiety and make "uncomplicated pregnancy more stressful" (18). Healthcare providers viewed their role as informants, but not as decision-makers for women. They believed that it should be women's choice to decide whether to consume calcium supplements or not (18). Women and healthcare providers mentioned that the scope of information provided to women should include symptoms of pre-eclampsia as well as effectiveness, benefits, and safety of calcium-containing supplements (18).

Learning about calcium supplements

Women typically learned about dietary calcium, including pre-eclampsia symptoms, from healthcare providers. They considered healthcare providers to be the most trusted and reliable source of information, and reported feeling confident about taking calcium-containing supplements after receiving adequate information from them. Women also appreciated receiving information on calcium supplements and pre-eclampsia via information, education, and communication (IEC) materials like videos, media, and trusted

For peer review only - http://bmjopen.bmj.com/site/about/guidelines.xhtml

websites (1.3 – *High confidence*) (18,19,29). Quantitative evidence supported qualitative findings regarding women's learning on calcium supplements. In the context of an intervention implementing training to healthcare providers to reinforce calcium-related messages to women, women reported that they would take calcium in a future pregnancy and that they would recommend calcium to other pregnant women (39).

Women's beliefs about calcium supplements in pregnancy

Fears about side-effects as a barrier to calcium supplements uptake

Women's fears about the side effects of calcium supplements affected their adherence. Women highlighted that assurance of safe use of calcium supplement is a key facilitator to consistent use. However, some women felt safety was not assured by healthcare providers, especially when calcium supplements were perceived as "experimental". Women had also received messages from their families or communities that any pills consumed during pregnancy could be harmful. (2.1 – *High confidence*) (18,19,29,30). Quantitative evidence supported qualitative findings that women could be discouraged by their family and community in consuming calcium supplements as it might affect baby's condition (27).

Women's experiences of side-effects

Some women reported experiencing side effects after taking calcium and iron-folic acid supplements, such as dizziness, vomiting, nausea, stomach aches, loss of appetite, tiredness, diarrhoea, bloating, and burping, yet noted that side effects subsided with time. Women also reported that they continued consuming calcium despite these side effects (2.2 – *High confidence*) (19,29,30). Quantitative evidence supported the qualitative findings as women reported experiencing side effects, usually related to gastrointestinal symptoms such as nausea, vomiting, and constipation (38,39,41,42). Those who reported stopping supplement intake after experiencing side effects did so between one to 10 days after side effects were felt (41).

Concerns about being stigmatized as HIV patients

Women expressed concerns that if they ingested calcium supplements, they could be stigmatized as HIV patients, which was a reported barrier to use. Some women were afraid of being stigmatized as their community often associated nutritional supplement consumption and accompanying reminder posters with HIV (2.3 – *Moderate confidence*) (18,29).

Positive perceptions of calcium supplements

Women reported that both their perceptions about expected benefits and previous experiences of taking calcium and iron-folic acid supplements were facilitators of use. Women believed that consuming pills could compensate for sub-optimal nutrition during pregnancy, and that supplements during pregnancy would help keep their baby safe (2.4 – *High confidence*) (18,19,29–31). Women also reported reduced cravings to consume soil (pica) during pregnancy (a cultural practice or cravings characterised by recurrent ingestion of

For peer review only - http://bmjopen.bmj.com/site/about/guidelines.xhtml

unusually high amounts of soil, and is related to iron deficiency anaemia, which is common during pregnancy) (29). Furthermore, some women appreciated the emphasis of "prevention" when encouraging consumption of supplements consumption (19,31). Quantitative evidence supported the qualitative findings that women's beliefs about the importance of calcium supplements to both the woman's and baby's health and on being able to consume it daily are associated with consuming calcium supplements (34).

Calcium supplement characteristics and regimens

Varying preferences about characteristics of calcium tablets

Positive perceptions about the characteristics of the calcium tablet played a role in motivating women to take it. Some women preferred the chewable, sweet-tasting tablets that could be swallowed without water, while others preferred the hard tablets which were smaller in size, had no smell, and needed to be taken with water. Based on individual preference, the taste, smell, size, and convenience affected calcium supplement use (3.1 – *Moderate confidence*) (19,30).

Quantitative evidence supported qualitative findings regarding varying preferences calcium supplement's organoleptic properties. In one study, most women reported preferences for conventional tablets that were easier to consume and swallow, and some women in the other studies preferred chewable tablets (30). Some characteristics that women considered while taking the supplements include tablet's flavour, chewable or swallow, consumption with water or not, smell, and size (30,38,39). The most frequently reported reason for stopping supplement consumption was a dislike of the taste (38).

Supplement regimen as a barrier to use

Women described that they feel overwhelmed with the number of calcium tablets they had to take each day, especially women with other comorbidities who needed to take additional medications for their health conditions. Women felt that 3-4 pills per day at multiple times was overly onerous and preferred if they were combined into one pill (3.2 – *High confidence*) (19,29,30). In two quantitative studies, women preferred to have fewer tablets consumed per day (30,38). However, in one study, women who were allocated to the study arm that used more frequent doses took more calcium overall. (32)

Daily routines and food insecurity

Adherence challenges due to routines

Adherence to calcium supplements consumption was challenging for some women because of conflicting activities in their daily routine, such as consuming other medications or supplements, traveling, being away from home, and household chores, which can lead them to forget to take calcium (4.1 – High confidence) (19,29,30). In quantitative evidence, women described busy work schedules and not being at home as also contributing to forgetting to consume their calcium supplements (38,39,41).

For peer review only - http://bmjopen.bmj.com/site/about/guidelines.xhtml

Food insecurity as a barrier to calcium uptake

Women believed that adequate food was necessary to be able to take the supplements, to avoid nausea. They perceived it as normal to eat before consuming any medication. However, women reported that food insecurity was a critical barrier to calcium uptake (4.2 – *Moderate confidence*) (19,29). Quantitative evidence supported qualitative findings that women with food security, high socio-economic status and living in urban areas are more likely to consume calcium supplements as compared to their counterparts(36,37,40).

Strategies to improve the use of calcium supplements

Implementation of reminders to promote adherence

Women and healthcare providers perceived reminders as beneficial in promoting women's adherence to consuming calcium supplements. Several reminder strategies were described as useful by women and healthcare providers, such as home-based posters, calendars with illustrations and daily reminders, and integrating supplement consumption into women's daily routine, such as mealtimes (5.1 – *High confidence*) (19,28–30). Quantitative evidence supported the qualitative findings that distribution of behaviour change materials to women, such as pill-taking calendars, were associated with increased adherence (30).

Importance of family support and 'adherence partner' implementation

Having family support was instrumental to pregnant women adhering to calcium supplement use. This could be leveraged by notifying family members on the importance of calcium, and appointing someone to be an "adherence partner" or "pill buddy" to help remind her to take it. Both women and healthcare providers were positive about adherence partners in providing support in terms of encouraging them to take the supplements, providing food, helping them around the house, providing emotional support, improving family relationships, and thereby increasing partner or husband involvement in pregnancy (5.2 – *High confidence*) (19,28–31). Women could choose who their adherence partner was, and some opted for their husband, a male or female relative, or their child. Some women reported that the support they received from adherence partners decreased over time (19,29–31), suggesting challenges with sustaining appropriate intake throughout pregnancy.

Quantitative evidence supported the qualitative findings that social support is important in encouraging women to consume calcium supplements (27,30,34–36). Women considered involving a husband, partner, or family in education sessions (35) or appointing someone as the "adherence partner" to be an acceptable strategy for promoting adherence (27,34–36). Women often chose their husband, children, older female relative, cousins or other relatives as adherence partners (27), and were satisfied with the reminders and support received from their adherence partner (27,34,36). However, a randomised trial assessing adherence partners in improving calcium supplement intake showed that high social support, instead of adherence partners alone, was associated with higher adherence to calcium supplement (27).

For peer review only - http://bmjopen.bmj.com/site/about/guidelines.xhtml

Counselling facilitates calcium supplements uptake

Both women and healthcare providers acknowledged that counselling women on the benefits of calcium was a motivator to calcium supplements uptake. Women valued the discussion they have with healthcare providers and felt more confident to take calcium supplements when they received counselling and information and pre-eclampsia and the benefits of calcium from their healthcare providers (5.3 – Moderate confidence) (19,29). This was confirmed by healthcare providers who reported that they have seen positive results following counselling women on iron-folic acid supplements and believed that this would be replicated for calcium-containing supplements (29).

Quantitative evidence extended qualitative findings where not only counselling, but also starting antenatal contacts at early gestational age, higher number of antenatal contacts, and receiving free calcium supplements were associated with high consumption of calcium by women (34,36,39,40).

Healthcare provider factors

Healthcare provider knowledge and training

Varied knowledge about pre-eclampsia among healthcare providers

Healthcare providers' knowledge about pre-eclampsia was varied. Some felt that pre-eclampsia is not a priority health concern in their area and reported never having encountered any case (6.1 – *Moderate confidence*) (19,28,43).

Inadequate training to diagnose and treat pre-eclampsia

While some healthcare providers mentioned that training about pre-eclampsia and calcium supplements was adequate, others reported that their training lacked depth and continuity, and thus felt unprepared to diagnose it and offer information these conditions and the use of calcium for prevention. Healthcare providers expressed the need to have more and continuous training on managing pre-eclampsia, as well as time to address concerns or resistance from the community (6.2 – *High confidence*) (19,28,29). Healthcare providers had positive views of trainings and IEC materials and felt that it helps improve their knowledge (43). They also valued supervision visits which help them solve problems and increases their accountability (43). Support was also needed throughout calcium roll-out to ensure challenges can be addressed promptly during implementation (19).

Beliefs about the intervention

Perceived overmedicalization when prescribing calcium supplements

Both healthcare providers and women perceived that prescribing more tablets to "low-risk" women during pregnancy was a form of over-medicalization of pregnancy. However, some

For peer review only - http://bmjopen.bmj.com/site/about/guidelines.xhtml

healthcare providers felt that calcium supplements were a way to prevent further medicalization due to pre-eclampsia-related complications (7.1 – *Low confidence*) (18).

Beliefs about calcium supplements

Healthcare providers generally had positive beliefs about calcium supplements and there was optimism that calcium could be delivered through antenatal care healthcare providers. Some facilitators motivating healthcare providers to prescribe calcium supplements included their beliefs in its prevention value and expected benefits, that women liked the calcium supplements and experienced benefits from it, and a perceived lack of knowledge on how to treat pre-eclampsia which motivated healthcare providers to side towards prevention (7.2 – High confidence) (18,19,29).

Structural factors

High workload, inadequate staffing, stock out, and lack of equipment

In general, healthcare providers felt that their workload increased by including calcium supplements in antenatal care for pregnant women. Healthcare providers reported existing inadequate staffing, yet they needed to provide additional counselling and prescription to women, especially pregnant women with comorbidities (8.1 – Moderate confidence) (19,29). Stock-outs were also reported as critical barriers, often due to logistical issues in the supply chain (e.g., centralization or procurement changes), errors in demand estimation by government staff, and inadequate storage facilities (43). Importantly, some healthcare providers reported that a lack of equipment to diagnose pre-eclampsia was a barrier to calcium implementation (19). Facility staff members and supervisors reported that utilisation of health information system to monitor calcium stocks, checklists to provide feedback on counselling and gaps to address, as well as collaboration with government staff members could be facilitators of use (43).

Quantitative evidence extended qualitative findings by showing that in the context of a comprehensive integrated program including the implementation of job aids, training, guidelines, monitoring, and feedback session for healthcare providers, could overcome barriers in prescribing women with calcium supplements (32,43).

Mapping to behaviour change models

Through COM-B and TDF mapping (Appendix 6), we identified that the critical facilitators and barriers to address to improve calcium use among women include knowledge, beliefs about consequences, beliefs about capabilities, emotion, social influences, and environmental context and resources to improve calcium use by women. To encourage calcium proscription by healthcare providers, facilitators and barriers related to knowledge, skills, beliefs about consequences and environmental context should be addressed.

Figure 2 shows the categorisation of barriers and facilitators across the Capability, Opportunity and Motivation Behaviour. The mapping shows that factors encouraging

For peer review only - http://bmjopen.bmj.com/site/about/guidelines.xhtml

women's use of and adherence to calcium includes receiving adequate information about pre-eclampsia through counselling with healthcare providers and IEC materials, assurance of calcium safety, receiving preferred characteristics of tablet and doses, presence of family and community support on consumption, early and frequent antenatal contacts, free calcium supplements, and reminder tools distribution. Likewise, factors that may encourage healthcare providers to prescribe calcium supplements include continuous training about identification and management of pre-eclampsia and calcium supplements, dissemination of consistent messages, reminders, and ensuring adequate number of human resources, equipment for diagnosing pre-eclampsia, and availability of calcium pills at health facilities.

Discussion

We included 18 reports from nine studies conducted primarily in LMICs and reporting views of women, adherence partners, and healthcare providers. Our review shows the importance of healthcare providers' knowledge and training about calcium supplements and pre-eclampsia, as women reported providers as the most reliable sources of information and reassurance on safety of calcium and would encourage adherence. Promoting early initiation of antenatal care and consistent messages on pre-eclampsia and calcium supplementation may improve women's use of calcium supplements. Free calcium supplements and options on doses and tablet preferences could help overcome barriers to calcium supplement use for women. Reminder systems and support from family and community may also help increase women´s calcium uptake.

As the most trusted informants, healthcare providers knowledge about calcium and pre-eclampsia are important to support women uptake of calcium supplements. Studies reported different levels of knowledge about calcium and pre-eclampsia among providers, with some providers having persistent beliefs that evidence on calcium supplements during pregnancy is still in the experimental phase. Some healthcare providers might be reluctant to talk about pre-eclampsia with the fear to stress low risk women; however, this reluctance should be balanced by the risk of symptoms of pre-eclampsia going unnoticed by women. Reinforcing messages related to improving pregnancy outcomes and similarities to other supplements taken during pregnancy such as folic acid and iron that also help to may facilitate use of calcium supplements. Healthcare providers also need to know about how to assess eligibility for calcium supplements, including how to screen and score women at high-risk, and how to assess low calcium intake.

Human resource shortages are a recurrent health system challenge, particularly in LMICs which results in overburdened staff unable to deliver recommended practices. Appropriate staffing, particularly of midwives and nurses who provide most antenatal care services, remains crucial to achieve quality of care. Where health providers constraints persist, innovative strategies to streamline antenatal care practices may be needed to improve efficiency.(44) Additionally, ensuring availability of diagnostic tools and calcium stocks is critical to ensure appropriate prescription and delivery of care.

For peer review only - http://bmjopen.bmj.com/site/about/guidelines.xhtml

Implications for research, policy, and practice

The TDF and COM-B mapping in our review can be used by researchers and programme implementers to inform the development of implementation models to optimise the use of calcium supplements. Assessing the extent to which the barriers and facilitators to calcium prescription and use identified in our review are potential implementation challenges in different contexts can be a useful starting point for formative research to scale-up implementation. Table 3 presents a list of questions derived from our findings and may help programme managers, policymakers, researchers, and other stakeholders to identify and address factors that may affect prescription and use of calcium supplements during pregnancy.

Table 3. Implications for research, policy, and practice

This table presents a list of questions derived from our findings and may help programme managers, policymakers, researchers, and other stakeholders to identify and address factors that may affect implementation and scale-up of calcium supplements for women during pregnancy. Assessing the extent to which the barriers and facilitators identified in our review are potential implementation challenges in different settings is a useful starting point for formative research to scale this intervention.

Domain	List of questions
**Guidelines and protocols**	1. Are guidelines and clinical protocols on pre-eclampsia/eclampsia and calcium supplements during pregnancy consistent between WHO, national, and facility-levels?
**Knowledge and learning**	2. Do women or healthcare providers have scepticism or concerns about adverse effects of calcium supplements during pregnancy that can be addressed?<br>3. Do women and their family members receive education and educational materials about signs of pre-eclampsia/eclampsia early in pregnancy?<br>4. Do women have sufficient time and opportunity to discuss pre-eclampsia/eclampsia with healthcare providers during antenatal care?<br>5. Do women have sufficient time and opportunity to discuss calcium supplementation with healthcare providers during antenatal care, including addressing fears about side effects, managing side effects, safety concerns, and reinforcing positive messaging about expected benefits?<br>6. Have concerns from both women and healthcare providers about calcium supplements as a form of overmedicalisation of pregnancy been addressed in culturally-appropriate ways?<br>7. Have healthcare providers received in-service training on pre-eclampsia/eclampsia prevention and management, including the importance of calcium supplements during pregnancy for prevention?
**Strategies to improve use**	8. Do all relevant cadres of healthcare providers (including midwives and nurses) have authority to prescribe calcium supplements during pregnancy?<br>9. Do women have the opportunity to try different types of calcium tablets to suit their preferences, such as chewable/non-chewable, different tastes, and different size tablets?<br>10. Do women have the opportunity to try different calcium dosing combinations to suit their schedules and preferences?<br>11. For women experiencing or at risk of food insecurity during pregnancy, are there additional social services to support adequate nutrition intake during pregnancy?<br>12. Have different types of reminder systems (e.g., posters for home, calendars, and integration into daily routines) been designed with women and their families to encourage use?

For peer review only - http://bmjopen.bmj.com/site/about/guidelines.xhtml

	13. Has support from family and/or adherence partners been integrated for women, and do family members or adherence partners have the opportunity to attend educational sessions?<br>14. Are stocks of calcium readily available in the antenatal care wards?<br>15. Is there sufficient funding and budget allocation to ensure continuous procurement and distribution of calcium supplements?

Strengths and limitations

Most included studies were from low- and middle-income countries and almost half came from the same project conducted in Kenya and Ethiopia, which may limit transferability of results to other contexts. The results from our review therefore can be used to guide formative research as well as implementation and programme planning in other contexts. Most included studies engaged women who attended antenatal care; therefore, might not be representative of those that do not reach the health system during pregnancy. None of the included studies reported the perspectives of policymakers.

Despite these limitations, this is the first systematic review of barriers and facilitators to calcium supplement use during pregnancy. We adopted a mixed methods approach which allowed for inclusion and consolidation of studies with a range of designs. Mapping the review findings to behaviour change models improved understanding of how barriers and facilitators influence calcium uptake, and consequently can be addressed in future interventional or programmatic work.

Conclusion

Our review identified a range of barriers and facilitators affecting calcium supplements during pregnancy to prevent pre-eclampsia. When formulating intervention and policies on calcium supplement use, relevant stakeholders should consider the identified barriers and facilitators to optimise uptake. Findings from this study can inform implementation considerations, to ensure effective and equitable provision and scale-up of calcium interventions.

Funding: This work was funded by the Medical Research Council [grant number MR/T038861/1] and the UNDP-UNFPA-UNICEF-WHO-World Bank Special Programme of Research, Development and Research Training in Human Reproduction (HRP), a cosponsored programme executed by the World Health Organization (WHO). MAB's time is supported by an Australian Research Council Discovery Early Career Researcher Award (DE200100264) and a Dame Kate Campbell Fellowship (University of Melbourne Faculty of Medicine, Dentistry and Health Sciences).

Competing interests: None declared

Ethics: Ethical approval was not required; no primary data were collected.

Patient consent for publication: Not required.

For peer review only - http://bmjopen.bmj.com/site/about/guidelines.xhtml

Contributor ship Statement: All authors have made substantial contributions to the following: Conceptualization: ST, APB, MAB, JA, GC Data curation: TR, JA, GC, HM, RIZ Formal analysis: GC, HM, RIZ, MAB, Funding acquisition: ST, APB, JA, Investigation and methodology: GC, HM, RIZ, MAB, Writing – original draft: GC, HM, RIZ, MAB, Writing – review and editing: all authors

Data Sharing Statement: All data relevant to the study are included in the article or uploaded as supplementary information.

For peer review only

References

1. Poon LC, Shennan A, Hyett JA, Kapur A, Hadar E, Divakar H, et al. The International Federation of Gynecology and Obstetrics (FIGO) initiative on pre-eclampsia: A pragmatic guide for first-trimester screening and prevention. Int J Gynecol Obstet. 2019;145(S1).
2. Mol BWJ, Roberts CT, Thangaratinam S, Magee LA, De Groot CJM, Hofmeyr GJ. Pre-eclampsia. In: The Lancet. 2016. p. 999–1011.
3. Gestational Hypertension and Preeclampsia: ACOG Practice Bulletin, Number 222. Obstet Gynecol. 2020;135(6).
4. Say L, Chou D, Gemmill A, Tunçalp Ö, Moller A-B, Daniels J, et al. Global causes of maternal death: a WHO systematic analysis. Lancet Glob Heal [Internet]. 2014 Jun;2(6):e323–33. Available from: https://linkinghub.elsevier.com/retrieve/pii/S2214109X1470227X
5. Steegers EAP, von Dadelszen P, Duvekot JJ, Pijnenborg R. Pre-eclampsia. Lancet [Internet]. 2010 Aug;376(9741):631–44. Available from: https://linkinghub.elsevier.com/retrieve/pii/S0140673610602796
6. Prevention and treatment of pre-eclampsia and eclampsia. World Health. 2011.
7. Hofmeyr GJ, Lawrie TA, Atallah ÁN, Torloni MR. Calcium supplementation during pregnancy for preventing hypertensive disorders and related problems. Vol. 2018, Cochrane Database of Systematic Reviews. 2018.
8. WHO recommendation: Calcium supplementation during pregnancy for the prevention of pre-eclampsia and its complications. Geneva; 2018.
9. WHO recommendation on Calcium supplementation before pregnancy for the prevention of pre-eclampsia and its complications. Geneva; 2020.
10. WHO Recommendations on Antenatal Care for a Positive Pregnancy Experience. Geneva; 2013.
11. Omotayo MO, Dickin KL, O'Brien KO, Neufeld LM, De Regil LM, Stoltzfus RJ. Calcium Supplementation to Prevent Preeclampsia: Translating Guidelines into Practice in Low-Income Countries. Adv Nutr. 2016;7(2):275–8.
12. Page MJ, McKenzie JE, Bossuyt PM, Boutron I, Hoffmann TC, Mulrow CD, et al. The PRISMA 2020 statement: An updated guideline for reporting systematic reviews. Vol. 372, The BMJ. 2021.
13. Tong A, Flemming K, McInnes E, Oliver S, Craig J. Enhancing transparency in reporting the synthesis of qualitative research: ENTREQ. BMC Med Res Methodol. 2012;12.
14. Glenton C, Lewin S, Downe S, Paulsen E, Munabi-Babigumira S, Johansen M, et al. Qualitative Evidence Syntheses Within Cochrane Effective Practice and Organisation of Care: Developing a Template and Guidance. Int J Qual Methods. 2021;20.

For peer review only - http://bmjopen.bmj.com/site/about/guidelines.xhtml

15. Covidence systematic review software. Melbourne, Australia: Veritas Health Innovation;

16. Hong QN, Fàbregues S, Bartlett G, Boardman F, Cargo M, Dagenais P, et al. The Mixed Methods Appraisal Tool (MMAT) version 2018 for information professionals and researchers. Educ Inf. 2018;34(4).

17. Thomas J, Harden A. Methods for the thematic synthesis of qualitative research in systematic reviews. BMC Med Res Methodol [Internet]. 2008 Dec 10;8(1):45. Available from: https://bmcmedresmethodol.biomedcentral.com/articles/10.1186/1471-2288-8-45

18. Vestering A, Bekker MN, Grobbee DE, van der Graaf R, Franx A, Crombag NMT, et al. Views and preferences of medical professionals and pregnant women about a novel primary prevention intervention for hypertensive disorders of pregnancy: a qualitative study. Reprod Health [Internet]. 2019 May 2;16(1):46. Available from: http://www.ncbi.nlm.nih.gov/pubmed/31046778

19. Birhanu Z, Chapleau GM, Ortolano SE, Mamo G, Martin SL, Dickin KL. Ethiopian women's perspectives on antenatal care and iron-folic acid supplementation: Insights for translating global antenatal calcium guidelines into practice. Matern Child Nutr [Internet]. 2018 Feb;14:e12424. Available from: https://onlinelibrary.wiley.com/doi/10.1111/mcn.12424

20. Michie S, van Stralen MM, West R. The behaviour change wheel: A new method for characterising and designing behaviour change interventions. Implement Sci [Internet]. 2011 Dec 23;6(1):42. Available from: http://implementationscience.biomedcentral.com/articles/10.1186/1748-5908-6-42

21. Atkins L, Francis J, Islam R, O'Connor D, Patey A, Ivers N, et al. A guide to using the Theoretical Domains Framework of behaviour change to investigate implementation problems. Implement Sci [Internet]. 2017 Dec 21;12(1):77. Available from: http://implementationscience.biomedcentral.com/articles/10.1186/s13012-017-0605-9

22. Lewin S, Bohren M, Rashidian A, Munthe-Kaas H, Glenton C, Colvin CJ, et al. Applying GRADE-CERQual to qualitative evidence synthesis findings—paper 2: how to make an overall CERQual assessment of confidence and create a Summary of Qualitative Findings table. Implement Sci [Internet]. 2018 Jan 25;13(S1):10. Available from: https://implementationscience.biomedcentral.com/articles/10.1186/s13012-017-0689-2

23. Munthe-Kaas H, Bohren MA, Glenton C, Lewin S, Noyes J, Tunçalp Ö, et al. Applying GRADE-CERQual to qualitative evidence synthesis findings—paper 3: how to assess methodological limitations. Implement Sci [Internet]. 2018 Jan 25;13(S1):9. Available from: https://implementationscience.biomedcentral.com/articles/10.1186/s13012-017-0690-9

24. Colvin CJ, Garside R, Wainwright M, Munthe-Kaas H, Glenton C, Bohren MA, et al. Applying GRADE-CERQual to qualitative evidence synthesis findings—paper 4: how to assess coherence. Implement Sci [Internet]. 2018 Jan 25;13(S1):13. Available from: https://implementationscience.biomedcentral.com/articles/10.1186/s13012-017-0691-8

For peer review only - http://bmjopen.bmj.com/site/about/guidelines.xhtml

25. Glenton C, Carlsen B, Lewin S, Munthe-Kaas H, Colvin CJ, Tunçalp Ö, et al. Applying GRADE-CERQual to qualitative evidence synthesis findings—paper 5: how to assess adequacy of data. Implement Sci [Internet]. 2018 Jan 25;13(S1):14. Available from: https://implementationscience.biomedcentral.com/articles/10.1186/s13012-017-0692-7

26. Noyes J, Booth A, Lewin S, Carlsen B, Glenton C, Colvin CJ, et al. Applying GRADE-CERQual to qualitative evidence synthesis findings–paper 6: how to assess relevance of the data. Implement Sci [Internet]. 2018 Jan 25;13(S1):4. Available from: https://implementationscience.biomedcentral.com/articles/10.1186/s13012-017-0693-6

27. Martin SL, Omotayo MO, Pelto GH, Chapleau GM, Stoltzfus RJ, Dickin KL. Adherence-specific social support enhances adherence to calcium supplementation regimens among pregnant women. J Nutr. 2017;147(4).

28. Martin SL, Seim GL, Wawire S, Chapleau GM, Young SL, Dickin KL. Translating formative research findings into a behaviour change strategy to promote antenatal calcium and iron and folic acid supplementation in western Kenya. Matern Child Nutr [Internet]. 2017 Jan 22;13(1):mcn.12233. Available from: https://onlinelibrary.wiley.com/doi/10.1111/mcn.12233

29. Martin SL, Wawire V, Ombunda H, Li T, Sklar K, Tzehaie H, et al. Integrating Calcium Supplementation into Facility-Based Antenatal Care Services in Western Kenya: A Qualitative Process Evaluation to Identify Implementation Barriers and Facilitators. Curr Dev Nutr [Internet]. 2018 Nov 1;2(11). Available from: https://academic.oup.com/cdn/article/doi/10.1093/cdn/nzy068/5078341

30. Omotayo MO, Martin SL, Stoltzfus RJ, Ortolano SE, Mwanga E, Dickin KL. With adaptation, the WHO guidelines on calcium supplementation for prevention of pre-eclampsia are adopted by pregnant women. Matern Child Nutr [Internet]. 2018 Apr;14(2):e12521. Available from: http://doi.wiley.com/10.1111/mcn.12521

31. Martin SL, Omotayo MO, Chapleau GM, Stoltzfus RJ, Birhanu Z, Ortolano SE, et al. Adherence partners are an acceptable behaviour change strategy to support calcium and iron-folic acid supplementation among pregnant women in Ethiopia and Kenya. Matern Child Nutr [Internet]. 2017 Jul;13(3):e12331. Available from: https://onlinelibrary.wiley.com/doi/10.1111/mcn.12331

32. Omotayo MO, Dickin KL, Pelletier DL, Martin SL, Kung'u JK, Stoltzfus RJ. Feasibility of integrating calcium and iron-folate supplementation to prevent preeclampsia and anemia in pregnancy in primary healthcare facilities in Kenya. Matern Child Nutr [Internet]. 2018 Feb;14:e12437. Available from: https://onlinelibrary.wiley.com/doi/10.1111/mcn.12437

33. Kachwaha S, Nguyen PH, Tran LM, Avula R, Young MF, Ghosh S, et al. Specificity Matters: Unpacking Impact Pathways of Individual Interventions within Bundled Packages Helps Interpret the Limited Impacts of a Maternal Nutrition Intervention in India. J Nutr [Internet]. 2022 Feb 8;152(2):612–29. Available from:

For peer review only - http://bmjopen.bmj.com/site/about/guidelines.xhtml

https://academic.oup.com/jn/article/152/2/612/6430865

34. Nguyen PH, Kachwaha S, Avula R, Young M, Tran LM, Ghosh S, et al. Maternal nutrition practices in Uttar Pradesh, India: Role of key influential demand and supply factors. Matern Child Nutr [Internet]. 2019 Oct 18;15(4). Available from: https://onlinelibrary.wiley.com/doi/10.1111/mcn.12839

35. Nguyen PH, Frongillo EA, Sanghvi T, Wable G, Mahmud Z, Tran LM, et al. Engagement of Husbands in a Maternal Nutrition Program Substantially Contributed to Greater Intake of Micronutrient Supplements and Dietary Diversity during Pregnancy: Results of a Cluster-Randomized Program Evaluation in Bangladesh. J Nutr [Internet]. 2018 Aug 1;148(8):1352–63. Available from: https://academic.oup.com/jn/article/148/8/1352/5040613

36. Nguyen PH, Sanghvi T, Kim SS, Tran LM, Afsana K, Mahmud Z, et al. Factors influencing maternal nutrition practices in a large scale maternal, newborn and child health program in Bangladesh. de Souza RJ, editor. PLoS One [Internet]. 2017 Jul 10;12(7):e0179873. Available from: https://dx.plos.org/10.1371/journal.pone.0179873

37. Bora K, Barman B, Pala S, Das A, Doke G, Tripura A. Coverage of antenatal iron-folic acid and calcium distribution during pregnancy and their contextual determinants in the northeastern region of India. Front Nutr [Internet]. 2022 Jul 18;9. Available from: https://www.frontiersin.org/articles/10.3389/fnut.2022.894245/full

38. Baxter J-AB, Roth DE, Al Mahmud A, Ahmed T, Islam M, Zlotkin SH. Tablets Are Preferred and More Acceptable Than Powdered Prenatal Calcium Supplements among Pregnant Women in Dhaka, Bangladesh. J Nutr [Internet]. 2014 Jul 1;144(7):1106–12. Available from: https://academic.oup.com/jn/article/144/7/1106/4569766

39. Thapa K, Sanghvi H, Rawlins B, Karki YB, Regmi K, Aryal S, et al. Coverage, compliance, acceptability and feasibility of a program to prevent pre-eclampsia and eclampsia through calcium supplementation for pregnant women: An operations research study in one district of Nepal. BMC Pregnancy Childbirth. 2016;

40. Liu D, Cheng Y, Dang S, Wang D, Zhao Y, Li C, et al. Maternal adherence to micronutrient supplementation before and during pregnancy in Northwest China: a large-scale population-based cross-sectional survey. BMJ Open [Internet]. 2019 Aug 8;9(8):e028843. Available from: https://bmjopen.bmj.com/lookup/doi/10.1136/bmjopen-2018-028843

41. Shakya Shrestha S, Adhikari R, Tamrakar S, Shrestha R, Shrestha A. Adherence to Iron, Folic Acid and Calcium Supplement and Factors Affecting it among the Antenatal Care Attending Women in a Tertiary Care Hospital: A Cross Sectional Study. Kathmandu Univ Med J (KUMJ) [Internet]. 18(70):186–92. Available from: http://www.ncbi.nlm.nih.gov/pubmed/33594028

42. Ghosh-Jerath S, Devasenapathy N, Singh A, Shankar A, Zodpey S. Ante natal care (ANC) utilization, dietary practices and nutritional outcomes in pregnant and recently delivered women in urban slums of Delhi, India: an exploratory cross-sectional study. Reprod Health

For peer review only

[Internet]. 2015 Dec 20;12(1):20. Available from: https://reproductive-health-journal.biomedcentral.com/articles/10.1186/s12978-015-0008-9

43. Kachwaha S, Nguyen PH, Tran LM, Avula R, Young MF, Ghosh S, et al. Specificity Matters: Unpacking Impact Pathways of Individual Interventions within Bundled Packages Helps Interpret the Limited Impacts of a Maternal Nutrition Intervention in India. J Nutr. 2022 Feb;152(2):612–29.

44. Betrán AP, Bergel E, Griffin S, Melo A, Nguyen MH, Carbonell A, et al. Provision of medical supply kits to improve quality of antenatal care in Mozambique: a stepped-wedge cluster randomised trial. Lancet Glob Heal [Internet]. 2018 Jan;6(1):e57–65. Available from: https://linkinghub.elsevier.com/retrieve/pii/S2214109X17304217

For peer review only

For peer review only

For peer review only - http://bmjopen.bmj.com/site/about/guidelines.xhtml

For peer review only

For peer review only - http://bmjopen.bmj.com/site/about/guidelines.xhtml

PRISMA 2020 flow diagram for new systematic reviews which included searches of databases, registers, and other sources

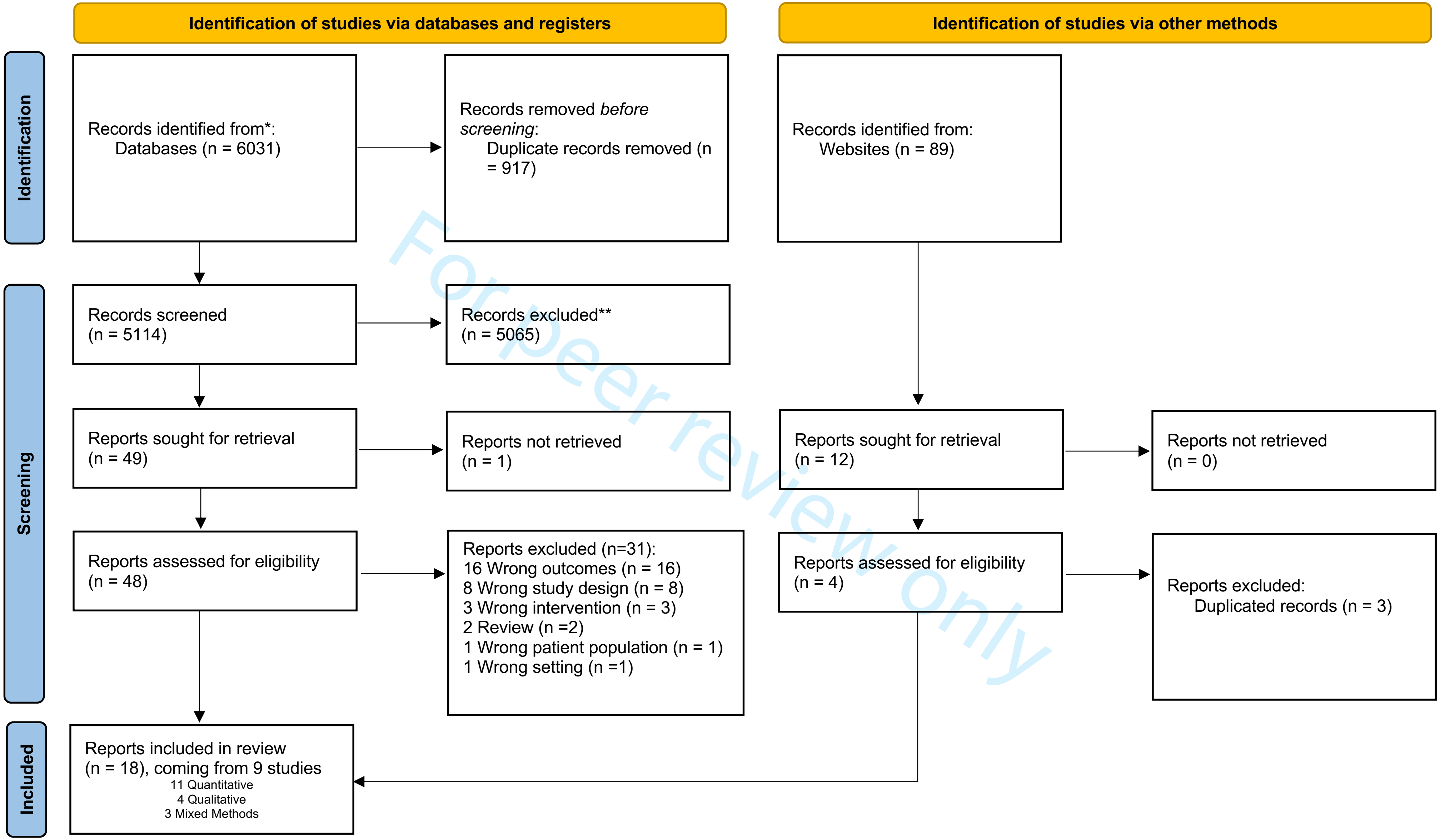

*Consider, if feasible to do so, reporting the number of records identified from each database or register searched (rather than the total number across all databases/registers).
**If automation tools were used, indicate how many records were excluded by a human and how many were excluded by automation tools.

From: Page MJ, McKenzie JE, Bossuyt PM, Boutron I, Hoffmann TC, Mulrow CD, et al. The PRISMA 2020 statement: an updated guideline for reporting systematic reviews. BMJ 2021;372:n71. doi: 10.1136/bmj.n71. For more information, visit: http://www.prisma-statement.org/

For peer review only

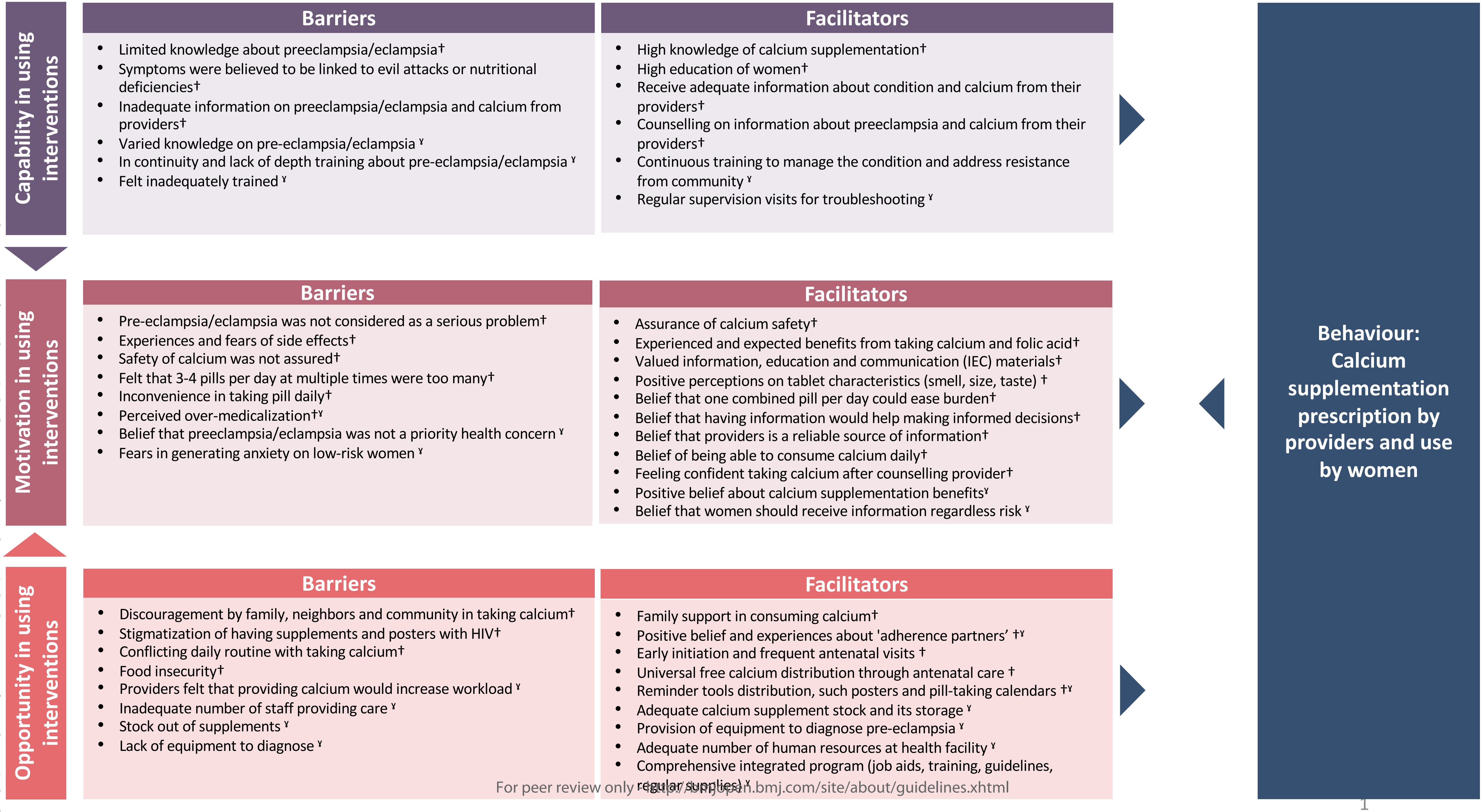

For peer review only - http://bmjopen.bmj.com/site/about/guidelines.xhtml

†: woman's factors; ¥: providers factors

Appendix 1. PRISMA 2020 Main Checklist

Topic	No.	Item	Location where item is reported
**TITLE**			
**Title**	1	Identify the report as a systematic review.	Title, page 1
**ABSTRACT**			
**Abstract**	2	See the PRISMA 2020 for Abstracts checklist	Abstract, page 2
**INTRODUCTION**			
**Rationale**	3	Describe the rationale for the review in the context of existing knowledge.	Background, page 4
**Objectives**	4	Provide an explicit statement of the objective(s) or question(s) the review addresses.	Background, page 4
**METHODS**			
**Eligibility criteria**	5	Specify the inclusion and exclusion criteria for the review and how studies were grouped for the syntheses.	Methods – Type of studies, Topic of interest, page 5
**Information sources**	6	Specify all databases, registers, websites, organisations, reference lists and other sources searched or consulted to identify studies. Specify the date when each source was last searched or consulted.	Methods – Search methods for identification of studies, page 5
**Search strategy**	7	Present the full search strategies for all databases, registers and websites, including any filters and limits used.	Methods – Appendix 1
**Selection process**	8	Specify the methods used to decide whether a study met the inclusion criteria of the review, including how many reviewers screened each record and each report retrieved, whether they worked independently, and if applicable, details of automation tools used in the process.	Methods – Selection of studies

For peer review only - http://bmjopen.bmj.com/site/about/guidelines.xhtml

Topic	No.	Item	Location where item is reported
**Data collection process**	9	Specify the methods used to collect data from reports, including how many reviewers collected data from each report, whether they worked independently, any processes for obtaining or confirming data from study investigators, and if applicable, details of automation tools used in the process.	Methods – Data extraction and assessing methodological limitations, page 6
**Data items**	10a	List and define all outcomes for which data were sought. Specify whether all results that were compatible with each outcome domain in each study were sought (e.g. for all measures, time points, analyses), and if not, the methods used to decide which results to collect.	Methods – Data extraction and assessing methodological limitations, page 6
	10b	List and define all other variables for which data were sought (e.g. participant and intervention characteristics, funding sources). Describe any assumptions made about any missing or unclear information.	Methods – Data extraction and assessing methodological limitations, page 6
**Study risk of bias assessment**	11	Specify the methods used to assess risk of bias in the included studies, including details of the tool(s) used, how many reviewers assessed each study and whether they worked independently, and if applicable, details of automation tools used in the process.	Methods – Data extraction and assessing methodological limitations, page 6
**Effect measures**	12	Specify for each outcome the effect measure(s) (e.g. risk ratio, mean difference) used in the synthesis or presentation of results.	Not applicable
**Synthesis methods**	13a	Describe the processes used to decide which studies were eligible for each synthesis (e.g. tabulating the study intervention characteristics and comparing against the planned groups for each synthesis (item 5)).	Not applicable
	13b	Describe any methods required to prepare the data for presentation or synthesis, such as handling of missing summary statistics, or data conversions.	Not applicable
	13c	Describe any methods used to tabulate or visually display results of individual studies and syntheses.	Methods – Data management, analysis, and synthesis, page 6
	13d	Describe any methods used to synthesize results and provide a rationale for the choice(s). If meta-analysis was performed, describe the model(s), method(s) to identify the presence and extent of statistical heterogeneity, and software package(s) used.	Methods – Data management, analysis, and synthesis, page 6

For peer review only - http://bmjopen.bmj.com/site/about/guidelines.xhtml

Topic	No.	Item	Location where item is reported
	13e	Describe any methods used to explore possible causes of heterogeneity among study results (e.g. subgroup analysis, meta-regression).	Not Applicable
	13f	Describe any sensitivity analyses conducted to assess robustness of the synthesized results.	Methods – Data management, analysis, and synthesis, page 6
**Reporting bias assessment**	14	Describe any methods used to assess risk of bias due to missing results in a synthesis (arising from reporting biases).	Methods – Data management, analysis, and synthesis, page 6
**Certainty assessment**	15	Describe any methods used to assess certainty (or confidence) in the body of evidence for an outcome.	Methods – Data management, analysis, and synthesis, page 6
**RESULTS**			
**Study selection**	16a	Describe the results of the search and selection process, from the number of records identified in the search to the number of studies included in the review, ideally using a flow diagram.	Results, page 7 and Figure 1. PRISMA flowchart
	16b	Cite studies that might appear to meet the inclusion criteria, but which were excluded, and explain why they were excluded.	Figure 2. PRISMA flowchart
**Study characteristics**	17	Cite each included study and present its characteristics.	Results, page 7 Table 1
**Risk of bias in studies**	18	Present assessments of risk of bias for each included study.	Results, page 7
**Results of individual studies**	19	For all outcomes, present, for each study: (a) summary statistics for each group (where appropriate) and (b) an effect estimate and its precision (e.g. confidence/credible interval), ideally using structured tables or plots.	Not applicable
**Results of syntheses**	20a	For each synthesis, briefly summarise the characteristics and risk of bias among contributing studies.	Results page 10 – Table 2. Summary of qualitative findings and GRADE-CERQual Evidence Profile

Topic	No.	Item	Location where item is reported
	20b	Present results of all statistical syntheses conducted. If meta-analysis was done, present for each the summary estimate and its precision (e.g. confidence/credible interval) and measures of statistical heterogeneity. If comparing groups, describe the direction of the effect.	Not applicable
	20c	Present results of all investigations of possible causes of heterogeneity among study results.	Not applicable
	20d	Present results of all sensitivity analyses conducted to assess the robustness of the synthesized results.	Not applicable
**Reporting biases**	21	Present assessments of risk of bias due to missing results (arising from reporting biases) for each synthesis assessed.	Not applicable
**Certainty of evidence**	22	Present assessments of certainty (or confidence) in the body of evidence for each outcome assessed.	Results Page 10-18– Table 2. Summary of qualitative findings and GRADE-CERQual Evidence Profile
**DISCUSSION**			
**Discussion**	23a	Provide a general interpretation of the results in the context of other evidence.	Discussions Page 18 – Interpretation
	23b	Discuss any limitations of the evidence included in the review.	Discussions Page 19–20 Strengths and limitations
	23c	Discuss any limitations of the review processes used.	Discussions Page 19-20– Strengths and limitations
	23d	Discuss implications of the results for practice, policy, and future research.	Discussions Page 19– Implications for practice and conclusions
**OTHER INFORMATION**			
**Registration and protocol**	24a	Provide registration information for the review, including register name and registration number, or state that the review was not registered.	Methods, page 5

Topic	No.	Item	Location where item is reported
	24b	Indicate where the review protocol can be accessed, or state that a protocol was not prepared.	Methods, Page 5
	24c	Describe and explain any amendments to information provided at registration or in the protocol.	Methods, Page 5
**Support**	25	Describe sources of financial or non-financial support for the review, and the role of the funders or sponsors in the review.	Sources of funding, Page 21
**Competing interests**	26	Declare any competing interests of review authors.	Competing interests, Page 21
**Availability of data, code and other materials**	27	Report which of the following are publicly available and where they can be found: template data collection forms; data extracted from included studies; data used for all analyses; analytic code; any other materials used in the review.	Development of themes can be found on the Appendix

For peer review only - http://bmjopen.bmj.com/site/about/guidelines.xhtml

Appendix 1.1. PRISMA Abstract Checklist

Topic	No.	Item	Reported?
**TITLE**			
**Title**	1	Identify the report as a systematic review.	Yes
**BACKGROUND**			
**Objectives**	2	Provide an explicit statement of the main objective(s) or question(s) the review addresses.	Yes
**METHODS**			
**Eligibility criteria**	3	Specify the inclusion and exclusion criteria for the review.	Yes
**Information sources**	4	Specify the information sources (e.g. databases, registers) used to identify studies and the date when each was last searched.	Yes
**Risk of bias**	5	Specify the methods used to assess risk of bias in the included studies.	Yes
**Synthesis of results**	6	Specify the methods used to present and synthesize results.	Yes
**RESULTS**			
**Included studies**	7	Give the total number of included studies and participants and summarise relevant characteristics of studies.	Yes
**Synthesis of results**	8	Present results for main outcomes, preferably indicating the number of included studies and participants for each. If meta-analysis was done, report the summary estimate and confidence/credible interval. If comparing groups, indicate the direction of the effect (i.e. which group is favoured).	Yes
**DISCUSSION**			
**Limitations of evidence**	9	Provide a brief summary of the limitations of the evidence included in the review (e.g. study risk of bias, inconsistency and imprecision).	Yes

For peer review only - http://bmjopen.bmj.com/site/about/guidelines.xhtml

Topic	No.	Item	Reported?
**Interpretation**	10	Provide a general interpretation of the results and important implications.	Yes
**OTHER**			
**Funding**	11	Specify the primary source of funding for the review.	Yes
**Registration**	12	Provide the register name and registration number.	Yes

From: Page MJ, McKenzie JE, Bossuyt PM, Boutron I, Hoffmann TC, Mulrow CD, et al. The PRISMA 2020 statement: an updated guideline for reporting systematic reviews. MetaArXiv. 2020, September 14. DOI: 10.31222/osf.io/v7gm2. For more information, visit: www.prisma-statement.org

For peer review only

For peer review only - http://bmjopen.bmj.com/site/about/guidelines.xhtml

Appendix 2. Enhancing transparency in reporting the synthesis of qualitative research: ENTREQ Checklist (Tong, et al., 2012)

Item No.	Guide and Description	Report Location
1. Aim	State the research question the synthesis addresses	Background
2. Synthesis methodology	Identify the synthesis methodology or theoretical framework which underpins the synthesis, and describe the rationale for choice of methodology (e.g. meta-ethnography, thematic synthesis, critical interpretive synthesis, grounded theory synthesis, realist synthesis, meta-aggregation, meta-study, framework synthesis)	Methods – _Data management, analysis, and synthesis
3. Approach to searching	Indicate whether the search was pre-planned (comprehensive search strategies to seek all available studies) or iterative (to seek all available concepts until they theoretical saturation is achieved)	Methods – Search methods for identification of studies
4. Inclusion criteria	Specify the inclusion/exclusion criteria (e.g. in terms of population, language, year limits, type of publication, study type)	Methods – Selection of studies
5. Data sources	Describe the information sources used (e.g. electronic databases (MEDLINE, EMBASE, CINAHL, psycINFO), grey literature databases (digital thesis, policy reports), relevant organisational websites, experts, information specialists, generic web searches (Google Scholar) hand searching, reference lists) and when the searches conducted; provide the rationale for using the data sources	Methods - Search methods for identification of studies
6. Electronic Search strategy	Describe the literature search (e.g. provide electronic search strategies with population terms, clinical or health topic terms, experiential or social phenomena related terms, filters for qualitative research, and search limits)	Appendix 5
7. Study screening methods	Describe the process of study screening and sifting (e.g. title, abstract and full text review, number of independent reviewers who screened studies)	Methods - Search methods for identification of studies
8. Study characteristics	Present the characteristics of the included studies (e.g. year of publication, country, population, number of participants, data collection, methodology, analysis, research questions)	Table 1 - Characteristics of included studies
9. Study selection results	Identify the number of studies screened and provide reasons for study exclusion (e.g. for comprehensive searching, provide numbers of studies screened and reasons for exclusion indicated in a figure/flowchart; for iterative searching describe reasons for study exclusion	Fig 1 - PRISMA flow diagram

For peer review only

	and inclusion based on modifications to the research question and/or contribution to theory development)	
10. Rationale for appraisal	Describe the rationale and approach used to appraise the included studies or selected findings (e.g. assessment of conduct (validity and robustness), assessment of reporting (transparency), assessment of content and utility of the findings)	Appendix 3 Table of critical appraisal results
11. Appraisal items	State the tools, frameworks and criteria used to appraise the studies or selected findings (e.g. Existing tools: CASP, QARI, COREQ, Mays and Pope [25]; reviewer developed tools; describe the domains assessed: research team, study design, data analysis and interpretations, reporting)	Methods - Data extraction and assessing the methodological limitations
12. Appraisal process	Indicate whether the appraisal was conducted independently by more than one reviewer and if consensus was required	Methods - Data extraction and assessing the methodological limitations
13. Appraisal results	Present results of the quality assessment and indicate which articles, if any, were weighted/excluded based on the assessment and give the rationale	
14. Data extraction	Indicate which sections of the primary studies were analysed and how were the data extracted from the primary studies? (e.g. all text under the headings "results /conclusions" were extracted electronically and entered into a computer software)	Methods- Data management, analysis, and synthesis
15. Software	State the computer software used, if any	None
16. Number of reviewers	Identify who was involved in coding and analysis	Methods - Assessing our confidence in the review findings
17. Coding	Describe the process for coding of data (e.g. line by line coding to search for concepts)	Methods - Assessing our confidence in the review findings
18. Study comparison	Describe how were comparisons made within and across studies (e.g. subsequent studies were coded into pre-existing concepts, and new concepts were created when deemed necessary)	Methods - Assessing our confidence in the review findings
19. Derivation of themes	Explain whether the process of deriving the themes or constructs was inductive or deductive	Methods - Assessing our confidence in the review findings
20. Quotations	Provide quotations from the primary studies to illustrate themes/constructs, and identify whether the quotations were participant quotations of the author's interpretation	Results
21. Synthesis output	Present rich, compelling and useful results that go beyond a summary of the primary studies (e.g. new	Discussion

For peer review only

	interpretation, models of evidence, conceptual models, analytical framework, development of a new theory or construct)	

For peer review only

Appendix 3. Search Strategy

Embase (inception to 2021 March 22)

1 exp Calcium/ or Calcium Carbonate/ (305629)
2 (calcium adj3 supplement*).mp. (9456)
3 1 or 2 (307747)
4 Pregnant Women/ or Prenatal Care/ (109074)
5 (pregnan* or prenatal).mp. (1086333)
6 4 or 5 (1086333)
7 3 and 6 (8440)
8 limit 7 to humans (5885)
9 limit 8 to (amphibia or ape or bird or cat or cattle or chicken or dog or "ducks and geese" or fish or "frogs and toads" or goat or guinea pig or "hamsters and gerbils" or horse or monkey or mouse or "pigeons and doves" or "rabbits and hares" or rat or reptile or sheep or swine) (277)
10 8 not 9 (5608)
11 limit 10 to (conference abstract or conference paper or "conference review" or editorial or erratum or letter or note or "review") (2567)
12 10 not 11 (**3041**)

Embase (inception to 2022 August 16) – Search update

1 exp Calcium/ or Calcium Carbonate/ (323807)
2 (calcium adj3 supplement*).mp. (10017)
3 1 or 2 (326035)
4 Pregnant Women/ or Prenatal Care/ (125455)
5 (pregnan* or prenatal).mp. (1161704)
6 4 or 5 (1161704)
7 3 and 6 (9061)
8 limit 7 to humans (6413)
9 limit 8 to (amphibia or ape or bird or cat or cattle or chicken or dog or "ducks and geese" or fish or "frogs and toads" or goat or guinea pig or "hamsters and gerbils" or horse or monkey or mouse or "pigeons and doves" or "rabbits and hares" or rat or reptile or sheep or swine) (303)
10 8 not 9 (6110)
11 limit 10 to (conference abstract or conference paper or "conference review" or editorial or erratum or letter or note or "review") (2744)
12 10 not 11 (3366)
13 limit 12 to yr="2022 - 2023" (**142**)

MEDLINE (1946 to March Week 2 2021)

1 exp Calcium/ or Calcium Carbonate/ (277850)
2 (calcium adj3 supplement*).mp. (5560)

For peer review only

3 1 or 2 (280742)
4 Pregnant Women/ or Prenatal Care/ (36709)
5 (pregnan* or prenatal).mp. (999134)
6 4 or 5 (999134)
7 3 and 6 (6417)
8 limit 7 to humans (3417)
9 limit 8 to animals (618)
10 8 not 9 (2799)
11 limit 10 to ("review articles" and case reports) (17)
12 10 not 11 (**2782 results**)

MEDLINE (inception to August Week 1, 2022) – Search update

1 exp Calcium/ or Calcium Carbonate/ (286238)
2 (calcium adj3 supplement*).mp. (5967)
3 1 or 2 (289314)
4 Pregnant Women/ or Prenatal Care/ (42373)
5 (pregnan* or prenatal).mp. (1068999)
6 4 or 5 (1068999)
7 3 and 6 6605
8 limit 7 to humans (3545)
9 limit 8 to animals (634)
10 8 not 9 (2911)
11 limit 10 to ("review articles" and case reports) (18)
12 10 not 11 (2893)
13 limit 12 to yr="2021 - 2022" (**89**)

CINAHL

CINAHL (inception to March 2021)

(calcium and supplement*) AND (pregnan* or prenatal) AND (preeclamp* or pre-eclamp* or eclamp* or "gestational hypertension" or "maternal hypertension") (**132**)

CINAHL (March 2021 to August 2022) – Search update

(calcium and supplement*) AND (pregnan* or prenatal) AND (preeclamp* or pre-eclamp* or eclamp* or "gestational hypertension" or "maternal hypertension") (**1**)

GLOBAL HEALTH

GLOBAL HEALTH (inception to March 2021)

(calcium and supplement*) AND (pregnan* or prenatal) AND (preeclamp* or pre-eclamp* or eclamp* or "gestational hypertension" or "maternal hypertension") (**158 results**)

GLOBAL HEALTH (2021 to August 2022) – Search update

(calcium and supplement*) AND (pregnan* or prenatal) AND (preeclamp* or pre-eclamp* or eclamp* or "gestational hypertension" or "maternal hypertension") (**158 results**)

For peer review only - http://bmjopen.bmj.com/site/about/guidelines.xhtml

For peer review only

For peer review only - http://bmjopen.bmj.com/site/about/guidelines.xhtml

Appendix 4. Critical Appraisal Table

Qualitative studies

STUDY DETAIL		SCREENING QUESTIONS		1. QUALITATIVE STUDIES							MMAT RATING
First author	Year	S1. Are there clear research questions?	S2. Do the collected data allow to address the research questions?	1.1. Is the qualitative approach appropriate to answer the research question? (Aim, appropriateness of a qualitative approach)	1.2. Are the qualitative data collection methods adequate to address the research question? (recruitment, data collection)	1.3. Are the findings adequately derived from the data? (rigor in analysis)	1.4. Is the interpretation of results sufficiently substantiated by data? (link from data to findings)	1.5. Is there coherence between qualitative data sources, collection, analysis and interpretation? (overall design from start to finish)	1.6. Have ethical issues been taken into consideration? (consent, confidentiality, ethics approval)	1.7. Is relationship between researcher and participants adequately considered? (interaction and reflection on how research team influences design & implementation)	
**Vestering**	**2019**	Yes	Yes	Yes	Yes	Yes	Yes	Yes	Partial	Partial	"Moderate" (minor issues impacting credibility/validity)
**Birhanu**	**2016**	Yes	Yes	Yes	Yes	Yes	Yes	Yes	Yes	Unclear	"Moderate" (minor issues impacting credibility/validity)
**Martin**	**2016**	Yes	Yes	Yes	Yes	Yes	Yes	Yes	Yes	Unclear	"Moderate" (minor issues impacting credibility/validity)
**Martin**	**2018**	Yes	Yes	Yes	Yes	Yes	Yes	Yes	Yes	Partial	"Moderate" (minor issues impacting credibility/validity)

Quantitative studies

STUDY DETAIL		SCREENING QUESTIONS		3. NON-RANDOMIZED STUDIES					4. QUANTITATIVE DESCRIPTIVE STUDIES					MMAT RATING
First author	Year	S1. Are there clear research questions?	S2. Do the collected data allow to address the research questions?	3.1. Are the participants representative of the target population?	3.2. Are measurements appropriate regarding both the outcome and intervention (or exposure)?	3.3. Are there complete outcome data?	3.4. Are the confounders accounted for in the design and analysis?	3.5. During the study period, is the intervention administered (or exposure occurred) as intended?	4.1. Is the sampling strategy relevant to address the research question?	4.2. Is the sample representative of the target population?	4.3. Are the measurements appropriate?	4.4. Is the risk of nonresponse bias low?	4.5. Is the statistical analysis appropriate to answer the research question?	
Martin	2017	Yes	Yes	No	Yes	Unclear	No	Unclear						“Low” (some issues likely to impact credibility/validity)
Baxter	2014	Yes	Yes						Yes	Unclear	Yes	No	Yes	“Low” (some issues likely to impact credibility/validity)
Thapa	2016	Yes	Yes	Yes	Yes	Yes	Yes	Yes						"High” (no or very minor significant issues)
Nguyen	2019	Yes	Yes						Yes	Yes	Yes	Unclear	Yes	“Moderate” (minor issues impacting credibility/validity)
Omotayo	2018	Yes	Yes	Yes	Yes	Partial	Yes	Yes						“Moderate” (minor issues impacting credibility/validity)
Nguyen	2017	Yes	Yes						Yes	Yes	Yes	Yes	Yes	"High” (no or very minor significant issues)
Nguyen	2018	Yes	Yes						Yes	Yes	Yes	Yes	Yes	"High” (no or very minor significant issues)

Liu	2019	Yes	Yes						Yes	Yes	Partial	No	Yes	"Low" (some issues likely to impact credibility/validity)
Shakya Shrestha	2020	Yes	Yes						Partial	Unclear	Partial	Yes	Partial	"Very low" (significant issues impacting credibility/validity)
Ghosh-Jerath	2015	Yes	Yes						Yes	Yes	Partial	No	Yes	"Low" (some issues likely to impact credibility/validity)
Bora	2022	Yes	Yes						Yes	Yes	Partial	Unclear	Yes	"Low" (some issues likely to impact credibility/validity)

Mixed methods studies

STUDY DETAIL	First author	Omotayo	Martin	Kachwaha
	Year	2017	2017	2022
SCREENING QUESTIONS	S1. Are there clear research questions?	Yes	Yes	Yes
	S2. Do the collected data allow to address the research questions?	Yes	Yes	Yes
1. QUALITATIVE STUDIES	1.1. Is the qualitative approach appropriate to answer the research question? (Aim, appropriateness of a qualitative approach)	Yes	Yes	Yes
	1.2. Are the qualitative data collection methods adequate to address the research question? (recruitment, data collection)	Yes	Yes	yes
	1.3. Are the findings adequately derived from the data? (rigor in analysis)	Yes	Yes	Yes
	1.4. Is the interpretation of results sufficiently substantiated by data? (link from data to findings)	Yes	Yes	Yes
	1.5. Is there coherence between qualitative data sources, collection, analysis and interpretation? (overall design from start to finish)	Yes	Yes	Yes
	1.6. Have ethical issues been taken into consideration? (consent, confidentiality, ethics approval)	Yes	Yes	Yes

	**1.7. Is relationship between researcher and participants adequately considered? (interaction and reflection on how research team influences design & implementation)**	Unclear	Unclear	Unclear
**4. QUANTITATIVE DESCRIPTIVE STUDIES**	**4.1. Is the sampling strategy relevant to address the research question?**	Yes	Yes	Yes
	**4.2. Is the sample representative of the target population?**	Yes	Yes	Yes
	**4.3. Are the measurements appropriate?**	Yes	Yes	Yes
	**4.4. Is the risk of nonresponse bias low?**	Yes	Partial	Partial
	**4.5. Is the statistical analysis appropriate to answer the research question?**	Yes	Yes	Yes
**5. MIXED METHODS STUDIES**	**5.1. Is there an adequate rationale for using a mixed methods design to address the research question?**	Yes	Yes	Yes
	**5.2. Are the different components of the study effectively integrated to answer the research question?**	Yes	Yes	Yes
	**5.3. Are the outputs of the integration of qualitative and quantitative components adequately interpreted?**	Yes	Yes	Yes
	**5.4. Are divergences and inconsistencies between quantitative and qualitative results adequately addressed?**	Yes	Yes	Yes
	**5.5. Do the different components of the study adhere to the quality criteria of each tradition of the methods involved?**	Yes	Partial	Partial
**MMAT RATING**		"High" (no or very minor significant issues)	"Moderate" (minor issues impacting credibility/validity)	"Moderate" (minor issues impacting credibility/validity)

For peer review only

Appendix 6. COM-B Mapping Table

Behavior aimed: calcium supplementation use by women and health providers						
No	COM-B Domain	TDF Domain*	List of factors affecting calcium use	Stakeholders affected (women, health providers, partner)	Evidence Source (Quantitative/Qualitative)	Barriers or facilitators to calcium use
**Women's factors**						
1	Capability	Know	Limited knowledge about preeclampsia/eclampsia	Women	Qualitative	
2	Capability	Know	Counselling on information about preeclampsia and calcium from their providers independent to woman risk	Women	Qualitative	
3	Capability	Know	High knowledge of calcium supplementation	Women	Quantitative	
4	Capability	Know	High education of women	Women	Quantitative	
5	Capability	Know	Symptoms were believed to be linked to evil attacks or nutritional deficiencies	Women	Qualitative	
6	Capability	Know	Inadequate information on preeclampsia/eclampsia and calcium from providers	Women	Qualitative	
7	Capability	Know	Receive adequate information about condition and calcium from their providers	Women	Qualitative	
8	Motivation	Bel Cons	Preeclampsia/eclampsia was not considered as a serious problem by women	Women	Qualitative	
9	Motivation	Bel Cons	Experiences and fears of side effects	Women	Qualitative and quantitative	
10	Motivation	Bel Cons	Assurance of calcium safety	Women	Qualitative	
11	Motivation	Bel Cons	Safety of calcium was not assured	Women	Qualitative	
12	Motivation	Bel Cons	Experienced and expected benefits from taking calcium and folic acid	Women	Qualitative and quantitative	
13	Motivation	Bel Cap	Belief that having essential information would help them making informed decisions	Women	Qualitative	
14	Motivation	Bel Cap	Belief that providers is a reliable source of information	Women	Qualitative	
15	Motivation	Bel Cap	Belief of being able to consume calcium daily	Women	Quantitative	
16	Motivation	Em	Valued information, education and communication (IEC) materials	Women	Qualitative	
17	Motivation	Em	Positive perceptions on calcium tablet characteristics (e.g. size, taste, smell)	Women	Qualitative and quantitative	
18	Motivation	Em	Women felt that 3-4 pills per day at multiple times were too many	Women	Quantitative and qualitative	
19	Motivation	Em	Inconvenience in taking pill daily	Women	Quantitative and qualitative	
20	Motivation	Em	Belief that one combined pill per day could ease burden	Women	Qualitative	
21	Motivation	Em	Feeling confident taking calcium after receiving adequate information from provider	Women	Quantitative	
22	Opportunity	Soc	Discouragement by family, neighbours and community in taking calcium	Women	Qualitative and quantitative	

For peer review only

23	Opportunity	Soc	Stigmatisation of having supplements and posters with HIV	Women	Qualitative	
24	Opportunity	Soc	Family support in consuming calcium	Women	Qualitative and quantitative	
25	Opportunity	Soc	Positive belief and experiences about 'adherence partners'	Women	Qualitative and quantitative	
26	Opportunity	Ev	Conflicting daily routine with taking calcium	Women	Qualitative and quantitative	
27	Opportunity	Ev	Food insecurity	Women	Qualitative and quantitative	
28	Opportunity	Ev	Early initiation and frequent antenatal visits	Women	Quantitative	
29	Opportunity	Ev	Receiving free calcium supplements	Women	Quantitative	
30	Opportunity	Ev	Reminder tools distribution, such as home based posters and pill-taking calendars	Women	Qualitative and Quantitative	
31	Opportunity	Ev	Universal free calcium distribution through antenatal care	Women	Quantitative	
**Providers factors**						
1	Capability	Know	Varied knowledge on pre-eclampsia/eclampsia	Providers	Qualitative	
3	Capability	Skills	Incontinuity and non-in-depth training about pre-eclampsia/eclampsia	Providers	Qualitative	
2	Capability	Skills	Felt inadequately trained	Providers	Qualitative	
4	Capability	Skills	Continuous training to manage the condition and address resistance from community	Providers	Qualitative	
5	Motivation	Bel Cons	Positive belief about calcium supplementation benefits	Providers	Qualitative	
6	Motivation	Bel Cons	Belief that preeclampsia/eclampsia was not a priority health concern	Providers	Qualitative	
7	Motivation	Bel Cons	Perceived over-medicalization to prescribe calcium to low risk women	Women and Providers	Qualitative	
8	Motivation	Bel Cons	Fears in generating anxiety on low-risk women	Providers	Qualitative	
9	Motivation	Bel Cons	Belief that women should get a chance to receive information regardless risk	Providers	Qualitative	
10	Opportunity	Ev	Providers felt that providing calcium would increase workload	Providers	Qualitative	
11	Opportunity	Ev	Inadequate number of staff providing care	Providers	Qualitative	
12	Opportunity	Ev	Comprehensive integrated program (job aids, training, guidelines, regular supplies)	Providers	Quantitative	

* Know: Knowledge, Phys: Physical skills, Mem: Memory, attention and decision processes, Beh Reg: Behavioural regulation, Em: Emotion, Id: Social/professional role and identity, Bel Cons: Belief about consequences, Bel Cap: Belief about capabilities, Int: Intentions, Opt: Optimisms, Ev: Environmental context and resources, Soc: Social influences

For peer review only

Appendix 5. Evidence Profile

No	Summary of qualitative review findings	Contributing qualitative studies	Methodological limitations assessment	Coherence assessment	Relevance assessment	Adequacy assessment	Overall CERQual assessment	Explanation of overall assessment
**Women's factors**								
**1. Women's knowledge and learning**								
**1.1**	**Women's knowledge about pre-eclampsia or eclampsia** Most women had limited knowledge about pre-eclampsia or eclampsia, and these conditions were not typically considered by women to be a serious problem. Symptoms of pre-eclampsia such as swollen feet, severe headache, blurred vision and vomiting were considered normal signs and symptoms in pregnancy, while seizures were associated with evil attacks or nutritional deficiencies.	(#2389 Martin 2016, #4971 Birhanu 2016, #293 Vestering 2019)	**Minor concerns:** Three studies with minor issues (reflexivity and ethics).	**No or very minor concerns**	**Moderate concerns:** One study is indirectly relevant to review aim and represented 3 countries (Kenya, Ethiopia, Netherlands), including 1 low-income, 1 lower middle income and 1 high income country. All perspectives came from health providers and women.	**Minor concerns:** 3 out of 6 studies contributed to review finding (2 thick data, 1 thin data).	**High confidence**	Due to no or very minor concerns on coherence, minor concerns on methodological limitations (reflexivity and ethics), moderate concerns on relevance (1 out of 3 studies are indirectly relevant to our review aim and small number of countries), and minor concerns of adequacy (3 out of 6 contributed with 2 thick and 1 thin data).
**1.2**	**Information provision to women**	(#293 Vestering	**Minor concerns:** Two studies	**Moderate concerns:**	**Moderate concerns:** All studies are	**Serious concerns:**	**Low confidence**	Minor concerns on methodological limitations (ethics and reflexivity), moderate

For peer review only - http://bmjopen.bmj.com/site/about/guidelines.xhtml

No	Summary of qualitative review findings	Contributing qualitative studies	Methodological limitations assessment	Coherence assessment	Relevance assessment	Adequacy assessment	Overall CERQual assessment	Explanation of overall assessment
	Women felt they did not receive adequate information during pregnancy from healthcare providers about pre-eclampsia or eclampsia and calcium supplements. They described wanting to be given more information, regardless of their pre-eclampsia risk status. Women believed that having this essential information could help them to make informed choices and actively participate in their care. There were, however, mixed opinions from healthcare providers - some feared that more information could generate anxiety for women, while others were more supportive of providing information.	2019, #2388 Martin 2018)	with minor issues (ethics and reflexivity).	No good understanding why some providers worry in generating anxiety to women while others are not.	directly relevant to review aim and represented 2 countries (Kenya, Netherlands), including 1 lower middle income and 1 high income country. All perspectives came from health providers and women.	2 out of 6 studies contributed to review finding (1 thick data, 1 thin data).		concerns on coherence (no clear understanding on why some providers worry in generating anxiety to women while the others are not), moderate concerns on relevance (small number of countries), and serious concerns on adequacy (2 out of 6 contributed with 1 thick and 1 thin data).
1.3	**Learning about calcium supplements** Women typically learned about dietary calcium,	(#293 Vestering 2019, #2388 Martin 2018,	**Minor concerns:** Three studies with	**No or very minor concerns**	**Minor concerns:** All studies are directly relevant	**Minor concerns:** 3 out of 6 studies	**High confidence**	Due to no or very minor concerns on coherence, minor concerns on relevance (small number of countries), minor concerns on

For peer review only

No	Summary of qualitative review findings	Contributing qualitative studies	Methodological limitations assessment	Coherence assessment	Relevance assessment	Adequacy assessment	Overall CERQual assessment	Explanation of overall assessment
	including pre-eclampsia and eclampsia symptoms, from healthcare providers. They considered healthcare providers to be the most trusted and reliable source of information, and reported feeling confident about taking calcium-containing supplements after receiving adequate information from them. Women also appreciated receiving information on calcium supplements and pre-eclampsia or eclampsia via information, education, and communication (IEC) materials like videos, media, and trusted websites.	#4971 Birhanu 2016)	minor issues (reflexivity and ethics).		to review aim and represented 3 countries (Kenya, Ethiopia, Netherlands), including 1 low-income, 1 lower middle income and 1 high income country. All perspectives came from health providers and women.	contributed to findings (2 with moderate-thick data and 1 with thin data.		methodological limitations (reflexivity and ethics), and minor concerns on adequacy (3 out of 6 contributed, 1 moderate thick and 2 thin data).
**2. Women's beliefs about calcium supplements in pregnancy**								
**2.1**	**Fears about side-effects as a barrier to calcium supplements uptake** Women's fears about the side effects of calcium supplements affected their adherence. Women	(#1837 Omotayo 2017, #4971 Birhanu 2016, #293 Vestering 2019, #2388	**Minor concerns:** Three out of four studies have minor issues (ethics and	**No or very minor concerns**	**Minor concerns:** All studies are directly relevant to review aim and represented 3	**Minor concerns** Overall, small number of studies contributing to the qualitative	**High Confidence**	Due to no or very minor concerns on coherence, minor concerns on relevance (small number of countries), minor concerns on adequacy (small number of studies contributing to the qualitative evidence synthesis),

For peer review only

No	Summary of qualitative review findings	Contributing qualitative studies	Methodological limitations assessment	Coherence assessment	Relevance assessment	Adequacy assessment	Overall CERQual assessment	Explanation of overall assessment
	highlighted that assurance of safe use of calcium supplement is a key facilitator to consistent use. However, some women felt safety was not assured by healthcare providers, especially when calcium supplements were perceived as "experimental". Women had also received messages from their families or communities that any pills consumed during pregnancy could be harmful.	Martin 2018)	reflexivity) and one study with no or very minor issues.		countries (Kenya, Ethiopia, Netherlands), including 1 low-income, 1 lower middle income and 1 high income country. All perspectives came from women.	evidence synthesis.		and minor concerns on methodological limitations (reflexivity and ethics).
**2.2**	**Women's experiences of side-effects** Some women reported experiencing side effects after taking calcium and iron-folic acid supplements, such as dizziness, vomiting, nausea, stomach aches, loss of appetite, tiredness, diarrhoea, bloating, and burping, yet noted that side effects subsided with time. Women also reported that they continued consuming calcium	(#1837 Omotayo 2017, #2388 Martin 2018, #4971 Birhanu 2016)	**Minor concerns:** Two out of three studies have minor issues (ethics and reflexivity) and one study with no or very minor issues	**No or very minor concerns**	**Moderate concerns:** All studies are directly relevant to review aim and represented 2 countries (Kenya, Ethiopia), including 1 low-income and 1 lower middle-income country.	**Minor concerns:** 3 out of 6 studies contributed to the findings (2 thin and 1 with thick data).	**High Confidence**	Due to no or very minor concerns on coherence, minor concerns on methodological limitations (ethics and reflexivity), moderate concerns on relevance (only low and lower middle-income country and small number of countries) and minor concerns on adequacy (3 out of 6 studies contributed with 2 thick and 1 with thin data).

For peer review only

No	Summary of qualitative review findings	Contributing qualitative studies	Methodological limitations assessment	Coherence assessment	Relevance assessment	Adequacy assessment	Overall CERQual assessment	Explanation of overall assessment
	despite these side effects.				All perspectives came from health providers and women.			
**2.3**	**Concerns about being stigmatized as HIV patients** Women expressed concerns that if they ingested calcium supplements, they could be stigmatized as HIV patients, which was a reported barrier to use. Some women were afraid of being stigmatized as their community often associated nutritional supplement consumption and accompanying reminder posters with HIV.	(#2388 Martin 2018, #293 vestering 2019)	**No or very minor concerns**	**No or very minor concerns**	**Moderate concerns:** All studies are directly relevant to review aim and represented 2 countries (Kenya, Netherlands), including 1 lower middle income and 1 high income country. All perspectives came from health providers and women.	**Serious concerns:** 2 out of 6 studies contributed to review finding (1 thick data and 1 thin data).	**Moderate Confidence**	Due to no or very minor concerns on methodological limitations, no or very minor concerns on coherence, minor concerns on relevance (small number of countries), and serious concerns on adequacy (2 out of 6 study contributed).
**2.4**	**Positive perceptions of calcium supplements** Women reported that both their perceptions about expected benefits and	(#293 Vestering 2019, #2388 Martin 2018, #2393 Martin	**Minor concerns:** Four out of five studies have minor	**No or very minor concerns**	**Minor concerns:** One study is indirectly relevant to	**Minor concerns** Overall, small number of studies	**High Confidence**	Due to no or very minor concerns on coherence, minor concerns on methodological limitations (ethics and reflexivity), minor concerns on relevance (1 study indirectly

No	Summary of qualitative review findings	Contributing qualitative studies	Methodological limitations assessment	Coherence assessment	Relevance assessment	Adequacy assessment	Overall CERQual assessment	Explanation of overall assessment
	previous experiences of taking calcium and iron-folic acid supplements were facilitators of use. Women believed that consuming pills could compensate for sub-optimal nutrition during pregnancy, and that supplements during pregnancy would help keep their baby safe.	2017, #4971 Birhanu 2016, #1837 Omotayo 2017)	issues (ethics and reflexivity) and one study with no or very minor issues.		review aim and represented 3 countries (Kenya, Ethiopia, Netherlands), including 1 low-income, 1 lower middle income and 1 high income country. All perspectives came from health providers and women.	contributing to the qualitative evidence synthesis.		relevant to review aim and small number of countries) and adequacy (small number of studies contributing to the qualitative evidence synthesis).
**3. Calcium supplement characteristics and regimens**								
3.1	**Varying preferences about characteristics of calcium tablets** Positive perceptions about the characteristics of the calcium tablet played a role in motivating women to take it. Some women preferred the chewable, sweet-tasting tablets that could be swallowed without water, while others preferred the	(#1837 Omotayo 2017, #4971 Birhanu 2016)	**Minor concerns:** One study has minor issues (reflexivity) and one study has no or very minor issues.	**No or very minor concerns**	**Moderate concerns:** All studies are directly relevant to review aim and represented 2 countries (Kenya, Ethiopia), including 1 low-income and 1	**Moderate concerns:** 2 out of 6 studies contributed to findings and both had moderate to thick data.	**Moderate confidence**	Due to no or very minor concerns on coherence, minor concerns on methodological limitations (reflexivity), moderate concerns on relevance (all low or lower middle-income countries and small number of countries) and moderate concerns on adequacy (2 out of 6 studies contributed with moderate to thick data).

For peer review only

No	Summary of qualitative review findings	Contributing qualitative studies	Methodological limitations assessment	Coherence assessment	Relevance assessment	Adequacy assessment	Overall CERQual assessment	Explanation of overall assessment
	hard tablets which were smaller in size, had no smell, and needed to be taken with water. Based on individual preference, the taste, smell, size, and convenience affected calcium supplement use.				lower middle-income country. All perspectives came from women.			
**3.2**	**Supplement regimen as a barrier to use** Women described that they feel overwhelmed with the number of calcium tablets they had to take each day, especially women with other comorbidities who needed to take additional medications for their health conditions. Women felt that 3-4 pills per day at multiple times was overly onerous and preferred if they were combined into one pill.	(# 2388 Martin 2018, #4971 Birhanu 2016, #1837 Omotayo 2017)	**Minor concerns:** Two studies have minor issues (reflexivity) and one study has no or very minor issues.	**No or very minor concerns**	**Moderate concerns:** All studies are directly relevant to review aim and represented 2 countries (Kenya, Ethiopia), including 1 low-income and 1 lower middle-income country. All perspectives came from health providers and women.	**Minor concerns:** 3 out of 6 studies contributing to findings and all have moderate to thick data.	**High Confidence**	No or very minor concerns on coherence, minor concerns on methodological limitations (reflexivity), minor concerns on adequacy (3 out of 6 studies contributed with moderate to thick data), and moderate concerns on relevance (small number of countries).
**4. Daily routines and food insecurity**								

For peer review only

No	Summary of qualitative review findings	Contributing qualitative studies	Methodological limitations assessment	Coherence assessment	Relevance assessment	Adequacy assessment	Overall CERQual assessment	Explanation of overall assessment
**4.1**	**Adherence challenges due to routines** Adherence to calcium supplements consumption was challenging for some women because of conflicting activities in their daily routine, such as consuming other medications, traveling, being away from home, and household chores, which can lead them to forget to take calcium.	(#2388 Martin 2018, #4971 Birhanu 2016, #1837 Omotayo 2017)	**Minor concerns:** Two out of three studies have minor issues (reflexivity) and one study with no or very minor issues.	**No or very minor concerns**	**Moderate concerns:** All studies are directly relevant to review aim and represented 2 countries (Kenya, Ethiopia), including 1 low-income and 1 lower middle-income country. All perspectives came from health providers and women.	**Minor concerns:** Overall, small number of studies contributing to qualitative evidence synthesis.	**High Confidence**	Due to no or very minor concerns with coherence, minor concerns on methodological limitations (reflexivity), moderate concerns on relevance (small number of countries) and moderate concerns on adequacy (small number of studies contributing to qualitative evidence synthesis).
**4.2**	**Food insecurity as a barrier to calcium uptake** Women believed that adequate food was necessary to be able to take the supplements, to avoid nausea. They perceived it as normal to eat before consuming any medication. However, women reported	(#4971 Birhanu 2016, #2388 Martin 2018)	**Minor concerns:** Two studies with minor issues (reflexivity).	**No or very minor concerns**	**Moderate concerns:** All studies are directly relevant to review aim and represented 2 countries (Kenya, Ethiopia),	**Moderate concerns:** 2 out of 6 studies contributed to findings with 1 thick and 1 thin data.	**Moderate Confidence**	Due to no or very minor concern with coherence, minor concerns on methodological limitations (reflexivity), moderate concerns on adequacy (2 out 6 studies contributed with 1 thick and 1 thin data), and moderate concerns on relevance (small number of countries).

For peer review only

No	Summary of qualitative review findings	Contributing qualitative studies	Methodological limitations assessment	Coherence assessment	Relevance assessment	Adequacy assessment	Overall CERQual assessment	Explanation of overall assessment
	that food insecurity was a critical barrier to calcium uptake.				including 1 low-income and 1 lower middle-income country. All perspectives came from health providers and women.			
**5. Strategies to improve the use of calcium supplements**								
**5.1**	**Implementation of reminders to promote adherence** Women and healthcare providers perceived reminders as beneficial in promoting women's adherence to consuming calcium supplements. Several reminder strategies were described as useful by women and healthcare providers, such as home-based posters, calendars with illustrations and daily reminders, and integrating supplement consumption into women's daily routine, such as mealtimes.	(#2389 Martin 2016, #2388 Martin 2018, #4971 Birhanu 2016, #1837 Omotayo 2017)	**No or very minor concerns**	**No or very minor concerns**	**Moderate concerns:** One study indirectly relevant to review aim and represented 2 countries (Kenya, Ethiopia), including 1 low-income and 1 lower middle-income country. All perspectives came from health providers and women.	**Minor concerns:** Overall, small numbers of studies contributing to qualitative evidence synthesis.	**High Confidence**	Due to no or very minor concerns on methodological limitations, no or very minor concerns on coherence, minor concerns on adequacy (small number of studies contributing to qualitative evidence synthesis), moderate concerns on relevance (due to all studies coming from low income or lower middle-income country and small number of countries).

For peer review only

No	Summary of qualitative review findings	Contributing qualitative studies	Methodological limitations assessment	Coherence assessment	Relevance assessment	Adequacy assessment	Overall CERQual assessment	Explanation of overall assessment
**5.2**	**Importance of family support and 'adherence partner' implementation** Having family support was instrumental to pregnant women adhering to calcium supplement use. This could be leveraged by notifying family members on the importance of calcium, and appointing someone to be an "adherence partner" or "pill buddy" to help remind her to take it. Both women and healthcare providers were positive about adherence partners in providing support in terms of encouraging them to take the supplements, providing food, helping them around the house, providing emotional support, improving family relationships, and thereby increasing partner or husband involvement in pregnancy.	(#2388 Martin 2018, #2393 Martin 2017, #4972 Birhanu 2016, #2389 Martin 2016, #1837 Omotayo 2017)	**No or very minor concerns**	**No or very minor concerns**	**Moderate concerns:** Two out of five studies indirectly relevant to review aim and represented 2 countries (Kenya, Ethiopia), including 1 low-income and 1 lower middle-income country. All perspectives came from health providers, partner and women.	**Minor concerns:** Overall, small number of studies contributing to qualitative evidence synthesis.	**High Confidence**	No or very minor concerns on methodological limitations, no or very minor concerns on coherence, minor concerns on adequacy (small number of studies contributing to qualitative evidence synthesis), and moderate concerns on relevance (due to all studies coming from low income or lower middle-income country only, 2 of 5 studies has indirectly relevant aim, and small number of countries).

For peer review only

No	Summary of qualitative review findings	Contributing qualitative studies	Methodological limitations assessment	Coherence assessment	Relevance assessment	Adequacy assessment	Overall CERQual assessment	Explanation of overall assessment
**5.3**	**Counselling facilitates calcium supplements uptake** Both women and healthcare providers acknowledged that counselling women on the benefits of calcium was a motivator to calcium supplements uptake. Women valued the discussion they have with healthcare providers and felt more confident to take calcium supplements when they received counselling and information and pre-eclampsia or eclampsia and the benefits of calcium from their healthcare providers.	(#4971 Birhanu 2016, #2388 Martin 2018)	**No or very minor concerns**	**No or very minor concerns**	**Moderate concerns:** All studies are directly relevant to review aim and represented 2 countries (Kenya, Ethiopia), including 1 low-income and 1 lower middle-income country. All perspectives came from health providers and women.	**Moderate concerns:** 2 out of 6 studies contributing to findings with all thick data.	**Moderate Confidence**	No or very minor concerns on methodological limitations, no or very minor concerns on coherence, moderate concerns on relevance (due to all studies coming from low or lower middle-income country and small number of countries) and moderate concerns on adequacy (2 out 6 studies contributed with thick data).
**Health care providers' factors**								
**6. Healthcare provider knowledge and training**								
**6.1**	**Varied knowledge about pre-eclampsia or eclampsia among healthcare providers** Healthcare providers' knowledge about pre-	(#4971 Birhanu 2016, #2389 Martin 2016, #5970	**No or very minor concerns**	**No or very minor concerns**	**Moderate concerns:** One study is indirectly relevant to	**Moderate concerns:** 2 out of 6 studies contributed to	**Moderate Confidence**	Due to no or very minor concerns on methodological limitations and coherence, moderate concerns on relevance (due to all studies coming from low or lower middle-

No	Summary of qualitative review findings	Contributing qualitative studies	Methodological limitations assessment	Coherence assessment	Relevance assessment	Adequacy assessment	Overall CERQual assessment	Explanation of overall assessment
	eclampsia and eclampsia was varied. Some felt that pre-eclampsia and eclampsia is not a priority health concern in their area and reported never having encountered any case.	Kachwaha 2022)			review aim and represented 2 countries (Kenya, Ethiopia), including 1 low-income and 1 lower middle-income country. All perspectives came from health providers.	findings with thick data.		income countries and small number of studies) and moderate concerns on adequacy (2 out 6 studies contributed with thick data).
6.2	**Inadequate training to diagnose and treat pre-eclampsia and eclampsia** While some healthcare providers mentioned that training about pre-eclampsia or eclampsia and calcium supplements was adequate, others reported that their training lacked depth and continuity, and thus felt unprepared to diagnose it and offer information these conditions and the use of calcium for prevention.	(#2388 Martin 2018, #4971 Birhanu 2016, #2389 Martin 2016)	**No or very minor concerns**	**No or very minor concerns**	**Moderate concerns:** One study is indirectly relevant to review aim and represented 2 countries (Kenya, Ethiopia), including 1 low-income and 1 lower middle-income country. All perspectives	**Minor concerns:** 3 out of 6 studies contributed with 2 thick and 1 thin data.	**High Confidence**	No or very minor concerns on methodological limitations, coherence, moderate concerns on relevance (due to all studies coming from low income or lower middle-income country and small number of countries) and minor concerns on adequacy (3 out of 6 studies contributed with 2 thick and 1 thin data).

For peer review only

No	Summary of qualitative review findings	Contributing qualitative studies	Methodological limitations assessment	Coherence assessment	Relevance assessment	Adequacy assessment	Overall CERQual assessment	Explanation of overall assessment
	Healthcare providers expressed the need to have more and continuous training on managing pre-eclampsia, as well as time to address concerns or resistance from the community.				came from health providers.			
**7. Beliefs about the intervention**								
**7.1**	**Perceived overmedicalization when prescribing calcium supplements** Both healthcare providers and women perceived that prescribing more tablets to "low-risk" women during pregnancy was a form of over-medicalization of pregnancy. However, some healthcare providers felt that calcium supplements were a way to prevent further medicalization due to pre-eclampsia-related complications.	(#293 Vestering 2019)	**Minor concerns:** One study with minor issues (reflexivity and ethics).	**No or very minor concerns**	**Serious concerns:** One study is directly relevant to review aim and represented 1 country (Netherlands), which is high income country. All perspectives came from health providers and women.	**Serious concerns:** 1 out of 6 study contributed to findings with thick data.	**Low confidence**	No or very minor concerns on coherence, minor concerns on methodological limitations (reflexivity and ethics), serious concerns on relevance (evidence coming from high income country only), serious concerns on adequacy (1 out of 6 study contributed).

For peer review only

No	Summary of qualitative review findings	Contributing qualitative studies	Methodological limitations assessment	Coherence assessment	Relevance assessment	Adequacy assessment	Overall CERQual assessment	Explanation of overall assessment
**7.2**	**Beliefs about calcium supplements** Healthcare providers generally had positive beliefs about calcium supplements and there was optimism that calcium could be delivered through antenatal care healthcare providers. Some facilitators motivating healthcare providers to prescribe calcium supplements included their beliefs in its prevention value and expected benefits, that women liked the calcium supplements and experienced benefits from it, and a perceived lack of knowledge on how to treat pre-eclampsia which motivated healthcare providers to side towards prevention.	(#2388 Martin 2018, #293 Vestering 2019, #4971 Birhanu 2016)	**Minor concerns:** Two studies with minor issues (ethics and reflexivity).	**No or very minor concerns**	**Minor concerns:** All studies are directly relevant to review aim and represented 3 countries (Kenya, Ethiopia, Netherlands), including 1 low-income, 1 lower middle income and 1 high income country. All perspectives came from health providers.	**Minor concerns:** 3 out of 6 studies contributed with 2 thick and 1 thin data.	**High Confidence**	No or very minor concerns on coherence, minor concerns on relevance (small number of countries), minor concerns on methodological limitations (ethics and reflexivity), and minor concerns on adequacy (3 out of 6 studies contributed with 2 thick and 1 thin data).
**8. Structural factors**								
**8.1**	**High workload, inadequate staffing, stock out, and lack of equipment**	(#2388 Martin 2018, #4971 Birhanu	**No or very minor concerns**	**No or very minor concerns**	**Moderate concerns:** All studies are directly relevant	**Serious concerns:** 2 out 6 studies contributed to	**Moderate Confidence**	No or very minor concerns on methodological limitations, no or very minor concerns on coherence, minor concerns on

For peer review only

No	Summary of qualitative review findings	Contributing qualitative studies	Methodological limitations assessment	Coherence assessment	Relevance assessment	Adequacy assessment	Overall CERQual assessment	Explanation of overall assessment
	In general, healthcare providers felt that their workload increased by including calcium supplements in antenatal care for pregnant women. Healthcare providers reported existing inadequate staffing, yet they needed to provide additional counselling and prescription to women, especially pregnant women with comorbidities.	2016)			to review aim and represented 2 countries (Kenya, Ethiopia), including 1 low-income and 1 lower middle-income country. All perspectives came from health providers.	findings (1 thick, 1 thin data).		relevance (due to all studies coming from low income or lower middle-income countries and small number of countries) and serious concerns on adequacy (2 out 6 studies contributed).

For peer review only

BMJ Open

Factors affecting the implementation of calcium supplements strategies during pregnancy to prevent pre-eclampsia: a mixed methods systematic review

Journal:	*BMJ Open*
Manuscript ID	bmjopen-2022-070677.R1
Article Type:	Original research
Date Submitted by the Author:	09-Aug-2023
Complete List of Authors:	Cormick, G; CONICET,<br>Moraa, Helen; University of Nairobi<br>Zahroh, Rana; University of Melbourne<br>Allotey, John; Univ Birmingham<br>Rocha, Thaís; University of Birmingham<br>Pena-Rosas, Juan-Pablo; Organisation mondiale de la Sante, Nutrition for Health and Development<br>Qureshi , Zahida ; University of Nairobi, Obstetrics and Gynaecology<br>Hofmeyr, G Justus ; University of the Witwatersrand Faculty of Health Sciences, Effective Care Research Unit<br>Mistry, Hema; University of Birmingham<br>Smits, Luc; Maastricht University<br>Vogel, Joshua; Burnet Institute; World Health Organization, Reproductive Health and Research<br>Palacios , Alfredo ; Institute for Clinical Effectiveness and Health Policy<br>Gwako, George N. ; University of Nairobi, Obstetrics and Gynaecology ;<br>Abalos, E; Univ Western Australia<br>Larbi, Koiwah Koi; ACTION ON PREECLAMPSIA<br>Carroli, Guillermo; CREP<br>Riley, Richard; University of Birmingham<br>Snell, Kym IE; Keele University<br>Thorson, Anna; Human Reproduction Program/World Health Organization (Geneva)<br>Young, Taryn; Stellenbosch University, Centre for Evidence-based Health Care, Division Epidemiology and Biostatistics, Dept. of Global Health<br>Betran, Ana Pilar; World Hlth Org, Reproductive Health and Research<br>Thangaratinam, Shakila; Institute of Metabolism and Systems Research University of Birmingham<br>Bohren, Meghan ; University of Melbourne School of Population and Global Health, Centre for Health Equity
**Primary Subject Heading**:	Obstetrics and gynaecology
Secondary Subject Heading:	Qualitative research, Public health, Health policy, Global health
Keywords:	Hypertension < CARDIOLOGY, OBSTETRICS, PUBLIC HEALTH

I, the Submitting Author has the right to grant and does grant on behalf of all authors of the Work (as defined in the below author licence), an exclusive licence and/or a non-exclusive licence for contributions from authors who are: i) UK Crown employees; ii) where BMJ has agreed a CC-BY licence shall apply, and/or iii) in accordance with the terms applicable for US Federal Government officers or employees acting as part of their official duties; on a worldwide, perpetual, irrevocable, royalty-free basis to BMJ Publishing Group Ltd ("BMJ") its licensees and where the relevant Journal is co-owned by BMJ to the co-owners of the Journal, to publish the Work in this journal and any other BMJ products and to exploit all rights, as set out in our licence.

The Submitting Author accepts and understands that any supply made under these terms is made by BMJ to the Submitting Author unless you are acting as an employee on behalf of your employer or a postgraduate student of an affiliated institution which is paying any applicable article publishing charge ("APC") for Open Access articles. Where the Submitting Author wishes to make the Work available on an Open Access basis (and intends to pay the relevant APC), the terms of reuse of such Open Access shall be governed by a Creative Commons licence – details of these licences and which Creative Commons licence will apply to this Work are set out in our licence referred to above.

Other than as permitted in any relevant BMJ Author's Self Archiving Policies, I confirm this Work has not been accepted for publication elsewhere, is not being considered for publication elsewhere and does not duplicate material already published. I confirm all authors consent to publication of this Work and authorise the granting of this licence.

For peer review only

Factors affecting the implementation of calcium supplements strategies during pregnancy to prevent pre-eclampsia: a mixed-methods systematic review

Gabriela Cormick [1,2,3], Helen Moraa [4], Rana Zahroh [5], John Allotey [6], Thais Rocha [6], Juan Pablo Peña-Rosas [7], Zahida Qureshi [8], G Justus Hofmeyr [9], Hema Mistry [10], Luc JM Smits [11], Joshua P. Vogel [12], Alfredo Palacios [13], Gwako George Nyakundi[4], Edgardo Abalos [14], Koiwah Koi-Larbi [15], Guillermo Carroli [16], Richard Riley [17], Kym Snell[17], Anna Thorson [18], Taryn Young [19], Ana Pilar Betran[18], Shakila Thangaratinam [6,17], Meghan A. Bohren[5] for I-CIP Collaborative Network

1. Centro de Investigaciones Epidemiológicas y Salud Pública (CIESP-IECS), CONICET, Buenos Aires, Argentina
2. Department of Mother and Child Health Research, Institute for Clinical Effectiveness and Health Policy (IECS-CONICET), Buenos Aires, Argentina
3. Departamento de Salud, Universidad Nacional de La Matanza (UNLAM), San Justo 1903, Argentina.
4. University of Nairobi
5. Gender and Women's Health Unit, Nossal Institute for Global Health, School of Population and Global Health, University of Melbourne, Carlton, Victoria, Australia
6. WHO Collaborating Centre for Global Women's Health, Institute of Metabolism and Systems Research, University of Birmingham, Birmingham, UK
7. Department of Nutrition and Food Safety, World Health Organization, Geneva, Switzerland
8. Department of Obstetrics and Gynaecology, University of Nairobi, Nairobi Kenya.
9. Department of Obstetrics and Gynaecology, University of Botswana, Gaborone, Botswana; Effective Care Research Unit, University of the Witwatersrand and Walter Sisulu University, East London, South Africa.
10. Clinical Trials Unit, Warwick Medical School, University of Warwick, Coventry, CV4 7AL, United Kingdom; University Hospitals Coventry and Warwickshire NHS Trust, Coventry, CV2 2DX, United Kingdom
11. Department of Epidemiology, CAPHRI Care and Public Health Research Institute, Maastricht University, Maastricht, The Netherlands
12. Maternal, Child and Adolescent Health Program, Burnet Institute, 85 Commercial Road, Melbourne, 3000, Australia

13. Department of Health Technology Assessment and Health Economics, Institute for Clinical Effectiveness and Health Policy (IECS), Buenos Aires, Argentina; Centre for Health Economics (CHE), University of York, York, United Kingdom
14. Centro de estudios de Estado y Sociedad, Buenos Aires, Argentina
15. Action on preeclampsia Charity (APEC), Ghana
16. Centro Rosarino de Estudios Perinatales (CREP), Rosario, Argentina
17. Institute of Applied Health Research, University of Birmingham, Birmingham, UK
18. UNDP-UNFPA-UNICEF-WHO-World Bank Special Programme of Research Development and Research Training in Human Reproduction (HRP), Department of Sexual and Reproductive Health and Research, World Health Organization, Geneva, Switzerland.
19. Centre for Evidence-based Health Care, Division of Epidemiology and Biostatistics, Department of Global Health, Faculty of Medicine and Health Sciences, Stellenbosch University, Stellenbosch, South Africa
20. Birmingham Women's and Children's NHS Foundation Trust, Birmingham, UK

Corresponding author: Gabriela Cormick, email: gabmick@yahoo.co.uk

For peer review only

Abstract (300 words)

Objectives Daily calcium supplements are recommended for pregnant women from 20 weeks' gestation to prevent pre-eclampsia in populations with low dietary calcium intake. This systematic review aims to improve understanding of barriers and facilitators for calcium supplement intake during pregnancy to prevent pre-eclampsia.

Design We conducted mixed-method systematic review and assessed confidence with GRADE-CERQual approach.

Data sources MEDLINE and EMBASE (via Ovid), CINAHL, Global Health, and grey literature databases were searched through 17 September 2022.

Eligibility criteria We included primary qualitative, quantitative, and mixed-methods studies reporting implementation or use of calcium supplements during pregnancy, excluding calcium fortification and non-primary studies. No restrictions were imposed on settings, language, or date.

Data extraction and synthesis Two independent reviewers extracted data and assessed risk of bias. We analysed the qualitative data using thematic synthesis, and quantitative findings were thematically mapped to qualitative findings. We then mapped the results to behavioural change frameworks to identify barriers and facilitators.

Results Eighteen reports from nine studies are included in this review. Women reported barriers to consuming calcium supplements included limited knowledge about calcium supplements and pre-eclampsia, fears and experiences of side effects, varying preferences for tablets, dosing, working schedules, being away from home and taking other supplements. Receiving information regarding pre-eclampsia and safety of calcium supplement use from reliable sources, alternative dosing options, supplement reminders, early antenatal care, free supplements and support from families and communities were reported as facilitators for women. Healthcare providers felt that consistent messaging about benefits and risks of calcium, training, and ensuring adequate staffing and calcium supply is available would be able to help them in promoting calcium.

Conclusion Relevant stakeholders should consider the identified barriers and facilitators when formulating intervention and policies on calcium supplement use. These review findings can inform implementation, to ensure effective and equitable provision and scale-up of calcium interventions.

PROSPERO registration number: CRD42021239143.

For peer review only - http://bmjopen.bmj.com/site/about/guidelines.xhtml

Strengths and limitations of this study

- Our systematic review is the first systematic review of barriers and facilitators to calcium supplement use during pregnancy.
- We adopted a mixed methods approach which allowed for inclusion and consolidation of studies with a range of designs.
- The strength of our review lies on behaviour change frameworks mapping which improved our understanding of how barriers and facilitators influence calcium uptake and potential strategies to address them.
- The transferability of our results may be limited, as studies were mostly from low- and middle-income countries and almost half came from the same project in Kenya and Ethiopia.
- Most included studies engaged women who attended antenatal care, therefore, might not be representative of those that do not reach the health system during pregnancy.

For peer review only

Introduction

Hypertensive disorders of pregnancy are among the leading causes of maternal and perinatal morbidity and mortality globally.(1) Pre-eclampsia is a hypertensive disorder of pregnancy characterised by hypertension developing after 20 weeks' gestation, combined with proteinuria or other new onset of maternal organ dysfunction, while eclampsia is a severe form of pre-eclampsia characterised by seizures.(2,3) Pre-eclampsia contribute to approximately 14% of the 300,000 maternal deaths worldwide annually.(4) Management of pre-eclampsia requires regular monitoring and evaluation of the woman and her baby to achieve an optimal timing of birth and prevent severe complications.(5) Preventive strategies are essential to reduce the burden of morbidity and mortality, especially in low- and middle-income countries (LMICs) where most complications occur.

The World Health Organization (WHO) recommends 1.5 to 2 grams a day from 20 weeks' gestation for women who are living in populations with low dietary calcium intake, especially those at high risk of developing pre-eclampsia. (6) This is aligned with findings from a systematic review that reported calcium supplements during pregnancy compared to placebo may reduce the risk of pre-eclampsia by 55% (13 trials, 15,730 participants; Relative Risk (RR) 0.45, 95% CI: 0.31 to 0. 65).(7) Moreover, maternal death or severe morbidity was reduced by 20% with calcium supplements (four trials, 9,732 participants; RR 0.80, 95% CI 0.66 to 0.98). (7) The evidence base has since been updated multiple times, with WHO recommendation consistently updated up to 2018.(8–10)

Despite the WHO recommendation, calcium supplement during pregnancy remains low in LMICs and rates of pre-eclampsia are not falling in regions where calcium supplementation is recommended.(10) Practical challenges to implementing WHO recommendations have been documented. For example, women need to take three, spaced tablets to achieve the requisite daily dose, and this needs be separated from timing of intake of other supplements (such as iron) to optimise calcium absorption.(11) In addition, antenatal care services need consistent supplies of calcium tablets, which can be hindered by logistical issues in supplement distribution and storage.(7) We conducted a mixed-methods systematic review aiming to improve understanding of the barriers and facilitators of calcium supplement intake during pregnancy to prevent pre-eclampsia, from the perspectives of women, families, community members, healthcare providers, and policymakers.

For peer review only - http://bmjopen.bmj.com/site/about/guidelines.xhtml

Methods

This mixed-methods review is reported according to Preferred Reporting Items for Systematic Reviews and Meta-Analyses (PRISMA) (Appendix 1) (12), Enhancing Transparency in Reporting the Synthesis of Qualitative Research (ENTREQ) statement (Appendix 2) (13), and based on guidance from Cochrane Effective Practice and Organisation of Care.(14) The protocol was registered on PROSPERO (CRD42021239143).

Topic of interest and types of studies

We included studies that documented perspectives, perceptions and experiences of women who experienced or were at risk of pre-eclampsia and/or received calcium-containing supplements. We also included studies on the views of their partners or families, as well as studies on maternity healthcare providers (e.g., midwives, nurses, doctors) and other relevant stakeholders (e.g., facility managers, policymakers) involved in decisions on calcium supplements in pregnancy. There were no limitations imposed on geographical location or type of health facility. The timeframe for using calcium supplement is during pregnancy, independent of the gestational age.

We included primary qualitative, quantitative, and mixed-methods studies reporting implementation or use of calcium supplements in any presentations including powder, granule, chewable tablet, capsule, liquid filled capsule, tablet, suspension, or powder for suspension. We did not include studies assessing the effects of calcium fortified foods or beverages. We excluded case reports or case series, letters, editorials, commentaries, reviews, study protocols, posters, and conference abstracts or other study sources that did not provide primary data.

Search methods for identification of studies

We searched MEDLINE and EMBASE via Ovid, CINAHL, Global Health databases to identify eligible studies from inception to 17 September 2022. A search strategy was developed and adapted for each database (Appendix 3), using different terms for calcium and pregnancy. No limitations on publication date or language were imposed. Grey literature searches were also conducted using OpenGrey and Google. We checked reference lists of included studies to identify any relevant record not retrieved in the database search.

Selection of studies

After removing duplicates in EndNote, records were imported to Covidence for screening.(15) Two of the following authors (GC, GG, APB, RIZ, HM) independently assessed eligibility of each record by comparing titles and abstracts against the eligibility criteria. Full texts of potentially eligible papers were retrieved and assessed, disagreements were resolved through discussion or consulting a third author. Papers emerging from the same study were collated and treated as one data source. Titles and abstracts of papers published in languages other than English, French, and Spanish were translated through open-source software (Google Translate) to assess their eligibility. Had we identified any relevant titles or abstracts

For peer review only - http://bmjopen.bmj.com/site/about/guidelines.xhtml

in languages other than English, French and Spanish, we would have sought formal translation of the full texts from a native speaker.

Data extraction and assessing the methodological limitations

Using a pre-designed form, two reviewers (GC, RIZ) independently extracted data from included studies on study characteristics (setting, sample size, characteristics of participants, objectives), design (data collection and analysis methods), qualitative data (themes, findings, and quotations) and quantitative data (data source, outcome measures, results, measures of compliance or uptake).

All included studies underwent quality appraisal by two authors (GC, HM, RIZ). As the review included quantitative, qualitative and mixed-methods studies, we used the Mixed Methods Appraisal Tool (MMAT), which produces a single quality rating on the basis of: aims, methodology, design, recruitment, data collection, blinding, data analysis, selective reporting, reflexivity, ethical considerations, results, research contribution, and other sources of bias.(16) Appraisal of study quality was used to inform data analysis, and not to exclude studies.

Quality appraisal, analysis and assessing confidence

We conducted a preliminary qualitative synthesis using a thematic analysis approach.(17) Thematic analysis is a valuable method in analysing qualitative data to examine perspectives, preferences, experiences, acceptability, feasibility, and other factors that can influence implementation.(17) The analysis begins with initial readings to build our familiarity with the data. Two reviewers (GC, HM) independently conducted line-by-line coding of findings of two qualitative studies.(18,19) From this we developed the qualitative codebook, which was then used to code all other included studies. Next, we generated analytical themes and interpretations to explore relationships within and across studies. This was achieved by organising codes into a hierarchy and identifying barriers and facilitators between study characteristics and findings or exploring different findings across studies. Once qualitative themes were generated, a summary of qualitative findings was developed. Quantitative findings were then narratively mapped to qualitative themes to explore areas of convergence or divergence. ATLAS.ti was used to manage data analysis.

After the thematic analysis, we mapped the qualitative themes to the Theoretical Domains Framework (TDF) and Capability, Opportunity, and Motivation of Behaviour (COM-B) models.(20,21) TDF and COM-B are interrelated behaviour change models which can guide implementation research and intervention design in understanding barriers and facilitators of intended behaviours. We used TDF and COM-B to explore barriers and facilitators of healthcare providers and women to use calcium supplements during pregnancy using evidence-based behaviours to identify potential behaviour change intervention strategies.

We used the GRADE-CERQual (Grading of Recommendations, Assessment, Development, and Evaluations - Confidence in the Evidence from Reviews of Qualitative research) approach to assess confidence in each qualitative finding. (22) GRADE-CERQual assessed confidence based on four key components: methodological limitations (23), coherence (24), adequacy (25), and

For peer review only - http://bmjopen.bmj.com/site/about/guidelines.xhtml

relevance. (26) After assessing each of the four components, we assessed the overall confidence (22) as high, moderate, low, or very low.

Patient and Public Involvement

None.

Results

We included 18 papers from nine studies (Figure 1). Included papers were published in English between 2014 and 2022. Out of 18 papers, 11 were quantitative (27–37), four were qualitative (18,19,38,39) and three were mixed-methods. (40–42) Detailed study characteristics can be found in Table 1.

For peer review only

For peer review only - http://bmjopen.bmj.com/site/about/guidelines.xhtml

Table 1. Characteristics of included studies

Project Name	Authors	Year	Country	Study designs	Sample Size	Participants
**Qualitative**						
**Micronutrient Initiative-Cornell University Calcium (MICa) trial**	Birhanu	2016	Ethiopia	Phenomology	20 women, 22 healthcare providers	Pregnant women and healthcare providers
	Martin	2016	Kenya	Phenomology	22 women, 20 healthcare providers	Pregnant women and healthcare providers
	Martin	2018	Kenya	Phenomology	7 healthcare providers, 32 pregnant women, 20 adherence partners.	Healthcare providers, pregnant women, adherence partners
**Individual study**	Vestering	2019	Netherlands	Phenomology	8 healthcare providers, 25 women	Healthcare providers and pregnant women
**Mixed methods**						
**Micronutrient Initiative-Cornell University Calcium (MICa) trial**	Omotayo	2017	Kenya	Trials of improved practices	38 pregnant women	Pregnant women
	Martin	2017	Kenya and Ethiopia	Trials of improved practices	85 pregnant women, 50 in Ethiopia, 35 in Kenya	Pregnant women
**Alive & Thrive (A&T) trial**	Kachwaha	2022	India	Mixed methods	~500 healthcare providers and supervisors, and 20 block level staffs	healthcare providers, supervisors and facility staffs
**Quantitative**						
**Micronutrient Initiative-Cornell University Calcium (MICa) trial**	Martin	2017	Kenya	Secondary analysis of randomized controlled trial	1036 pregnant women	Pregnant women
	Omotayo	2018	Kenya	Process evaluation study adopting program impact pathway	990 women and 16 facilities (unclear how many healthcare providers participated)	Pregnant women, healthcare providers, community healthcare providers
**Alive & Thrive (A&T) trial**	Nguyen	2019	India	Baseline household survey	667 pregnant women and 1835 recently delivered women	Pregnant women and recently delivered women
**Alive & Thrive (A&T) trial**	Nguyen	2017	Bangladesh	Cross-sectional study	600 pregnant women and 2000 recently delivered women	Pregnant women and recently delivered women
**Alive & Thrive (A&T) trial**	Nguyen	2018	Bangladesh	Cluster randomised control trial with cross-sectional household surveys	1000 women and 70% of their husbands	Women and husbands
**Individual study**	Bora	2022	India	Cross-sectional study	3,097,274 pregnant women	Pregnant women
**Individual study**	Baxter	2014	Bangladesh	Modified discrete-choice trial	132 pregnant women	Pregnant women
**Individual study**	Thapa	2016	Nepal	Prospective collection and secondary analysis of monitoring data captured by the MOHP	1240 women	Pregnant and postpartum women, health facilities, female community health volunteers
**Individual study**	Liu	2019	China	Cross-sectional study	30,027 women	Women aged from 16 to 49 years who were pregnant between 2010 and 2013 and had specific pregnancy outcomes before the survey
**Individual study**	Shakya Shrestha	2020	Nepal	Cross-sectional study	191 pregnant women	Pregnant women

For peer review only - http://bmjopen.bmj.com/site/about/guidelines.xhtml

**Individual study**	Ghosh-Jareth	2015	India	Cross-sectional study	184 pregnant women and 160 recently delivered women	Pregnant women and recently delivered women
	11 Bora	2022	India	Cross-sectional study	3,097,274 pregnant women	Pregnant women

Three studies aimed to evaluate the implementation of calcium supplements in pregnancy (5 papers). (27,30,31,39,40) Four studies evaluated the incorporation of calcium supplement recommendation to other recommended supplements taken during pregnancy, including aspirin (1 paper) (18), and iron and folic acid supplements (7 papers). (19,28,33,37,38,41,42) Two studies focused on general nutritional practices during pregnancy (4 papers) (29,32,34,35) and one study on all types of micronutrient supplements used before and during pregnancy (1 paper). (36)

The studies were conducted in seven different countries across four regions. One study, the MICa Trial, was conducted in Kenya and Ethiopia study (7 papers). (19,28,33,38–41) One study conducted in Bangladesh and India (5 papers) (27,32,34,35,42), one study only in Bangladesh (1 paper) (30), two studies only in India (2 papers) (27,29), and two studies in Nepal (2 papers). (31,37) The remaining two studies were conducted in China (36), and the Netherlands. (18)

All four qualitative papers involved pregnant women and healthcare providers (3 papers) (18,19,38), while one report also included adherence partners to support and remind women to take medication (1 paper). (39) Two mixed-methods papers included pregnant women (40,41) and one included healthcare providers and facility staff. (42) Among the ten quantitative papers, eight included pregnant women or women who had recently given birth (27–30,32,34,36,37), two included healthcare providers (31,33) and one included both women and their husbands. (35)

Results of critical appraisal of included studies is available in Appendix 4. The main areas of concern for qualitative studies were an unclear or partial description of reflexivity and limited information regarding ethical considerations. For the quantitative studies, main concerns were regarding the appropriateness of measurement tools, lack of detail regarding nonresponse bias, and insufficient information regarding statistical analysis.

Qualitative and quantitative synthesis

We identified five themes related to factors affecting calcium supplement intake during pregnancy: 1) women's existing knowledge and learning; 2) women's beliefs about calcium supplements; 3) calcium supplement characteristics and dose regimens; 4) challenges due to daily routines and food insecurity; and 5) strategies to improve the use of calcium. We also identified three themes related to factors affecting healthcare providers' prescription of calcium supplements: 1) health provider knowledge and training, 2) their beliefs about calcium supplements, 3) structural factors on site. Across all themes there were 19 qualitative findings (Appendix 5): 11 findings were high confidence, six were moderate confidence and two were low confidence (Appendix 6: Evidence profile). The quantitative findings which were mapped to qualitative themes can be found in Appendix 7.

For peer review only

For peer review only - http://bmjopen.bmj.com/site/about/guidelines.xhtml

Women’s knowledge and learning

Women’s knowledge about pre-eclampsia

Most women had limited knowledge about pre-eclampsia, and these conditions were not typically considered by women to be a serious problem. Symptoms of pre-eclampsia such as swollen feet, severe headache, blurred vision and vomiting were considered normal signs and symptoms in pregnancy, while seizures were associated with evil attacks or nutritional deficiencies (Finding 1.1 – High confidence). (18,19,38) Women and healthcare providers from Ethiopia and Kenya stated that there was no local language for pre-eclampsia, which made it difficult for healthcare providers to explain the condition to women, and served as a barrier in providing adequate knowledge to women and encouraging them to use calcium supplements. (19,38)

Information provision to women

Women felt they did not receive adequate information during pregnancy from healthcare providers about pre-eclampsia and calcium supplements. They described wanting to be given more information, regardless of their pre-eclampsia risk status. Women believed that having this essential information could help them to make informed choices and actively participate in their care. There were, however, mixed opinions from healthcare providers - some feared that more information could generate anxiety for women, while others were more supportive of providing information (Finding 1.2 – Low confidence). (18,39) Healthcare providers were worried that sharing information about pre-eclampsia, especially with low-risk pregnant women, could lead to anxiety and make an “uncomplicated pregnancy more stressful”. (18) Healthcare providers viewed their role as informants, but not as decision-makers for women. They believed that it should be woman’s choice to decide whether to consume calcium supplements or not. (18) Women and healthcare providers mentioned that the scope of information provided to women should include symptoms of pre-eclampsia as well as effectiveness, benefits, and safety of calcium-containing supplements. (18)

Learning about calcium supplements

Women typically learned about dietary calcium, including pre-eclampsia symptoms, from healthcare providers. They considered healthcare providers to be the most trusted and reliable source of information and reported feeling confident about taking calcium-containing supplements after receiving adequate information from them. Women also appreciated receiving information on calcium supplements and pre-eclampsia via information, education, and communication (IEC) materials like videos, media campaigns, and trusted websites (Finding 1.3 – High confidence). (18,19,32,34,39) Quantitative evidence supported qualitative findings regarding women’s learning on calcium supplements. In the context of an intervention implementing training to healthcare providers to reinforce calcium-related messages to women, women reported that they would take calcium in a future pregnancy and that they would recommend calcium to other pregnant women. (31)

For peer review only

Women were more likely to consume calcium supplements if they had higher knowledge of calcium benefits (Odds Ratio (OR) 11.7, 95% Confidence Interval (CI): 5.97-22.86) and higher general education (OR 2.59, 95% CI: 2.21-3.05). (32) Higher adherence was also reported in those with higher education (OR 1.45, 95% CI: CI 1.31 to 1.60).(36) Higher nutrition knowledge was also associated with taking 6 times more calcium tablets.(32)
One paper, evaluating calcium supplement intake before and after nutritional interventions, showed an association between maternal knowledge of nutrition and higher calcium supplement intake (β ~31.9, 95% CI: 20.9, 43.0), however the paper also highlighted there were still large gaps between knowledge and practices, as the intake of calcium supplement tablets during 6 month was low, 82 ±66 out of the recommended 180 tablets.(34)

Women's beliefs about calcium supplements in pregnancy

Fears about side-effects as a barrier to calcium supplements uptake

Women's fears about the side effects of calcium supplements affected their adherence. Women highlighted that assurance of safe use of calcium supplement is a key facilitator to consistent use. However, some women felt safety was not assured by healthcare providers, especially when calcium supplements were perceived as "experimental". Women had also received messages from their families or communities that any pills consumed during pregnancy could be harmful. (Finding 2.1 – High confidence). (18,19,39,40) Quantitative evidence supported qualitative findings as few women reported being discouraged to take supplements by friends (4%) and elder women (3%).(28)

Women's experiences of side-effects

Some women reported experiencing side effects after taking calcium and iron-folic acid supplements, such as dizziness, vomiting, nausea, stomach aches, loss of appetite, tiredness, diarrhoea, bloating, and burping, yet noted that side effects subsided with time. Women also reported that they continued taking calcium supplements despite these side effects (Finding 2.2 – High confidence). (19,39,40) Quantitative evidence supported the qualitative findings as some women reported experiencing side effects, usually related to gastrointestinal symptoms. (29–31,37) The reported side-effects rates were usually low, 4% of women mentioning nausea or vomiting or constipation in one paper while in another paper 14.9% of women reported that side effects was the reason for missing a dose of IFA or calcium.(30,37) These could cause supplement discontinuation or erratic supplement intake for one to 10 days after side effects were felt in around 5% of women.(37)

Concerns about being stigmatized as HIV patients

Women expressed concerns that if they took calcium supplements, they could be stigmatized as HIV patients, which was a reported barrier to use. Some women were afraid of being stigmatized as their community often associated nutritional supplement intake and accompanying reminder posters, with HIV (Finding 2.3 – Moderate confidence). (18,39)

For peer review only - http://bmjopen.bmj.com/site/about/guidelines.xhtml

For peer review only

Positive perceptions of calcium supplements

Women reported that both their perceptions about expected benefits and previous experiences of taking calcium and iron-folic acid supplements were facilitators of use. Women believed that taking pills could compensate for sub-optimal nutrition during pregnancy, and that supplements during pregnancy would help keep their baby safe (Finding 2.4 – High confidence). (18,19,39–41) Women also reported reduced cravings to consume soil (pica) during pregnancy (a cultural practice or cravings characterised by recurrent ingestion of unusually high amounts of soil, and is related to iron deficiency anaemia, which is common during pregnancy). (39) Furthermore, some women appreciated the emphasis of "prevention" when encouraging supplement intake. (19,41) Quantitative evidence supported the qualitative findings that women's beliefs about the importance of calcium supplements to both woman's and baby's health are associated with calcium supplement intake. (32) Positive beliefs about calcium supplements and self-efficacy were associated with taking calcium supplements (OR 4.6, 95% CI: 2.0 to 10.5) and with taking a higher number of supplements (OR 2.77, 95% CI: 1.68 to 4.57). (32)

Calcium supplement characteristics and regimens

Varying preferences about characteristics of calcium tablets

Positive perceptions about the characteristics of the calcium tablet played a role in motivating women to take it. Some women preferred the chewable, sweet-tasting tablets that could be swallowed without water, while others preferred the hard tablets which were smaller in size, had no smell, and needed to be taken with water. Based on individual preference, the taste, smell, size, and convenience affected calcium supplement use (Finding 3.1 – Moderate confidence). (19,40)

Quantitative evidence supported qualitative findings regarding varying preferences calcium supplement's organoleptic properties. One paper which evaluate the impact of a program to implement calcium supplementation showed that most women (77%) reported preferences for conventional tablets that were easier to take and swallow, while the least preferred vehicle was unflavoured powder, as women dislike the taste.(30) Another paper reported that conventional tablets had an acceptable taste (83.9% of women).(31) Chewable tablets were preferred by most women (74%) in another paper. (40) Some characteristics that women considered while taking the supplements include tablet's flavour, chewable or swallow, taken with water or not, smell, and size.(30,31,40)

Supplement regimen as a barrier to use

Women described that they feel overwhelmed with the number of calcium tablets they had to take each day, especially women with other comorbidities who needed to take additional medications for their health conditions. Women felt that 3-4 pills per day at multiple times was onerous and preferred if they were combined into one pill (Finding 3.2 – High confidence). (19,39,40) In two quantitative studies, women preferred taking fewer tablets per

For peer review only - http://bmjopen.bmj.com/site/about/guidelines.xhtml

day. (30,40) However, those allocated to the study arm that used more frequent doses took more calcium overall. (40)

Daily routines and food insecurity

Adherence challenges due to routines

Adherence to calcium supplements was challenging for some women because of conflicting activities in their daily routine, such as taking other medications or supplements, traveling, being away from home, and household chores, which can lead them to forget to take calcium (Finding 4.1 – High confidence). (19,39,40) In quantitative evidence, women described busy work schedules and not being at home as also contributing to forgetting to consume their calcium supplements. (30,31,37) Forgetting to take calcium supplements was the most frequent reason (52.1%) for not taking IFA or calcium supplements and busy work schedules was inversely associated with adherence to calcium supplementation. (31,37)

Food insecurity as a barrier to calcium uptake

Women believed that adequate food was necessary to take the supplements and to avoid nausea. They perceived it as normal to eat before consuming any medication. However, women reported that food insecurity was a critical barrier to calcium uptake (Finding 4.2 – Moderate confidence). (19,39) Quantitative evidence supported qualitative findings that women with food security, high socio-economic status and living in urban areas are more likely to consume calcium supplements as compared to their counterparts.(34,36) Food security was associated with taking 6 more calcium tablets. (34)

Strategies to improve the use of calcium supplements

Implementation of reminders to promote adherence

Women and healthcare providers perceived reminders as beneficial in promoting women's adherence to calcium supplements. Several reminder strategies were described as useful by women and healthcare providers, such as home-based posters, calendars with illustrations and daily reminders, and integrating calcium supplement intake into women's daily routine, such as mealtimes (Finding 5.1 – High confidence). (19,38–40) Quantitative evidence supported the qualitative findings that distribution of behaviour change materials to women, such as pill-taking calendars, were associated with increased adherence. (40)

Importance of family support and 'adherence partner' implementation

Having family support was instrumental to pregnant women adhering to calcium supplements. This could be leveraged by notifying family members on the importance of calcium, and appointing someone to be an "adherence partner" or "pill buddy" to help remind her to take it. Both women and healthcare providers were positive about adherence partners in providing support in terms of encouraging them to take the supplements, providing food, helping them around the house, providing emotional support, improving family relationships,

For peer review only - http://bmjopen.bmj.com/site/about/guidelines.xhtml

and thereby increasing partner or husband involvement in pregnancy (Finding 5.2 – High confidence). (19,38–41) Women could choose who their adherence partner was, and some opted for their husband, a male or female relative, or their child. Some women reported that the support they received from adherence partners decreased over time (19,39–41), suggesting challenges with sustaining appropriate intake throughout pregnancy.

Quantitative evidence supported the qualitative findings that social support is important in encouraging women to take calcium supplements. (28,32,34,35,40) Women considered involving a husband, partner, or family in education sessions or appointing someone as the "adherence partner" to be an acceptable strategy for promoting adherence. (28,32,34,35) Women often chose their husband (52%), older female relative (23%), children (14%), cousins or other relatives as adherence partners (8%) (28), and were satisfied with the reminders and support received from their adherence partner. (28,32,34) However, a randomised trial assessing adherence partners in improving calcium supplement intake showed that high social support, instead of adherence partners alone, was associated with higher adherence to calcium supplements (OR: 2.10; 95% CI: 1.32, 3.34). (28) Women with high family support reported higher intake of calcium supplements (OR = 2.1).(32)

Counselling facilitates calcium supplements uptake

Both women and healthcare providers acknowledged that counselling women on the benefits of calcium was a motivator to calcium supplement intake. Women valued the discussion they have with healthcare providers and felt more confident to take calcium supplements when they received counselling and information and pre-eclampsia and the benefits of calcium from their healthcare providers (Finding 5.3 – Moderate confidence). (19,39) This was confirmed by healthcare providers who reported that they have seen positive results following counselling women on iron-folic acid supplements and believed that this would be replicated for calcium-containing supplements. (39)

Quantitative evidence extended qualitative findings where not only counselling, but also starting antenatal contacts at early gestational age, higher number of antenatal contacts, and receiving free calcium supplements were associated with higher calcium intake by women. (31,32,34,36) One paper reports that women were 59 times more likely to consume calcium supplements if they had received them for free.(32)

Healthcare provider factors

Healthcare provider knowledge and training

Varied knowledge about pre-eclampsia among healthcare providers

Healthcare providers' knowledge about pre-eclampsia was varied. Some felt that pre-eclampsia is not a priority health concern in their area and reported never having encountered any case (Finding 6.1 – Moderate confidence). (19,38,42)

For peer review only

Inadequate training to diagnose and treat pre-eclampsia

While some healthcare providers mentioned that training about pre-eclampsia and calcium supplements was adequate, others reported that their training lacked depth and continuity, and thus felt unprepared to diagnose it and offer information these conditions and the use of calcium for prevention. Healthcare providers expressed the need to have more and continuous training on managing pre-eclampsia, as well as time to address concerns or resistance from the community (Finding 6.2 – High confidence). (19,38,39) Healthcare providers had positive views of trainings and IEC materials and felt that it helps improve their knowledge. (42) They also valued supervision visits which help them solve problems and increases their accountability. (42) Support was also needed throughout calcium roll-out to ensure challenges can be addressed promptly during implementation. (19)

Beliefs about the intervention

Perceived overmedicalization when prescribing calcium supplements

Both healthcare providers and women perceived that prescribing more tablets to "low-risk" women during pregnancy was a form of over-medicalization of pregnancy. However, some healthcare providers felt that calcium supplements were a way to prevent further medicalization due to pre-eclampsia-related complications (Finding 7.1 – Low confidence). (18)

Beliefs about calcium supplements

Healthcare providers generally had positive beliefs about calcium supplements and there was optimism that calcium could be delivered through antenatal care healthcare providers. Some facilitators motivating healthcare providers to prescribe calcium supplements included their beliefs in its prevention value and expected benefits, that women liked the calcium supplements and experienced benefits from it, and a perceived lack of knowledge on how to treat pre-eclampsia which motivated healthcare providers to side towards prevention (Finding 7.2 – High confidence). (18,19,39)

Structural factors

High workload, inadequate staffing, stock out, and lack of equipment

In general, healthcare providers felt that their workload increased by including calcium supplements in antenatal care for pregnant women. Healthcare providers reported existing inadequate staffing, yet they needed to provide additional counselling and prescription to women, especially pregnant women with comorbidities (Finding 8.1 – Moderate confidence). (19,39) Stock-outs were also reported as critical barriers, often due to logistical issues in the supply chain (e.g., centralization or procurement changes), errors in demand estimation by government staff, and inadequate storage facilities. (42) Importantly, some healthcare providers reported that a lack of equipment to diagnose pre-eclampsia was a barrier to

For peer review only - http://bmjopen.bmj.com/site/about/guidelines.xhtml

calcium implementation .(19) Facility staff members and supervisors reported that utilisation of health information systems to monitor calcium stocks, checklists to provide feedback on counselling, and gaps to address, as well as collaboration with government staff members, could be facilitators of use. (42)

Quantitative evidence extended qualitative findings by showing that in the context of a comprehensive integrated program including the implementation of job aids, training, guidelines, monitoring, and feedback session for healthcare providers, could overcome barriers in prescribing women with calcium supplements. (33)

Mapping to behaviour change models

Through COM-B and TDF mapping (Appendix 8), we identified that the critical domains on facilitators and barriers to improve calcium use among women, which include: knowledge, beliefs about consequences, beliefs about capabilities, emotion, social influences, and environmental context and resources to improve calcium use by women. To encourage calcium prescription by healthcare providers, facilitators and barriers related to knowledge, skills, beliefs about consequences and environmental context domains should be addressed. Figure 2 shows the categorisation of barriers and facilitators across the Capability, Opportunity and Motivation Behaviour. The mapping shows that factors encouraging women's use of and adherence to calcium includes receiving adequate information about pre-eclampsia through counselling with healthcare providers and IEC materials, assurance of calcium safety, receiving preferred characteristics of tablet and doses, family and community support, early and frequent antenatal contacts, free calcium supplements, and reminder tools distribution. Likewise, factors that may encourage healthcare providers to prescribe calcium supplements include continuous training about identification and management of pre-eclampsia and calcium supplements, dissemination of consistent messages, reminders, and ensuring adequate number of human resources, equipment for diagnosing pre-eclampsia, and availability of calcium pills at health facilities.

Discussion

We included 18 papers from nine studies conducted primarily in LMICs and reporting views of women, adherence partners, and healthcare providers. Our review shows the importance of healthcare providers' knowledge and training about calcium supplements and pre-eclampsia, as women reported providers as the most reliable sources of information and reassurance on safety of calcium and would encourage adherence. Promoting early initiation of antenatal care and consistent messages on pre-eclampsia and calcium supplementation may improve women's use of calcium supplements. Free calcium supplements and options on doses and tablet preferences could help overcome barriers to calcium supplement use for women. Reminder systems and support from family and community may also help increase women´s calcium uptake.

Women play an important role in decision-making about calcium supplement during pregnancy. Our review shows that women's limited knowledge about and fears of side effects, and potential impacts on their baby serve as the critical barriers to calcium

For peer review only - http://bmjopen.bmj.com/site/about/guidelines.xhtml

supplement intake. These barriers about calcium supplementation have been reported on the use of other supplements during pregnancy. For example, studies on factors affecting multiple micronutrient supplementation, iron folic acid, and lipid-based nutrients reported women's knowledge, acceptance, motivation, and attitudes toward the medication play an important role. (43,44) Furthermore, a study on factors affecting use of intervention for preterm management also reported women felt hesitant in consuming the medications to improve labour outcome due to fears about baby's growth and development.(45) This highlights the need to ensure that women are aware on the benefits of the supplement and given assurance on the safety of its use.

As the most trusted informants, healthcare providers knowledge about calcium and pre-eclampsia are important to support women uptake of calcium supplements. Studies reported different levels of knowledge about calcium and pre-eclampsia among providers, with some providers having persistent beliefs that evidence on calcium supplements during pregnancy is still in the experimental phase. Some healthcare providers might be reluctant to talk about pre-eclampsia with the fear to stress low risk women; however, this reluctance should be balanced by the risk of symptoms of pre-eclampsia going unnoticed by women. Reinforcing messages related to improving pregnancy outcomes and similarities to other supplements taken during pregnancy such as folic acid and iron that also help to may facilitate use of calcium supplements. Healthcare providers also need to know about how to assess eligibility for calcium supplements, including how to screen and score women at high-risk and to identify populations with low calcium intake. There is lack of acceptable biomarkers of individual calcium intake and calcium status, which complicates screening individuals.(46) WHO recommendations on calcium supplementation were set for populations with low calcium intake, as dietary assessments are more reliable to identify populations with low calcium intake rather than to identify individuals. (46)

Human resource shortages are a recurrent health system challenge, particularly in LMICs which results in overburdened staff unable to deliver recommended practices. Appropriate staffing, particularly of midwives and nurses who provide most antenatal care services, remains crucial to achieve quality of care. Unfortunately, this is also applicable on the context of other medication delivered during pregnancy. For example, providers reported unavailability of stock, inadequate staffs and equipment as the main barriers in prescribing interventions to pregnant women experiencing preterm labour. Therefore, ensuring availability of diagnostic tools and calcium stocks is critical to ensure appropriate prescription and delivery of care.(45) Where health providers constraints persist, innovative strategies to streamline antenatal care practices may be needed to improve efficiency.(47)

Implications for research, policy, and practice

The TDF and COM-B mapping in our review can be used by researchers and programme managers to inform the development of implementation models to optimise the use of calcium supplements. Assessing the extent to which the barriers and facilitators to calcium prescription and use identified in our review are potential implementation challenges in different contexts can be a useful starting point for formative research to scale-up implementation. Table 2 presents a list of questions derived from our findings and may help programme implementers, policymakers, researchers, and other stakeholders to identify

For peer review only - http://bmjopen.bmj.com/site/about/guidelines.xhtml

and address factors that may affect prescription and use of calcium supplements during pregnancy. Assessing the extent to which the barriers and facilitators identified in our review are potential implementation challenges in different settings is a useful starting point for formative research to scale this intervention.

Table 2. Implications for research, policy, and practice

Domain	List of questions
**Guidelines and protocols**	1. Are guidelines and clinical protocols on pre-eclampsia/eclampsia and calcium supplements during pregnancy consistent between WHO, national, and facility-levels?
**Knowledge and learning**	2. Do women or healthcare providers have scepticism or concerns about adverse effects of calcium supplements during pregnancy that can be addressed?<br>3. Do women and their family members receive education and educational materials about signs of pre-eclampsia/eclampsia early in pregnancy?<br>4. Do women have sufficient time and opportunity to discuss pre-eclampsia/eclampsia with healthcare providers during antenatal care?<br>5. Do women have sufficient time and opportunity to discuss calcium supplementation with healthcare providers during antenatal care, including addressing fears about side effects, managing side effects, safety concerns, and reinforcing positive messaging about expected benefits?<br>6. Have concerns from both women and healthcare providers about calcium supplements as a form of overmedicalisation of pregnancy been addressed in culturally-appropriate ways?<br>7. Have healthcare providers received in-service training on pre-eclampsia/eclampsia prevention and management, including the importance of calcium supplements during pregnancy for prevention?
**Strategies to improve use**	8. Do all relevant cadres of healthcare providers (including midwives and nurses) have authority to prescribe calcium supplements during pregnancy?<br>9. Do women have the opportunity to try different types of calcium tablets to suit their preferences, such as chewable/non-chewable, different tastes, and different size tablets?<br>10. Do women have the opportunity to try different calcium dosing combinations to suit their schedules and preferences?<br>11. For women experiencing or at risk of food insecurity during pregnancy, are there additional social services to support adequate nutrition intake during pregnancy?<br>12. Have different types of reminder systems (e.g., posters for home, calendars, and integration into daily routines) been designed with women and their families to encourage use?<br>13. Has support from family and/or adherence partners been integrated for women, and do family members or adherence partners have the opportunity to attend educational sessions?<br>14. Are stocks of calcium readily available in the antenatal care wards?<br>15. Is there sufficient funding and budget allocation to ensure continuous procurement and distribution of calcium supplements?

Strengths and limitations

Most included studies were from low- and middle-income countries and almost half came from the same project conducted in Kenya and Ethiopia, which may limit transferability of results to other contexts. The results from our review therefore can be used to guide formative research as well as implementation and programme planning in other contexts.

For peer review only - http://bmjopen.bmj.com/site/about/guidelines.xhtml

Most included studies engaged women who attended antenatal care; therefore, might not be representative of those that do not reach the health system during pregnancy. None of the included studies reported the perspectives of policymakers.

Despite these limitations, this is the first systematic review of barriers and facilitators to calcium supplement use during pregnancy. We adopted a mixed methods approach which allowed for inclusion and consolidation of studies with a range of designs. Mapping the review findings to behaviour change models improved understanding of how barriers and facilitators influence calcium uptake, and consequently can be addressed in future interventional or programmatic work.

Conclusion

Our review identified a range of barriers and facilitators affecting calcium supplements during pregnancy to prevent pre-eclampsia. When formulating intervention and policies on calcium supplement use, relevant stakeholders should consider the identified barriers and facilitators to optimise uptake. Findings from this study can inform implementation considerations, to ensure effective and equitable provision and scale-up of calcium interventions.

Figure 1: PRISMA Flow Diagram

Figure 2: Categorisation of barriers and facilitators across the Capability, Opportunity and Motivation Behaviour

Funding: This work was funded by the Medical Research Council [grant number MR/T038861/1] and the UNDP-UNFPA-UNICEF-WHO-World Bank Special Programme of Research, Development and Research Training in Human Reproduction (HRP), a cosponsored programme executed by the World Health Organization (WHO). MAB's time is supported by an Australian Research Council Discovery Early Career Researcher Award (DE200100264) and a Dame Kate Campbell Fellowship (University of Melbourne Faculty of Medicine, Dentistry and Health Sciences).

Competing interests: None declared

Ethics: Ethical approval was not required; no primary data were collected.

Patient consent for publication: Not required.

Contributor ship Statement: All authors have made substantial contributions to the following: Conceptualisation: ST, APB, MAB, JA, GC Data curation: TR, JA, GC, HM, RIZ Formal analysis: RIZ, GC, HM, MAB, Funding acquisition: ST, APB, JA, Investigation and methodology: RIZ, MAB, GC, HM Writing – original draft: GC, HM, RIZ, MAB, Writing – review and editing: GC, HM, RIZ, JA, TR, JPP, ZQ, GJH, HM, LS, JPV, AP, GNG, EA, KKL, GC, RR, KS, TA, TY, APB, TS and MAB.

Data Sharing Statement: All data relevant to the study are included in the article or uploaded as supplementary information.

For peer review only

For peer review only

For peer review only - http://bmjopen.bmj.com/site/about/guidelines.xhtml

References

1. Poon LC, Shennan A, Hyett JA, Kapur A, Hadar E, Divakar H, et al. The International Federation of Gynecology and Obstetrics (FIGO) initiative on pre-eclampsia: A pragmatic guide for first-trimester screening and prevention. Int J Gynecol Obstet. 2019;145(S1).

2. Mol BWJ, Roberts CT, Thangaratinam S, Magee LA, De Groot CJM, Hofmeyr GJ. Pre-eclampsia. In: The Lancet. 2016. p. 999–1011.

3. Gestational Hypertension and Preeclampsia: ACOG Practice Bulletin, Number 222. Obstet Gynecol. 2020;135(6).

4. Say L, Chou D, Gemmill A, Tunçalp Ö, Moller A-B, Daniels J, et al. Global causes of maternal death: a WHO systematic analysis. Lancet Glob Heal [Internet]. 2014 Jun;2(6):e323–33. Available from: https://linkinghub.elsevier.com/retrieve/pii/S2214109X1470227X

5. Steegers EAP, von Dadelszen P, Duvekot JJ, Pijnenborg R. Pre-eclampsia. Lancet [Internet]. 2010 Aug;376(9741):631–44. Available from: https://linkinghub.elsevier.com/retrieve/pii/S0140673610602796

6. Prevention and treatment of pre-eclampsia and eclampsia. World Health. 2011.

7. Hofmeyr GJ, Lawrie TA, Atallah ÁN, Torloni MR. Calcium supplementation during pregnancy for preventing hypertensive disorders and related problems. Vol. 2018, Cochrane Database of Systematic Reviews. 2018.

8. WHO recommendation: Calcium supplementation during pregnancy for the prevention of pre-eclampsia and its complications. Geneva; 2018.

9. WHO recommendation on Calcium supplementation before pregnancy for the prevention of pre-eclampsia and its complications. Geneva; 2020.

10. WHO Recommendations on Antenatal Care for a Positive Pregnancy Experience. Geneva; 2013.

11. Omotayo MO, Dickin KL, O'Brien KO, Neufeld LM, De Regil LM, Stoltzfus RJ. Calcium Supplementation to Prevent Preeclampsia: Translating Guidelines into Practice in Low-Income Countries. Adv Nutr. 2016;7(2):275–8.

12. Page MJ, McKenzie JE, Bossuyt PM, Boutron I, Hoffmann TC, Mulrow CD, et al. The PRISMA 2020 statement: An updated guideline for reporting systematic reviews. Vol. 372, The BMJ. 2021.

13. Tong A, Flemming K, McInnes E, Oliver S, Craig J. Enhancing transparency in reporting the synthesis of qualitative research: ENTREQ. BMC Med Res Methodol. 2012;12.

14. Glenton C, Lewin S, Downe S, Paulsen E, Munabi-Babigumira S, Johansen M, et al. Qualitative Evidence Syntheses Within Cochrane Effective Practice and Organisation of Care: Developing a Template and Guidance. Int J Qual Methods. 2021;20.

For peer review only - http://bmjopen.bmj.com/site/about/guidelines.xhtml

15. Covidence systematic review software. Melbourne, Australia: Veritas Health Innovation;

16. Hong QN, Fàbregues S, Bartlett G, Boardman F, Cargo M, Dagenais P, et al. The Mixed Methods Appraisal Tool (MMAT) version 2018 for information professionals and researchers. Educ Inf. 2018;34(4).

17. Thomas J, Harden A. Methods for the thematic synthesis of qualitative research in systematic reviews. BMC Med Res Methodol [Internet]. 2008 Dec 10;8(1):45. Available from: https://bmcmedresmethodol.biomedcentral.com/articles/10.1186/1471-2288-8-45

18. Vestering A, Bekker MN, Grobbee DE, van der Graaf R, Franx A, Crombag NMT, et al. Views and preferences of medical professionals and pregnant women about a novel primary prevention intervention for hypertensive disorders of pregnancy: a qualitative study. Reprod Health [Internet]. 2019 May 2;16(1):46. Available from: http://www.ncbi.nlm.nih.gov/pubmed/31046778

19. Birhanu Z, Chapleau GM, Ortolano SE, Mamo G, Martin SL, Dickin KL. Ethiopian women's perspectives on antenatal care and iron-folic acid supplementation: Insights for translating global antenatal calcium guidelines into practice. Matern Child Nutr [Internet]. 2018 Feb;14:e12424. Available from: https://onlinelibrary.wiley.com/doi/10.1111/mcn.12424

20. Michie S, van Stralen MM, West R. The behaviour change wheel: A new method for characterising and designing behaviour change interventions. Implement Sci [Internet]. 2011 Dec 23;6(1):42. Available from: http://implementationscience.biomedcentral.com/articles/10.1186/1748-5908-6-42

21. Atkins L, Francis J, Islam R, O'Connor D, Patey A, Ivers N, et al. A guide to using the Theoretical Domains Framework of behaviour change to investigate implementation problems. Implement Sci [Internet]. 2017 Dec 21;12(1):77. Available from: http://implementationscience.biomedcentral.com/articles/10.1186/s13012-017-0605-9

22. Lewin S, Bohren M, Rashidian A, Munthe-Kaas H, Glenton C, Colvin CJ, et al. Applying GRADE-CERQual to qualitative evidence synthesis findings—paper 2: how to make an overall CERQual assessment of confidence and create a Summary of Qualitative Findings table. Implement Sci [Internet]. 2018 Jan 25;13(S1):10. Available from: https://implementationscience.biomedcentral.com/articles/10.1186/s13012-017-0689-2

23. Munthe-Kaas H, Bohren MA, Glenton C, Lewin S, Noyes J, Tunçalp Ö, et al. Applying GRADE-CERQual to qualitative evidence synthesis findings—paper 3: how to assess methodological limitations. Implement Sci [Internet]. 2018 Jan 25;13(S1):9. Available from: https://implementationscience.biomedcentral.com/articles/10.1186/s13012-017-0690-9

24. Colvin CJ, Garside R, Wainwright M, Munthe-Kaas H, Glenton C, Bohren MA, et al. Applying GRADE-CERQual to qualitative evidence synthesis findings—paper 4: how to assess coherence. Implement Sci [Internet]. 2018 Jan 25;13(S1):13. Available from: https://implementationscience.biomedcentral.com/articles/10.1186/s13012-017-0691-8

For peer review only

For peer review only - http://bmjopen.bmj.com/site/about/guidelines.xhtml

25. Glenton C, Carlsen B, Lewin S, Munthe-Kaas H, Colvin CJ, Tunçalp Ö, et al. Applying GRADE-CERQual to qualitative evidence synthesis findings—paper 5: how to assess adequacy of data. Implement Sci [Internet]. 2018 Jan 25;13(S1):14. Available from: https://implementationscience.biomedcentral.com/articles/10.1186/s13012-017-0692-7

26. Noyes J, Booth A, Lewin S, Carlsen B, Glenton C, Colvin CJ, et al. Applying GRADE-CERQual to qualitative evidence synthesis findings–paper 6: how to assess relevance of the data. Implement Sci [Internet]. 2018 Jan 25;13(S1):4. Available from: https://implementationscience.biomedcentral.com/articles/10.1186/s13012-017-0693-6

27. Bora K, Barman B, Pala S, Das A, Doke G, Tripura A. Coverage of antenatal iron-folic acid and calcium distribution during pregnancy and their contextual determinants in the northeastern region of India. Front Nutr [Internet]. 2022 Jul 18;9. Available from: https://www.frontiersin.org/articles/10.3389/fnut.2022.894245/full

28. Martin SL, Omotayo MO, Pelto GH, Chapleau GM, Stoltzfus RJ, Dickin KL. Adherence-specific social support enhances adherence to calcium supplementation regimens among pregnant women. J Nutr. 2017;147(4).

29. Ghosh-Jerath S, Devasenapathy N, Singh A, Shankar A, Zodpey S. Ante natal care (ANC) utilization, dietary practices and nutritional outcomes in pregnant and recently delivered women in urban slums of Delhi, India: an exploratory cross-sectional study. Reprod Health [Internet]. 2015 Dec 20;12(1):20. Available from: https://reproductive-health-journal.biomedcentral.com/articles/10.1186/s12978-015-0008-9

30. Baxter J-AB, Roth DE, Al Mahmud A, Ahmed T, Islam M, Zlotkin SH. Tablets Are Preferred and More Acceptable Than Powdered Prenatal Calcium Supplements among Pregnant Women in Dhaka, Bangladesh. J Nutr [Internet]. 2014 Jul 1;144(7):1106–12. Available from: https://academic.oup.com/jn/article/144/7/1106/4569766

31. Thapa K, Sanghvi H, Rawlins B, Karki YB, Regmi K, Aryal S, et al. Coverage, compliance, acceptability and feasibility of a program to prevent pre-eclampsia and eclampsia through calcium supplementation for pregnant women: An operations research study in one district of Nepal. BMC Pregnancy Childbirth. 2016;

32. Nguyen PH, Kachwaha S, Avula R, Young M, Tran LM, Ghosh S, et al. Maternal nutrition practices in Uttar Pradesh, India: Role of key influential demand and supply factors. Matern Child Nutr [Internet]. 2019 Oct 18;15(4). Available from: https://onlinelibrary.wiley.com/doi/10.1111/mcn.12839

33. Omotayo MO, Dickin KL, Pelletier DL, Martin SL, Kung'u JK, Stoltzfus RJ. Feasibility of integrating calcium and iron-folate supplementation to prevent preeclampsia and anemia in pregnancy in primary healthcare facilities in Kenya. Matern Child Nutr [Internet]. 2018 Feb;14:e12437. Available from: https://onlinelibrary.wiley.com/doi/10.1111/mcn.12437

34. Nguyen PH, Sanghvi T, Kim SS, Tran LM, Afsana K, Mahmud Z, et al. Factors influencing

For peer review only - http://bmjopen.bmj.com/site/about/guidelines.xhtml

maternal nutrition practices in a large scale maternal, newborn and child health program in Bangladesh. de Souza RJ, editor. PLoS One [Internet]. 2017 Jul 10;12(7):e0179873. Available from: https://dx.plos.org/10.1371/journal.pone.0179873

35. Nguyen PH, Frongillo EA, Sanghvi T, Wable G, Mahmud Z, Tran LM, et al. Engagement of Husbands in a Maternal Nutrition Program Substantially Contributed to Greater Intake of Micronutrient Supplements and Dietary Diversity during Pregnancy: Results of a Cluster-Randomized Program Evaluation in Bangladesh. J Nutr [Internet]. 2018 Aug 1;148(8):1352–63. Available from: https://academic.oup.com/jn/article/148/8/1352/5040613

36. Liu D, Cheng Y, Dang S, Wang D, Zhao Y, Li C, et al. Maternal adherence to micronutrient supplementation before and during pregnancy in Northwest China: a large-scale population-based cross-sectional survey. BMJ Open [Internet]. 2019 Aug 8;9(8):e028843. Available from: https://bmjopen.bmj.com/lookup/doi/10.1136/bmjopen-2018-028843

37. Shakya Shrestha S, Adhikari R, Tamrakar S, Shrestha R, Shrestha A. Adherence to Iron, Folic Acid and Calcium Supplement and Factors Affecting it among the Antenatal Care Attending Women in a Tertiary Care Hospital: A Cross Sectional Study. Kathmandu Univ Med J (KUMJ) [Internet]. 18(70):186–92. Available from: http://www.ncbi.nlm.nih.gov/pubmed/33594028

38. Martin SL, Seim GL, Wawire S, Chapleau GM, Young SL, Dickin KL. Translating formative research findings into a behaviour change strategy to promote antenatal calcium and iron and folic acid supplementation in western Kenya. Matern Child Nutr [Internet]. 2017 Jan 22;13(1):mcn.12233. Available from: https://onlinelibrary.wiley.com/doi/10.1111/mcn.12233

39. Martin SL, Wawire V, Ombunda H, Li T, Sklar K, Tzehaie H, et al. Integrating Calcium Supplementation into Facility-Based Antenatal Care Services in Western Kenya: A Qualitative Process Evaluation to Identify Implementation Barriers and Facilitators. Curr Dev Nutr [Internet]. 2018 Nov 1;2(11). Available from: https://academic.oup.com/cdn/article/doi/10.1093/cdn/nzy068/5078341

40. Omotayo MO, Martin SL, Stoltzfus RJ, Ortolano SE, Mwanga E, Dickin KL. With adaptation, the WHO guidelines on calcium supplementation for prevention of pre-eclampsia are adopted by pregnant women. Matern Child Nutr [Internet]. 2018 Apr;14(2):e12521. Available from: http://doi.wiley.com/10.1111/mcn.12521

41. Martin SL, Omotayo MO, Chapleau GM, Stoltzfus RJ, Birhanu Z, Ortolano SE, et al. Adherence partners are an acceptable behaviour change strategy to support calcium and iron-folic acid supplementation among pregnant women in Ethiopia and Kenya. Matern Child Nutr [Internet]. 2017 Jul;13(3):e12331. Available from: https://onlinelibrary.wiley.com/doi/10.1111/mcn.12331

42. Kachwaha S, Nguyen PH, Tran LM, Avula R, Young MF, Ghosh S, et al. Specificity Matters: Unpacking Impact Pathways of Individual Interventions within Bundled Packages Helps Interpret the Limited Impacts of a Maternal Nutrition Intervention in India. J Nutr. 2022

For peer review only

Feb;152(2):612–29.

43. Clermont A, Kodish S, Matar Seck A, Salifou A, Rosen J, Grais R, et al. Acceptability and Utilization of Three Nutritional Supplements during Pregnancy: Findings from a Longitudinal, Mixed-Methods Study in Niger. Nutrients [Internet]. 2018 Aug 12;10(8):1073. Available from: http://www.mdpi.com/2072-6643/10/8/1073

44. Garcia-Casal MN, Estevez D, De-Regil LM. Multiple micronutrient supplements in pregnancy: Implementation considerations for integration as part of quality services in routine antenatal care. Objectives, results, and conclusions of the meeting. Matern Child Nutr [Internet]. 2018 Dec;14:e12704. Available from: https://onlinelibrary.wiley.com/doi/10.1111/mcn.12704

45. Zahroh RI, Hazfiarini A, Eddy KE, Vogel JP, Tunçalp Özge, Minckas N, et al. Factors influencing appropriate use of interventions for management of women experiencing preterm birth: A mixed-methods systematic review and narrative synthesis. Stock SJ, editor. PLOS Med [Internet]. 2022 Aug 23;19(8):e1004074. Available from: https://dx.plos.org/10.1371/journal.pmed.1004074

46. (EFSA) EFSA. Scientific Opinion on Dietary Reference Values for calcium. EFSA J. 2015;13(5):1–82.

47. Betrán AP, Bergel E, Griffin S, Melo A, Nguyen MH, Carbonell A, et al. Provision of medical supply kits to improve quality of antenatal care in Mozambique: a stepped-wedge cluster randomised trial. Lancet Glob Heal [Internet]. 2018 Jan;6(1):e57–65. Available from: https://linkinghub.elsevier.com/retrieve/pii/S2214109X17304217

For peer review only

For peer review only

For peer review only - http://bmjopen.bmj.com/site/about/guidelines.xhtml

For peer review only

PRISMA 2020 flow diagram for new systematic reviews which included searches of databases, registers, and other sources

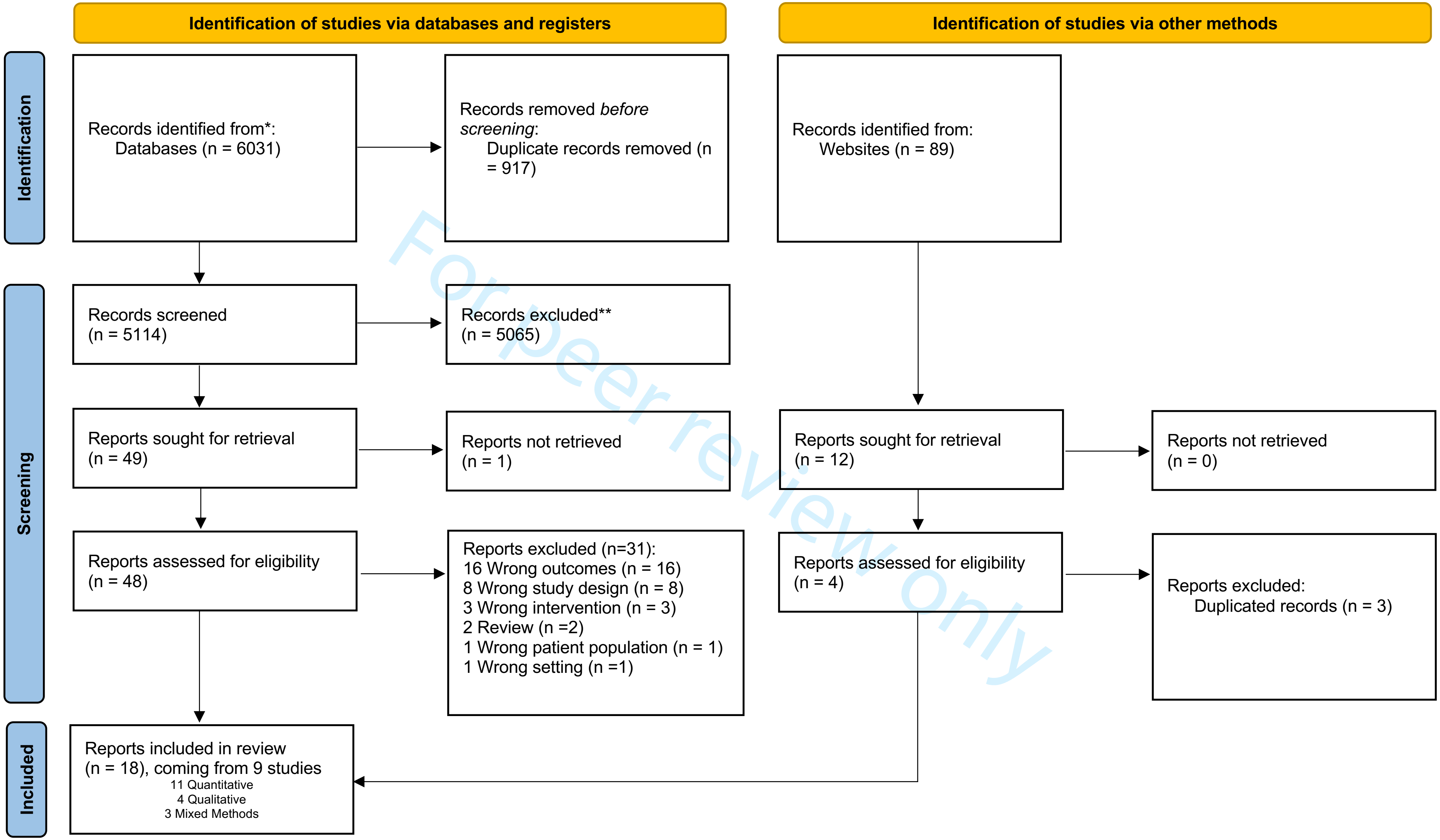

*Consider, if feasible to do so, reporting the number of records identified from each database or register searched (rather than the total number across all databases/registers).
**If automation tools were used, indicate how many records were excluded by a human and how many were excluded by automation tools.

From: Page MJ, McKenzie JE, Bossuyt PM, Boutron I, Hoffmann TC, Mulrow CD, et al. The PRISMA 2020 statement: an updated guideline for reporting systematic reviews. BMJ 2021;372:n71. doi: 10.1136/bmj.n71. For more information, visit: http://www.prisma-statement.org/

For peer review only

Capability in using interventions

Barriers

- Limited knowledge about preeclampsia/eclampsia†
- Symptoms were believed to be linked to evil attacks or nutritional deficiencies†
- Inadequate information on preeclampsia/eclampsia and calcium from providers†
- Varied knowledge on pre-eclampsia/eclampsia ¥
- In continuity and lack of depth training about pre-eclampsia/eclampsia ¥
- Felt inadequately trained ¥

Facilitators

- High knowledge of calcium supplementation†
- High education of women†
- Receive adequate information about condition and calcium from their providers†
- Counselling on information about preeclampsia and calcium from their providers†
- Continuous training to manage the condition and address resistance from community ¥
- Regular supervision visits for troubleshooting ¥

Motivation in using interventions

Barriers

- Pre-eclampsia/eclampsia was not considered as a serious problem†
- Experiences and fears of side effects†
- Safety of calcium was not assured†
- Felt that 3-4 pills per day at multiple times were too many†
- Inconvenience in taking pill daily†
- Perceived over-medicalization†¥
- Belief that preeclampsia/eclampsia was not a priority health concern ¥
- Fears in generating anxiety on low-risk women ¥

Facilitators

- Assurance of calcium safety†
- Experienced and expected benefits from taking calcium and folic acid†
- Valued information, education and communication (IEC) materials†
- Positive perceptions on tablet characteristics (smell, size, taste) †
- Belief that one combined pill per day could ease burden†
- Belief that having information would help making informed decisions†
- Belief that providers is a reliable source of information†
- Belief of being able to consume calcium daily†
- Feeling confident taking calcium after counselling provider†
- Positive belief about calcium supplementation benefits¥
- Belief that women should receive information regardless risk ¥

Opportunity in using interventions

Barriers

- Discouragement by family, neighbors and community in taking calcium†
- Stigmatization of having supplements and posters with HIV†
- Conflicting daily routine with taking calcium†
- Food insecurity†
- Providers felt that providing calcium would increase workload ¥
- Inadequate number of staff providing care ¥
- Stock out of supplements ¥
- Lack of equipment to diagnose ¥

Facilitators

- Family support in consuming calcium†
- Positive belief and experiences about 'adherence partners' †¥
- Early initiation and frequent antenatal visits †
- Universal free calcium distribution through antenatal care †
- Reminder tools distribution, such posters and pill-taking calendars †¥
- Adequate calcium supplement stock and its storage ¥
- Provision of equipment to diagnose pre-eclampsia ¥
- Adequate number of human resources at health facility ¥
- Comprehensive integrated program (job aids, training, guidelines, regular supplies) ¥

Behaviour: Calcium supplementation prescription by providers and use by women

†: woman's factors; ¥: providers factors

For peer review only - http://bmjopen.bmj.com/site/about/guidelines.xhtml

Appendix 1. PRISMA 2020 Main Checklist

Topic	No.	Item	Location where item is reported
**TITLE**			
**Title**	1	Identify the report as a systematic review.	Title
**ABSTRACT**			
**Abstract**	2	See the PRISMA 2020 for Abstracts checklist	Abstract
**INTRODUCTION**			
**Rationale**	3	Describe the rationale for the review in the context of existing knowledge.	Introduction
**Objectives**	4	Provide an explicit statement of the objective(s) or question(s) the review addresses.	Introduction
**METHODS**			
**Eligibility criteria**	5	Specify the inclusion and exclusion criteria for the review and how studies were grouped for the syntheses.	Methods – Type of studies, Topic of interest
**Information sources**	6	Specify all databases, registers, websites, organisations, reference lists and other sources searched or consulted to identify studies. Specify the date when each source was last searched or consulted.	Methods – Search methods for identification of studies
**Search strategy**	7	Present the full search strategies for all databases, registers and websites, including any filters and limits used.	Methods – Appendix 1
**Selection process**	8	Specify the methods used to decide whether a study met the inclusion criteria of the review, including how many reviewers screened each record and each report retrieved, whether they	Methods – Selection of studies

For peer review only - http://bmjopen.bmj.com/site/about/guidelines.xhtml

		worked independently, and if applicable, details of automation tools used in the process.	
**Data collection process**	9	Specify the methods used to collect data from reports, including how many reviewers collected data from each report, whether they worked independently, any processes for obtaining or confirming data from study investigators, and if applicable, details of automation tools used in the process.	Methods – Data extraction and assessing methodological limitations
**Data items**	10a	List and define all outcomes for which data were sought. Specify whether all results that were compatible with each outcome domain in each study were sought (e.g. for all measures, time points, analyses), and if not, the methods used to decide which results to collect.	Methods – Data extraction and assessing methodological limitations
	10b	List and define all other variables for which data were sought (e.g. participant and intervention characteristics, funding sources). Describe any assumptions made about any missing or unclear information.	Methods – Data extraction and assessing methodological limitations
**Study risk of bias assessment**	11	Specify the methods used to assess risk of bias in the included studies, including details of the tool(s) used, how many reviewers assessed each study and whether they worked independently, and if applicable, details of automation tools used in the process.	Methods – Data extraction and assessing methodological limitations
**Effect measures**	12	Specify for each outcome the effect measure(s) (e.g. risk ratio, mean difference) used in the synthesis or presentation of results.	Not applicable
**Synthesis methods**	13a	Describe the processes used to decide which studies were eligible for each synthesis (e.g. tabulating the study intervention characteristics and comparing against the planned groups for each synthesis (item 5)).	Not applicable
	13b	Describe any methods required to prepare the data for presentation or synthesis, such as handling of missing summary statistics, or data conversions.	Not applicable
	13c	Describe any methods used to tabulate or visually display results of individual studies and syntheses.	Methods – Data management, analysis, and synthesis

For peer review only - http://bmjopen.bmj.com/site/about/guidelines.xhtml

	13d	Describe any methods used to synthesize results and provide a rationale for the choice(s). If meta-analysis was performed, describe the model(s), method(s) to identify the presence and extent of statistical heterogeneity, and software package(s) used.	Methods – Data management, analysis, and synthesis
	13e	Describe any methods used to explore possible causes of heterogeneity among study results (e.g. subgroup analysis, meta-regression).	Not Applicable
	13f	Describe any sensitivity analyses conducted to assess robustness of the synthesized results.	Methods – Data management, analysis, and synthesis
**Reporting bias assessment**	14	Describe any methods used to assess risk of bias due to missing results in a synthesis (arising from reporting biases).	Methods – Data management, analysis, and synthesis
**Certainty assessment**	15	Describe any methods used to assess certainty (or confidence) in the body of evidence for an outcome.	Methods – Data management, analysis, and synthesis
**RESULTS**			
**Study selection**	16a	Describe the results of the search and selection process, from the number of records identified in the search to the number of studies included in the review, ideally using a flow diagram.	Results, page 7 and Figure 1. PRISMA flowchart
	16b	Cite studies that might appear to meet the inclusion criteria, but which were excluded, and explain why they were excluded.	Figure 2. PRISMA flowchart
**Study characteristics**	17	Cite each included study and present its characteristics.	Results, page 7 Table 1
**Risk of bias in studies**	18	Present assessments of risk of bias for each included study.	Results
**Results of individual studies**	19	For all outcomes, present, for each study: (a) summary statistics for each group (where appropriate) and (b) an effect estimate and its precision (e.g. confidence/credible interval), ideally using structured tables or plots.	Appendix 5

For peer review only - http://bmjopen.bmj.com/site/about/guidelines.xhtml

**Results of syntheses**	20a	For each synthesis, briefly summarise the characteristics and risk of bias among contributing studies.	Results page 10 – Table 2. Summary of qualitative findings and GRADE-CERQual Evidence Profile
	20b	Present results of all statistical syntheses conducted. If meta-analysis was done, present for each the summary estimate and its precision (e.g. confidence/credible interval) and measures of statistical heterogeneity. If comparing groups, describe the direction of the effect.	Not applicable
	20c	Present results of all investigations of possible causes of heterogeneity among study results.	Not applicable
	20d	Present results of all sensitivity analyses conducted to assess the robustness of the synthesized results.	Not applicable
**Reporting biases**	21	Present assessments of risk of bias due to missing results (arising from reporting biases) for each synthesis assessed.	Not applicable
**Certainty of evidence**	22	Present assessments of certainty (or confidence) in the body of evidence for each outcome assessed.	Results Page 10-18– Table 2. Summary of qualitative findings and GRADE-CERQual Evidence Profile
**DISCUSSION**			
**Discussion**	23a	Provide a general interpretation of the results in the context of other evidence.	Discussions, Interpretation
	23b	Discuss any limitations of the evidence included in the review.	Discussions, Strengths and limitations
	23c	Discuss any limitations of the review processes used.	Discussions, Strengths and limitations
	23d	Discuss implications of the results for practice, policy, and future research.	Discussions, Implications for practice and conclusions
**OTHER INFORMATION**			

For peer review only - http://bmjopen.bmj.com/site/about/guidelines.xhtml

**Registration and protocol**	24a	Provide registration information for the review, including register name and registration number, or state that the review was not registered.	Methods
	24b	Indicate where the review protocol can be accessed, or state that a protocol was not prepared.	Methods
	24c	Describe and explain any amendments to information provided at registration or in the protocol.	Methods
**Support**	25	Describe sources of financial or non-financial support for the review, and the role of the funders or sponsors in the review.	Sources of funding
**Competing interests**	26	Declare any competing interests of review authors.	Competing interests
**Availability of data, code and other materials**	27	Report which of the following are publicly available and where they can be found: template data collection forms; data extracted from included studies; data used for all analyses; analytic code; any other materials used in the review.	Development of themes can be found on the Appendix 6, 7,8

For peer review only

Appendix 1.1. PRISMA Abstract Checklist

Topic	No.	Item	Reported?
**TITLE**			
**Title**	1	Identify the report as a systematic review.	Yes
**INTRODUCTION**			
**Objectives**	2	Provide an explicit statement of the main objective(s) or question(s) the review addresses.	Yes
**METHODS**			
**Eligibility criteria**	3	Specify the inclusion and exclusion criteria for the review.	Yes
**Information sources**	4	Specify the information sources (e.g. databases, registers) used to identify studies and the date when each was last searched.	Yes
**Risk of bias**	5	Specify the methods used to assess risk of bias in the included studies.	Yes
**Synthesis of results**	6	Specify the methods used to present and synthesize results.	Yes
**RESULTS**			
**Included studies**	7	Give the total number of included studies and participants and summarise relevant characteristics of studies.	Yes
**Synthesis of results**	8	Present results for main outcomes, preferably indicating the number of included studies and participants for each. If meta-analysis was done, report the summary estimate and confidence/credible interval. If comparing groups, indicate the direction of the effect (i.e. which group is favoured).	Yes
**DISCUSSION**			

For peer review only - http://bmjopen.bmj.com/site/about/guidelines.xhtml

**Limitations of evidence**	9	Provide a brief summary of the limitations of the evidence included in the review (e.g. study risk of bias, inconsistency and imprecision).	Yes
**Interpretation**	10	Provide a general interpretation of the results and important implications.	Yes
**OTHER**			
**Funding**	11	Specify the primary source of funding for the review.	Yes
**Registration**	12	Provide the register name and registration number.	Yes

From: Page MJ, McKenzie JE, Bossuyt PM, Boutron I, Hoffmann TC, Mulrow CD, et al. The PRISMA 2020 statement: an updated guideline for reporting systematic reviews. MetaArXiv. 2020, September 14. DOI: 10.31222/osf.io/v7gm2. For more information, visit: www.prisma-statement.org

For peer review only

For peer review only - http://bmjopen.bmj.com/site/about/guidelines.xhtml

Appendix 2. Enhancing transparency in reporting the synthesis of qualitative research: ENTREQ Checklist (Tong, et al., 2012)

Item No.	Guide and Description	Report Location
1. Aim	State the research question the synthesis addresses	Introduction
2. Synthesis methodology	Identify the synthesis methodology or theoretical framework which underpins the synthesis, and describe the rationale for choice of methodology (e.g. meta-ethnography, thematic synthesis, critical interpretive synthesis, grounded theory synthesis, realist synthesis, meta-aggregation, meta-study, framework synthesis)	Methods – _Data management, analysis, and synthesis
3. Approach to searching	Indicate whether the search was pre-planned (comprehensive search strategies to seek all available studies) or iterative (to seek all available concepts until they theoretical saturation is achieved)	Methods – Search methods for identification of studies
4. Inclusion criteria	Specify the inclusion/exclusion criteria (e.g. in terms of population, language, year limits, type of publication, study type)	Methods – Selection of studies
5. Data sources	Describe the information sources used (e.g. electronic databases (MEDLINE, EMBASE, CINAHL, psycINFO), grey literature databases (digital thesis, policy reports), relevant organisational websites, experts, information specialists, generic web searches (Google Scholar) hand searching, reference lists) and when the searches conducted; provide the rationale for using the data sources	Methods - Search methods for identification of studies
6. Electronic Search strategy	Describe the literature search (e.g. provide electronic search strategies with population terms, clinical or health topic terms, experiential or social phenomena related terms, filters for qualitative research, and search limits)	Appendix 5
7. Study screening methods	Describe the process of study screening and sifting (e.g. title, abstract and full text review, number of independent reviewers who screened studies)	Methods - Search methods for identification of studies
8. Study characteristics	Present the characteristics of the included studies (e.g. year of publication, country, population, number of participants, data collection, methodology, analysis, research questions)	Table 1 - Characteristics of included studies
9. Study selection results	Identify the number of studies screened and provide reasons for study exclusion (e.g. for comprehensive searching, provide numbers of studies screened and reasons for exclusion indicated in a figure/flowchart; for iterative searching describe reasons for study exclusion	Fig 1 - PRISMA flow diagram

For peer review only

	and inclusion based on modifications to the research question and/or contribution to theory development)	
10. Rationale for appraisal	Describe the rationale and approach used to appraise the included studies or selected findings (e.g. assessment of conduct (validity and robustness), assessment of reporting (transparency), assessment of content and utility of the findings)	Appendix 3 Table of critical appraisal results
11. Appraisal items	State the tools, frameworks and criteria used to appraise the studies or selected findings (e.g. Existing tools: CASP, QARI, COREQ, Mays and Pope [25]; reviewer developed tools; describe the domains assessed: research team, study design, data analysis and interpretations, reporting)	Methods - Data extraction and assessing the methodological limitations
12. Appraisal process	Indicate whether the appraisal was conducted independently by more than one reviewer and if consensus was required	Methods - Data extraction and assessing the methodological limitations
13. Appraisal results	Present results of the quality assessment and indicate which articles, if any, were weighted/excluded based on the assessment and give the rationale	Appendix 4
14. Data extraction	Indicate which sections of the primary studies were analysed and how were the data extracted from the primary studies? (e.g. all text under the headings “results /conclusions” were extracted electronically and entered into a computer software)	Methods- Data management, analysis, and synthesis
15. Software	State the computer software used, if any	None
16. Number of reviewers	Identify who was involved in coding and analysis	Methods - Assessing our confidence in the review findings
17. Coding	Describe the process for coding of data (e.g. line by line coding to search for concepts)	Methods - Assessing our confidence in the review findings
18. Study comparison	Describe how were comparisons made within and across studies (e.g. subsequent studies were coded into pre-existing concepts, and new concepts were created when deemed necessary)	Methods - Assessing our confidence in the review findings
19. Derivation of themes	Explain whether the process of deriving the themes or constructs was inductive or deductive	Methods - Assessing our confidence in the review findings
20. Quotations	Provide quotations from the primary studies to illustrate themes/constructs, and identify whether the quotations were participant quotations of the author’s interpretation	Results
21. Synthesis output	Present rich, compelling and useful results that go beyond a summary of the primary studies (e.g. new	Discussion

For peer review only

	interpretation, models of evidence, conceptual models, analytical framework, development of a new theory or construct)	

For peer review only

Appendix 3. Search Strategy

Embase (inception to 2021 March 22)

1 exp Calcium/ or Calcium Carbonate/ (305629)
2 (calcium adj3 supplement*).mp. (9456)
3 1 or 2 (307747)
4 Pregnant Women/ or Prenatal Care/ (109074)
5 (pregnan* or prenatal).mp. (1086333)
6 4 or 5 (1086333)
7 3 and 6 (8440)
8 limit 7 to humans (5885)
9 limit 8 to (amphibia or ape or bird or cat or cattle or chicken or dog or "ducks and geese" or fish or "frogs and toads" or goat or guinea pig or "hamsters and gerbils" or horse or monkey or mouse or "pigeons and doves" or "rabbits and hares" or rat or reptile or sheep or swine) (277)
10 8 not 9 (5608)
11 limit 10 to (conference abstract or conference paper or "conference review" or editorial or erratum or letter or note or "review") (2567)
12 10 not 11 (**3041**)

Embase (inception to 2022 August 16) – Search update

1 exp Calcium/ or Calcium Carbonate/ (323807)
2 (calcium adj3 supplement*).mp. (10017)
3 1 or 2 (326035)
4 Pregnant Women/ or Prenatal Care/ (125455)
5 (pregnan* or prenatal).mp. (1161704)
6 4 or 5 (1161704)
7 3 and 6 (9061)
8 limit 7 to humans (6413)
9 limit 8 to (amphibia or ape or bird or cat or cattle or chicken or dog or "ducks and geese" or fish or "frogs and toads" or goat or guinea pig or "hamsters and gerbils" or horse or monkey or mouse or "pigeons and doves" or "rabbits and hares" or rat or reptile or sheep or swine) (303)
10 8 not 9 (6110)
11 limit 10 to (conference abstract or conference paper or "conference review" or editorial or erratum or letter or note or "review") (2744)
12 10 not 11 (3366)
13 limit 12 to yr="2022 - 2023" (**142**)

MEDLINE (1946 to March Week 2 2021)

1 exp Calcium/ or Calcium Carbonate/ (277850)
2 (calcium adj3 supplement*).mp. (5560)

For peer review only

3 1 or 2 (280742)
4 Pregnant Women/ or Prenatal Care/ (36709)
5 (pregnan* or prenatal).mp. (999134)
6 4 or 5 (999134)
7 3 and 6 (6417)
8 limit 7 to humans (3417)
9 limit 8 to animals (618)
10 8 not 9 (2799)
11 limit 10 to ("review articles" and case reports) (17)
12 10 not 11 (**2782 results**)

MEDLINE (inception to August Week 1, 2022) – Search update

1 exp Calcium/ or Calcium Carbonate/ (286238)
2 (calcium adj3 supplement*).mp. (5967)
3 1 or 2 (289314)
4 Pregnant Women/ or Prenatal Care/ (42373)
5 (pregnan* or prenatal).mp. (1068999)
6 4 or 5 (1068999)
7 3 and 6 6605
8 limit 7 to humans (3545)
9 limit 8 to animals (634)
10 8 not 9 (2911)
11 limit 10 to ("review articles" and case reports) (18)
12 10 not 11 (2893)
13 limit 12 to yr="2021 - 2022" (**89**)

CINAHL

CINAHL (inception to March 2021)

(calcium and supplement*) AND (pregnan* or prenatal) AND (preeclamp* or pre-eclamp* or eclamp* or "gestational hypertension" or "maternal hypertension") (**132**)

CINAHL (March 2021 to August 2022) – Search update

(calcium and supplement*) AND (pregnan* or prenatal) AND (preeclamp* or pre-eclamp* or eclamp* or "gestational hypertension" or "maternal hypertension") (**1**)

GLOBAL HEALTH

GLOBAL HEALTH (inception to March 2021)

(calcium and supplement*) AND (pregnan* or prenatal) AND (preeclamp* or pre-eclamp* or eclamp* or "gestational hypertension" or "maternal hypertension") (**158 results**)

GLOBAL HEALTH (2021 to August 2022) – Search update

(calcium and supplement*) AND (pregnan* or prenatal) AND (preeclamp* or pre-eclamp* or eclamp* or "gestational hypertension" or "maternal hypertension") (**158 results**)

For peer review only - http://bmjopen.bmj.com/site/about/guidelines.xhtml

For peer review only

For peer review only - http://bmjopen.bmj.com/site/about/guidelines.xhtml

Appendix 4. Critical Appraisal Table

Qualitative studies

STUDY DETAIL		SCREENING QUESTIONS		1. QUALITATIVE STUDIES							MMAT RATING
First author	Year	S1. Are there clear research questions?	S2. Do the collected data allow to address the research questions?	1.1. Is the qualitative approach appropriate to answer the research question? (Aim, appropriateness of a qualitative approach)	1.2. Are the qualitative data collection methods adequate to address the research question? (recruitment, data collection)	1.3. Are the findings adequately derived from the data? (rigor in analysis)	1.4. Is the interpretation of results sufficiently substantiated by data? (link from data to findings)	1.5. Is there coherence between qualitative data sources, collection, analysis and interpretation? (overall design from start to finish)	1.6. Have ethical issues been taken into consideration? (consent, confidentiality, ethics approval)	1.7. Is relationship between researcher and participants adequately considered? (interaction and reflection on how research team influences design & implementation)	
**Vestering**	**2019**	Yes	Yes	Yes	Yes	Yes	Yes	Yes	Partial	Partial	"Moderate" (minor issues impacting credibility/validity)
**Birhanu**	**2016**	Yes	Yes	Yes	Yes	Yes	Yes	Yes	Yes	Unclear	"Moderate" (minor issues impacting credibility/validity)
**Martin**	**2016**	Yes	Yes	Yes	Yes	Yes	Yes	Yes	Yes	Unclear	"Moderate" (minor issues impacting credibility/validity)
**Martin**	**2018**	Yes	Yes	Yes	Yes	Yes	Yes	Yes	Yes	Partial	"Moderate" (minor issues impacting credibility/validity)

For peer review

Quantitative studies

STUDY DETAIL		SCREENING QUESTIONS		3. NON-RANDOMIZED STUDIES					4. QUANTITATIVE DESCRIPTIVE STUDIES					MMAT RATING
First author	Year	S1. Are there clear research questions?	S2. Do the collected data allow to address the research questions?	3.1. Are the participants representative of the target population?	3.2. Are measurements appropriate regarding both the outcome and intervention (or exposure)?	3.3. Are there complete outcome data?	3.4. Are the confounders accounted for in the design and analysis?	3.5. During the study period, is the intervention administered (or exposure occurred) as intended?	4.1. Is the sampling strategy relevant to address the research question?	4.2. Is the sample representative of the target population?	4.3. Are the measurements appropriate?	4.4. Is the risk of nonresponse bias low?	4.5. Is the statistical analysis appropriate to answer the research question?	
Martin	2017	Yes	Yes	No	Yes	Unclear	No	Unclear						“Low” (some issues likely to impact credibility/validity)
Baxter	2014	Yes	Yes						Yes	Unclear	Yes	No	Yes	“Low” (some issues likely to impact credibility/validity)
Thapa	2016	Yes	Yes	Yes	Yes	Yes	Yes	Yes						"High” (no or very minor significant issues)
Nguyen	2019	Yes	Yes						Yes	Yes	Yes	Unclear	Yes	“Moderate” (minor issues impacting credibility/validity)
Omotayo	2018	Yes	Yes	Yes	Yes	Partial	Yes	Yes						“Moderate” (minor issues impacting credibility/validity)
Nguyen	2017	Yes	Yes						Yes	Yes	Yes	Yes	Yes	"High” (no or very minor significant issues)
Nguyen	2018	Yes	Yes						Yes	Yes	Yes	Yes	Yes	"High” (no or very minor significant issues)

Liu	2019	Yes	Yes						Yes	Yes	Partial	No	Yes	"Low" (some issues likely to impact credibility/validity)
Shakya Shrestha	2020	Yes	Yes						Partial	Unclear	Partial	Yes	Partial	"Very low" (significant issues impacting credibility/validity)
Ghosh-Jerath	2015	Yes	Yes						Yes	Yes	Partial	No	Yes	"Low" (some issues likely to impact credibility/validity)
Bora	2022	Yes	Yes						Yes	Yes	Partial	Unclear	Yes	"Low" (some issues likely to impact credibility/validity)

Mixed methods studies

STUDY DETAIL	First author	Omotayo	Martin	Kachwaha
	Year	2017	2017	2022
SCREENING QUESTIONS	S1. Are there clear research questions?	Yes	Yes	Yes
	S2. Do the collected data allow to address the research questions?	Yes	Yes	Yes
1. QUALITATIVE STUDIES	1.1. Is the qualitative approach appropriate to answer the research question? (Aim, appropriateness of a qualitative approach)	Yes	Yes	Yes
	1.2. Are the qualitative data collection methods adequate to address the research question? (recruitment, data collection)	Yes	Yes	yes
	1.3. Are the findings adequately derived from the data? (rigor in analysis)	Yes	Yes	Yes
	1.4. Is the interpretation of results sufficiently substantiated by data? (link from data to findings)	Yes	Yes	Yes
	1.5. Is there coherence between qualitative data sources, collection, analysis and interpretation? (overall design from start to finish)	Yes	Yes	Yes
	1.6. Have ethical issues been taken into consideration? (consent, confidentiality, ethics approval)	Yes	Yes	Yes

For peer review only - http://bmjopen.bmj.com/site/about/guidelines.xhtml

	**1.7. Is relationship between researcher and participants adequately considered? (interaction and reflection on how research team influences design & implementation)**	Unclear	Unclear	Unclear
**4. QUANTITATIVE DESCRIPTIVE STUDIES**	**4.1. Is the sampling strategy relevant to address the research question?**	Yes	Yes	Yes
	**4.2. Is the sample representative of the target population?**	Yes	Yes	Yes
	**4.3. Are the measurements appropriate?**	Yes	Yes	Yes
	**4.4. Is the risk of nonresponse bias low?**	Yes	Partial	Partial
	**4.5. Is the statistical analysis appropriate to answer the research question?**	Yes	Yes	Yes
**5. MIXED METHODS STUDIES**	**5.1. Is there an adequate rationale for using a mixed methods design to address the research question?**	Yes	Yes	Yes
	**5.2. Are the different components of the study effectively integrated to answer the research question?**	Yes	Yes	Yes
	**5.3. Are the outputs of the integration of qualitative and quantitative components adequately interpreted?**	Yes	Yes	Yes
	**5.4. Are divergences and inconsistencies between quantitative and qualitative results adequately addressed?**	Yes	Yes	Yes
	**5.5. Do the different components of the study adhere to the quality criteria of each tradition of the methods involved?**	Yes	Partial	Partial
**MMAT RATING**		"High" (no or very minor significant issues)	"Moderate" (minor issues impacting credibility/validity)	"Moderate" (minor issues impacting credibility/validity)

For peer review only

Table 2. Summary of qualitative findings

Finding no	Summary of qualitative review findings	Contributing qualitative studies	Overall CERQual assessment	Explanation of overall assessment
**1. Women's knowledge and learning**				
1.1	**Women's knowledge about pre-eclampsia or eclampsia** Most women had limited knowledge about pre-eclampsia or eclampsia, and these conditions were not typically considered by women to be a serious problem. Symptoms of pre-eclampsia such as swollen feet, severe headache, blurred vision and vomiting were considered normal signs and symptoms in pregnancy, while seizures were associated with evil attacks or nutritional deficiencies.	(18,19,38)	**High confidence**	Due to no or very minor concerns on coherence, minor concerns on methodological limitations (reflexivity and ethics), moderate concerns on relevance (1 out of 3 studies are indirectly relevant to our review aim and small number of countries), and minor concerns of adequacy (3 out of 6 contributed with 2 thick and 1 thin data).
1.2	**Information provision to women** Women felt they did not receive adequate information during pregnancy from healthcare providers about pre-eclampsia or eclampsia and calcium supplements. They described wanting to be given more information, regardless of their pre-eclampsia risk status. Women believed that having this essential information could help them to make informed choices and actively participate in their care. There were, however, mixed opinions from healthcare providers - some feared that more information could generate anxiety for women, while others were more supportive of providing information.	(18,39)	**Low confidence**	Minor concerns on methodological limitations (ethics and reflexivity), moderate concerns on coherence (no clear understanding on why some health providers worry in generating anxiety to women while the others are not), moderate concerns on relevance (small number of countries), and serious concerns on adequacy (2 out of 6 contributed with 1 thick and 1 thin data).
1.3	**Learning about calcium supplements** Women typically learned about dietary calcium, including pre-eclampsia and eclampsia symptoms, from healthcare providers. They considered healthcare providers to be the most trusted and reliable source of information, and reported feeling confident about taking calcium-containing supplements after receiving adequate information from them. Women also appreciated receiving information on calcium supplements and pre-eclampsia or eclampsia via information, education, and communication (IEC) materials like videos, media campaigns, and trusted websites.	(18,19,39)	**High confidence**	Due to no or very minor concerns on coherence, minor concerns on relevance (small number of countries), minor concerns on methodological limitations (reflexivity and ethics), and minor concerns on adequacy (3 out of 6 contributed, 1 moderate thick and 2 thin data).
**2. Women's beliefs about calcium supplements in pregnancy**				
2.1	**Fears about side-effects as a barrier to calcium supplements uptake** Women's fears about the side effects of calcium supplements affected their adherence. Women highlighted that assurance of safe use of calcium supplement is a key facilitator to consistent use. However, some women felt safety was not assured by healthcare providers, especially when calcium supplements were perceived as "experimental". Women had also received messages from their families or communities that any pills consumed during pregnancy could be harmful.	(18,19,39,40)	**High Confidence**	Due to no or very minor concerns on coherence, minor concerns on relevance (small number of countries), minor concerns on adequacy (small number of studies contributing to the qualitative evidence synthesis), and minor concerns on methodological limitations (reflexivity and ethics).
2.2	**Women's experiences of side-effects** Some women reported experiencing side effects after taking calcium and iron-folic acid supplements, such as dizziness, vomiting, nausea, stomach aches, loss of appetite, tiredness, diarrhoea, bloating, and burping, yet noted that side effects subsided with time. Women also reported that they continued consuming calcium despite these side effects.	(19,39,40)	**High Confidence**	Due to no or very minor concerns on coherence, minor concerns on methodological limitations (ethics and reflexivity), moderate concerns on relevance (only low and lower middle-income country and small number of countries) and minor concerns on adequacy (3 out of 6 studies contributed with 2 thick and 1 with thin data).
2.3	**Concerns about being stigmatized as HIV patients** Women expressed concerns that if they ingested calcium supplements, they could be stigmatized as HIV patients, which was a reported barrier to use. Some women were afraid of being stigmatized as their community often associated nutritional supplement intake and accompanying reminder posters with HIV.	(18,39)	**Moderate Confidence**	Due to no or very minor concerns on methodological limitations, no or very minor concerns on coherence, minor concerns on relevance (small number of countries), and serious concerns on adequacy (2 out of 6 study contributed).
2.4	**Positive perceptions of calcium supplements** Women reported that both their perceptions about expected benefits and previous experiences of taking calcium and iron-folic acid supplements were facilitators of use. Women believed that consuming pills could compensate for sub-optimal nutrition during pregnancy, and that supplements during pregnancy would help keep their baby safe.	(18,19,39–41)	**High Confidence**	Due to no or very minor concerns on coherence, minor concerns on methodological limitations (ethics and reflexivity), minor concerns on relevance (1 study indirectly relevant to review aim and small number of countries) and adequacy (small number of studies contributing to the qualitative evidence synthesis).
**3. Calcium supplement characteristics and regimens**				
3.1	**Varying preferences about characteristics of calcium tablets**	(19,40)	**Moderate confidence**	Due to no or very minor concerns on coherence, minor concerns on methodological limitations

For peer review only

	Positive perceptions about the characteristics of the calcium tablet played a role in motivating women to take it. Some women preferred the chewable, sweet-tasting tablets that could be swallowed without water, while others preferred the hard tablets which were smaller in size, had no smell, and needed to be taken with water. Based on individual preference, the taste, smell, size, and convenience affected calcium supplement use.			(reflexivity), moderate concerns on relevance (all low or lower middle-income countries and small number of countries) and moderate concerns on adequacy (2 out of 6 studies contributed with moderate to thick data).
**3.2**	**Supplement regimen as a barrier to use** Women described that they feel overwhelmed with the number of calcium tablets they had to take each day, especially women with other comorbidities who needed to take additional medications for their health condition s. Women felt that 3-4 pills per day at multiple times was overly onerous and preferred if they were combined into one pill.	(19,39,40)	**High Confidence**	No or very minor concerns on coherence, minor concerns on methodological limitations (reflexivity), minor concerns on adequacy (3 out of 6 studies contributed with moderate to thick data), and moderate concerns on relevance (small number of countries).
**4**	**Daily routines and food insecurity**			
**4.1**	**Adherence challenges due to routines** Adherence to calcium supplements was challenging for some women because of conflicting activities in their daily routine, such as consuming other medications, traveling, being away from home, and household chores, which can lead them to forget to take calcium.	(19,39,40)	**High Confidence**	Due to no or very minor concerns with coherence, minor concerns on methodological limitations (reflexivity), moderate concerns on relevance (small number of countries) and moderate concerns on adequacy (small number of studies contributing to qualitative evidence synthesis).
**4.2**	**Food insecurity as a barrier to calcium uptake** Women believed that adequate food was necessary to be able to take the supplements, to avoid nausea. They perceived it as normal to eat before consuming any medication. However, women reported that food insecurity was a critical barrier to calcium uptake.	(19,39)	**Moderate Confidence**	Due to no or very minor concern with coherence, minor concerns on methodological limitations (reflexivity), moderate concerns on adequacy (2 out 6 studies contributed with 1 thick and 1 thin data), and moderate concerns on relevance (small number of countries).
**5. Strategies to improve the use of calcium supplements**				
**5.1**	**Implementation of reminders to promote adherence** Women and healthcare providers perceived reminders as beneficial in promoting women's adherence to consuming calcium supplements. Several reminder strategies were described as useful by women and healthcare providers, such as home-based posters, calendars with illustrations and daily reminders, and integrating supplement intake into women's daily routine, such as mealtimes.	(19,38–40)	**High Confidence**	Due to no or very minor concerns on methodological limitations, no or very minor concerns on coherence, minor concerns on adequacy (small number of studies contributing to qualitative evidence synthesis), moderate concerns on relevance (due to all studies coming from low income or lower middle-income country and small number of countries).
**5.2**	**Importance of family support and 'adherence partner' implementation** Having family support was instrumental to pregnant women adhering to calcium supplement use. This could be leveraged by notifying family members on the importance of calcium, and appointing someone to be an "adherence partner" or "pill buddy" to help remind her to take it. Both women and healthcare providers were positive about adherence partners in providing support in terms of encouraging them to take the supplements, providing food, helping them around the house, providing emotional support, improving family relationships, and thereby increasing partner or husband involvement in pregnancy.	(38–41)	**High Confidence**	No or very minor concerns on methodological limitations, no or very minor concerns on coherence, minor concerns on adequacy (small number of studies contributing to qualitative evidence synthesis), and moderate concerns on relevance (due to all studies coming from low income or lower middle-income country only, 2 of 5 studies has indirectly relevant aim, and small number of countries).
**5.3**	**Counselling facilitates calcium supplements uptake** Both women and healthcare providers acknowledged that counselling women on the benefits of calcium was a motivator to calcium supplements uptake. Women valued the discussion they have with healthcare providers and felt more confident to take calcium supplements when they received counselling and information and pre-eclampsia or eclampsia and the benefits of calcium from their healthcare providers.	(19,39)	**Moderate Confidence**	No or very minor concerns on methodological limitations, no or very minor concerns on coherence, moderate concerns on relevance (due to all studies coming from low or lower middle-income country and small number of countries) and moderate concerns on adequacy (2 out 6 studies contributed with thick data).
**6. Healthcare provider knowledge and training**				
**6.1**	**Varied knowledge about pre-eclampsia or eclampsia among healthcare providers** Healthcare providers' knowledge about pre-eclampsia and eclampsia was varied. Some felt that pre-eclampsia and eclampsia is not a priority health concern in their area and reported never having encountered any case.	(19,38)	**Moderate Confidence**	Due to no or very minor concerns on methodological limitations and coherence, moderate concerns on relevance (due to all studies coming from low or lower middle-income countries and small number of studies) and moderate concerns on adequacy (2 out 6 studies contributed with thick data).
**6.2**	**Inadequate training to diagnose and treat pre-eclampsia and eclampsia**	(19,38,39)	**High Confidence**	No or very minor concerns on methodological limitations, coherence, moderate concerns on relevance (due to all studies coming from low

For peer review only

	While some healthcare providers mentioned that training about pre-eclampsia or eclampsia and calcium supplements was adequate, others reported that their training lacked depth and continuity, and thus felt unprepared to diagnose it and offer information these conditions and the use of calcium for prevention. Healthcare providers expressed the need to have more and continuous training on managing pre-eclampsia, as well as time to address concerns or resistance from the community.			income or lower middle-income country and small number of countries) and minor concerns on adequacy (3 out of 6 studies contributed with 2 thick and 1 thin data).
**7. Beliefs about the intervention**				
**7.1**	**Perceived overmedicalization when prescribing calcium supplements** Both healthcare providers and women perceived that prescribing more tablets to "low-risk" women during pregnancy was a form of over-medicalization of pregnancy. However, some healthcare providers felt that calcium supplements were a way to prevent further medicalization due to pre-eclampsia-related complications.	(18)	**Low confidence**	No or very minor concerns on coherence, minor concerns on methodological limitations (reflexivity and ethics), serious concerns on relevance (evidence coming from high income country only), serious concerns on adequacy (1 out of 6 study contributed).
**7.2**	**Beliefs about calcium supplements** Healthcare providers generally had positive beliefs about calcium supplements and there was optimism that calcium could be delivered through antenatal care healthcare providers. Some facilitators motivating healthcare providers to prescribe calcium supplements included their beliefs in its prevention value and expected benefits, that women liked the calcium supplements and experienced benefits from it, and a perceived lack of knowledge on how to treat pre-eclampsia which motivated healthcare providers to side towards prevention.	(18,19,39)	**High Confidence**	No or very minor concerns on coherence, minor concerns on relevance (small number of countries), minor concerns on methodological limitations (ethics and reflexivity), and minor concerns on adequacy (3 out of 6 studies contributed with 2 thick and 1 thin data).
**8. Structural factors**				
**8.1**	**High workload, inadequate staffing, stock out, and lack of equipment** In general, healthcare providers felt that their workload increased by including calcium supplements in antenatal care for pregnant women. Healthcare providers reported existing inadequate staffing, yet they needed to provide additional counselling and prescription to women, especially pregnant women with comorbidities.	(19,39)	**Moderate Confidence**	No or very minor concerns on methodological limitations, no or very minor concerns on coherence, minor concerns on relevance (due to all studies coming from low income or lower middle-income countries and small number of countries) and serious concerns on adequacy (2 out 6 studies contributed).

For peer review only

Appendix 5. Evidence Profile

No	Summary of qualitative review findings	Contributing qualitative studies	Methodological limitations assessment	Coherence assessment	Relevance assessment	Adequacy assessment	Overall CERQual assessment	Explanation of overall assessment
**Women's factors**								
**1. Women's knowledge and learning**								
**1.1**	**Women's knowledge about pre-eclampsia or eclampsia** Most women had limited knowledge about pre-eclampsia or eclampsia, and these conditions were not typically considered by women to be a serious problem. Symptoms of pre-eclampsia such as swollen feet, severe headache, blurred vision and vomiting were considered normal signs and symptoms in pregnancy, while seizures were associated with evil attacks or nutritional deficiencies.	(#2389 Martin 2016, #4971 Birhanu 2016, #293 Vestering 2019)	**Minor concerns:** Three studies with minor issues (reflexivity and ethics).	**No or very minor concerns**	**Moderate concerns:** One study is indirectly relevant to review aim and represented 3 countries (Kenya, Ethiopia, Netherlands), including 1 low-income, 1 lower middle income and 1 high income country. All perspectives came from health providers and women.	**Minor concerns:** 3 out of 6 studies contributed to review finding (2 thick data, 1 thin data).	**High confidence**	Due to no or very minor concerns on coherence, minor concerns on methodological limitations (reflexivity and ethics), moderate concerns on relevance (1 out of 3 studies are indirectly relevant to our review aim and small number of countries), and minor concerns of adequacy (3 out of 6 contributed with 2 thick and 1 thin data).
**1.2**	**Information provision to women**	(#293 Vestering	**Minor concerns:** Two studies	**Moderate concerns:**	**Moderate concerns:** All studies are	**Serious concerns:**	**Low confidence**	Minor concerns on methodological limitations (ethics and reflexivity), moderate

For peer review only

No	Summary of qualitative review findings	Contributing qualitative studies	Methodological limitations assessment	Coherence assessment	Relevance assessment	Adequacy assessment	Overall CERQual assessment	Explanation of overall assessment
	Women felt they did not receive adequate information during pregnancy from healthcare providers about pre-eclampsia or eclampsia and calcium supplements. They described wanting to be given more information, regardless of their pre-eclampsia risk status. Women believed that having this essential information could help them to make informed choices and actively participate in their care. There were, however, mixed opinions from healthcare providers - some feared that more information could generate anxiety for women, while others were more supportive of providing information.	2019, #2388 Martin 2018)	with minor issues (ethics and reflexivity).	No good understanding why some providers worry in generating anxiety to women while others are not.	directly relevant to review aim and represented 2 countries (Kenya, Netherlands), including 1 lower middle income and 1 high income country. All perspectives came from health providers and women.	2 out of 6 studies contributed to review finding (1 thick data, 1 thin data).		concerns on coherence (no clear understanding on why some providers worry in generating anxiety to women while the others are not), moderate concerns on relevance (small number of countries), and serious concerns on adequacy (2 out of 6 contributed with 1 thick and 1 thin data).
1.3	**Learning about calcium supplements** Women typically learned about dietary calcium,	(#293 Vestering 2019, #2388 Martin 2018,	**Minor concerns:** Three studies with	**No or very minor concerns**	**Minor concerns:** All studies are directly relevant	**Minor concerns:** 3 out of 6 studies	**High confidence**	Due to no or very minor concerns on coherence, minor concerns on relevance (small number of countries), minor concerns on

For peer review only

No	Summary of qualitative review findings	Contributing qualitative studies	Methodological limitations assessment	Coherence assessment	Relevance assessment	Adequacy assessment	Overall CERQual assessment	Explanation of overall assessment
	including pre-eclampsia and eclampsia symptoms, from healthcare providers. They considered healthcare providers to be the most trusted and reliable source of information, and reported feeling confident about taking calcium-containing supplements after receiving adequate information from them. Women also appreciated receiving information on calcium supplements and pre-eclampsia or eclampsia via information, education, and communication (IEC) materials like videos, media, and trusted websites.	#4971 Birhanu 2016)	minor issues (reflexivity and ethics).		to review aim and represented 3 countries (Kenya, Ethiopia, Netherlands), including 1 low-income, 1 lower middle income and 1 high income country. All perspectives came from health providers and women.	contributed to findings (2 with moderate-thick data and 1 with thin data.		methodological limitations (reflexivity and ethics), and minor concerns on adequacy (3 out of 6 contributed, 1 moderate thick and 2 thin data).
**2. Women's beliefs about calcium supplements in pregnancy**								
**2.1**	**Fears about side-effects as a barrier to calcium supplements uptake** Women's fears about the side effects of calcium supplements affected their adherence. Women	(#1837 Omotayo 2017, #4971 Birhanu 2016, #293 Vestering 2019, #2388	**Minor concerns:** Three out of four studies have minor issues (ethics and	**No or very minor concerns**	**Minor concerns:** All studies are directly relevant to review aim and represented 3	**Minor concerns** Overall, small number of studies contributing to the qualitative	**High Confidence**	Due to no or very minor concerns on coherence, minor concerns on relevance (small number of countries), minor concerns on adequacy (small number of studies contributing to the qualitative evidence synthesis),

No	Summary of qualitative review findings	Contributing qualitative studies	Methodological limitations assessment	Coherence assessment	Relevance assessment	Adequacy assessment	Overall CERQual assessment	Explanation of overall assessment
	highlighted that assurance of safe use of calcium supplement is a key facilitator to consistent use. However, some women felt safety was not assured by healthcare providers, especially when calcium supplements were perceived as "experimental". Women had also received messages from their families or communities that any pills consumed during pregnancy could be harmful.	Martin 2018)	reflexivity) and one study with no or very minor issues.		countries (Kenya, Ethiopia, Netherlands), including 1 low-income, 1 lower middle income and 1 high income country. All perspectives came from women.	evidence synthesis.		and minor concerns on methodological limitations (reflexivity and ethics).
2.2	**Women's experiences of side-effects** Some women reported experiencing side effects after taking calcium and iron-folic acid supplements, such as dizziness, vomiting, nausea, stomach aches, loss of appetite, tiredness, diarrhoea, bloating, and burping, yet noted that side effects subsided with time. Women also reported that they continued consuming calcium	(#1837 Omotayo 2017, #2388 Martin 2018, #4971 Birhanu 2016)	**Minor concerns:** Two out of three studies have minor issues (ethics and reflexivity) and one study with no or very minor issues	**No or very minor concerns**	**Moderate concerns:** All studies are directly relevant to review aim and represented 2 countries (Kenya, Ethiopia), including 1 low-income and 1 lower middle-income country.	**Minor concerns:** 3 out of 6 studies contributed to the findings (2 thin and 1 with thick data).	**High Confidence**	Due to no or very minor concerns on coherence, minor concerns on methodological limitations (ethics and reflexivity), moderate concerns on relevance (only low and lower middle-income country and small number of countries) and minor concerns on adequacy (3 out of 6 studies contributed with 2 thick and 1 with thin data).

For peer review only

No	Summary of qualitative review findings	Contributing qualitative studies	Methodological limitations assessment	Coherence assessment	Relevance assessment	Adequacy assessment	Overall CERQual assessment	Explanation of overall assessment
	despite these side effects.				All perspectives came from health providers and women.			
**2.3**	**Concerns about being stigmatized as HIV patients** Women expressed concerns that if they ingested calcium supplements, they could be stigmatized as HIV patients, which was a reported barrier to use. Some women were afraid of being stigmatized as their community often associated nutritional supplement consumption and accompanying reminder posters with HIV.	(#2388 Martin 2018, #293 vestering 2019)	**No or very minor concerns**	**No or very minor concerns**	**Moderate concerns:** All studies are directly relevant to review aim and represented 2 countries (Kenya, Netherlands), including 1 lower middle income and 1 high income country. All perspectives came from health providers and women.	**Serious concerns:** 2 out of 6 studies contributed to review finding (1 thick data and 1 thin data).	**Moderate Confidence**	Due to no or very minor concerns on methodological limitations, no or very minor concerns on coherence, minor concerns on relevance (small number of countries), and serious concerns on adequacy (2 out of 6 study contributed).
**2.4**	**Positive perceptions of calcium supplements** Women reported that both their perceptions about expected benefits and	(#293 Vestering 2019, #2388 Martin 2018, #2393 Martin	**Minor concerns:** Four out of five studies have minor	**No or very minor concerns**	**Minor concerns:** One study is indirectly relevant to	**Minor concerns** Overall, small number of studies	**High Confidence**	Due to no or very minor concerns on coherence, minor concerns on methodological limitations (ethics and reflexivity), minor concerns on relevance (1 study indirectly

No	Summary of qualitative review findings	Contributing qualitative studies	Methodological limitations assessment	Coherence assessment	Relevance assessment	Adequacy assessment	Overall CERQual assessment	Explanation of overall assessment
	previous experiences of taking calcium and iron-folic acid supplements were facilitators of use. Women believed that consuming pills could compensate for sub-optimal nutrition during pregnancy, and that supplements during pregnancy would help keep their baby safe.	2017, #4971 Birhanu 2016, #1837 Omotayo 2017)	issues (ethics and reflexivity) and one study with no or very minor issues.		review aim and represented 3 countries (Kenya, Ethiopia, Netherlands), including 1 low-income, 1 lower middle income and 1 high income country. All perspectives came from health providers and women.	contributing to the qualitative evidence synthesis.		relevant to review aim and small number of countries) and adequacy (small number of studies contributing to the qualitative evidence synthesis).
**3. Calcium supplement characteristics and regimens**								
3.1	**Varying preferences about characteristics of calcium tablets** Positive perceptions about the characteristics of the calcium tablet played a role in motivating women to take it. Some women preferred the chewable, sweet-tasting tablets that could be swallowed without water, while others preferred the	(#1837 Omotayo 2017, #4971 Birhanu 2016)	**Minor concerns:** One study has minor issues (reflexivity) and one study has no or very minor issues.	**No or very minor concerns**	**Moderate concerns:** All studies are directly relevant to review aim and represented 2 countries (Kenya, Ethiopia), including 1 low-income and 1	**Moderate concerns:** 2 out of 6 studies contributed to findings and both had moderate to thick data.	**Moderate confidence**	Due to no or very minor concerns on coherence, minor concerns on methodological limitations (reflexivity), moderate concerns on relevance (all low or lower middle-income countries and small number of countries) and moderate concerns on adequacy (2 out of 6 studies contributed with moderate to thick data).

For peer review only

No	Summary of qualitative review findings	Contributing qualitative studies	Methodological limitations assessment	Coherence assessment	Relevance assessment	Adequacy assessment	Overall CERQual assessment	Explanation of overall assessment
	hard tablets which were smaller in size, had no smell, and needed to be taken with water. Based on individual preference, the taste, smell, size, and convenience affected calcium supplement use.				lower middle-income country. All perspectives came from women.			
3.2	**Supplement regimen as a barrier to use** Women described that they feel overwhelmed with the number of calcium tablets they had to take each day, especially women with other comorbidities who needed to take additional medications for their health conditions. Women felt that 3-4 pills per day at multiple times was overly onerous and preferred if they were combined into one pill.	(# 2388 Martin 2018, #4971 Birhanu 2016, #1837 Omotayo 2017)	**Minor concerns:** Two studies have minor issues (reflexivity) and one study has no or very minor issues.	**No or very minor concerns**	**Moderate concerns:** All studies are directly relevant to review aim and represented 2 countries (Kenya, Ethiopia), including 1 low-income and 1 lower middle-income country. All perspectives came from health providers and women.	**Minor concerns:** 3 out of 6 studies contributing to findings and all have moderate to thick data.	**High Confidence**	No or very minor concerns on coherence, minor concerns on methodological limitations (reflexivity), minor concerns on adequacy (3 out of 6 studies contributed with moderate to thick data), and moderate concerns on relevance (small number of countries).
**4. Daily routines and food insecurity**								

For peer review only

No	Summary of qualitative review findings	Contributing qualitative studies	Methodological limitations assessment	Coherence assessment	Relevance assessment	Adequacy assessment	Overall CERQual assessment	Explanation of overall assessment
**4.1**	**Adherence challenges due to routines** Adherence to calcium supplements consumption was challenging for some women because of conflicting activities in their daily routine, such as consuming other medications, traveling, being away from home, and household chores, which can lead them to forget to take calcium.	(#2388 Martin 2018, #4971 Birhanu 2016, #1837 Omotayo 2017)	**Minor concerns:** Two out of three studies have minor issues (reflexivity) and one study with no or very minor issues.	**No or very minor concerns**	**Moderate concerns:** All studies are directly relevant to review aim and represented 2 countries (Kenya, Ethiopia), including 1 low-income and 1 lower middle-income country. All perspectives came from health providers and women.	**Minor concerns:** Overall, small number of studies contributing to qualitative evidence synthesis.	**High Confidence**	Due to no or very minor concerns with coherence, minor concerns on methodological limitations (reflexivity), moderate concerns on relevance (small number of countries) and moderate concerns on adequacy (small number of studies contributing to qualitative evidence synthesis).
**4.2**	**Food insecurity as a barrier to calcium uptake** Women believed that adequate food was necessary to be able to take the supplements, to avoid nausea. They perceived it as normal to eat before consuming any medication. However, women reported	(#4971 Birhanu 2016, #2388 Martin 2018)	**Minor concerns:** Two studies with minor issues (reflexivity).	**No or very minor concerns**	**Moderate concerns:** All studies are directly relevant to review aim and represented 2 countries (Kenya, Ethiopia),	**Moderate concerns:** 2 out of 6 studies contributed to findings with 1 thick and 1 thin data.	**Moderate Confidence**	Due to no or very minor concern with coherence, minor concerns on methodological limitations (reflexivity), moderate concerns on adequacy (2 out 6 studies contributed with 1 thick and 1 thin data), and moderate concerns on relevance (small number of countries).

No	Summary of qualitative review findings	Contributing qualitative studies	Methodological limitations assessment	Coherence assessment	Relevance assessment	Adequacy assessment	Overall CERQual assessment	Explanation of overall assessment
	that food insecurity was a critical barrier to calcium uptake.				including 1 low-income and 1 lower middle-income country. All perspectives came from health providers and women.			
**5. Strategies to improve the use of calcium supplements**								
**5.1**	**Implementation of reminders to promote adherence** Women and healthcare providers perceived reminders as beneficial in promoting women's adherence to consuming calcium supplements. Several reminder strategies were described as useful by women and healthcare providers, such as home-based posters, calendars with illustrations and daily reminders, and integrating supplement consumption into women's daily routine, such as mealtimes.	(#2389 Martin 2016, #2388 Martin 2018, #4971 Birhanu 2016, #1837 Omotayo 2017)	**No or very minor concerns**	**No or very minor concerns**	**Moderate concerns:** One study indirectly relevant to review aim and represented 2 countries (Kenya, Ethiopia), including 1 low-income and 1 lower middle-income country. All perspectives came from health providers and women.	**Minor concerns:** Overall, small numbers of studies contributing to qualitative evidence synthesis.	**High Confidence**	Due to no or very minor concerns on methodological limitations, no or very minor concerns on coherence, minor concerns on adequacy (small number of studies contributing to qualitative evidence synthesis), moderate concerns on relevance (due to all studies coming from low income or lower middle-income country and small number of countries).

No	Summary of qualitative review findings	Contributing qualitative studies	Methodological limitations assessment	Coherence assessment	Relevance assessment	Adequacy assessment	Overall CERQual assessment	Explanation of overall assessment
**5.2**	**Importance of family support and 'adherence partner' implementation** Having family support was instrumental to pregnant women adhering to calcium supplement use. This could be leveraged by notifying family members on the importance of calcium, and appointing someone to be an "adherence partner" or "pill buddy" to help remind her to take it. Both women and healthcare providers were positive about adherence partners in providing support in terms of encouraging them to take the supplements, providing food, helping them around the house, providing emotional support, improving family relationships, and thereby increasing partner or husband involvement in pregnancy.	(#2388 Martin 2018, #2393 Martin 2017, #4972 Birhanu 2016, #2389 Martin 2016, #1837 Omotayo 2017)	**No or very minor concerns**	**No or very minor concerns**	**Moderate concerns:** Two out of five studies indirectly relevant to review aim and represented 2 countries (Kenya, Ethiopia), including 1 low-income and 1 lower middle-income country. All perspectives came from health providers, partner and women.	**Minor concerns:** Overall, small number of studies contributing to qualitative evidence synthesis.	**High Confidence**	No or very minor concerns on methodological limitations, no or very minor concerns on coherence, minor concerns on adequacy (small number of studies contributing to qualitative evidence synthesis), and moderate concerns on relevance (due to all studies coming from low income or lower middle-income country only, 2 of 5 studies has indirectly relevant aim, and small number of countries).

For peer review only

No	Summary of qualitative review findings	Contributing qualitative studies	Methodological limitations assessment	Coherence assessment	Relevance assessment	Adequacy assessment	Overall CERQual assessment	Explanation of overall assessment
**5.3**	**Counselling facilitates calcium supplements uptake** Both women and healthcare providers acknowledged that counselling women on the benefits of calcium was a motivator to calcium supplements uptake. Women valued the discussion they have with healthcare providers and felt more confident to take calcium supplements when they received counselling and information and pre-eclampsia or eclampsia and the benefits of calcium from their healthcare providers.	(#4971 Birhanu 2016, #2388 Martin 2018)	**No or very minor concerns**	**No or very minor concerns**	**Moderate concerns:** All studies are directly relevant to review aim and represented 2 countries (Kenya, Ethiopia), including 1 low-income and 1 lower middle-income country. All perspectives came from health providers and women.	**Moderate concerns:** 2 out of 6 studies contributing to findings with all thick data.	**Moderate Confidence**	No or very minor concerns on methodological limitations, no or very minor concerns on coherence, moderate concerns on relevance (due to all studies coming from low or lower middle-income country and small number of countries) and moderate concerns on adequacy (2 out 6 studies contributed with thick data).
**Health care providers' factors**								
**6. Healthcare provider knowledge and training**								
**6.1**	**Varied knowledge about pre-eclampsia or eclampsia among healthcare providers** Healthcare providers' knowledge about pre-	(#4971 Birhanu 2016, #2389 Martin 2016, #5970	**No or very minor concerns**	**No or very minor concerns**	**Moderate concerns:** One study is indirectly relevant to	**Moderate concerns:** 2 out of 6 studies contributed to	**Moderate Confidence**	Due to no or very minor concerns on methodological limitations and coherence, moderate concerns on relevance (due to all studies coming from low or lower middle-

For peer review only

No	Summary of qualitative review findings	Contributing qualitative studies	Methodological limitations assessment	Coherence assessment	Relevance assessment	Adequacy assessment	Overall CERQual assessment	Explanation of overall assessment
	eclampsia and eclampsia was varied. Some felt that pre-eclampsia and eclampsia is not a priority health concern in their area and reported never having encountered any case.	Kachwaha 2022)			review aim and represented 2 countries (Kenya, Ethiopia), including 1 low-income and 1 lower middle-income country. All perspectives came from health providers.	findings with thick data.		income countries and small number of studies) and moderate concerns on adequacy (2 out 6 studies contributed with thick data).
6.2	**Inadequate training to diagnose and treat pre-eclampsia and eclampsia** While some healthcare providers mentioned that training about pre-eclampsia or eclampsia and calcium supplements was adequate, others reported that their training lacked depth and continuity, and thus felt unprepared to diagnose it and offer information these conditions and the use of calcium for prevention.	(#2388 Martin 2018, #4971 Birhanu 2016, #2389 Martin 2016)	**No or very minor concerns**	**No or very minor concerns**	**Moderate concerns:** One study is indirectly relevant to review aim and represented 2 countries (Kenya, Ethiopia), including 1 low-income and 1 lower middle-income country. All perspectives	**Minor concerns:** 3 out of 6 studies contributed with 2 thick and 1 thin data.	**High Confidence**	No or very minor concerns on methodological limitations, coherence, moderate concerns on relevance (due to all studies coming from low income or lower middle-income country and small number of countries) and minor concerns on adequacy (3 out of 6 studies contributed with 2 thick and 1 thin data).

No	Summary of qualitative review findings	Contributing qualitative studies	Methodological limitations assessment	Coherence assessment	Relevance assessment	Adequacy assessment	Overall CERQual assessment	Explanation of overall assessment
	Healthcare providers expressed the need to have more and continuous training on managing pre-eclampsia, as well as time to address concerns or resistance from the community.				came from health providers.			
**7. Beliefs about the intervention**								
**7.1**	**Perceived overmedicalization when prescribing calcium supplements** Both healthcare providers and women perceived that prescribing more tablets to “low-risk” women during pregnancy was a form of over-medicalization of pregnancy. However, some healthcare providers felt that calcium supplements were a way to prevent further medicalization due to pre-eclampsia-related complications.	(#293 Vestering 2019)	**Minor concerns:** One study with minor issues (reflexivity and ethics).	**No or very minor concerns**	**Serious concerns:** One study is directly relevant to review aim and represented 1 country (Netherlands), which is high income country. All perspectives came from health providers and women.	**Serious concerns:** 1 out of 6 study contributed to findings with thick data.	**Low confidence**	No or very minor concerns on coherence, minor concerns on methodological limitations (reflexivity and ethics), serious concerns on relevance (evidence coming from high income country only), serious concerns on adequacy (1 out of 6 study contributed).

For peer review only

No	Summary of qualitative review findings	Contributing qualitative studies	Methodological limitations assessment	Coherence assessment	Relevance assessment	Adequacy assessment	Overall CERQual assessment	Explanation of overall assessment
**7.2**	**Beliefs about calcium supplements** Healthcare providers generally had positive beliefs about calcium supplements and there was optimism that calcium could be delivered through antenatal care healthcare providers. Some facilitators motivating healthcare providers to prescribe calcium supplements included their beliefs in its prevention value and expected benefits, that women liked the calcium supplements and experienced benefits from it, and a perceived lack of knowledge on how to treat pre-eclampsia which motivated healthcare providers to side towards prevention.	(#2388 Martin 2018, #293 Vestering 2019, #4971 Birhanu 2016)	**Minor concerns:** Two studies with minor issues (ethics and reflexivity).	**No or very minor concerns**	**Minor concerns:** All studies are directly relevant to review aim and represented 3 countries (Kenya, Ethiopia, Netherlands), including 1 low-income, 1 lower middle income and 1 high income country. All perspectives came from health providers.	**Minor concerns:** 3 out of 6 studies contributed with 2 thick and 1 thin data.	**High Confidence**	No or very minor concerns on coherence, minor concerns on relevance (small number of countries), minor concerns on methodological limitations (ethics and reflexivity), and minor concerns on adequacy (3 out of 6 studies contributed with 2 thick and 1 thin data).
**8. Structural factors**								
**8.1**	**High workload, inadequate staffing, stock out, and lack of equipment**	(#2388 Martin 2018, #4971 Birhanu	**No or very minor concerns**	**No or very minor concerns**	**Moderate concerns:** All studies are directly relevant	**Serious concerns:** 2 out 6 studies contributed to	**Moderate Confidence**	No or very minor concerns on methodological limitations, no or very minor concerns on coherence, minor concerns on

For peer review only

No	Summary of qualitative review findings	Contributing qualitative studies	Methodological limitations assessment	Coherence assessment	Relevance assessment	Adequacy assessment	Overall CERQual assessment	Explanation of overall assessment
	In general, healthcare providers felt that their workload increased by including calcium supplements in antenatal care for pregnant women. Healthcare providers reported existing inadequate staffing, yet they needed to provide additional counselling and prescription to women, especially pregnant women with comorbidities.	2016)			to review aim and represented 2 countries (Kenya, Ethiopia), including 1 low-income and 1 lower middle-income country. All perspectives came from health providers.	findings (1 thick, 1 thin data).		relevance (due to all studies coming from low income or lower middle-income countries and small number of countries) and serious concerns on adequacy (2 out 6 studies contributed).

For peer review only

Appendix 6. Summary of Quantitative Findings

#	Summary of Quantitative review findings	Contributing quantitative studies	Quality ratings
**Women's factors**			
**1**	**Knowledge and learning**		
1.1	**Women's knowledge about pre-eclampsia or eclampsia**<br>No data supporting this theme from quantitative evidence.	No data supporting this theme from quantitative evidence.	No data supporting this theme from quantitative evidence.
1.2	**Information provision to women**<br>No data supporting this theme from quantitative evidence.	No data supporting this theme from quantitative evidence.	No data supporting this theme from quantitative evidence.
1.3	**Learning about calcium supplementation**<br>Quantitative evidence supported qualitative findings regarding women's learning on calcium supplements. In the context of an intervention implementing training to healthcare providers to reinforce calcium-related messages to women, women reported that they would take calcium in a future pregnancy and that they would recommend calcium to other pregnant women. Women were more likely to consume calcium supplements if they had higher knowledge of calcium benefits (Odds Ratio (OR) 11.7, 95% Confidence Interval (CI): 5.97-22.86) and higher general education (OR 2.59, 95% CI: 2.21-3.05). Higher adherence was also reported in those with higher education (OR 1.45, 95% CI: CI 1.31 to 1.60).(Liu et al., 2019) Higher nutrition knowledge was also associated with taking 6 times more calcium tablets. One paper, evaluating calcium supplement intake before and after nutritional interventions, showed an association between maternal knowledge of nutrition and higher calcium supplement intake (β ~31.9, 95% CI: 20.9, 43.0), however the paper also highlighted there were still large gaps between knowledge and practices, as the intake of calcium supplement tablets during 6 month was low, 82 ±66 out of the recommended 180 tablets.	(Liu et al., 2019; Nguyen et al., 2019, 2017; Thapa et al., 2016)	Four studies. 1 high quality, 1 low quality and 2 moderate quality studies.

**2**	**Believe about the intervention**		
2.1	**Fears about side-effects as barriers to calcium uptake among women**<br>Quantitative evidence supported qualitative findings as few women reported being discouraged to take the supplements by friends (4%) and elder women (3%).	(Martin et al., 2017)	1 Low quality study.
2.2	**Experiences of side effects**<br>Quantitative evidence supported the qualitative findings as some women reported experiencing side effects, usually related to gastrointestinal symptoms. The reported side-effects rates were usually low, 4% of women mentioning nausea or vomiting or constipation in one paper while in another paper 14.9% of women reported that side effects was the reason for missing a dose of IFA or calcium. These could cause supplement discontinuation or erratic supplement intake for one to 10 days after side effects were felt in around 5% of women.	(Baxter et al., 2014; Ghosh-Jerath et al., 2015; Shakya Shrestha et al., n.d.; Thapa et al., 2016)	Four studies. 1 high quality, 2 low quality and 1 very low quality study.
2.3	**Concerns of being stigmatized as HIV patient**<br>No data supporting this theme from quantitative evidence.	No data supporting this theme from quantitative evidence.	No data supporting this theme from quantitative evidence.
2.4	**Positive perceptions about calcium**<br>Quantitative evidence supported the qualitative findings that women's beliefs about the importance of calcium supplements to both the woman's and baby's health and on being able to consume it daily are associated with consuming calcium supplements. Positive beliefs about calcium supplements and self-efficacy were associated with taking calcium supplements (OR 4.6, 95% CI: 2.0 to 10.5) and with taking a higher number of supplements (OR 2.77, 95% CI: 1.68 to 4.57).	(Nguyen et al., 2019)	1 Moderate quality study.
**3**	**Calcium supplement characteristics and regimens**		
3.1	**Varying preferences about characteristics of calcium tablets**<br>Quantitative evidence supported qualitative findings regarding varying preferences calcium supplement's organoleptic properties. One paper which evaluate the impact of a program to implement calcium supplementation showed that most women (77%) reported preferences for conventional tablets that were easier to take and swallow, while the least preferred vehicle was unflavoured powder, as women dislike the taste.(Baxter et al., 2014) Another paper reported that conventional tablets had an acceptable taste (83.9% of women). Chewable tablets	(Baxter et al., 2014; Omotayo et al., 2018b; Thapa et al., 2016)	Three studies. 1 Low quality and 2 high quality studies.

	were preferred by most women (74%) in another paper. Some characteristics that women considered while taking the supplements include tablet's flavour, chewable or swallow, taken with water or not, smell, and size.		
3.2	**Adherence challenges due to routines**<br>In two quantitative studies, women preferred to take fewer tablets per day. However, in one study, women who were allocated to the study arm that used more frequent doses took more calcium overall.	(Baxter et al., 2014; Omotayo et al., 2018b, 2018a)	Three studies. 1 High quality, 1 moderate and 1 low quality studies.
**4**	**Daily routines and food insecurity**		
4.1	**Adherence challenges due to routines**<br>In quantitative evidence, women described busy work schedules and not being at home as also contributing to forgetting to consume their calcium supplements.	(Baxter et al., 2014; Shakya Shrestha et al., n.d.; Thapa et al., 2016)	Three studies. 1 High quality, 1 low and 1 very low-quality studies.
4.2	**Food insecurity as a barrier to calcium uptake**<br>Quantitative evidence supported qualitative findings that women with food security, high socio-economic status and living in urban areas are more likely to consume calcium supplements as compared to their counterparts. Food security was associated with consumption of 6 more calcium tablets.	(Liu et al., 2019; Nguyen et al., 2017)	Two studies. 1 High quality and 1 low quality studies.
**5**	**Strategies to improve use**		
5.1	**Implementation of reminders to promote adherence**<br>Quantitative evidence supported the qualitative findings that distribution of behaviour change materials to women, such as pill-taking calendars, were associated with increased adherence	(Omotayo et al., 2018b)	1 High quality study.
5.2	**Importance of family support and adherence partner implementation**<br>Quantitative evidence supported the qualitative findings that social support is important in encouraging women to consume calcium supplements. Women considered involving a husband, partner, or family in education sessions or appointing someone as the "adherence partner" to be an acceptable strategy for promoting adherence. Women often chose their husband (52%), older female relative (23%), children (14%), cousins or other relatives as adherence partners (8%), and were satisfied with the reminders and support received from their adherence partner. However, a randomised trial assessing adherence partners in improving calcium supplement intake showed that high social support, instead of	(Martin et al., 2017; Nguyen et al., 2019, 2018, 2017; Omotayo et al., 2018b)	Five studies. 3 high quality studies, 1 moderate and 1 low quality studies.

For peer review only

	adherence partners alone, was associated with higher adherence to calcium supplement (OR: 2.10; 95% CI: 1.32, 3.34). Women with high family support reported higher intake of calcium supplements (OR = 2.1).		
5.3	**Counselling facilitates calcium uptake**<br>Quantitative evidence extended qualitative findings where not only counselling, but also starting antenatal contacts at early gestational age, higher number of antenatal contacts, and receiving free calcium supplements were associated with higher calcium intake by women. One paper reports that women were 59 times more likely to consume calcium supplements if they had received them for free.	(Liu et al., 2019; Nguyen et al., 2019, 2017; Thapa et al., 2016)	Four studies. 2 High quality studies, 1 moderate and 1 low quality studies.
**Health care providers' factors**			
**6**	**Healthcare provider knowledge and training**		
6.1	**Varied knowledge about preeclampsia/eclampsia among providers**<br>No data supporting this theme from quantitative evidence.	No data supporting this theme from quantitative evidence.	No data supporting this theme from quantitative evidence.
6.2	**Inadequate training**<br>No data supporting this theme from quantitative evidence.	No data supporting this theme from quantitative evidence.	No data supporting this theme from quantitative evidence.
**7**	**Beliefs on the intervention**		
7.1	**Perceived overmedicalization when prescribing calcium supplements**<br>No data supporting this theme from quantitative evidence.	No data supporting this theme from quantitative evidence.	No data supporting this theme from quantitative evidence.
7.2	**Beliefs about calcium**<br>No data supporting this theme from quantitative evidence.	No data supporting this theme from quantitative evidence.	No data supporting this theme from quantitative evidence.
**8**	**Structural factors**		
8.1	**High workload, inadequate staffing, stock out, and lack of equipment**<br>Quantitative evidence extended qualitative findings by showing that in the context of a comprehensive integrated program including the implementation of job aids, training, guidelines, monitoring, and feedback session for healthcare providers, could overcome barriers in prescribing women with calcium supplements.	(Omotayo et al., 2018a)	1 Moderate study

For peer review only - http://bmjopen.bmj.com/site/about/guidelines.xhtml

References

Baxter, J.-A.B., Roth, D.E., Al Mahmud, A., Ahmed, T., Islam, M., Zlotkin, S.H., 2014. Tablets Are Preferred and More Acceptable Than Powdered Prenatal Calcium Supplements among Pregnant Women in Dhaka, Bangladesh. J. Nutr. 144, 1106–1112. https://doi.org/10.3945/jn.113.188524

Ghosh-Jerath, S., Devasenapathy, N., Singh, A., Shankar, A., Zodpey, S., 2015. Ante natal care (ANC) utilization, dietary practices and nutritional outcomes in pregnant and recently delivered women in urban slums of Delhi, India: an exploratory cross-sectional study. Reprod. Health 12, 20. https://doi.org/10.1186/s12978-015-0008-9

Liu, D., Cheng, Y., Dang, S., Wang, D., Zhao, Y., Li, C., Li, S., Lei, F., Qu, P., Mi, B., Zhang, R., Li, J., Zeng, L., Yan, H., 2019. Maternal adherence to micronutrient supplementation before and during pregnancy in Northwest China: a large-scale population-based cross-sectional survey. BMJ Open 9, e028843. https://doi.org/10.1136/bmjopen-2018-028843

Martin, S.L., Omotayo, M.O., Pelto, G.H., Chapleau, G.M., Stoltzfus, R.J., Dickin, K.L., 2017. Adherence-specific social support enhances adherence to calcium supplementation regimens among pregnant women. J. Nutr. 147. https://doi.org/10.3945/jn.116.242503

Nguyen, P.H., Frongillo, E.A., Sanghvi, T., Wable, G., Mahmud, Z., Tran, L.M., Aktar, B., Afsana, K., Alayon, S., Ruel, M.T., Menon, P., 2018. Engagement of Husbands in a Maternal Nutrition Program Substantially Contributed to Greater Intake of Micronutrient Supplements and Dietary Diversity during Pregnancy: Results of a Cluster-Randomized Program Evaluation in Bangladesh. J. Nutr. 148, 1352–1363. https://doi.org/10.1093/jn/nxy090

Nguyen, P.H., Kachwaha, S., Avula, R., Young, M., Tran, L.M., Ghosh, S., Agrawal, R., Escobar-Alegria, J., Patil, S., Menon, P., 2019. Maternal nutrition practices in Uttar Pradesh, India: Role of key influential demand and supply factors. Matern. Child Nutr. 15. https://doi.org/10.1111/mcn.12839

Nguyen, P.H., Sanghvi, T., Kim, S.S., Tran, L.M., Afsana, K., Mahmud, Z., Aktar, B., Menon, P., 2017. Factors influencing maternal nutrition practices in a large scale maternal, newborn and child health program in Bangladesh. PLoS One 12, e0179873. https://doi.org/10.1371/journal.pone.0179873

Omotayo, M.O., Dickin, K.L., Pelletier, D.L., Martin, S.L., Kung'u, J.K., Stoltzfus, R.J., 2018a. Feasibility of integrating calcium and iron-folate supplementation to prevent preeclampsia and anemia in pregnancy in primary healthcare facilities in Kenya. Matern. Child Nutr. 14, e12437. https://doi.org/10.1111/mcn.12437

Omotayo, M.O., Martin, S.L., Stoltzfus, R.J., Ortolano, S.E., Mwanga, E., Dickin, K.L., 2018b. With adaptation, the WHO guidelines on calcium supplementation for prevention of pre-eclampsia are adopted by pregnant women. Matern. Child Nutr. 14, e12521. https://doi.org/10.1111/mcn.12521

Shakya Shrestha, S., Adhikari, R., Tamrakar, S., Shrestha, R., Shrestha, A., n.d. Adherence to Iron, Folic Acid and Calcium Supplement and Factors Affecting it among the Antenatal Care Attending Women in a Tertiary Care Hospital: A Cross Sectional Study. Kathmandu Univ. Med. J. (KUMJ) 18, 186–192.

Thapa, K., Sanghvi, H., Rawlins, B., Karki, Y.B., Regmi, K., Aryal, S., Aryal, Y., Murakami, P., Bhattarai, J., Suhowatsky, S., K., T., H., S., B., R., Y.B., K., K., R., S., A., Y., A., P., M., J., B., S., S., 2016. Coverage, compliance, acceptability and feasibility of a program to prevent pre-eclampsia and eclampsia through calcium supplementation for pregnant women: An operations research study in one district of Nepal. BMC Pregnancy Childbirth. https://doi.org/10.1186/s12884-016-1033-6

For peer review only

Appendix 7. COM-B Mapping Table

Behavior aimed: calcium supplementation use by women and health providers						
No	COM-B Domain	TDF Domain*	List of factors affecting calcium use	Stakeholders affected (women, health providers, partner)	Evidence Source (Quantitative/Qualitative)	Barriers or facilitators to calcium use
**Women's factors**						
1	Capability	Know	Limited knowledge about preeclampsia/eclampsia	Women	Qualitative	
2	Capability	Know	Counselling on information about preeclampsia and calcium from their providers independent to woman risk	Women	Qualitative	
3	Capability	Know	High knowledge of calcium supplementation	Women	Quantitative	
4	Capability	Know	High education of women	Women	Quantitative	
5	Capability	Know	Symptoms were believed to be linked to evil attacks or nutritional deficiencies	Women	Qualitative	
6	Capability	Know	Inadequate information on preeclampsia/eclampsia and calcium from providers	Women	Qualitative	
7	Capability	Know	Receive adequate information about condition and calcium from their providers	Women	Qualitative	
8	Motivation	Bel Cons	Preeclampsia/eclampsia was not considered as a serious problem by women	Women	Qualitative	
9	Motivation	Bel Cons	Experiences and fears of side effects	Women	Qualitative and quantitative	
10	Motivation	Bel Cons	Assurance of calcium safety	Women	Qualitative	
11	Motivation	Bel Cons	Safety of calcium was not assured	Women	Qualitative	
12	Motivation	Bel Cons	Experienced and expected benefits from taking calcium and folic acid	Women	Qualitative and quantitative	
13	Motivation	Bel Cap	Belief that having essential information would help them making informed decisions	Women	Qualitative	
14	Motivation	Bel Cap	Belief that providers is a reliable source of information	Women	Qualitative	
15	Motivation	Bel Cap	Belief of being able to consume calcium daily	Women	Quantitative	
16	Motivation	Em	Valued information, education and communication (IEC) materials	Women	Qualitative	
17	Motivation	Em	Positive perceptions on calcium tablet characteristics (e.g. size, taste, smell)	Women	Qualitative and quantitative	
18	Motivation	Em	Women felt that 3-4 pills per day at multiple times were too many	Women	Quantitative and qualitative	
19	Motivation	Em	Inconvenience in taking pill daily	Women	Quantitative and qualitative	
20	Motivation	Em	Belief that one combined pill per day could ease burden	Women	Qualitative	
21	Motivation	Em	Feeling confident taking calcium after receiving adequate information from provider	Women	Quantitative	
22	Opportunity	Soc	Discouragement by family, neighbours and community in taking calcium	Women	Qualitative and quantitative	

For peer review only

23	Opportunity	Soc	Stigmatisation of having supplements and posters with HIV	Women	Qualitative
24	Opportunity	Soc	Family support in consuming calcium	Women	Qualitative and quantitative
25	Opportunity	Soc	Positive belief and experiences about 'adherence partners'	Women	Qualitative and quantitative
26	Opportunity	Ev	Conflicting daily routine with taking calcium	Women	Qualitative and quantitative
27	Opportunity	Ev	Food insecurity	Women	Qualitative and quantitative
28	Opportunity	Ev	Early initiation and frequent antenatal visits	Women	Quantitative
29	Opportunity	Ev	Receiving free calcium supplements	Women	Quantitative
30	Opportunity	Ev	Reminder tools distribution, such as home-based posters and pill-taking calendars	Women	Qualitative and Quantitative
31	Opportunity	Ev	Universal free calcium distribution through antenatal care	Women	Quantitative
**Providers factors**					
1	Capability	Know	Varied knowledge on pre-eclampsia/eclampsia	Providers	Qualitative
3	Capability	Skills	Incontinuity and non-in-depth training about pre-eclampsia/eclampsia	Providers	Qualitative
2	Capability	Skills	Felt inadequately trained	Providers	Qualitative
4	Capability	Skills	Continuous training to manage the condition and address resistance from community	Providers	Qualitative
5	Motivation	Bel Cons	Positive belief about calcium supplementation benefits	Providers	Qualitative
6	Motivation	Bel Cons	Belief that preeclampsia/eclampsia was not a priority health concern	Providers	Qualitative
7	Motivation	Bel Cons	Perceived over-medicalization to prescribe calcium to low risk women	Women and Providers	Qualitative
8	Motivation	Bel Cons	Fears in generating anxiety on low-risk women	Providers	Qualitative
9	Motivation	Bel Cons	Belief that women should get a chance to receive information regardless risk	Providers	Qualitative
10	Opportunity	Ev	Providers felt that providing calcium would increase workload	Providers	Qualitative
11	Opportunity	Ev	Inadequate number of staff providing care	Providers	Qualitative
12	Opportunity	Ev	Comprehensive integrated program (job aids, training, guidelines, regular supplies)	Providers	Quantitative

* Know: Knowledge, Phys: Physical skills, Mem: Memory, attention and decision processes, Beh Reg: Behavioural regulation, Em: Emotion, Id: Social/professional role and identity, Bel Cons: Belief about consequences, Bel Cap: Belief about capabilities, Int: Intentions, Opt: Optimisms, Ev: Environmental context and resources, Soc: Social influences

For peer review only

BMJ Open

Factors affecting the implementation of calcium supplements strategies during pregnancy to prevent pre-eclampsia: a mixed methods systematic review

Journal:	*BMJ Open*
Manuscript ID	bmjopen-2022-070677.R2
Article Type:	Original research
Date Submitted by the Author:	31-Oct-2023
Complete List of Authors:	Cormick, G; CONICET,<br>Moraa, Helen; University of Nairobi<br>Zahroh, Rana; University of Melbourne<br>Allotey, John; Univ Birmingham<br>Rocha, Thaís; University of Birmingham<br>Pena-Rosas, Juan-Pablo; Organisation mondiale de la Sante, Nutrition for Health and Development<br>Qureshi , Zahida ; University of Nairobi, Obstetrics and Gynaecology<br>Hofmeyr, G Justus ; University of the Witwatersrand Faculty of Health Sciences, Effective Care Research Unit<br>Mistry, Hema; University of Birmingham<br>Smits, Luc; Maastricht University<br>Vogel, Joshua; Burnet Institute; World Health Organization, Reproductive Health and Research<br>Palacios , Alfredo ; Institute for Clinical Effectiveness and Health Policy<br>Gwako, George N. ; University of Nairobi, Obstetrics and Gynaecology ;<br>Abalos, E; Univ Western Australia<br>Larbi, Koiwah Koi; ACTION ON PREECLAMPSIA<br>Carroli, Guillermo; CREP<br>Riley, Richard; University of Birmingham<br>Snell, Kym IE; Keele University<br>Thorson, Anna; Human Reproduction Program/World Health Organization (Geneva)<br>Young, Taryn; Stellenbosch University, Centre for Evidence-based Health Care, Division Epidemiology and Biostatistics, Dept. of Global Health<br>Betran, Ana Pilar; World Hlth Org, Reproductive Health and Research<br>Thangaratinam, Shakila; Institute of Metabolism and Systems Research University of Birmingham<br>Bohren, Meghan ; University of Melbourne School of Population and Global Health, Centre for Health Equity
**Primary Subject Heading**:	Obstetrics and gynaecology
Secondary Subject Heading:	Qualitative research, Public health, Health policy, Global health
Keywords:	Hypertension < CARDIOLOGY, OBSTETRICS, PUBLIC HEALTH

SCHOLARONE™
Manuscripts

I, the Submitting Author has the right to grant and does grant on behalf of all authors of the Work (as defined in the below author licence), an exclusive licence and/or a non-exclusive licence for contributions from authors who are: i) UK Crown employees; ii) where BMJ has agreed a CC-BY licence shall apply, and/or iii) in accordance with the terms applicable for US Federal Government officers or employees acting as part of their official duties; on a worldwide, perpetual, irrevocable, royalty-free basis to BMJ Publishing Group Ltd ("BMJ") its licensees and where the relevant Journal is co-owned by BMJ to the co-owners of the Journal, to publish the Work in this journal and any other BMJ products and to exploit all rights, as set out in our licence.

The Submitting Author accepts and understands that any supply made under these terms is made by BMJ to the Submitting Author unless you are acting as an employee on behalf of your employer or a postgraduate student of an affiliated institution which is paying any applicable article publishing charge ("APC") for Open Access articles. Where the Submitting Author wishes to make the Work available on an Open Access basis (and intends to pay the relevant APC), the terms of reuse of such Open Access shall be governed by a Creative Commons licence – details of these licences and which Creative Commons licence will apply to this Work are set out in our licence referred to above.

Other than as permitted in any relevant BMJ Author's Self Archiving Policies, I confirm this Work has not been accepted for publication elsewhere, is not being considered for publication elsewhere and does not duplicate material already published. I confirm all authors consent to publication of this Work and authorise the granting of this licence.

For peer review only

Factors affecting the implementation of calcium supplements strategies during pregnancy to prevent pre-eclampsia: a mixed-methods systematic review

Gabriela Cormick [1,2,3], Helen Moraa [4], Rana Islamiah Zahroh [5], John Allotey [6], Thais Rocha [6], Juan Pablo Peña-Rosas [7], Zahida Qureshi [8], G Justus Hofmeyr [9], Hema Mistry [10], Luc JM Smits [11], Joshua P. Vogel [12], Alfredo Palacios [13], Gwako George Nyakundi[4], Edgardo Abalos [14], Koiwah Koi-Larbi [15], Guillermo Carroli [16], Richard Riley [17], Kym Snell[17], Anna Thorson [18], Taryn Young [19], Ana Pilar Betran[18], Shakila Thangaratinam [6,17], Meghan A. Bohren[5] for I-CIP Collaborative Network

1. Centro de Investigaciones en Epidemiología y Salud Pública (CIESP-IECS), CONICET, Buenos Aires, Argentina
2. Instituto de Efectividad Clinica y Sanitaria (IECS-CONICET), Buenos Aires, Argentina
3. Departamento de Salud, Universidad Nacional de La Matanza (UNLAM), San Justo 1903, Argentina.
4. University of Nairobi
5. Gender and Women's Health Unit, Nossal Institute for Global Health, School of Population and Global Health, University of Melbourne, Carlton, Victoria, Australia
6. WHO Collaborating Centre for Global Women's Health, Institute of Metabolism and Systems Research, University of Birmingham, Birmingham, UK
7. Department of Nutrition and Food Safety, World Health Organization, Geneva, Switzerland
8. Department of Obstetrics and Gynaecology, University of Nairobi, Nairobi Kenya.
9. Department of Obstetrics and Gynaecology, University of Botswana, Gaborone, Botswana; Effective Care Research Unit, University of the Witwatersrand and Walter Sisulu University, East London, South Africa.
10. Clinical Trials Unit, Warwick Medical School, University of Warwick, Coventry, CV4 7AL, United Kingdom; University Hospitals Coventry and Warwickshire NHS Trust, Coventry, CV2 2DX, United Kingdom
11. Department of Epidemiology, CAPHRI Care and Public Health Research Institute, Maastricht University, Maastricht, The Netherlands
12. Maternal, Child and Adolescent Health Program, Burnet Institute, 85 Commercial Road, Melbourne, 3000, Australia

For peer review only - http://bmjopen.bmj.com/site/about/guidelines.xhtml

13. Department of Health Technology Assessment and Health Economics, Institute for Clinical Effectiveness and Health Policy (IECS), Buenos Aires, Argentina; Centre for Health Economics (CHE), University of York, York, United Kingdom
14. Centro de estudios de Estado y Sociedad, Buenos Aires, Argentina
15. Action on preeclampsia Charity (APEC), Ghana
16. Centro Rosarino de Estudios Perinatales (CREP), Rosario, Argentina
17. Institute of Applied Health Research, University of Birmingham, Birmingham, UK
18. UNDP-UNFPA-UNICEF-WHO-World Bank Special Programme of Research Development and Research Training in Human Reproduction (HRP), Department of Sexual and Reproductive Health and Research, World Health Organization, Geneva, Switzerland.
19. Centre for Evidence-based Health Care, Division of Epidemiology and Biostatistics, Department of Global Health, Faculty of Medicine and Health Sciences, Stellenbosch University, Stellenbosch, South Africa
20. Birmingham Women's and Children's NHS Foundation Trust, Birmingham, UK

Corresponding author: Gabriela Cormick, email: gabmick@yahoo.co.uk

For peer review only

Abstract (300 words)

Objectives Daily calcium supplements are recommended for pregnant women from 20 weeks' gestation to prevent pre-eclampsia in populations with low dietary calcium intake. This systematic review aims to improve understanding of barriers and facilitators for calcium supplement intake during pregnancy to prevent pre-eclampsia.

Design We conducted mixed-method systematic review and assessed confidence with GRADE-CERQual approach.

Data sources MEDLINE and EMBASE (via Ovid), CINAHL and Global Health (via EBSCO) and grey literature databases were searched through 17 September 2022.

Eligibility criteria We included primary qualitative, quantitative, and mixed-methods studies reporting implementation or use of calcium supplements during pregnancy, excluding calcium fortification and non-primary studies. No restrictions were imposed on settings, language, or date.

Data extraction and synthesis Two independent reviewers extracted data and assessed risk of bias. We analysed the qualitative data using thematic synthesis, and quantitative findings were thematically mapped to qualitative findings. We then mapped the results to behavioural change frameworks to identify barriers and facilitators.

Results Eighteen reports from nine studies are included in this review. Women reported barriers to consuming calcium supplements included limited knowledge about calcium supplements and pre-eclampsia, fears and experiences of side effects, varying preferences for tablets, dosing, working schedules, being away from home and taking other supplements. Receiving information regarding pre-eclampsia and safety of calcium supplement use from reliable sources, alternative dosing options, supplement reminders, early antenatal care, free supplements and support from families and communities were reported as facilitators for women. Healthcare providers felt that consistent messaging about benefits and risks of calcium, training, and ensuring adequate staffing and calcium supply is available would be able to help them in promoting calcium.

Conclusion Relevant stakeholders should consider the identified barriers and facilitators when formulating intervention and policies on calcium supplement use. These review findings can inform implementation, to ensure effective and equitable provision and scale-up of calcium interventions.

PROSPERO registration number: CRD42021239143.

For peer review only

For peer review only - http://bmjopen.bmj.com/site/about/guidelines.xhtml

Strengths and limitations of this study

- We adopted a mixed methods approach which allowed for inclusion and consolidation of studies with a range of designs.
- The strength of our review lies on behaviour change frameworks mapping which improved our understanding of how barriers and facilitators influence calcium uptake and potential strategies to address them.
- The transferability of our results may be limited, as studies were mostly from low- and middle-income countries and almost half came from the same project in Kenya and Ethiopia.
- Most included studies engaged women who attended antenatal care, therefore, might not be representative of those that do not reach the health system during pregnancy.

For peer review only

Introduction

Hypertensive disorders of pregnancy are among the leading causes of maternal and perinatal morbidity and mortality globally.(1) Pre-eclampsia is a hypertensive disorder of pregnancy characterised by hypertension developing after 20 weeks' gestation, combined with proteinuria or other new onset of maternal organ dysfunction, while eclampsia is a severe form of pre-eclampsia characterised by seizures.(2,3) Pre-eclampsia contribute to approximately 14% of the 300,000 maternal deaths worldwide annually.(4) Management of pre-eclampsia requires regular monitoring and evaluation of the woman and her baby to achieve an optimal timing of birth and prevent severe complications.(5) Preventive strategies are essential to reduce the burden of morbidity and mortality, especially in low- and middle-income countries (LMICs) where most complications occur.

The World Health Organization (WHO) recommends 1.5 to 2 grams a day from 20 weeks' gestation for women who are living in populations with low dietary calcium intake, especially those at high risk of developing pre-eclampsia. (6) This is aligned with findings from a systematic review that reported calcium supplements during pregnancy compared to placebo may reduce the risk of pre-eclampsia by 55% (13 trials, 15,730 participants; Relative Risk (RR) 0.45, 95% CI: 0.31 to 0. 65).(7) Moreover, maternal death or severe morbidity was reduced by 20% with calcium supplements (four trials, 9,732 participants; RR 0.80, 95% CI 0.66 to 0.98). (7) The evidence base has since been updated multiple times, with WHO recommendation consistently updated up to 2018.(8–10)

Despite the WHO recommendation, calcium supplement during pregnancy remains low in LMICs and rates of pre-eclampsia are not falling in regions where calcium supplementation is recommended.(10) Practical challenges to implementing WHO recommendations have been documented. For example, women need to take three, spaced tablets to achieve the requisite daily dose, and this needs be separated from timing of intake of other supplements (such as iron) to optimise calcium absorption.(11) In addition, antenatal care services need consistent supplies of calcium tablets, which can be hindered by logistical issues in supplement distribution and storage.(7) We conducted a mixed-methods systematic review aiming to improve understanding of the barriers and facilitators of calcium supplement intake during pregnancy to prevent pre-eclampsia, from the perspectives of women, families, community members, healthcare providers, and policymakers.

For peer review only - http://bmjopen.bmj.com/site/about/guidelines.xhtml

Methods

This mixed-methods review is reported according to Preferred Reporting Items for Systematic Reviews and Meta-Analyses (PRISMA) (Appendix 1) (12), Enhancing Transparency in Reporting the Synthesis of Qualitative Research (ENTREQ) statement (Appendix 2) (13), and based on guidance from Cochrane Effective Practice and Organisation of Care.(14) The protocol was registered on PROSPERO (CRD42021239143).

Topic of interest and types of studies

We included studies that documented perspectives, perceptions and experiences of women who experienced or were at risk of pre-eclampsia and/or received calcium-containing supplements. We also included studies on the views of their partners or families, as well as studies on maternity healthcare providers (e.g., midwives, nurses, doctors) and other relevant stakeholders (e.g., facility managers, policymakers) involved in decisions on calcium supplements in pregnancy. There were no limitations imposed on geographical location or type of health facility. The timeframe for using calcium supplement is during pregnancy, independent of the gestational age.

We included primary qualitative, quantitative, and mixed-methods studies reporting implementation or use of calcium supplements in any presentations including powder, granule, chewable tablet, capsule, liquid filled capsule, tablet, suspension, or powder for suspension. We did not include studies assessing the effects of calcium fortified foods or beverages. We excluded case reports or case series, letters, editorials, commentaries, reviews, study protocols, posters, and conference abstracts or other study sources that did not provide primary data.

Search methods for identification of studies

We searched MEDLINE and EMBASE via Ovid, CINAHL and Global Health via EBSCO to identify eligible studies from inception to 17 September 2022. A search strategy was developed and adapted for each database (Appendix 3), using different terms for calcium and pregnancy. No limitations on publication date or language were imposed. Grey literature searches were also conducted using OpenGrey and Google. We checked reference lists of included studies to identify any relevant record not retrieved in the database search.

Selection of studies

After removing duplicates in EndNote, records were imported to Covidence for screening.(15) Two of the following authors (GC, GG, APB, RIZ, HM) independently assessed eligibility of each record by comparing titles and abstracts against the eligibility criteria. Full texts of potentially eligible papers were retrieved and assessed, disagreements were resolved through discussion or consulting a third author. Papers emerging from the same study were collated and treated as one data source. Titles and abstracts of papers published in languages other than English, French, and Spanish were translated through open-source software (Google Translate) to assess their eligibility. Had we identified any relevant titles or abstracts

For peer review only - http://bmjopen.bmj.com/site/about/guidelines.xhtml

in languages other than English, French and Spanish, we would have sought formal translation of the full texts from a native speaker.

Data extraction and assessing the methodological limitations

Using a pre-designed form, two reviewers (GC, RIZ) independently extracted data from included studies on study characteristics (setting, sample size, characteristics of participants, objectives), design (data collection and analysis methods), qualitative data (themes, findings, and quotations) and quantitative data (data source, outcome measures, results, measures of compliance or uptake).

All included studies underwent quality appraisal by two authors (GC, HM, RIZ). As the review included quantitative, qualitative and mixed-methods studies, we used the Mixed Methods Appraisal Tool (MMAT), which produces a single quality rating on the basis of: aims, methodology, design, recruitment, data collection, blinding, data analysis, selective reporting, reflexivity, ethical considerations, results, research contribution, and other sources of bias.(16) Appraisal of study quality was used to inform data analysis, and not to exclude studies.

Quality appraisal, analysis and assessing confidence

We conducted a preliminary qualitative synthesis using a thematic analysis approach.(17) Thematic analysis is a valuable method in analysing qualitative data to examine perspectives, preferences, experiences, acceptability, feasibility, and other factors that can influence implementation.(17) The analysis begins with initial readings to build our familiarity with the data. Two reviewers (GC, HM) independently conducted line-by-line coding of findings of two qualitative studies.(18,19) From this we developed the qualitative codebook, which was then used to code all other included studies. Next, we generated analytical themes and interpretations to explore relationships within and across studies. This was achieved by organising codes into a hierarchy and identifying barriers and facilitators between study characteristics and findings or exploring different findings across studies. Once qualitative themes were generated, a summary of qualitative findings was developed. Quantitative findings were then narratively mapped to qualitative themes to explore areas of convergence or divergence. ATLAS.ti was used to manage data analysis.

After the thematic analysis, we mapped the qualitative themes to the Theoretical Domains Framework (TDF) and Capability, Opportunity, and Motivation of Behaviour (COM-B) models.(20,21) TDF and COM-B are interrelated behaviour change models which can guide implementation research and intervention design in understanding barriers and facilitators of intended behaviours. We used TDF and COM-B to explore barriers and facilitators of healthcare providers and women to use calcium supplements during pregnancy using evidence-based behaviours to identify potential behaviour change intervention strategies.

We used the GRADE-CERQual (Grading of Recommendations, Assessment, Development, and Evaluations - Confidence in the Evidence from Reviews of Qualitative research) approach to assess confidence in each qualitative finding. (22) GRADE-CERQual assessed confidence based on four key components: methodological limitations (23), coherence (24), adequacy (25), and

For peer review only - http://bmjopen.bmj.com/site/about/guidelines.xhtml

relevance. (26) After assessing each of the four components, we assessed the overall confidence (22) as high, moderate, low, or very low.

Patient and Public Involvement

None.

Results

We included 18 papers from 16 studies (Figure 1). Included papers were published in English between 2014 and 2022. Out of 16 studies, 10 were quantitative and came from 11 papers (27–37), four were qualitative (18,19,38,39) and two were mixed-methods and came from three papers (40–42) Detailed study characteristics can be found in Table 1.

Table 1. Characteristics of included studies

Study	Country	Population	Number of participants	Methods	Ref
Qualitative					
Birhanu 2018	Ethiopia	Pregnant women and healthcare providers	20 women, 22 healthcare providers	Phenomology	19
Martin 2017a	Kenya	Pregnant women and healthcare providers	22 women, 20 healthcare providers	Phenomology	38
Martin 2018	Kenya	Healthcare providers, pregnant women, adherence partners	7 healthcare providers, 32 pregnant women, 20 adherence partners	Phenomology	39
Vestering 2019	Netherlands	Healthcare providers and pregnant women	8 healthcare providers, 25 women	Phenomology	18
Mixed methods					
Martin 2017b & Omatoyo 2018a	Kenya and Ethiopia	Pregnant women	85 pregnant women, 50 in Ethiopia, 38 in Kenya	Trials of improved practices	41, 40
Kachwaha 2022	India	Healthcare providers, supervisors, and facility staffs	~500 healthcare providers and supervisors, and 20 block level staffs	Mixed methods	42
Quantitative					
Omotayo 2018b & Martin 2017c	Kenya	Pregnant women	990 pregnant women and 16 facilities	Process evaluation study adopting program impact pathway	33, 28
Nguyen 2019	India	Pregnant women and recently delivered women	667 pregnant women and 1835 recently delivered women	Cross-sectional household surveys	32
Nguyen 2017	Bangladesh	Pregnant women and recently delivered women	600 pregnant women and 2000 recently delivered women	Cross-sectional household surveys	34
Nguyen 2018	Bangladesh	Women and husbands	1000 women and 70% of their husbands	Cluster randomised control trial with cross-sectional household surveys	35
Bora 2022	India	Pregnant women	3,097,274	Cross-sectional study	27
Baxter 2014	Bangladesh	Pregnant women	132	Modified discrete-choice trial	30
Thapa 2016	Nepal	Pregnant and postpartum women, health facilities, female community health volunteers	1240	Prospective collection and secondary analysis of monitoring data captured by the MOHP	31
Liu 2019	China	Women aged from 16 to 49 years who were pregnant between 2010 and 2013 and had specific pregnancy outcomes before the survey	30,027	Cross-sectional study	36

For peer review only - http://bmjopen.bmj.com/site/about/guidelines.xhtml

Shakya Shrestha 2020	Nepal	Pregnant women	191	Cross-sectional study	37
Ghosh-Jareth 2015	India	Pregnant women and recently delivered women	184 pregnant women and 160 recently delivered women	Cross-sectional study	29

Five studies aimed to evaluate the implementation of calcium supplements in pregnancy.(27,30,31,39,40) Seven studies evaluated the incorporation of calcium supplement recommendation to other recommended supplements taken during pregnancy, including aspirin (18), and iron and folic acid supplements. (19,28,33,37,38,41,42) Four studies focused on general nutritional practices during pregnancy (29,32,34,35) and one study on all types of micronutrient supplements used before and during pregnancy. (36) Five studies came from one project, Micronutrient Initiative-Cornell University Calcium (MICa) projects (19,28,33,38–41), and four studies came from Alive & Thrive (A&T) project (32, 34, 35, 42).

The studies were conducted in seven different countries across three regions. In Sub-Saharan Africa, three studies were conducted in Kenya (28, 33, 38,39), one study in Ethiopia (19), and one study in Kenya in Ethiopia (40,41). In Asia, four studies were conducted in India (27, 29, 32, 42), three studies in Bangladesh (30, 34, 35), two studies in Nepal (31,37), and one study in China (36). There is one study conducted in Europe, specifically the Netherlands. (18)

All four qualitative studies involved pregnant women and healthcare providers (18,19,38), while one study also included adherence partners to support and remind women to take medication. (39) One mixed-method study included pregnant women (40,41) and one included healthcare providers and facility staff. (42) Among the ten quantitative studies, eight included pregnant women or women who had recently given birth (27–30,32,33, 34,36,37), one included healthcare providers (31) and one included both women and their husbands. (35)

Results of the critical appraisal of the included studies are available in Appendix 4. The main areas of concern for qualitative studies were an unclear or partial description of reflexivity and limited information regarding ethical considerations. For the quantitative studies, main concerns were regarding the appropriateness of measurement tools, lack of detail regarding nonresponse bias, and insufficient information regarding statistical analysis.

Qualitative and quantitative synthesis

We identified five themes related to factors affecting calcium supplement intake during pregnancy: 1) women's existing knowledge and learning; 2) women's beliefs about calcium supplements; 3) calcium supplement characteristics and dose regimens; 4) challenges due to daily routines and food insecurity; and 5) strategies to improve the use of calcium. We also identified three themes related to factors affecting healthcare providers' prescription of calcium supplements: 1) health provider knowledge and training; 2) their beliefs about calcium supplements; and 3) structural factors on site. Across all themes, there were 19 qualitative findings (Table 2): 11 findings were high confidence, six were moderate confidence

For peer review only - http://bmjopen.bmj.com/site/about/guidelines.xhtml

and two were low confidence (Appendix 5: Evidence profile). The quantitative findings which were mapped to qualitative themes can be found in Appendix 6.

Table 2. Summary of qualitative findings

No	Summary of qualitative review findings	Contributing qualitative studies	Overall CERQual assessment	Explanation of overall assessment
**1. Knowledge and learning**				
**1.1**	**Women's knowledge about pre-eclampsia/eclampsia** Most women had limited knowledge about preeclampsia/eclampsia, and preeclampsia/eclampsia was not typically considered as a serious problem by women. General symptoms of preeclampsia/eclampsia such as swollen feet, severe headache, blurred vision and vomiting were considered normal in pregnancy, while seizures were linked to evil attacks or nutritional deficiencies	(26,27,34)	**High confidence**	Due to no or very minor concerns on coherence, minor concerns on methodological limitations (reflexivity and ethics), moderate concerns on relevance (1 out of 3 studies are indirectly relevant to our review aim and small number of countries), and minor concerns of adequacy (3 out of 6 contributed with 2 thick and 1 thin data)
**1.2**	**Information provision to women** Women felt they did not receive adequate information during pregnancy from health workers about preeclampsia/eclampsia and calcium supplementation and would like to be given more information regardless of their risk status. Women believed that having this essential information could help them to make informed choices and to actively participate in their care. There were, however, mixed opinions from health workers, where some feared that more information could generate anxiety for women, while others were supportive of providing information to women	(13,34)	**Low confidence**	Minor concerns on methodological limitations (ethics and reflexivity), moderate concerns on coherence (no clear understanding on why some health workers worry in generating anxiety to women while the others are not), moderate concerns on relevance (small number of countries), and serious concerns on adequacy (2 out of 6 contributed with 1 thick and 1 thin data)
**1.3**	**Learning about calcium supplementation** Women typically learned about calcium, including pre-eclampsia and eclampsia symptoms, from health workers, and considered health workers as the most trusted and reliable source of information. They reported feeling confident about taking calcium after receiving adequate information from their health workers. Women also appreciated receiving information on calcium supplementation and preeclampsia/eclampsia from information, education, and communication (IEC) materials like videos, media and trusted websites	(13,27,34)	**High confidence**	Due to no or very minor concerns on coherence, minor concerns on relevance (small number of countries), minor concerns on methodological limitations (reflexivity and ethics), and minor concerns on adequacy (3 out of 6 contributed, 1 moderate thick and 2 thin data)
**2. Believe about the intervention**				
**2.1**	**Fears about side-effects as barriers to calcium uptake among women** Fears about side effects impact adherence to calcium consumption by women. Women highlighted that assurance of safe use of calcium is a key facilitator to consistent use. However, some women felt safety was not assured by health workers and were told by their families or communities that any pills consumed during pregnancy could be harmful, especially when the intervention was perceived as "experimental"	(11,13,27,34)	**High Confidence**	Due to no or very minor concerns on coherence, minor concerns on relevance (small number of countries), minor concerns on adequacy (small number of studies contributing to the qualitative evidence synthesis), and minor concerns on methodological limitations (reflexivity and ethics)
**2.2**	**Experiences of side effects** Some women reported experiencing side effects after taking calcium and iron folic acid, which include dizziness, vomiting, nausea, stomach-ache, loss appetite, tiredness, diarrhea, bloating, and burping, yet noted that the side effects subsided with time. Women also reported to keep consuming the medication despite experiencing the side effects	(11,13,27)	**High Confidence**	Due to no or very minor concerns on coherence, minor concerns on methodological limitations (ethics and reflexivity), moderate concerns on relevance (only low and lower middle-income country and small number of countries) and minor concerns on adequacy (3 out of 6 studies contributed with 2 thick and 1 with thin data)
**2.3**	**Concerns of being stigmatized as HIV patient** Women expressed concerns about being stigmatized as HIV patients if they ingested calcium, which was a reported barrier to use. Some women were afraid of being stigmatized as their community often associated supplement consumption and accompanying reminder posters with HIV	(13,34)	**Moderate Confidence**	Due to no or very minor concerns on methodological limitations, no or very minor concerns on coherence, minor concerns on relevance (small number of countries), and serious concerns on adequacy (2 out of 6 study contributed)
**2.4**	**Positive perceptions about calcium** Women reported that both their perceptions about expected benefits and previous experiences of taking calcium and iron-folic acid were facilitators of use. Women believed that consuming pills could compensate for sub-optimal nutrition during pregnancy and that consuming nutrient supplementation during pregnancy would help keep the baby safe	(11,13,27,28, 34)	**High Confidence**	Due to no or very minor concerns on coherence, minor concerns on methodological limitations (ethics and reflexivity), minor concerns on relevance (1 study indirectly relevant to review aim and small number of countries) and adequacy (small number of studies contributing to the qualitative evidence synthesis)
**3. Medication characteristics and doses**				
**3.1**	**Varying preferences about characteristics of calcium tablets** Positive perceptions about the characteristics of the calcium tablet played a role in motivating women to take it. Some women preferred the chewable, sweet-tasting tablets that could be swallowed without water, while others preferred the hard tablets which were smaller in size, had no smell, and	(11,27)	**Moderate confidence**	Due to no or very minor concerns on coherence, minor concerns on methodological limitations (reflexivity), moderate concerns on relevance (all low or lower middle-income countries and small number of countries) and moderate concerns on adequacy (2 out of 6 studies contributed with moderate to thick data)

For peer review only - http://bmjopen.bmj.com/site/about/guidelines.xhtml

	needed to be taken with water. Based on individual preference, the taste, smell, size, and convenience were instrumental in uptake of the calcium supplements			
**3.2**	**Medication dosing as a barrier of use** Women described that they could feel overwhelmed with the number of calcium pills they had to take per day, this includes women with comorbidities who need to take additional medications to manage their health condition. Women felt that 3-4 pills per day at multiple times was overly onerous and recommended combining them into one pill could ease the burden	(11,13,27)	**High Confidence**	No or very minor concerns on coherence, minor concerns on methodological limitations (reflexivity), minor concerns on adequacy (3 out of 6 studies contributed with moderate to thick data), and moderate concerns on relevance (small number of countries)
**4**	**Routine and food insecurity**			
**4.1**	**Adherence challenges due to routines** Adherence to calcium consumption was challenging for some women because of conflicting activities in women's daily routine such as consuming other medications, travelling, being away from home and household chores, which make women forgetting to take their medication	(11,13,27)	**High Confidence**	Due to no or very minor concerns with coherence, minor concerns on methodological limitations (reflexivity), moderate concerns on relevance (small number of countries) and moderate concerns on adequacy (small number of studies contributing to qualitative evidence synthesis)
**4.2**	**Food insecurity as a barrier to calcium uptake** Women believed that adequate foo d was necessary to be able to take the supplements, to avoid nausea associated with the supplement and perceived it as a standard practice to eat before consuming any medication. However, women reported that food insecurity was a critical barrier to calcium uptake	(13,27)	**Moderate Confidence**	Due to no or very minor concern with coherence, minor concerns on methodological limitations (reflexivity), moderate concerns on adequacy (2 out 6 studies contributed with 1 thick and 1 thin data), and moderate concerns on relevance (small number of countries)
**5. Strategies to improve use**				
**5.1**	**Implementation of reminders to promote adherence** Women and health workers perceived reminders as beneficial in promoting women's adherence in consuming calcium. Several reminder strategies were deemed to be useful by women and health workers, such as home-based posters, calendars with illustrations and daily reminders, and integrating supplement consumption into the women's daily routine such as at mealtime	(11,13,26,27)	**High Confidence**	Due to no or very minor concerns on methodological limitations, no or very minor concerns on coherence, minor concerns on adequacy (small number of studies contributing to qualitative evidence synthesis), moderate concerns on relevance (due to all studies coming from low income or lower middle-income country and small number of countries)
**5.2**	**Importance of family support and adherence partner implementation** Having family support was instrumental to women in adhering to calcium use and could be leveraged by notifying them on the importance of women's adherence to consumption and appointing someone to be woman's "adherence partner" to help remind woman in taking the supplement. Both women and health workers were positive about adherence partners in providing support in terms of encouraging them to take the supplements, providing food, helping them around the house, emotional support, improving family relationships, and increased partner or husband involvement in pregnancy	(11,13,26,28)	**High Confidence**	No or very minor concerns on methodological limitations, no or very minor concerns on coherence, minor concerns on adequacy (small number of studies contributing to qualitative evidence synthesis), and moderate concerns on relevance (due to all studies coming from low income or lower middle-income country only, 2 of 5 studies has indirectly relevant aim, and small number of countries)
**5.3**	**Counselling facilitates calcium uptake** Both women and health workers and acknowledged that the counselling they received from health workers was a motivator to calcium uptake. Women valued the discussion they have with health workers and felt more confident to take calcium supplements when they received counselling on information of preeclampsia/eclampsia and the benefits of calcium from their health workers	(13,27)	**Moderate Confidence**	No or very minor concerns on methodological limitations, no or very minor concerns on coherence, moderate concerns on relevance (due to all studies coming from low or lower middle-income country and small number of countries) and moderate concerns on adequacy (2 out 6 studies contributed with thick data)
**6. Knowledge and training**				
**6.1**	**Varied knowledge about preeclampsia or eclampsia among health workers** Health workers' knowledge about preeclampsia and eclampsia was variable among health workers, and some health workers felt that preeclampsia or eclampsia is not a priority health concern in their area and reported never having encountered any case	(26,27, 30)	**Moderate Confidence**	Due to no or very minor concerns on methodological limitations and coherence, moderate concerns on relevance (due to all studies coming from low or lower middle-income countries and small number of studies) and moderate concerns on adequacy (2 out 6 studies contributed with thick data)
**6.2**	**Inadequate training to diagnose and treat pre-eclampsia and eclampsia** While some health workers mentioned that training about preeclampsia/eclampsia and calcium supplementation was adequate, others reported that their training lacked depth and continuity, and thus felt unprepared to diagnose and offer information about preeclampsia/eclampsia and calcium to pregnant women. Health workers expressed the needs to have more and continuous training to manage the condition and to address any concerns and resistance from the community	(13,26,27)	**High Confidence**	No or very minor concerns on methodological limitations, coherence, moderate concerns on relevance (due to all studies coming from low income or lower middle-income country and small number of countries) and minor concerns on adequacy (3 out of 6 studies contributed with 2 thick and 1 thin data)
**7. Beliefs on the intervention**				
**7.1**	**Perceived overmedicalization when prescribing calcium supplementation** Both health workers and women perceived that prescribing more pills to general low-risk women during pregnancy was a form of over-medicalization of pregnancy. Some health workers, however, felt that calcium supplementation during pregnancy was a way to prevent further medicalization when complications occurred	(34)	**Low confidence**	No or very minor concerns on coherence, minor concerns on methodological limitations (reflexivity and ethics), serious concerns on relevance (evidence coming from high income country only), serious concerns on adequacy (1 out of 6 study contributed)
**7.2**	**Beliefs that pre-eclampsia is not a serious problem in their settings** Health workers generally had positive beliefs about calcium supplementation and there was optimism that calcium could be delivered through antenatal care health workers. Some facilitators motivating health workers in prescribing calcium supplementation include belief in its prevention value and expected benefits, women liked the calcium supplements, and benefits experienced by women, and perceived lack of knowledge on how to treat preeclampsia which motivated health workers to err towards prevention	(13,27,34)	**High Confidence**	No or very minor concerns on coherence, minor concerns on relevance (small number of countries), minor concerns on methodological limitations (ethics and reflexivity), and minor concerns on adequacy (3 out of 6 studies contributed with 2 thick and 1 thin data)
**8. Structural factors**				
**8.1**	**Increased workload** In general, health workers felt that their workload increased by	(13,27)	**Moderate Confidence**	No or very minor concerns on methodological limitations, no or very minor concerns on coherence, minor concerns

For peer review only - http://bmjopen.bmj.com/site/about/guidelines.xhtml

	including calcium supplementation in the services they were providing to pregnant women. Health workers reported inadequate number of staff providing care, yet they needed to provide additional counselling and prescribing to women, especially pregnant women with comorbidities.	on relevance (due to all studies coming from low income or lower middle-income countries and small number of countries) and serious concerns on adequacy (2 out 6 studies contributed)

For peer review only

Women’s knowledge and learning

Women’s knowledge about pre-eclampsia

Most women had limited knowledge about pre-eclampsia, and these conditions were not typically considered by women to be a serious problem. Symptoms of pre-eclampsia such as swollen feet, severe headache, blurred vision and vomiting were considered normal signs and symptoms in pregnancy, while seizures were associated with evil attacks or nutritional deficiencies (Finding 1.1 – High confidence). (18,19,38) Women and healthcare providers from Ethiopia and Kenya stated that there was no local language for pre-eclampsia, which made it difficult for healthcare providers to explain the condition to women, and served as a barrier in providing adequate knowledge to women and encouraging them to use calcium supplements. (19,38)

Information provision to women

Women felt they did not receive adequate information during pregnancy from healthcare providers about pre-eclampsia and calcium supplements. They described wanting to be given more information, regardless of their pre-eclampsia risk status. Women believed that having this essential information could help them to make informed choices and actively participate in their care. There were, however, mixed opinions from healthcare providers - some feared that more information could generate anxiety for women, while others were more supportive of providing information (Finding 1.2 – Low confidence). (18,39) Healthcare providers were worried that sharing information about pre-eclampsia, especially with low-risk pregnant women, could lead to anxiety and make an “uncomplicated pregnancy more stressful”. (18) Healthcare providers viewed their role as informants, but not as decision-makers for women. They believed that it should be woman’s choice to decide whether to consume calcium supplements or not. (18) Women and healthcare providers mentioned that the scope of information provided to women should include symptoms of pre-eclampsia as well as effectiveness, benefits, and safety of calcium-containing supplements. (18)

Learning about calcium supplements

Women typically learned about dietary calcium, including pre-eclampsia symptoms, from healthcare providers. They considered healthcare providers to be the most trusted and reliable source of information and reported feeling confident about taking calcium-containing supplements after receiving adequate information from them. Women also appreciated receiving information on calcium supplements and pre-eclampsia via information, education, and communication (IEC) materials like videos, media campaigns, and trusted websites (Finding 1.3 – High confidence). (18,19,32,34,39) Quantitative evidence supported qualitative findings regarding women’s learning on calcium supplements. In the context of an intervention implementing training to healthcare providers to reinforce calcium-related messages to women, women reported that they would take calcium in a future pregnancy and that they would recommend calcium to other pregnant women. (31)

For peer review only

Women were more likely to consume calcium supplements if they had higher knowledge of calcium benefits (Odds Ratio (OR) 11.7, 95% Confidence Interval (CI): 5.97-22.86) and higher general education (OR 2.59, 95% CI: 2.21-3.05). (32) Higher adherence was also reported in those with higher education (OR 1.45, 95% CI: CI 1.31 to 1.60).(36) Higher nutrition knowledge was also associated with taking 6 times more calcium tablets.(32)
One paper, evaluating calcium supplement intake before and after nutritional interventions, showed an association between maternal knowledge of nutrition and higher calcium supplement intake (β ~31.9, 95% CI: 20.9, 43.0), however the paper also highlighted there were still large gaps between knowledge and practices, as the intake of calcium supplement tablets during 6 month was low, 82 ±66 out of the recommended 180 tablets.(34)

Women's beliefs about calcium supplements in pregnancy

Fears about side-effects as a barrier to calcium supplements uptake

Women's fears about the side effects of calcium supplements affected their adherence. Women highlighted that assurance of safe use of calcium supplement is a key facilitator to consistent use. However, some women felt safety was not assured by healthcare providers, especially when calcium supplements were perceived as "experimental". Women had also received messages from their families or communities that any pills consumed during pregnancy could be harmful. (Finding 2.1 – High confidence). (18,19,39,40) Quantitative evidence supported qualitative findings as few women reported being discouraged to take supplements by friends (4%) and elder women (3%).(28)

Women's experiences of side-effects

Some women reported experiencing side effects after taking calcium and iron-folic acid supplements, such as dizziness, vomiting, nausea, stomach aches, loss of appetite, tiredness, diarrhoea, bloating, and burping, yet noted that side effects subsided with time. Women also reported that they continued taking calcium supplements despite these side effects (Finding 2.2 – High confidence). (19,39,40) Quantitative evidence supported the qualitative findings as some women reported experiencing side effects, usually related to gastrointestinal symptoms. (29–31,37) The reported side-effects rates were usually low, 4% of women mentioning nausea or vomiting or constipation in one paper while in another paper 14.9% of women reported that side effects was the reason for missing a dose of IFA or calcium.(30,37) These could cause supplement discontinuation or erratic supplement intake for one to 10 days after side effects were felt in around 5% of women.(37)

Concerns about being stigmatized as HIV patients

Women expressed concerns that if they took calcium supplements, they could be stigmatized as HIV patients, which was a reported barrier to use. Some women were afraid of being stigmatized as their community often associated nutritional supplement intake and accompanying reminder posters, with HIV (Finding 2.3 – Moderate confidence). (18,39)

For peer review only - http://bmjopen.bmj.com/site/about/guidelines.xhtml

Positive perceptions of calcium supplements

Women reported that both their perceptions about expected benefits and previous experiences of taking calcium and iron-folic acid supplements were facilitators of use. Women believed that taking pills could compensate for sub-optimal nutrition during pregnancy, and that supplements during pregnancy would help keep their baby safe (Finding 2.4 – High confidence). (18,19,39–41) Women also reported reduced cravings to consume soil (pica) during pregnancy (a cultural practice or cravings characterised by recurrent ingestion of unusually high amounts of soil, and is related to iron deficiency anaemia, which is common during pregnancy). (39) Furthermore, some women appreciated the emphasis of "prevention" when encouraging supplement intake. (19,41) Quantitative evidence supported the qualitative findings that women's beliefs about the importance of calcium supplements to both woman's and baby's health are associated with calcium supplement intake. (32) Positive beliefs about calcium supplements and self-efficacy were associated with taking calcium supplements (OR 4.6, 95% CI: 2.0 to 10.5) and with taking a higher number of supplements (OR 2.77, 95% CI: 1.68 to 4.57). (32)

Calcium supplement characteristics and regimens

Varying preferences about characteristics of calcium tablets

Positive perceptions about the characteristics of the calcium tablet played a role in motivating women to take it. Some women preferred the chewable, sweet-tasting tablets that could be swallowed without water, while others preferred the hard tablets which were smaller in size, had no smell, and needed to be taken with water. Based on individual preference, the taste, smell, size, and convenience affected calcium supplement use (Finding 3.1 – Moderate confidence). (19,40)

Quantitative evidence supported qualitative findings regarding varying preferences calcium supplement's organoleptic properties. One paper which evaluate the impact of a program to implement calcium supplementation showed that most women (77%) reported preferences for conventional tablets that were easier to take and swallow, while the least preferred vehicle was unflavoured powder, as women dislike the taste.(30) Another paper reported that conventional tablets had an acceptable taste (83.9% of women).(31) Chewable tablets were preferred by most women (74%) in another paper. (40) Some characteristics that women considered while taking the supplements include tablet's flavour, chewable or swallow, taken with water or not, smell, and size.(30,31,40)

Supplement regimen as a barrier to use

Women described that they feel overwhelmed with the number of calcium tablets they had to take each day, especially women with other comorbidities who needed to take additional medications for their health conditions. Women felt that 3-4 pills per day at multiple times was onerous and preferred if they were combined into one pill (Finding 3.2 – High confidence). (19,39,40) In two quantitative studies, women preferred taking fewer tablets per

For peer review only - http://bmjopen.bmj.com/site/about/guidelines.xhtml

day. (30,40) However, those allocated to the study arm that used more frequent doses took more calcium overall. (40)

Daily routines and food insecurity

Adherence challenges due to routines

Adherence to calcium supplements was challenging for some women because of conflicting activities in their daily routine, such as taking other medications or supplements, traveling, being away from home, and household chores, which can lead them to forget to take calcium (Finding 4.1 – High confidence). (19,39,40) In quantitative evidence, women described busy work schedules and not being at home as also contributing to forgetting to consume their calcium supplements. (30,31,37) Forgetting to take calcium supplements was the most frequent reason (52.1%) for not taking IFA or calcium supplements and busy work schedules was inversely associated with adherence to calcium supplementation. (31,37)

Food insecurity as a barrier to calcium uptake

Women believed that adequate food was necessary to take the supplements and to avoid nausea. They perceived it as normal to eat before consuming any medication. However, women reported that food insecurity was a critical barrier to calcium uptake (Finding 4.2 – Moderate confidence). (19,39) Quantitative evidence supported qualitative findings that women with food security, high socio-economic status and living in urban areas are more likely to consume calcium supplements as compared to their counterparts.(34,36) Food security was associated with taking 6 more calcium tablets. (34)

Strategies to improve the use of calcium supplements

Implementation of reminders to promote adherence

Women and healthcare providers perceived reminders as beneficial in promoting women's adherence to calcium supplements. Several reminder strategies were described as useful by women and healthcare providers, such as home-based posters, calendars with illustrations and daily reminders, and integrating calcium supplement intake into women's daily routine, such as mealtimes (Finding 5.1 – High confidence). (19,38–40) Quantitative evidence supported the qualitative findings that distribution of behaviour change materials to women, such as pill-taking calendars, were associated with increased adherence. (40)

Importance of family support and 'adherence partner' implementation

Having family support was instrumental to pregnant women adhering to calcium supplements. This could be leveraged by notifying family members on the importance of calcium, and appointing someone to be an "adherence partner" or "pill buddy" to help remind her to take it. Both women and healthcare providers were positive about adherence partners in providing support in terms of encouraging them to take the supplements, providing food, helping them around the house, providing emotional support, improving family relationships,

For peer review only - http://bmjopen.bmj.com/site/about/guidelines.xhtml

and thereby increasing partner or husband involvement in pregnancy (Finding 5.2 – High confidence). (19,38–41) Women could choose who their adherence partner was, and some opted for their husband, a male or female relative, or their child. Some women reported that the support they received from adherence partners decreased over time (19,39–41), suggesting challenges with sustaining appropriate intake throughout pregnancy.

Quantitative evidence supported the qualitative findings that social support is important in encouraging women to take calcium supplements. (28,32,34,35,40) Women considered involving a husband, partner, or family in education sessions or appointing someone as the "adherence partner" to be an acceptable strategy for promoting adherence. (28,32,34,35) Women often chose their husband (52%), older female relative (23%), children (14%), cousins or other relatives as adherence partners (8%) (28), and were satisfied with the reminders and support received from their adherence partner. (28,32,34) However, a randomised trial assessing adherence partners in improving calcium supplement intake showed that high social support, instead of adherence partners alone, was associated with higher adherence to calcium supplements (OR: 2.10; 95% CI: 1.32, 3.34). (28) Women with high family support reported higher intake of calcium supplements (OR = 2.1).(32)

Counselling facilitates calcium supplements uptake

Both women and healthcare providers acknowledged that counselling women on the benefits of calcium was a motivator to calcium supplement intake. Women valued the discussion they have with healthcare providers and felt more confident to take calcium supplements when they received counselling and information and pre-eclampsia and the benefits of calcium from their healthcare providers (Finding 5.3 – Moderate confidence). (19,39) This was confirmed by healthcare providers who reported that they have seen positive results following counselling women on iron-folic acid supplements and believed that this would be replicated for calcium-containing supplements. (39)

Quantitative evidence extended qualitative findings where not only counselling, but also starting antenatal contacts at early gestational age, higher number of antenatal contacts, and receiving free calcium supplements were associated with higher calcium intake by women. (31,32,34,36) One paper reports that women were 59 times more likely to consume calcium supplements if they had received them for free.(32)

Healthcare provider factors

Healthcare provider knowledge and training

Varied knowledge about pre-eclampsia among healthcare providers

Healthcare providers' knowledge about pre-eclampsia was varied. Some felt that pre-eclampsia is not a priority health concern in their area and reported never having encountered any case (Finding 6.1 – Moderate confidence). (19,38,42)

For peer review only

Inadequate training to diagnose and treat pre-eclampsia

While some healthcare providers mentioned that training about pre-eclampsia and calcium supplements was adequate, others reported that their training lacked depth and continuity, and thus felt unprepared to diagnose it and offer information these conditions and the use of calcium for prevention. Healthcare providers expressed the need to have more and continuous training on managing pre-eclampsia, as well as time to address concerns or resistance from the community (Finding 6.2 – High confidence). (19,38,39) Healthcare providers had positive views of trainings and IEC materials and felt that it helps improve their knowledge. (42) They also valued supervision visits which help them solve problems and increases their accountability. (42) Support was also needed throughout calcium roll-out to ensure challenges can be addressed promptly during implementation. (19)

Beliefs about the intervention

Perceived overmedicalization when prescribing calcium supplements

Both healthcare providers and women perceived that prescribing more tablets to "low-risk" women during pregnancy was a form of over-medicalization of pregnancy. However, some healthcare providers felt that calcium supplements were a way to prevent further medicalization due to pre-eclampsia-related complications (Finding 7.1 – Low confidence). (18)

Beliefs about calcium supplements

Healthcare providers generally had positive beliefs about calcium supplements and there was optimism that calcium could be delivered through antenatal care healthcare providers. Some facilitators motivating healthcare providers to prescribe calcium supplements included their beliefs in its prevention value and expected benefits, that women liked the calcium supplements and experienced benefits from it, and a perceived lack of knowledge on how to treat pre-eclampsia which motivated healthcare providers to side towards prevention (Finding 7.2 – High confidence). (18,19,39)

Structural factors

High workload, inadequate staffing, stock out, and lack of equipment

In general, healthcare providers felt that their workload increased by including calcium supplements in antenatal care for pregnant women. Healthcare providers reported existing inadequate staffing, yet they needed to provide additional counselling and prescription to women, especially pregnant women with comorbidities (Finding 8.1 – Moderate confidence). (19,39) Stock-outs were also reported as critical barriers, often due to logistical issues in the supply chain (e.g., centralization or procurement changes), errors in demand estimation by government staff, and inadequate storage facilities. (42) Importantly, some healthcare providers reported that a lack of equipment to diagnose pre-eclampsia was a barrier to

For peer review only - http://bmjopen.bmj.com/site/about/guidelines.xhtml

calcium implementation .(19) Facility staff members and supervisors reported that utilisation of health information systems to monitor calcium stocks, checklists to provide feedback on counselling, and gaps to address, as well as collaboration with government staff members, could be facilitators of use. (42)

Quantitative evidence extended qualitative findings by showing that in the context of a comprehensive integrated program including the implementation of job aids, training, guidelines, monitoring, and feedback session for healthcare providers, could overcome barriers in prescribing women with calcium supplements. (33)

Mapping to behaviour change models

Through COM-B and TDF mapping (Appendix 7), we identified that the critical domains on facilitators and barriers to improve calcium use among women, which include: knowledge, beliefs about consequences, beliefs about capabilities, emotion, social influences, and environmental context and resources to improve calcium use by women. To encourage calcium prescription by healthcare providers, facilitators and barriers related to knowledge, skills, beliefs about consequences and environmental context domains should be addressed. Figure 2 shows the categorisation of barriers and facilitators across the Capability, Opportunity and Motivation Behaviour. The mapping shows that factors encouraging women's use of and adherence to calcium includes receiving adequate information about pre-eclampsia through counselling with healthcare providers and IEC materials, assurance of calcium safety, receiving preferred characteristics of tablet and doses, family and community support, early and frequent antenatal contacts, free calcium supplements, and reminder tools distribution. Likewise, factors that may encourage healthcare providers to prescribe calcium supplements include continuous training about identification and management of pre-eclampsia and calcium supplements, dissemination of consistent messages, reminders, and ensuring adequate number of human resources, equipment for diagnosing pre-eclampsia, and availability of calcium pills at health facilities.

Discussion

We included 18 papers from 16 studies conducted primarily in LMICs and reporting views of women, adherence partners, and healthcare providers. Our review shows the importance of healthcare providers' knowledge and training about calcium supplements and pre-eclampsia, as women reported providers as the most reliable sources of information and reassurance on safety of calcium and would encourage adherence. Promoting early initiation of antenatal care and consistent messages on pre-eclampsia and calcium supplementation may improve women's use of calcium supplements. Free calcium supplements and options on doses and tablet preferences could help overcome barriers to calcium supplement use for women. Reminder systems and support from family and community may also help increase women´s calcium uptake.

Women play an important role in decision-making about calcium supplement during pregnancy. Our review shows that women's limited knowledge about and fears of side effects, and potential impacts on their baby serve as the critical barriers to calcium

For peer review only - http://bmjopen.bmj.com/site/about/guidelines.xhtml

supplement intake. These barriers about calcium supplementation have been reported on the use of other supplements during pregnancy. For example, studies on factors affecting multiple micronutrient supplementation, iron folic acid, and lipid-based nutrients reported women's knowledge, acceptance, motivation, and attitudes toward the medication play an important role. (43,44) Furthermore, a study on factors affecting use of intervention for preterm management also reported women felt hesitant in consuming the medications to improve labour outcome due to fears about baby's growth and development.(45) This highlights the need to ensure that women are aware on the benefits of the supplement and given assurance on the safety of its use.

As the most trusted informants, healthcare providers knowledge about calcium and pre-eclampsia are important to support women uptake of calcium supplements. Studies reported different levels of knowledge about calcium and pre-eclampsia among providers, with some providers having persistent beliefs that evidence on calcium supplements during pregnancy is still in the experimental phase. Some healthcare providers might be reluctant to talk about pre-eclampsia with the fear to stress low risk women; however, this reluctance should be balanced by the risk of symptoms of pre-eclampsia going unnoticed by women. Reinforcing messages related to improving pregnancy outcomes and similarities to other supplements taken during pregnancy such as folic acid and iron that also help to may facilitate use of calcium supplements. Healthcare providers also need to know about how to assess eligibility for calcium supplements, including how to screen and score women at high-risk and to identify populations with low calcium intake. There is lack of acceptable biomarkers of individual calcium intake and calcium status, which complicates screening individuals.(46) WHO recommendations on calcium supplementation were set for populations with low calcium intake, as dietary assessments are more reliable to identify populations with low calcium intake rather than to identify individuals. (46)

Human resource shortages are a recurrent health system challenge, particularly in LMICs which results in overburdened staff unable to deliver recommended practices. Appropriate staffing, particularly of midwives and nurses who provide most antenatal care services, remains crucial to achieve quality of care. Unfortunately, this is also applicable on the context of other medication delivered during pregnancy. For example, providers reported unavailability of stock, inadequate staffs and equipment as the main barriers in prescribing interventions to pregnant women experiencing preterm labour. Therefore, ensuring availability of diagnostic tools and calcium stocks is critical to ensure appropriate prescription and delivery of care.(45) Where health providers constraints persist, innovative strategies to streamline antenatal care practices may be needed to improve efficiency.(47)

Implications for research, policy, and practice

The TDF and COM-B mapping in our review can be used by researchers and programme managers to inform the development of implementation models to optimise the use of calcium supplements. Assessing the extent to which the barriers and facilitators to calcium prescription and use identified in our review are potential implementation challenges in different contexts can be a useful starting point for formative research to scale-up implementation. Table 2 presents a list of questions derived from our findings and may help programme implementers, policymakers, researchers, and other stakeholders to identify

For peer review only - http://bmjopen.bmj.com/site/about/guidelines.xhtml

and address factors that may affect prescription and use of calcium supplements during pregnancy. Assessing the extent to which the barriers and facilitators identified in our review are potential implementation challenges in different settings is a useful starting point for formative research to scale this intervention.

Table 2. Implications for research, policy, and practice

Domain	List of questions
**Guidelines and protocols**	1. Are guidelines and clinical protocols on pre-eclampsia/eclampsia and calcium supplements during pregnancy consistent between WHO, national, and facility-levels?
**Knowledge and learning**	2. Do women or healthcare providers have scepticism or concerns about adverse effects of calcium supplements during pregnancy that can be addressed?<br>3. Do women and their family members receive education and educational materials about signs of pre-eclampsia/eclampsia early in pregnancy?<br>4. Do women have sufficient time and opportunity to discuss pre-eclampsia/eclampsia with healthcare providers during antenatal care?<br>5. Do women have sufficient time and opportunity to discuss calcium supplementation with healthcare providers during antenatal care, including addressing fears about side effects, managing side effects, safety concerns, and reinforcing positive messaging about expected benefits?<br>6. Have concerns from both women and healthcare providers about calcium supplements as a form of overmedicalisation of pregnancy been addressed in culturally-appropriate ways?<br>7. Have healthcare providers received in-service training on pre-eclampsia/eclampsia prevention and management, including the importance of calcium supplements during pregnancy for prevention?
**Strategies to improve use**	8. Do all relevant cadres of healthcare providers (including midwives and nurses) have authority to prescribe calcium supplements during pregnancy?<br>9. Do women have the opportunity to try different types of calcium tablets to suit their preferences, such as chewable/non-chewable, different tastes, and different size tablets?<br>10. Do women have the opportunity to try different calcium dosing combinations to suit their schedules and preferences?<br>11. For women experiencing or at risk of food insecurity during pregnancy, are there additional social services to support adequate nutrition intake during pregnancy?<br>12. Have different types of reminder systems (e.g., posters for home, calendars, and integration into daily routines) been designed with women and their families to encourage use?<br>13. Has support from family and/or adherence partners been integrated for women, and do family members or adherence partners have the opportunity to attend educational sessions?<br>14. Are stocks of calcium readily available in the antenatal care wards?<br>15. Is there sufficient funding and budget allocation to ensure continuous procurement and distribution of calcium supplements?

Strengths and limitations

Most included studies were from low- and middle-income countries and almost half came from the same project conducted in Kenya and Ethiopia, which may limit transferability of results to other contexts. The results from our review therefore can be used to guide formative research as well as implementation and programme planning in other contexts.

For peer review only - http://bmjopen.bmj.com/site/about/guidelines.xhtml

Most included studies engaged women who attended antenatal care; therefore, might not be representative of those that do not reach the health system during pregnancy. None of the included studies reported the perspectives of policymakers.

Despite these limitations, this is the first systematic review of barriers and facilitators to calcium supplement use during pregnancy. We adopted a mixed methods approach which allowed for inclusion and consolidation of studies with a range of designs. Mapping the review findings to behaviour change models improved understanding of how barriers and facilitators influence calcium uptake, and consequently can be addressed in future interventional or programmatic work.

Conclusion

Our review identified a range of barriers and facilitators affecting calcium supplements during pregnancy to prevent pre-eclampsia. When formulating intervention and policies on calcium supplement use, relevant stakeholders should consider the identified barriers and facilitators to optimise uptake. Findings from this study can inform implementation considerations, to ensure effective and equitable provision and scale-up of calcium interventions.

Funding: This work was funded by the Medical Research Council [grant number MR/T038861/1] and the UNDP-UNFPA-UNICEF-WHO-World Bank Special Programme of Research, Development and Research Training in Human Reproduction (HRP), a cosponsored programme executed by the World Health Organization (WHO). MAB's time is supported by an Australian Research Council Discovery Early Career Researcher Award (DE200100264) and a Dame Kate Campbell Fellowship (University of Melbourne Faculty of Medicine, Dentistry and Health Sciences).

Competing interests: None declared

Ethics: Ethical approval was not required; no primary data were collected.

Patient consent for publication: Not required.

Contributors: All authors have made substantial contributions to the following: Conceptualisation: ST, APB, MAB, JA, GC Data curation: TR, JA, GC, HM, RIZ Formal analysis: RIZ, GC, HM, MAB, Funding acquisition: ST, APB, JA, Investigation and methodology: RIZ, MAB, GC, HM Writing – original draft: GC, HM, RIZ, MAB, Writing – review and editing: GC, HM, RIZ, JA, TR, JPP, ZQ, GJH, HM, LS, JPV, AP, GNG, EA, KKL, GC, RR, KS, TA, TY, APB, TS and MAB.

Data availability statement: All data relevant to the study are included in the article or uploaded as supplementary information.

For peer review only - http://bmjopen.bmj.com/site/about/guidelines.xhtml

For peer review only

For peer review only - http://bmjopen.bmj.com/site/about/guidelines.xhtml

References

1. Poon LC, Shennan A, Hyett JA, Kapur A, Hadar E, Divakar H, et al. The International Federation of Gynecology and Obstetrics (FIGO) initiative on pre-eclampsia: A pragmatic guide for first-trimester screening and prevention. Int J Gynecol Obstet. 2019;145(S1).

2. Mol BWJ, Roberts CT, Thangaratinam S, Magee LA, De Groot CJM, Hofmeyr GJ. Pre-eclampsia. In: The Lancet. 2016. p. 999–1011.

3. Gestational Hypertension and Preeclampsia: ACOG Practice Bulletin, Number 222. Obstet Gynecol. 2020;135(6).

4. Say L, Chou D, Gemmill A, Tunçalp Ö, Moller A-B, Daniels J, et al. Global causes of maternal death: a WHO systematic analysis. Lancet Glob Heal [Internet]. 2014 Jun;2(6):e323–33. Available from: https://linkinghub.elsevier.com/retrieve/pii/S2214109X1470227X

5. Steegers EAP, von Dadelszen P, Duvekot JJ, Pijnenborg R. Pre-eclampsia. Lancet [Internet]. 2010 Aug;376(9741):631–44. Available from: https://linkinghub.elsevier.com/retrieve/pii/S0140673610602796

6. Prevention and treatment of pre-eclampsia and eclampsia. World Health. 2011.

7. Hofmeyr GJ, Lawrie TA, Atallah ÁN, Torloni MR. Calcium supplementation during pregnancy for preventing hypertensive disorders and related problems. Vol. 2018, Cochrane Database of Systematic Reviews. 2018.

8. WHO recommendation: Calcium supplementation during pregnancy for the prevention of pre-eclampsia and its complications. Geneva; 2018.

9. WHO recommendation on Calcium supplementation before pregnancy for the prevention of pre-eclampsia and its complications. Geneva; 2020.

10. WHO Recommendations on Antenatal Care for a Positive Pregnancy Experience. Geneva; 2013.

11. Omotayo MO, Dickin KL, O'Brien KO, Neufeld LM, De Regil LM, Stoltzfus RJ. Calcium Supplementation to Prevent Preeclampsia: Translating Guidelines into Practice in Low-Income Countries. Adv Nutr. 2016;7(2):275–8.

12. Page MJ, McKenzie JE, Bossuyt PM, Boutron I, Hoffmann TC, Mulrow CD, et al. The PRISMA 2020 statement: An updated guideline for reporting systematic reviews. Vol. 372, The BMJ. 2021.

13. Tong A, Flemming K, McInnes E, Oliver S, Craig J. Enhancing transparency in reporting the synthesis of qualitative research: ENTREQ. BMC Med Res Methodol. 2012;12.

14. Glenton C, Lewin S, Downe S, Paulsen E, Munabi-Babigumira S, Johansen M, et al. Qualitative Evidence Syntheses Within Cochrane Effective Practice and Organisation of Care: Developing a Template and Guidance. Int J Qual Methods. 2021;20.

For peer review only

For peer review only - http://bmjopen.bmj.com/site/about/guidelines.xhtml

15. Covidence systematic review software. Melbourne, Australia: Veritas Health Innovation;

16. Hong QN, Fàbregues S, Bartlett G, Boardman F, Cargo M, Dagenais P, et al. The Mixed Methods Appraisal Tool (MMAT) version 2018 for information professionals and researchers. Educ Inf. 2018;34(4).

17. Thomas J, Harden A. Methods for the thematic synthesis of qualitative research in systematic reviews. BMC Med Res Methodol [Internet]. 2008 Dec 10;8(1):45. Available from: https://bmcmedresmethodol.biomedcentral.com/articles/10.1186/1471-2288-8-45

18. Vestering A, Bekker MN, Grobbee DE, van der Graaf R, Franx A, Crombag NMT, et al. Views and preferences of medical professionals and pregnant women about a novel primary prevention intervention for hypertensive disorders of pregnancy: a qualitative study. Reprod Health [Internet]. 2019 May 2;16(1):46. Available from: http://www.ncbi.nlm.nih.gov/pubmed/31046778

19. Birhanu Z, Chapleau GM, Ortolano SE, Mamo G, Martin SL, Dickin KL. Ethiopian women's perspectives on antenatal care and iron-folic acid supplementation: Insights for translating global antenatal calcium guidelines into practice. Matern Child Nutr [Internet]. 2018 Feb;14:e12424. Available from: https://onlinelibrary.wiley.com/doi/10.1111/mcn.12424

20. Michie S, van Stralen MM, West R. The behaviour change wheel: A new method for characterising and designing behaviour change interventions. Implement Sci [Internet]. 2011 Dec 23;6(1):42. Available from: http://implementationscience.biomedcentral.com/articles/10.1186/1748-5908-6-42

21. Atkins L, Francis J, Islam R, O'Connor D, Patey A, Ivers N, et al. A guide to using the Theoretical Domains Framework of behaviour change to investigate implementation problems. Implement Sci [Internet]. 2017 Dec 21;12(1):77. Available from: http://implementationscience.biomedcentral.com/articles/10.1186/s13012-017-0605-9

22. Lewin S, Bohren M, Rashidian A, Munthe-Kaas H, Glenton C, Colvin CJ, et al. Applying GRADE-CERQual to qualitative evidence synthesis findings—paper 2: how to make an overall CERQual assessment of confidence and create a Summary of Qualitative Findings table. Implement Sci [Internet]. 2018 Jan 25;13(S1):10. Available from: https://implementationscience.biomedcentral.com/articles/10.1186/s13012-017-0689-2

23. Munthe-Kaas H, Bohren MA, Glenton C, Lewin S, Noyes J, Tunçalp Ö, et al. Applying GRADE-CERQual to qualitative evidence synthesis findings—paper 3: how to assess methodological limitations. Implement Sci [Internet]. 2018 Jan 25;13(S1):9. Available from: https://implementationscience.biomedcentral.com/articles/10.1186/s13012-017-0690-9

24. Colvin CJ, Garside R, Wainwright M, Munthe-Kaas H, Glenton C, Bohren MA, et al. Applying GRADE-CERQual to qualitative evidence synthesis findings—paper 4: how to assess coherence. Implement Sci [Internet]. 2018 Jan 25;13(S1):13. Available from: https://implementationscience.biomedcentral.com/articles/10.1186/s13012-017-0691-8

For peer review only

For peer review only - http://bmjopen.bmj.com/site/about/guidelines.xhtml

25. Glenton C, Carlsen B, Lewin S, Munthe-Kaas H, Colvin CJ, Tunçalp Ö, et al. Applying GRADE-CERQual to qualitative evidence synthesis findings—paper 5: how to assess adequacy of data. Implement Sci [Internet]. 2018 Jan 25;13(S1):14. Available from: https://implementationscience.biomedcentral.com/articles/10.1186/s13012-017-0692-7

26. Noyes J, Booth A, Lewin S, Carlsen B, Glenton C, Colvin CJ, et al. Applying GRADE-CERQual to qualitative evidence synthesis findings–paper 6: how to assess relevance of the data. Implement Sci [Internet]. 2018 Jan 25;13(S1):4. Available from: https://implementationscience.biomedcentral.com/articles/10.1186/s13012-017-0693-6

27. Bora K, Barman B, Pala S, Das A, Doke G, Tripura A. Coverage of antenatal iron-folic acid and calcium distribution during pregnancy and their contextual determinants in the northeastern region of India. Front Nutr [Internet]. 2022 Jul 18;9. Available from: https://www.frontiersin.org/articles/10.3389/fnut.2022.894245/full

28. Martin SL, Omotayo MO, Pelto GH, Chapleau GM, Stoltzfus RJ, Dickin KL. Adherence-specific social support enhances adherence to calcium supplementation regimens among pregnant women. J Nutr. 2017;147(4).

29. Ghosh-Jerath S, Devasenapathy N, Singh A, Shankar A, Zodpey S. Ante natal care (ANC) utilization, dietary practices and nutritional outcomes in pregnant and recently delivered women in urban slums of Delhi, India: an exploratory cross-sectional study. Reprod Health [Internet]. 2015 Dec 20;12(1):20. Available from: https://reproductive-health-journal.biomedcentral.com/articles/10.1186/s12978-015-0008-9

30. Baxter J-AB, Roth DE, Al Mahmud A, Ahmed T, Islam M, Zlotkin SH. Tablets Are Preferred and More Acceptable Than Powdered Prenatal Calcium Supplements among Pregnant Women in Dhaka, Bangladesh. J Nutr [Internet]. 2014 Jul 1;144(7):1106–12. Available from: https://academic.oup.com/jn/article/144/7/1106/4569766

31. Thapa K, Sanghvi H, Rawlins B, Karki YB, Regmi K, Aryal S, et al. Coverage, compliance, acceptability and feasibility of a program to prevent pre-eclampsia and eclampsia through calcium supplementation for pregnant women: An operations research study in one district of Nepal. BMC Pregnancy Childbirth. 2016;

32. Nguyen PH, Kachwaha S, Avula R, Young M, Tran LM, Ghosh S, et al. Maternal nutrition practices in Uttar Pradesh, India: Role of key influential demand and supply factors. Matern Child Nutr [Internet]. 2019 Oct 18;15(4). Available from: https://onlinelibrary.wiley.com/doi/10.1111/mcn.12839

33. Omotayo MO, Dickin KL, Pelletier DL, Martin SL, Kung'u JK, Stoltzfus RJ. Feasibility of integrating calcium and iron-folate supplementation to prevent preeclampsia and anemia in pregnancy in primary healthcare facilities in Kenya. Matern Child Nutr [Internet]. 2018 Feb;14:e12437. Available from: https://onlinelibrary.wiley.com/doi/10.1111/mcn.12437

34. Nguyen PH, Sanghvi T, Kim SS, Tran LM, Afsana K, Mahmud Z, et al. Factors influencing

For peer review only - http://bmjopen.bmj.com/site/about/guidelines.xhtml

maternal nutrition practices in a large scale maternal, newborn and child health program in Bangladesh. de Souza RJ, editor. PLoS One [Internet]. 2017 Jul 10;12(7):e0179873. Available from: https://dx.plos.org/10.1371/journal.pone.0179873

35. Nguyen PH, Frongillo EA, Sanghvi T, Wable G, Mahmud Z, Tran LM, et al. Engagement of Husbands in a Maternal Nutrition Program Substantially Contributed to Greater Intake of Micronutrient Supplements and Dietary Diversity during Pregnancy: Results of a Cluster-Randomized Program Evaluation in Bangladesh. J Nutr [Internet]. 2018 Aug 1;148(8):1352–63. Available from: https://academic.oup.com/jn/article/148/8/1352/5040613

36. Liu D, Cheng Y, Dang S, Wang D, Zhao Y, Li C, et al. Maternal adherence to micronutrient supplementation before and during pregnancy in Northwest China: a large-scale population-based cross-sectional survey. BMJ Open [Internet]. 2019 Aug 8;9(8):e028843. Available from: https://bmjopen.bmj.com/lookup/doi/10.1136/bmjopen-2018-028843

37. Shakya Shrestha S, Adhikari R, Tamrakar S, Shrestha R, Shrestha A. Adherence to Iron, Folic Acid and Calcium Supplement and Factors Affecting it among the Antenatal Care Attending Women in a Tertiary Care Hospital: A Cross Sectional Study. Kathmandu Univ Med J (KUMJ) [Internet]. 18(70):186–92. Available from: http://www.ncbi.nlm.nih.gov/pubmed/33594028

38. Martin SL, Seim GL, Wawire S, Chapleau GM, Young SL, Dickin KL. Translating formative research findings into a behaviour change strategy to promote antenatal calcium and iron and folic acid supplementation in western Kenya. Matern Child Nutr [Internet]. 2017 Jan 22;13(1):mcn.12233. Available from: https://onlinelibrary.wiley.com/doi/10.1111/mcn.12233

39. Martin SL, Wawire V, Ombunda H, Li T, Sklar K, Tzehaie H, et al. Integrating Calcium Supplementation into Facility-Based Antenatal Care Services in Western Kenya: A Qualitative Process Evaluation to Identify Implementation Barriers and Facilitators. Curr Dev Nutr [Internet]. 2018 Nov 1;2(11). Available from: https://academic.oup.com/cdn/article/doi/10.1093/cdn/nzy068/5078341

40. Omotayo MO, Martin SL, Stoltzfus RJ, Ortolano SE, Mwanga E, Dickin KL. With adaptation, the WHO guidelines on calcium supplementation for prevention of pre-eclampsia are adopted by pregnant women. Matern Child Nutr [Internet]. 2018 Apr;14(2):e12521. Available from: http://doi.wiley.com/10.1111/mcn.12521

41. Martin SL, Omotayo MO, Chapleau GM, Stoltzfus RJ, Birhanu Z, Ortolano SE, et al. Adherence partners are an acceptable behaviour change strategy to support calcium and iron-folic acid supplementation among pregnant women in Ethiopia and Kenya. Matern Child Nutr [Internet]. 2017 Jul;13(3):e12331. Available from: https://onlinelibrary.wiley.com/doi/10.1111/mcn.12331

42. Kachwaha S, Nguyen PH, Tran LM, Avula R, Young MF, Ghosh S, et al. Specificity Matters: Unpacking Impact Pathways of Individual Interventions within Bundled Packages Helps Interpret the Limited Impacts of a Maternal Nutrition Intervention in India. J Nutr. 2022

For peer review only

For peer review only - http://bmjopen.bmj.com/site/about/guidelines.xhtml

Feb;152(2):612–29.

43. Clermont A, Kodish S, Matar Seck A, Salifou A, Rosen J, Grais R, et al. Acceptability and Utilization of Three Nutritional Supplements during Pregnancy: Findings from a Longitudinal, Mixed-Methods Study in Niger. Nutrients [Internet]. 2018 Aug 12;10(8):1073. Available from: http://www.mdpi.com/2072-6643/10/8/1073

44. Garcia-Casal MN, Estevez D, De-Regil LM. Multiple micronutrient supplements in pregnancy: Implementation considerations for integration as part of quality services in routine antenatal care. Objectives, results, and conclusions of the meeting. Matern Child Nutr [Internet]. 2018 Dec;14:e12704. Available from: https://onlinelibrary.wiley.com/doi/10.1111/mcn.12704

45. Zahroh RI, Hazfiarini A, Eddy KE, Vogel JP, Tunçalp Özge, Minckas N, et al. Factors influencing appropriate use of interventions for management of women experiencing preterm birth: A mixed-methods systematic review and narrative synthesis. Stock SJ, editor. PLOS Med [Internet]. 2022 Aug 23;19(8):e1004074. Available from: https://dx.plos.org/10.1371/journal.pmed.1004074

46. (EFSA) EFSA. Scientific Opinion on Dietary Reference Values for calcium. EFSA J. 2015;13(5):1–82.

47. Betrán AP, Bergel E, Griffin S, Melo A, Nguyen MH, Carbonell A, et al. Provision of medical supply kits to improve quality of antenatal care in Mozambique: a stepped-wedge cluster randomised trial. Lancet Glob Heal [Internet]. 2018 Jan;6(1):e57–65. Available from: https://linkinghub.elsevier.com/retrieve/pii/S2214109X17304217

Figure 1: PRISMA Flow Diagram

Figure 1 Legend: PRISMA flow chart illustrating the number of records included and excluded at various screening and reviewing steps, leading to final list of records for data extraction and meta-analysis.

Figure 2: Capability, Opportunity and Motivation Model of Behaviour

Figure 2 Legend: Categorisation of barriers and facilitators across the Capability, Opportunity and Motivation model of Behaviour

For peer review only

For peer review only

For peer review only

For peer review only - http://bmjopen.bmj.com/site/about/guidelines.xhtml

PRISMA 2020 flow diagram for new systematic reviews which included searches of databases, registers, and other sources

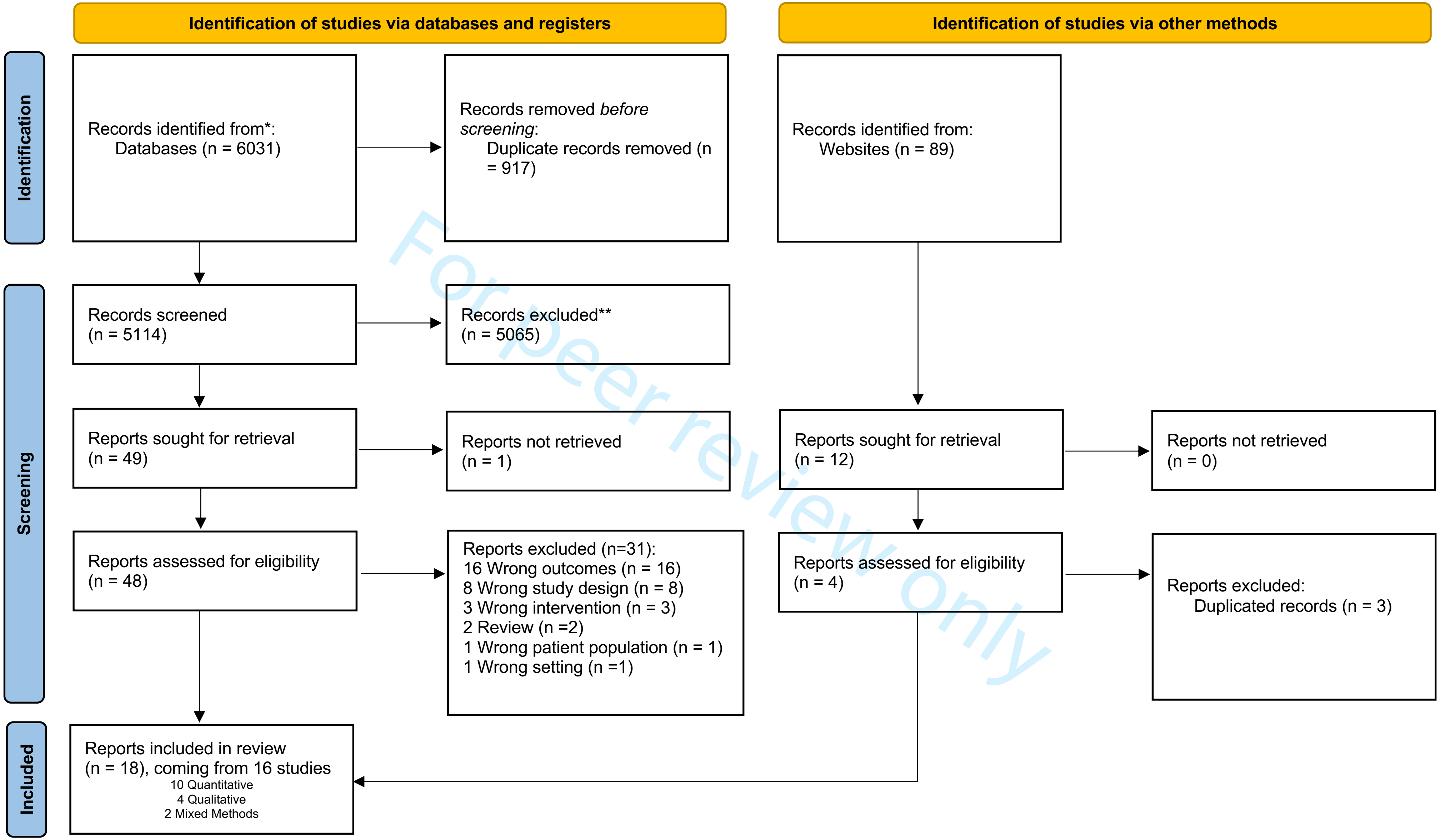

For peer review only

*Consider, if feasible to do so, reporting the number of records identified from each database or register searched (rather than the total number across all databases/registers).
**If automation tools were used, indicate how many records were excluded by a human and how many were excluded by automation tools.

From: Page MJ, McKenzie JE, Bossuyt PM, Boutron I, Hoffmann TC, Mulrow CD, et al. The PRISMA 2020 statement: an updated guideline for reporting systematic reviews. BMJ 2021;372:n71. doi: 10.1136/bmj.n71. For more information, visit: http://www.prisma-statement.org/

Capability in using interventions

Barriers

- Limited knowledge about preeclampsia/eclampsia†
- Symptoms were believed to be linked to evil attacks or nutritional deficiencies†
- Inadequate information on preeclampsia/eclampsia and calcium from providers†
- Varied knowledge on pre-eclampsia/eclampsia ¥
- In continuity and lack of depth training about pre-eclampsia/eclampsia ¥
- Felt inadequately trained ¥

Facilitators

- High knowledge of calcium supplementation†
- High education of women†
- Receive adequate information about condition and calcium from their providers†
- Counselling on information about preeclampsia and calcium from their providers†
- Continuous training to manage the condition and address resistance from community ¥
- Regular supervision visits for troubleshooting ¥

Motivation in using interventions

Barriers

- Pre-eclampsia/eclampsia was not considered as a serious problem†
- Experiences and fears of side effects†
- Safety of calcium was not assured†
- Felt that 3-4 pills per day at multiple times were too many†
- Inconvenience in taking pill daily†
- Perceived over-medicalization†¥
- Belief that preeclampsia/eclampsia was not a priority health concern ¥
- Fears in generating anxiety on low-risk women ¥

Facilitators

- Assurance of calcium safety†
- Experienced and expected benefits from taking calcium and folic acid†
- Valued information, education and communication (IEC) materials†
- Positive perceptions on tablet characteristics (smell, size, taste) †
- Belief that one combined pill per day could ease burden†
- Belief that having information would help making informed decisions†
- Belief that providers is a reliable source of information†
- Belief of being able to consume calcium daily†
- Feeling confident taking calcium after counselling provider†
- Positive belief about calcium supplementation benefits¥
- Belief that women should receive information regardless risk ¥

Opportunity in using interventions

Barriers

- Discouragement by family, neighbors and community in taking calcium†
- Stigmatization of having supplements and posters with HIV†
- Conflicting daily routine with taking calcium†
- Food insecurity†
- Providers felt that providing calcium would increase workload ¥
- Inadequate number of staff providing care ¥
- Stock out of supplements ¥
- Lack of equipment to diagnose ¥

Facilitators

- Family support in consuming calcium†
- Positive belief and experiences about 'adherence partners' †¥
- Early initiation and frequent antenatal visits †
- Universal free calcium distribution through antenatal care †
- Reminder tools distribution, such posters and pill-taking calendars †¥
- Adequate calcium supplement stock and its storage ¥
- Provision of equipment to diagnose pre-eclampsia ¥
- Adequate number of human resources at health facility ¥
- Comprehensive integrated program (job aids, training, guidelines, regular supplies) ¥

Behaviour: Calcium supplementation prescription by providers and use by women

†: woman's factors; ¥: providers factors

For peer review only - http://bmjopen.bmj.com/site/about/guidelines.xhtml

Appendix 1. PRISMA 2020 Main Checklist

Topic	No.	Item	Location where item is reported
**TITLE**			
**Title**	1	Identify the report as a systematic review.	Title
**ABSTRACT**			
**Abstract**	2	See the PRISMA 2020 for Abstracts checklist	Abstract
**INTRODUCTION**			
**Rationale**	3	Describe the rationale for the review in the context of existing knowledge.	Introduction
**Objectives**	4	Provide an explicit statement of the objective(s) or question(s) the review addresses.	Introduction
**METHODS**			
**Eligibility criteria**	5	Specify the inclusion and exclusion criteria for the review and how studies were grouped for the syntheses.	Methods –Topic of interest and type of studies
**Information sources**	6	Specify all databases, registers, websites, organisations, reference lists and other sources searched or consulted to identify studies. Specify the date when each source was last searched or consulted.	Methods – Search methods for identification of studies
**Search strategy**	7	Present the full search strategies for all databases, registers and websites, including any filters and limits used.	Appendix 3
**Selection process**	8	Specify the methods used to decide whether a study met the inclusion criteria of the review, including how many reviewers screened each record and each report retrieved, whether they	Methods – Selection of studies

For peer review only - http://bmjopen.bmj.com/site/about/guidelines.xhtml

		worked independently, and if applicable, details of automation tools used in the process.	
**Data collection process**	9	Specify the methods used to collect data from reports, including how many reviewers collected data from each report, whether they worked independently, any processes for obtaining or confirming data from study investigators, and if applicable, details of automation tools used in the process.	Methods – Data extraction and assessing the methodological limitations
**Data items**	10a	List and define all outcomes for which data were sought. Specify whether all results that were compatible with each outcome domain in each study were sought (e.g. for all measures, time points, analyses), and if not, the methods used to decide which results to collect.	Methods – Data extraction and assessing the methodological limitations
	10b	List and define all other variables for which data were sought (e.g. participant and intervention characteristics, funding sources). Describe any assumptions made about any missing or unclear information.	Methods – Data extraction and assessing the methodological limitations
**Study risk of bias assessment**	11	Specify the methods used to assess risk of bias in the included studies, including details of the tool(s) used, how many reviewers assessed each study and whether they worked independently, and if applicable, details of automation tools used in the process.	Methods – Data extraction and assessing the methodological limitations
**Effect measures**	12	Specify for each outcome the effect measure(s) (e.g. risk ratio, mean difference) used in the synthesis or presentation of results.	Not applicable
**Synthesis methods**	13a	Describe the processes used to decide which studies were eligible for each synthesis (e.g. tabulating the study intervention characteristics and comparing against the planned groups for each synthesis (item 5)).	Not applicable
	13b	Describe any methods required to prepare the data for presentation or synthesis, such as handling of missing summary statistics, or data conversions.	Not applicable
	13c	Describe any methods used to tabulate or visually display results of individual studies and syntheses.	Methods – Quality appraisal, analysis and assessing confidence

For peer review only - http://bmjopen.bmj.com/site/about/guidelines.xhtml

	13d	Describe any methods used to synthesize results and provide a rationale for the choice(s). If meta-analysis was performed, describe the model(s), method(s) to identify the presence and extent of statistical heterogeneity, and software package(s) used.	Methods – Quality appraisal, analysis and assessing confidence
	13e	Describe any methods used to explore possible causes of heterogeneity among study results (e.g. subgroup analysis, meta-regression).	Not Applicable
	13f	Describe any sensitivity analyses conducted to assess robustness of the synthesized results.	Not Applicable
**Reporting bias assessment**	14	Describe any methods used to assess risk of bias due to missing results in a synthesis (arising from reporting biases).	Methods – Quality appraisal, analysis and assessing confidence
**Certainty assessment**	15	Describe any methods used to assess certainty (or confidence) in the body of evidence for an outcome.	Methods – Quality appraisal, analysis and assessing confidence
**RESULTS**			
**Study selection**	16a	Describe the results of the search and selection process, from the number of records identified in the search to the number of studies included in the review, ideally using a flow diagram.	Results and Figure 1. PRISMA flowchart
	16b	Cite studies that might appear to meet the inclusion criteria, but which were excluded, and explain why they were excluded.	PRISMA flowchart
**Study characteristics**	17	Cite each included study and present its characteristics.	Results and Table 1
**Risk of bias in studies**	18	Present assessments of risk of bias for each included study.	Results
**Results of individual studies**	19	For all outcomes, present, for each study: (a) summary statistics for each group (where appropriate) and (b) an effect estimate and its	Appendix 5 and 6

For peer review only - http://bmjopen.bmj.com/site/about/guidelines.xhtml

		precision (e.g. confidence/credible interval), ideally using structured tables or plots.	
**Results of syntheses**	20a	For each synthesis, briefly summarise the characteristics and risk of bias among contributing studies.	Results. Summary of qualitative findings and GRADE-CERQual Evidence Profile
	20b	Present results of all statistical syntheses conducted. If meta-analysis was done, present for each the summary estimate and its precision (e.g. confidence/credible interval) and measures of statistical heterogeneity. If comparing groups, describe the direction of the effect.	Not applicable
	20c	Present results of all investigations of possible causes of heterogeneity among study results.	Not applicable
	20d	Present results of all sensitivity analyses conducted to assess the robustness of the synthesized results.	Not applicable
**Reporting biases**	21	Present assessments of risk of bias due to missing results (arising from reporting biases) for each synthesis assessed.	Not applicable
**Certainty of evidence**	22	Present assessments of certainty (or confidence) in the body of evidence for each outcome assessed.	Results– Table 2: Summary of qualitative findings and GRADE-CERQual Evidence Profile
**DISCUSSION**			
**Discussion**	23a	Provide a general interpretation of the results in the context of other evidence.	Discussions, Interpretation
	23b	Discuss any limitations of the evidence included in the review.	Discussions, Strengths and limitations
	23c	Discuss any limitations of the review processes used.	Discussions, Strengths and limitations
	23d	Discuss implications of the results for practice, policy, and future research.	Discussions, Implications for practice and conclusions

For peer review only - http://bmjopen.bmj.com/site/about/guidelines.xhtml

OTHER INFORMATION			
**Registration and protocol**	24a	Provide registration information for the review, including register name and registration number, or state that the review was not registered.	Methods
	24b	Indicate where the review protocol can be accessed, or state that a protocol was not prepared.	Methods
	24c	Describe and explain any amendments to information provided at registration or in the protocol.	Methods
**Support**	25	Describe sources of financial or non-financial support for the review, and the role of the funders or sponsors in the review.	Sources of funding
**Competing interests**	26	Declare any competing interests of review authors.	Competing interests
**Availability of data, code and other materials**	27	Report which of the following are publicly available and where they can be found: template data collection forms; data extracted from included studies; data used for all analyses; analytic code; any other materials used in the review.	Appendix 5 and 6

For peer review only

For peer review only - http://bmjopen.bmj.com/site/about/guidelines.xhtml

Appendix 1.1. PRISMA Abstract Checklist

Topic	No.	Item	Reported?
**TITLE**			
**Title**	1	Identify the report as a systematic review.	Yes
**INTRODUCTION**			
**Objectives**	2	Provide an explicit statement of the main objective(s) or question(s) the review addresses.	Yes
**METHODS**			
**Eligibility criteria**	3	Specify the inclusion and exclusion criteria for the review.	Yes
**Information sources**	4	Specify the information sources (e.g. databases, registers) used to identify studies and the date when each was last searched.	Yes
**Risk of bias**	5	Specify the methods used to assess risk of bias in the included studies.	Yes
**Synthesis of results**	6	Specify the methods used to present and synthesize results.	Yes
**RESULTS**			
**Included studies**	7	Give the total number of included studies and participants and summarise relevant characteristics of studies.	Yes
**Synthesis of results**	8	Present results for main outcomes, preferably indicating the number of included studies and participants for each. If meta-analysis was done, report the summary estimate and confidence/credible interval. If comparing groups, indicate the direction of the effect (i.e. which group is favoured).	Yes
**DISCUSSION**			

For peer review only - http://bmjopen.bmj.com/site/about/guidelines.xhtml

**Limitations of evidence**	9	Provide a brief summary of the limitations of the evidence included in the review (e.g. study risk of bias, inconsistency and imprecision).	Yes
**Interpretation**	10	Provide a general interpretation of the results and important implications.	Yes
**OTHER**			
**Funding**	11	Specify the primary source of funding for the review.	Yes
**Registration**	12	Provide the register name and registration number.	Yes

From: Page MJ, McKenzie JE, Bossuyt PM, Boutron I, Hoffmann TC, Mulrow CD, et al. The PRISMA 2020 statement: an updated guideline for reporting systematic reviews. MetaArXiv. 2020, September 14. DOI: 10.31222/osf.io/v7gm2. For more information, visit: www.prisma-statement.org

For peer review only

Appendix 2. Enhancing transparency in reporting the synthesis of qualitative research: ENTREQ Checklist (Tong, et al., 2012)

Item No.	Guide and Description	Report Location
1. Aim	State the research question the synthesis addresses	Introduction
2. Synthesis methodology	Identify the synthesis methodology or theoretical framework which underpins the synthesis, and describe the rationale for choice of methodology (e.g. meta-ethnography, thematic synthesis, critical interpretive synthesis, grounded theory synthesis, realist synthesis, meta-aggregation, meta-study, framework synthesis)	Methods – _ Data extraction and assessing the methodological limitations
3. Approach to searching	Indicate whether the search was pre-planned (comprehensive search strategies to seek all available studies) or iterative (to seek all available concepts until they theoretical saturation is achieved)	Methods – Search methods for identification of studies
4. Inclusion criteria	Specify the inclusion/exclusion criteria (e.g. in terms of population, language, year limits, type of publication, study type)	Methods – Selection of studies
5. Data sources	Describe the information sources used (e.g. electronic databases (MEDLINE, EMBASE, CINAHL, psycINFO), grey literature databases (digital thesis, policy reports), relevant organisational websites, experts, information specialists, generic web searches (Google Scholar) hand searching, reference lists) and when the searches conducted; provide the rationale for using the data sources	Methods - Search methods for identification of studies
6. Electronic Search strategy	Describe the literature search (e.g. provide electronic search strategies with population terms, clinical or health topic terms, experiential or social phenomena related terms, filters for qualitative research, and search limits)	Appendix 3
7. Study screening methods	Describe the process of study screening and sifting (e.g. title, abstract and full text review, number of independent reviewers who screened studies)	Methods - Search methods for identification of studies
8. Study characteristics	Present the characteristics of the included studies (e.g. year of publication, country, population, number of participants, data collection, methodology, analysis, research questions)	Table 1 - Characteristics of included studies
9. Study selection results	Identify the number of studies screened and provide reasons for study exclusion (e.g. for comprehensive searching, provide numbers of studies screened and reasons for exclusion indicated in a figure/flowchart; for iterative searching describe reasons for study exclusion	Fig 1 - PRISMA flow diagram

For peer review only

	and inclusion based on modifications to the research question and/or contribution to theory development)	
10. Rationale for appraisal	Describe the rationale and approach used to appraise the included studies or selected findings (e.g. assessment of conduct (validity and robustness), assessment of reporting (transparency), assessment of content and utility of the findings)	Methods - Quality appraisal, analysis and assessing confidence and Appendix 4
11. Appraisal items	State the tools, frameworks and criteria used to appraise the studies or selected findings (e.g. Existing tools: CASP, QARI, COREQ, Mays and Pope [25]; reviewer developed tools; describe the domains assessed: research team, study design, data analysis and interpretations, reporting)	Methods - Data extraction and assessing the methodological limitations
12. Appraisal process	Indicate whether the appraisal was conducted independently by more than one reviewer and if consensus was required	Methods - Data extraction and assessing the methodological limitations
13. Appraisal results	Present results of the quality assessment and indicate which articles, if any, were weighted/excluded based on the assessment and give the rationale	Appendix 5
14. Data extraction	Indicate which sections of the primary studies were analysed and how were the data extracted from the primary studies? (e.g. all text under the headings "results /conclusions" were extracted electronically and entered into a computer software)	Methods- Data extraction and assessing the methodological limitations
15. Software	State the computer software used, if any	None
16. Number of reviewers	Identify who was involved in coding and analysis	Methods - Data extraction and assessing the methodological limitations
17. Coding	Describe the process for coding of data (e.g. line by line coding to search for concepts)	Methods - Quality appraisal, analysis and assessing confidence
18. Study comparison	Describe how were comparisons made within and across studies (e.g. subsequent studies were coded into pre-existing concepts, and new concepts were created when deemed necessary)	Methods - Quality appraisal, analysis and assessing confidence

For peer review only

19. Derivation of themes	Explain whether the process of deriving the themes or constructs was inductive or deductive	Methods - Quality appraisal, analysis and assessing confidence
20. Quotations	Provide quotations from the primary studies to illustrate themes/constructs, and identify whether the quotations were participant quotations of the author's interpretation	Results
21. Synthesis output	Present rich, compelling and useful results that go beyond a summary of the primary studies (e.g. new interpretation, models of evidence, conceptual models, analytical framework, development of a new theory or construct)	Discussion

For peer review only

Appendix 3. Search Strategy

Embase (inception to 2021 March 22)

1 exp Calcium/ or Calcium Carbonate/ (305629)
2 (calcium adj3 supplement*).mp. (9456)
3 1 or 2 (307747)
4 Pregnant Women/ or Prenatal Care/ (109074)
5 (pregnan* or prenatal).mp. (1086333)
6 4 or 5 (1086333)
7 3 and 6 (8440)
8 limit 7 to humans (5885)
9 limit 8 to (amphibia or ape or bird or cat or cattle or chicken or dog or "ducks and geese" or fish or "frogs and toads" or goat or guinea pig or "hamsters and gerbils" or horse or monkey or mouse or "pigeons and doves" or "rabbits and hares" or rat or reptile or sheep or swine) (277)
10 8 not 9 (5608)
11 limit 10 to (conference abstract or conference paper or "conference review" or editorial or erratum or letter or note or "review") (2567)
12 10 not 11 (**3041**)

Embase (inception to 2022 August 16) – Search update

1 exp Calcium/ or Calcium Carbonate/ (323807)
2 (calcium adj3 supplement*).mp. (10017)
3 1 or 2 (326035)
4 Pregnant Women/ or Prenatal Care/ (125455)
5 (pregnan* or prenatal).mp. (1161704)
6 4 or 5 (1161704)
7 3 and 6 (9061)
8 limit 7 to humans (6413)
9 limit 8 to (amphibia or ape or bird or cat or cattle or chicken or dog or "ducks and geese" or fish or "frogs and toads" or goat or guinea pig or "hamsters and gerbils" or horse or monkey or mouse or "pigeons and doves" or "rabbits and hares" or rat or reptile or sheep or swine) (303)
10 8 not 9 (6110)
11 limit 10 to (conference abstract or conference paper or "conference review" or editorial or erratum or letter or note or "review") (2744)
12 10 not 11 (3366)
13 limit 12 to yr="2022 - 2023" (**142**)

MEDLINE (1946 to March Week 2 2021)

1 exp Calcium/ or Calcium Carbonate/ (277850)
2 (calcium adj3 supplement*).mp. (5560)

For peer review only

3 1 or 2 (280742)
4 Pregnant Women/ or Prenatal Care/ (36709)
5 (pregnan* or prenatal).mp. (999134)
6 4 or 5 (999134)
7 3 and 6 (6417)
8 limit 7 to humans (3417)
9 limit 8 to animals (618)
10 8 not 9 (2799)
11 limit 10 to ("review articles" and case reports) (17)
12 10 not 11 (**2782 results**)

MEDLINE (inception to August Week 1, 2022) – Search update

1 exp Calcium/ or Calcium Carbonate/ (286238)
2 (calcium adj3 supplement*).mp. (5967)
3 1 or 2 (289314)
4 Pregnant Women/ or Prenatal Care/ (42373)
5 (pregnan* or prenatal).mp. (1068999)
6 4 or 5 (1068999)
7 3 and 6 6605
8 limit 7 to humans (3545)
9 limit 8 to animals (634)
10 8 not 9 (2911)
11 limit 10 to ("review articles" and case reports) (18)
12 10 not 11 (2893)
13 limit 12 to yr="2021 - 2022" (**89**)

CINAHL

CINAHL (inception to March 2021)

(calcium and supplement*) AND (pregnan* or prenatal) AND (preeclamp* or pre-eclamp* or eclamp* or "gestational hypertension" or "maternal hypertension") (**132**)

CINAHL (March 2021 to August 2022) – Search update

(calcium and supplement*) AND (pregnan* or prenatal) AND (preeclamp* or pre-eclamp* or eclamp* or "gestational hypertension" or "maternal hypertension") (**1**)

GLOBAL HEALTH

GLOBAL HEALTH (inception to March 2021)

(calcium and supplement*) AND (pregnan* or prenatal) AND (preeclamp* or pre-eclamp* or eclamp* or "gestational hypertension" or "maternal hypertension") (**158 results**)

GLOBAL HEALTH (2021 to August 2022) – Search update

(calcium and supplement*) AND (pregnan* or prenatal) AND (preeclamp* or pre-eclamp* or eclamp* or "gestational hypertension" or "maternal hypertension") (**158 results**)

For peer review only - http://bmjopen.bmj.com/site/about/guidelines.xhtml

For peer review only

For peer review only - http://bmjopen.bmj.com/site/about/guidelines.xhtml

Appendix 4. Critical Appraisal Table

Qualitative studies

STUDY DETAIL	SCREENING QUESTIONS		1. QUALITATIVE STUDIES							MMAT RATING
**First author**	**S1. Are there clear research questions?**	**S2. Do the collected data allow to address the research questions?**	**1.1. Is the qualitative approach appropriate to answer the research question? (Aim, appropriateness of a qualitative approach)**	**1.2. Are the qualitative data collection methods adequate to address the research question? (recruitment, data collection)**	**1.3. Are the findings adequately derived from the data? (rigor in analysis)**	**1.4. Is the interpretation of results sufficiently substantiated by data? (link from data to findings)**	**1.5. Is there coherence between qualitative data sources, collection, analysis and interpretation? (overall design from start to finish)**	**1.6. Have ethical issues been taken into consideration? (consent, confidentiality, ethics approval)**	**1.7. Is relationship between researcher and participants adequately considered? (interaction and reflection on how research team influences design & implementation)**	
**Vestering 2019**	Yes	Yes	Yes	Yes	Yes	Yes	Yes	Partial	Partial	"Moderate" (minor issues impacting credibility/validity)
**Birhanu 2018**	Yes	Yes	Yes	Yes	Yes	Yes	Yes	Yes	Unclear	"Moderate" (minor issues impacting credibility/validity)
**Martin 2017a**	Yes	Yes	Yes	Yes	Yes	Yes	Yes	Yes	Unclear	"Moderate" (minor issues impacting credibility/validity)
**Martin 2018**	Yes	Yes	Yes	Yes	Yes	Yes	Yes	Yes	Partial	"Moderate" (minor issues impacting credibility/validity)

For peer review only - http://bmjopen.bmj.com/site/about/guidelines.xhtml

Quantitative studies

STUDY DETAIL	SCREENING QUESTIONS		3. NON-RANDOMIZED STUDIES					4. QUANTITATIVE DESCRIPTIVE STUDIES					MMAT RATING
**First author**	**S1. Are there clear research questions?**	**S2. Do the collected data allow to address the research questions?**	**3.1. Are the participants representative of the target population?**	**3.2. Are measurements appropriate regarding both the outcome and intervention (or exposure)?**	**3.3. Are there complete outcome data?**	**3.4. Are the confounders accounted for in the design and analysis?**	**3.5. During the study period, is the intervention administered (or exposure occurred) as intended?**	**4.1. Is the sampling strategy relevant to address the research question?**	**4.2. Is the sample representative of the target population?**	**4.3. Are the measurements appropriate?**	**4.4. Is the risk of nonresponse bias low?**	**4.5. Is the statistical analysis appropriate to answer the research question?**	
Baxter 2014	Yes	Yes						Yes	Unclear	Yes	No	Yes	"Low" (some issues likely to impact credibility/validity)
Thapa 2016	Yes	Yes	Yes	Yes	Yes	Yes	Yes						"High" (no or very minor significant issues)
Nguyen 2019	Yes	Yes						Yes	Yes	Yes	Unclear	Yes	"Moderate" (minor issues impacting credibility/validity)
Omotayo 2018a & Martin 2017b	Yes	Yes	Yes	Yes	Partial	Yes	Yes						"Moderate" (minor issues impacting credibility/validity)
Nguyen 2017	Yes	Yes						Yes	Yes	Yes	Yes	Yes	"High" (no or very minor significant issues)
Nguyen 2018	Yes	Yes						Yes	Yes	Yes	Yes	Yes	"High" (no or very minor significant issues)

Liu 2019	Yes	Yes						Yes	Yes	Partial	No	Yes	"Low" (some issues likely to impact credibility/validity)
Shakya Shrestha 2020	Yes	Yes						Partial	Unclear	Partial	Yes	Partial	"Very low" (significant issues impacting credibility/validity)
Ghosh-Jerath 2015	Yes	Yes						Yes	Yes	Partial	No	Yes	"Low" (some issues likely to impact credibility/validity)

*Grey shades or empty cells refer to not applicable.

Mixed methods studies

STUDY DETAIL	First author	Omotayo 2018b & Martin 2017c	Kachwaha 2022
SCREENING QUESTIONS	S1. Are there clear research questions?	Yes	Yes
	S2. Do the collected data allow to address the research questions?	Yes	Yes
1. QUALITATIVE STUDIES	1.1. Is the qualitative approach appropriate to answer the research question? (Aim, appropriateness of a qualitative approach)	Yes	Yes
	1.2. Are the qualitative data collection methods adequate to address the research question? (recruitment, data collection)	Yes	Yes
	1.3. Are the findings adequately derived from the data? (rigor in analysis)	Yes	Yes
	1.4. Is the interpretation of results sufficiently substantiated by data? (link from data to findings)	Yes	Yes
	1.5. Is there coherence between qualitative data sources, collection, analysis and interpretation? (overall design from start to finish)	Yes	Yes
	1.6. Have ethical issues been taken into consideration? (consent, confidentiality, ethics approval)	Yes	Yes
	1.7. Is relationship between researcher and participants adequately considered? (interaction and reflection on how research team influences design & implementation)	Unclear	Unclear
	4.1. Is the sampling strategy relevant to address the research question?	Yes	Yes

For peer review only - http://bmjopen.bmj.com/site/about/guidelines.xhtml

**4. QUANTITATIVE DESCRIPTIVE STUDIES**	**4.2. Is the sample representative of the target population?**	Yes	Yes
	**4.3. Are the measurements appropriate?**	Yes	Yes
	**4.4. Is the risk of nonresponse bias low?**	Yes	Partial
	**4.5. Is the statistical analysis appropriate to answer the research question?**	Yes	Yes
**5. MIXED METHODS STUDIES**	**5.1. Is there an adequate rationale for using a mixed methods design to address the research question?**	Yes	Yes
	**5.2. Are the different components of the study effectively integrated to answer the research question?**	Yes	Yes
	**5.3. Are the outputs of the integration of qualitative and quantitative components adequately interpreted?**	Yes	Yes
	**5.4. Are divergences and inconsistencies between quantitative and qualitative results adequately addressed?**	Yes	Yes
	**5.5. Do the different components of the study adhere to the quality criteria of each tradition of the methods involved?**	Yes	Partial
**MMAT RATING**		"High" (no or very minor significant issues)	"Moderate" (minor issues impacting credibility/validity)

For peer review only

Appendix 5. **Evidence profile**

No	Summary of qualitative review findings	Contributing qualitative studies	Methodological limitations assessment	Coherence assessment	Relevance assessment	Adequacy assessment	Overall CERQual assessment	Explanation of overall assessment
**Women's factors**								
**1. Women's knowledge and learning**								
**1.1**	**Women's knowledge about pre-eclampsia or eclampsia** Most women had limited knowledge about pre-eclampsia or eclampsia, and these conditions were not typically considered by women to be a serious problem. Symptoms of pre-eclampsia such as swollen feet, severe headache, blurred vision and vomiting were considered normal signs and symptoms in pregnancy, while seizures were associated with evil attacks or nutritional deficiencies.	(Martin 2017b, Birhanu 2018, Vestering 2019)	**Minor concerns:** Three studies with minor issues (reflexivity and ethics).	**No or very minor concerns**	**Moderate concerns:** One study is indirectly relevant to review aim and represented 3 countries (Kenya, Ethiopia, Netherlands), including 1 low-income, 1 lower middle income and 1 high income country. All perspectives came from health providers and women.	**Minor concerns:** 3 out of 6 studies contributed to review finding (2 thick data, 1 thin data).	**High confidence**	Due to no or very minor concerns on coherence, minor concerns on methodological limitations (reflexivity and ethics), moderate concerns on relevance (1 out of 3 studies are indirectly relevant to our review aim and small number of countries), and minor concerns of adequacy (3 out of 6 contributed with 2 thick and 1 thin data).
**1.2**	**Information provision to women** Women felt they did not receive adequate information	(Vestering 2019, Martin 2018)	**Minor concerns:** Two studies with minor	**Moderate concerns:** No good understanding	**Moderate concerns:** All studies are directly relevant	**Serious concerns:** 2 out of 6 studies	**Low confidence**	Minor concerns on methodological limitations (ethics and reflexivity), moderate concerns on coherence (no clear

Formatted Table

Formatted Table

For peer review only

No	Summary of qualitative review findings	Contributing qualitative studies	Methodological limitations assessment	Coherence assessment	Relevance assessment	Adequacy assessment	Overall CERQual assessment	Explanation of overall assessment
	during pregnancy from healthcare providers about pre-eclampsia or eclampsia and calcium supplements. They described wanting to be given more information, regardless of their pre-eclampsia risk status. Women believed that having this essential information could help them to make informed choices and actively participate in their care. There were, however, mixed opinions from healthcare providers - some feared that more information could generate anxiety for women, while others were more supportive of providing information.		issues (ethics and reflexivity).	why some providers worry in generating anxiety to women while others are not.	to review aim and represented 2 countries (Kenya, Netherlands), including 1 lower middle income and 1 high income country. All perspectives came from health providers and women.	contributed to review finding (1 thick data, 1 thin data).		understanding on why some providers worry in generating anxiety to women while the others are not), moderate concerns on relevance (small number of countries), and serious concerns on adequacy (2 out of 6 contributed with 1 thick and 1 thin data).
1.3	**Learning about calcium supplements** Women typically learned about dietary calcium, including pre-eclampsia and eclampsia symptoms, from healthcare providers. They	(Vestering 2019, Martin 2018, Birhanu 2018)	**Minor concerns:** Three studies with minor issues (reflexivity and ethics).	**No or very minor concerns**	**Minor concerns:** All studies are directly relevant to review aim and represented 3	**Minor concerns:** 3 out of 6 studies contributed to findings (2 with moderate-thick	**High confidence**	Due to no or very minor concerns on coherence, minor concerns on relevance (small number of countries), minor concerns on methodological limitations (reflexivity and ethics), and minor concerns on adequacy (3 out of 6

Formatted Table

No	Summary of qualitative review findings	Contributing qualitative studies	Methodological limitations assessment	Coherence assessment	Relevance assessment	Adequacy assessment	Overall CERQual assessment	Explanation of overall assessment
	considered healthcare providers to be the most trusted and reliable source of information, and reported feeling confident about taking calcium-containing supplements after receiving adequate information from them. Women also appreciated receiving information on calcium supplements and pre-eclampsia or eclampsia via information, education, and communication (IEC) materials like videos, media, and trusted websites.				countries (Kenya, Ethiopia, Netherlands), including 1 low-income, 1 lower middle income and 1 high income country. All perspectives came from health providers and women.	data and 1 with thin data.		contributed, 1 moderate thick and 2 thin data).
**2. Women's beliefs about calcium supplements in pregnancy**								
**2.1**	**Fears about side-effects as a barrier to calcium supplements uptake** Women's fears about the side effects of calcium supplements affected their adherence. Women highlighted that assurance of safe use of calcium supplement is a key facilitator to consistent use. However,	(Omotayo 2017, Birhanu 2018, Vestering 2019, Martin 2018)	**Minor concerns:** Three out of four studies have minor issues (ethics and reflexivity) and one study with no or very	**No or very minor concerns**	**Minor concerns:** All studies are directly relevant to review aim and represented 3 countries (Kenya, Ethiopia, Netherlands),	**Minor concerns** Overall, small number of studies contributing to the qualitative evidence synthesis.	**High Confidence**	Due to no or very minor conc[illegible] on coherence, minor concerns on relevance (small number of countries), minor concerns on adequacy (small number of studies contributing to the qualitative evidence synthesis), and minor concerns on methodological limitations (reflexivity and ethics).

Formatted Table

Formatted Table

For peer review only

No	Summary of qualitative review findings	Contributing qualitative studies	Methodological limitations assessment	Coherence assessment	Relevance assessment	Adequacy assessment	Overall CERQual assessment	Explanation of overall assessment
	some women felt safety was not assured by healthcare providers, especially when calcium supplements were perceived as "experimental". Women had also received messages from their families or communities that any pills consumed during pregnancy could be harmful.		minor issues.		including 1 low-income, 1 lower middle income and 1 high income country. All perspectives came from women.			
2.2	**Women's experiences of side-effects** Some women reported experiencing side effects after taking calcium and iron-folic acid supplements, such as dizziness, vomiting, nausea, stomach aches, loss of appetite, tiredness, diarrhoea, bloating, and burping, yet noted that side effects subsided with time. Women also reported that they continued consuming calcium despite these side effects.	(Omotayo 2017, Martin 2018, Birhanu 2018)	**Minor concerns:** Two out of three studies have minor issues (ethics and reflexivity) and one study with no or very minor issues	**No or very minor concerns**	**Moderate concerns:** All studies are directly relevant to review aim and represented 2 countries (Kenya, Ethiopia), including 1 low-income and 1 lower middle-income country. All perspectives came from health providers and women.	**Minor concerns:** 3 out of 6 studies contributed to the findings (2 thin and 1 with thick data).	**High Confidence**	Due to no or very minor concerns on coherence, minor concerns on methodological limitations (ethics and reflexivity), moderate concerns on relevance (only low and lower middle-income country and small number of countries) and minor concerns on adequacy (3 out of 6 studies contributed with 2 thick and 1 with thin data).

Formatted Table

For peer review only

No	Summary of qualitative review findings	Contributing qualitative studies	Methodological limitations assessment	Coherence assessment	Relevance assessment	Adequacy assessment	Overall CERQual assessment	Explanation of overall assessment
2.3	**Concerns about being stigmatized as HIV patients** Women expressed concerns that if they ingested calcium supplements, they could be stigmatized as HIV patients, which was a reported barrier to use. Some women were afraid of being stigmatized as their community often associated nutritional supplement consumption and accompanying reminder posters with HIV.	(Martin 2018, Vestering 2019)	**No or very minor concerns**	**No or very minor concerns**	**Moderate concerns:** All studies are directly relevant to review aim and represented 2 countries (Kenya, Netherlands), including 1 lower middle income and 1 high income country. All perspectives came from health providers and women.	**Serious concerns:** 2 out of 6 studies contributed to review finding (1 thick data and 1 thin data).	**Moderate Confidence**	Due to no or very minor concerns on methodological limitations, no or very minor concerns on coherence, minor concerns on relevance (small number of countries), and serious concerns on adequacy (2 out of 6 study contributed).
2.4	**Positive perceptions of calcium supplements** Women reported that both their perceptions about expected benefits and previous experiences of taking calcium and iron-folic acid supplements were facilitators of use. Women believed that consuming pills could	(Vestering 2019, Martin 2018, Martin 2017b, Birhanu 2018, Omotayo 2017)	**Minor concerns:** Four out of five studies have minor issues (ethics and reflexivity) and one study with	**No or very minor concerns**	**Minor concerns:** One study is indirectly relevant to review aim and represented 3 countries (Kenya, Ethiopia,	**Minor concerns** Overall, small number of studies contributing to the qualitative evidence synthesis.	**High Confidence**	Due to no or very minor concerns on coherence, minor concerns on methodological limitations (ethics and reflexivity), minor concerns on relevance (1 study indirectly relevant to review aim and small number of countries) and adequacy (small number of studies contributing to the qualitative evidence synthesis).

Formatted Table

For peer review only

No	Summary of qualitative review findings	Contributing qualitative studies	Methodological limitations assessment	Coherence assessment	Relevance assessment	Adequacy assessment	Overall CERQual assessment	Explanation of overall assessment
	compensate for sub-optimal nutrition during pregnancy, and that supplements during pregnancy would help keep their baby safe.		no or very minor issues.		Netherlands), including 1 low-income, 1 lower middle income and 1 high income country. All perspectives came from health providers and women.			
**3. Calcium supplement characteristics and regimens**								
**3.1**	**Varying preferences about characteristics of calcium tablets** Positive perceptions about the characteristics of the calcium tablet played a role in motivating women to take it. Some women preferred the chewable, sweet-tasting tablets that could be swallowed without water, while others preferred the hard tablets which were smaller in size, had no smell, and needed to be taken with water. Based on individual preference, the taste, smell, size, and convenience	(Omotayo 2017, Birhanu 2018)	**Minor concerns:** One study has minor issues (reflexivity) and one study has no or very minor issues.	**No or very minor concerns**	**Moderate concerns:** All studies are directly relevant to review aim and represented 2 countries (Kenya, Ethiopia), including 1 low-income and 1 lower middle-income country. All perspectives came from women.	**Moderate concerns:** 2 out of 6 studies contributed to findings and both had moderate to thick data.	**Moderate confidence**	Due to no or very minor concerns on coherence, minor concerns on methodological limitations (reflexivity), moderate concerns on relevance (all low or lower middle-income countries and small number of countries) and moderate concerns on adequacy (2 out of 6 studies contributed with moderate to thick data).

Formatted Table

No	Summary of qualitative review findings	Contributing qualitative studies	Methodological limitations assessment	Coherence assessment	Relevance assessment	Adequacy assessment	Overall CERQual assessment	Explanation of overall assessment
	affected calcium supplement use.							
3.2	**Supplement regimen as a barrier to use** Women described that they feel overwhelmed with the number of calcium tablets they had to take each day, especially women with other comorbidities who needed to take additional medications for their health conditions. Women felt that 3-4 pills per day at multiple times was overly onerous and preferred if they were combined into one pill.	(Martin 2018, Birhanu 2018, Omotayo 2017)	**Minor concerns:** Two studies have minor issues (reflexivity) and one study has no or very minor issues.	**No or very minor concerns**	**Moderate concerns:** All studies are directly relevant to review aim and represented 2 countries (Kenya, Ethiopia), including 1 low-income and 1 lower middle-income country. All perspectives came from health providers and women.	**Minor concerns:** 3 out of 6 studies contributing to findings and all have moderate to thick data.	**High Confidence**	No or very minor concerns on coherence, minor concerns on methodological limitations (reflexivity), minor concerns on adequacy (3 out of 6 studies contributed with moderate to thick data), and moderate concerns on relevance (small number of countries).
**4. Daily routines and food insecurity**								
4.1	**Adherence challenges due to routines** Adherence to calcium supplements consumption was challenging for some women because of conflicting activities in their daily routine, such as consuming other	(Martin 2018, Birhanu 2018, Omotayo 2017)	**Minor concerns:** Two out of three studies have minor issues (reflexivity) and one	**No or very minor concerns**	**Moderate concerns:** All studies are directly relevant to review aim and represented 2 countries	**Minor concerns:** Overall, small number of studies contributing to qualitative	**High Confidence**	Due to no or very minor concerns with coherence, minor concerns on methodological limitations (reflexivity), moderate concerns on relevance (small number of countries) and moderate concerns on adequacy (small

Formatted Table

For peer review only

No	Summary of qualitative review findings	Contributing qualitative studies	Methodological limitations assessment	Coherence assessment	Relevance assessment	Adequacy assessment	Overall CERQual assessment	Explanation of overall assessment
	medications, traveling, being away from home, and household chores, which can lead them to forget to take calcium.		study with no or very minor issues.		(Kenya, Ethiopia), including 1 low-income and 1 lower middle-income country. All perspectives came from health providers and women.	evidence synthesis.		number of studies contributing to qualitative evidence synthesis).
4.2	**Food insecurity as a barrier to calcium uptake** Women believed that adequate food was necessary to be able to take the supplements, to avoid nausea. They perceived it as normal to eat before consuming any medication. However, women reported that food insecurity was a critical barrier to calcium uptake.	(Birhanu 2018, Martin 2018)	**Minor concerns:** Two studies with minor issues (reflexivity).	**No or very minor concerns**	**Moderate concerns:** All studies are directly relevant to review aim and represented 2 countries (Kenya, Ethiopia), including 1 low-income and 1 lower middle-income country. All perspectives came from health providers and women.	**Moderate concerns:** 2 out of 6 studies contributed to findings with 1 thick and 1 thin data.	**Moderate Confidence**	Due to no or very minor concern with coherence, minor concerns on methodological limitations (reflexivity), moderate concerns on adequacy (2 out 6 studies contributed with 1 thick and 1 thin data), and moderate concerns on relevance (small number of countries).
**5. Strategies to improve the use of calcium supplements**								

Formatted Table

No	Summary of qualitative review findings	Contributing qualitative studies	Methodological limitations assessment	Coherence assessment	Relevance assessment	Adequacy assessment	Overall CERQual assessment	Explanation of overall assessment
5.1	**Implementation of reminders to promote adherence** Women and healthcare providers perceived reminders as beneficial in promoting women's adherence to consuming calcium supplements. Several reminder strategies were described as useful by women and healthcare providers, such as home-based posters, calendars with illustrations and daily reminders, and integrating supplement consumption into women's daily routine, such as mealtimes.	(Martin 2017a, Martin 2018, Birhanu 2018, Omotayo 2017)	**No or very minor concerns**	**No or very minor concerns**	**Moderate concerns:** One study indirectly relevant to review aim and represented 2 countries (Kenya, Ethiopia), including 1 low-income and 1 lower middle-income country. All perspectives came from health providers and women.	**Minor concerns:** Overall, small numbers of studies contributing to qualitative evidence synthesis.	**High Confidence**	Due to no or very minor concerns on methodological limitations, no or very minor concerns on coherence, minor concerns on adequacy (small number of studies contributing to qualitative evidence synthesis), moderate concerns on relevance (due to all studies coming from low income or lower middle-income country and small number of countries).
5.2	**Importance of family support and 'adherence partner' implementation** Having family support was instrumental to pregnant women adhering to calcium supplement use. This could be leveraged by notifying family members on the	(Martin 2018, Martin 2017b, Birhanu 2018, Martin 2017a, Omotayo 2017)	**No or very minor concerns**	**No or very minor concerns**	**Moderate concerns:** Two out of five studies indirectly relevant to review aim and represented 2 countries	**Minor concerns:** Overall, small number of studies contributing to qualitative evidence synthesis.	**High Confidence**	No or very minor concerns on methodological limitations, no or very minor concerns on coherence, minor concerns on adequacy (small number of studies contributing to qualitative evidence synthesis), and moderate concerns on relevance (due to all studies coming from

Formatted Table

No	Summary of qualitative review findings	Contributing qualitative studies	Methodological limitations assessment	Coherence assessment	Relevance assessment	Adequacy assessment	Overall CERQual assessment	Explanation of overall assessment
	importance of calcium, and appointing someone to be an "adherence partner" or "pill buddy" to help remind her to take it. Both women and healthcare providers were positive about adherence partners in providing support in terms of encouraging them to take the supplements, providing food, helping them around the house, providing emotional support, improving family relationships, and thereby increasing partner or husband involvement in pregnancy.				(Kenya, Ethiopia), including 1 low-income and 1 lower middle-income country. All perspectives came from health providers, partner and women.			low income or lower middle-income country only, 2 of 5 studies has indirectly relevant aim, and small number of countries).
5.3	**Counselling facilitates calcium supplements uptake** Both women and healthcare providers acknowledged that counselling women on the benefits of calcium was a motivator to calcium supplements uptake. Women valued the discussion they have with healthcare providers and felt more	(Birhanu 2018, Martin 2018)	**No or very minor concerns**	**No or very minor concerns**	**Moderate concerns:** All studies are directly relevant to review aim and represented 2 countries (Kenya, Ethiopia), including 1 low-income and 1	**Moderate concerns:** 2 out of 6 studies contributing to findings with all thick data.	**Moderate Confidence**	No or very minor concerns on methodological limitations, no or very minor concerns on coherence, moderate concerns on relevance (due to all studies coming from low or lower middle-income country and small number of countries) and moderate concerns on adequacy (2 out 6 studies contributed with thick data).

Formatted Table

No	Summary of qualitative review findings	Contributing qualitative studies	Methodological limitations assessment	Coherence assessment	Relevance assessment	Adequacy assessment	Overall CERQual assessment	Explanation of overall assessment
	confident to take calcium supplements when they received counselling and information and pre-eclampsia or eclampsia and the benefits of calcium from their healthcare providers.				lower middle-income country. All perspectives came from health providers and women.			
**Health care providers' factors**								
**6. Healthcare provider knowledge and training**								
6.1	**Varied knowledge about pre-eclampsia or eclampsia among healthcare providers** Healthcare providers' knowledge about pre-eclampsia and eclampsia was varied. Some felt that pre-eclampsia and eclampsia is not a priority health concern in their area and reported never having encountered any case.	(Birhanu 2018, Martin 2017a, Kachwaha 2022)	**No or very minor concerns**	**No or very minor concerns**	**Moderate concerns:** One study is indirectly relevant to review aim and represented 2 countries (Kenya, Ethiopia), including 1 low-income and 1 lower middle-income country. All perspectives came from health providers.	**Moderate concerns:** 2 out of 6 studies contributed to findings with thick data.	**Moderate Confidence**	Due to no or very minor concerns on methodological limitations and coherence, moderate concerns on relevance (due to all studies coming from low or lower middle-income countries and small number of studies) and moderate concerns on adequacy (2 out 6 studies contributed with thick data).

Formatted Table

For peer review only

No	Summary of qualitative review findings	Contributing qualitative studies	Methodological limitations assessment	Coherence assessment	Relevance assessment	Adequacy assessment	Overall CERQual assessment	Explanation of overall assessment
6.2	**Inadequate training to diagnose and treat pre-eclampsia and eclampsia** While some healthcare providers mentioned that training about pre-eclampsia or eclampsia and calcium supplements was adequate, others reported that their training lacked depth and continuity, and thus felt unprepared to diagnose it and offer information these conditions and the use of calcium for prevention. Healthcare providers expressed the need to have more and continuous training on managing pre-eclampsia, as well as time to address concerns or resistance from the community.	(Martin 2018, Birhanu 2018, Martin 2017a)	**No or very minor concerns**	**No or very minor concerns**	**Moderate concerns:** One study is indirectly relevant to review aim and represented 2 countries (Kenya, Ethiopia), including 1 low-income and 1 lower middle-income country. All perspectives came from health providers.	**Minor concerns:** 3 out of 6 studies contributed with 2 thick and 1 thin data.	**High Confidence**	No or very minor concerns on methodological limitations, coherence, moderate concerns on relevance (due to all studies coming from low income or lower middle-income country and small number of countries) and minor concerns on adequacy (3 out of 6 studies contributed with 2 thick and 1 thin data).
**7. Beliefs about the intervention**								
7.1	**Perceived overmedicalization when prescribing calcium supplements**	(Vestering 2019)	**Minor concerns:** One study with minor issues	**No or very minor concerns**	**Serious concerns:** One study is directly relevant to review aim	**Serious concerns:** 1 out of 6 study contributed to	**Low confidence**	No or very minor concerns on coherence, minor concerns on methodological limitations (reflexivity and ethics), serious concerns on relevance (evidence

Formatted Table

For peer review only

No	Summary of qualitative review findings	Contributing qualitative studies	Methodological limitations assessment	Coherence assessment	Relevance assessment	Adequacy assessment	Overall CERQual assessment	Explanation of overall assessment
	Both healthcare providers and women perceived that prescribing more tablets to "low-risk" women during pregnancy was a form of over-medicalization of pregnancy. However, some healthcare providers felt that calcium supplements were a way to prevent further medicalization due to pre-eclampsia-related complications.		(reflexivity and ethics).		and represented 1 country (Netherlands), which is high income country. All perspectives came from health providers and women.	findings with thick data.		coming from high income country only), serious concerns on adequacy (1 out of 6 study contributed).
2.2	**Beliefs about calcium supplements** Healthcare providers generally had positive beliefs about calcium supplements and there was optimism that calcium could be delivered through antenatal care healthcare providers. Some facilitators motivating healthcare providers to prescribe calcium supplements included their beliefs in its prevention value and expected benefits, that women liked the calcium	(Martin 2018, Vestering 2019, Birhanu 2018)	**Minor concerns:** Two studies with minor issues (ethics and reflexivity).	**No or very minor concerns**	**Minor concerns:** All studies are directly relevant to review aim and represented 3 countries (Kenya, Ethiopia, Netherlands), including 1 low-income, 1 lower middle income and 1 high income country.	**Minor concerns:** 3 out of 6 studies contributed with 2 thick and 1 thin data.	**High Confidence**	No or very minor concerns on coherence, minor concerns on relevance (small number of countries), minor concerns on methodological limitations (ethics and reflexivity), and minor concerns on adequacy (3 out of 6 studies contributed with 2 thick and 1 thin data).

Formatted Table

For peer review only

No	Summary of qualitative review findings	Contributing qualitative studies	Methodological limitations assessment	Coherence assessment	Relevance assessment	Adequacy assessment	Overall CERQual assessment	Explanation of overall assessment
	supplements and experienced benefits from it, and a perceived lack of knowledge on how to treat pre-eclampsia which motivated healthcare providers to side towards prevention.				All perspectives came from health providers.			
**8. Structural factors**								
8.1	**High workload, inadequate staffing, stock out, and lack of equipment** In general, healthcare providers felt that their workload increased by including calcium supplements in antenatal care for pregnant women. Healthcare providers reported existing inadequate staffing, yet they needed to provide additional counselling and prescription to women, especially pregnant women with comorbidities.	(Martin 2018, Birhanu 2018)	**No or very minor concerns**	**No or very minor concerns**	**Moderate concerns:** All studies are directly relevant to review aim and represented 2 countries (Kenya, Ethiopia), including 1 low-income and 1 lower middle-income country. All perspectives came from health providers.	**Serious concerns:** 2 out 6 studies contributed to findings (1 thick, 1 thin data).	**Moderate Confidence**	No or very minor concerns on methodological limitations, no or very minor concerns on coherence, minor concerns on relevance (due to all studies coming from low income or lower middle-income countries and small number of countries) and serious concerns on adequacy (2 out 6 studies contributed).

Formatted Table

References

Birhanu Z, Chapleau GM, Ortolano SE, Mamo G, Martin SL, Dickin KL. Ethiopian women's perspectives on antenatal care and iron-folic acid supplementation: Insights for translating global antenatal calcium guidelines into practice. Matern Child Nutr [Internet]. 2018 Feb;14:e12424. Available from: https://onlinelibrary.wiley.com/doi/10.1111/mcn.12424

Kachwaha S, Nguyen PH, Tran LM, Avula R, Young MF, Ghosh S, et al. Specificity Matters: Unpacking Impact Pathways of Individual Interventions within Bundled Packages Helps Interpret the Limited Impacts of a Maternal Nutrition Intervention in India. J Nutr. 2022 Feb;152(2):612–29.

Martin SL, Seim GL, Wawire S, Chapleau GM, Young SL, Dickin KL. Translating formative research findings into a behaviour change strategy to promote antenatal calcium and iron and folic acid supplementation in western Kenya. Matern Child Nutr [Internet]. 2017 Jan 22;13(1):mcn.12233. Available from: https://onlinelibrary.wiley.com/doi/10.1111/mcn.12233 Martin 2017a 38

Martin SL, Omotayo MO, Chapleau GM, Stoltzfus RJ, Birhanu Z, Ortolano SE, et al. Adherence partners are an acceptable behaviour change strategy to support calcium and iron-folic acid supplementation among pregnant women in Ethiopia and Kenya. Matern Child Nutr [Internet]. 2017 Jul;13(3):e12331. Available from: https://onlinelibrary.wiley.com/doi/10.1111/mcn.12331 Martin 2017b 41

Martin SL, Wawire V, Ombunda H, Li T, Sklar K, Tzehaie H, et al. Integrating Calcium Supplementation into Facility-Based Antenatal Care Services in Western Kenya: A Qualitative Process Evaluation to Identify Implementation Barriers and Facilitators. Curr Dev Nutr [Internet]. 2018 Nov 1;2(11). Available from: https://academic.oup.com/cdn/article/doi/10.1093/cdn/nzy068/5078341 39

Omotayo MO, Martin SL, Stoltzfus RJ, Ortolano SE, Mwanga E, Dickin KL. With adaptation, the WHO guidelines on calcium supplementation for prevention of pre-eclampsia are adopted by pregnant women. Matern Child Nutr [Internet]. 2018 Apr;14(2):e12521. Available from: http://doi.wiley.com/10.1111/mcn.12521

Vestering A, Bekker MN, Grobbee DE, van der Graaf R, Franx A, Crombag NMT, et al. Views and preferences of medical professionals and pregnant women about a novel primary prevention intervention for hypertensive disorders of pregnancy: a qualitative study. Reprod Health [Internet]. 2019 May 2;16(1):46. Available from: http://www.ncbi.nlm.nih.gov/pubmed/31046778

For peer review only

Appendix 6. Summary of Quantitative Findings

#	Summary of Quantitative review findings	Contributing quantitative studies	Quality ratings
**Women's factors**			
**1**	**Knowledge and learning**		
1.1	**Women's knowledge about pre-eclampsia or eclampsia**<br>No data supporting this theme from quantitative evidence.	No data supporting this theme from quantitative evidence.	No data supporting this theme from quantitative evidence.
1.2	**Information provision to women**<br>No data supporting this theme from quantitative evidence.	No data supporting this theme from quantitative evidence.	No data supporting this theme from quantitative evidence.
1.3	**Learning about calcium supplementation**<br>Quantitative evidence supported qualitative findings regarding women's learning on calcium supplements. In the context of an intervention implementing training to healthcare providers to reinforce calcium-related messages to women, women reported that they would take calcium in a future pregnancy and that they would recommend calcium to other pregnant women. Women were more likely to consume calcium supplements if they had higher knowledge of calcium benefits (Odds Ratio (OR) 11.7, 95% Confidence Interval (CI): 5.97-22.86) and higher general education (OR 2.59, 95% CI: 2.21-3.05). Higher adherence was also reported in those with higher education (OR 1.45, 95% CI: CI 1.31 to 1.60).(Liu et al., 2019) Higher nutrition knowledge was also associated with taking 6 times more calcium tablets. One paper, evaluating calcium supplement intake before and after nutritional interventions, showed an association between maternal knowledge of nutrition and higher calcium supplement intake (β ~31.9, 95% CI: 20.9, 43.0), however the paper also highlighted there were still large gaps between knowledge and practices, as the intake of calcium supplement tablets during 6 month was low, 82 ±66 out of the recommended 180 tablets.	(Liu et al., 2019; Nguyen et al., 2019, 2017; Thapa et al., 2016)	Four studies. 1 high quality, 1 low quality and 2 moderate quality studies.

For peer review only

**2**	**Believe about the intervention**		
2.1	**Fears about side-effects as barriers to calcium uptake among women**<br>Quantitative evidence supported qualitative findings as few women reported being discouraged to take the supplements by friends (4%) and elder women (3%).	(Martin et al., 2017)	1 Low quality study.
2.2	**Experiences of side effects**<br>Quantitative evidence supported the qualitative findings as some women reported experiencing side effects, usually related to gastrointestinal symptoms. The reported side-effects rates were usually low, 4% of women mentioning nausea or vomiting or constipation in one paper while in another paper 14.9% of women reported that side effects was the reason for missing a dose of IFA or calcium. These could cause supplement discontinuation or erratic supplement intake for one to 10 days after side effects were felt in around 5% of women.	(Baxter et al., 2014; Ghosh-Jerath et al., 2015; Shakya Shrestha et al., n.d.; Thapa et al., 2016)	Four studies. 1 high quality, 2 low quality and 1 very low quality study.
2.3	**Concerns of being stigmatized as HIV patient**<br>No data supporting this theme from quantitative evidence.	No data supporting this theme from quantitative evidence.	No data supporting this theme from quantitative evidence.
2.4	**Positive perceptions about calcium**<br>Quantitative evidence supported the qualitative findings that women's beliefs about the importance of calcium supplements to both the woman's and baby's health and on being able to consume it daily are associated with consuming calcium supplements. Positive beliefs about calcium supplements and self-efficacy were associated with taking calcium supplements (OR 4.6, 95% CI: 2.0 to 10.5) and with taking a higher number of supplements (OR 2.77, 95% CI: 1.68 to 4.57).	(Nguyen et al., 2019)	1 Moderate quality study.
**3**	**Calcium supplement characteristics and regimens**		
3.1	**Varying preferences about characteristics of calcium tablets**<br>Quantitative evidence supported qualitative findings regarding varying preferences calcium supplement's organoleptic properties. One paper which evaluate the impact of a program to implement calcium supplementation showed that most women (77%) reported preferences for conventional tablets that were easier to take and swallow, while the least preferred vehicle was unflavoured powder, as women dislike the taste.(Baxter et al., 2014) Another paper reported that conventional tablets had an acceptable taste (83.9% of women). Chewable tablets	(Baxter et al., 2014; Omotayo et al., 2018b; Thapa et al., 2016)	Three studies. 1 Low quality and 2 high quality studies.

For peer review only - http://bmjopen.bmj.com/site/about/guidelines.xhtml

	were preferred by most women (74%) in another paper. Some characteristics that women considered while taking the supplements include tablet's flavour, chewable or swallow, taken with water or not, smell, and size.		
3.2	**Adherence challenges due to routines**<br>In two quantitative studies, women preferred to take fewer tablets per day. However, in one study, women who were allocated to the study arm that used more frequent doses took more calcium overall.	(Baxter et al., 2014; Omotayo et al., 2018b, 2018a)	Three studies. 1 High quality, 1 moderate and 1 low quality studies.
**4**	**Daily routines and food insecurity**		
4.1	**Adherence challenges due to routines**<br>In quantitative evidence, women described busy work schedules and not being at home as also contributing to forgetting to consume their calcium supplements.	(Baxter et al., 2014; Shakya Shrestha et al., n.d.; Thapa et al., 2016)	Three studies. 1 High quality, 1 low and 1 very low-quality studies.
4.2	**Food insecurity as a barrier to calcium uptake**<br>Quantitative evidence supported qualitative findings that women with food security, high socio-economic status and living in urban areas are more likely to consume calcium supplements as compared to their counterparts. Food security was associated with consumption of 6 more calcium tablets.	(Liu et al., 2019; Nguyen et al., 2017)	Two studies. 1 High quality and 1 low quality studies.
**5**	**Strategies to improve use**		
5.1	**Implementation of reminders to promote adherence**<br>Quantitative evidence supported the qualitative findings that distribution of behaviour change materials to women, such as pill-taking calendars, were associated with increased adherence	(Omotayo et al., 2018b)	1 High quality study.
5.2	**Importance of family support and adherence partner implementation**<br>Quantitative evidence supported the qualitative findings that social support is important in encouraging women to consume calcium supplements. Women considered involving a husband, partner, or family in education sessions or appointing someone as the "adherence partner" to be an acceptable strategy for promoting adherence. Women often chose their husband (52%), older female relative (23%), children (14%), cousins or other relatives as adherence partners (8%), and were satisfied with the reminders and support received from their adherence partner. However, a randomised trial assessing adherence partners in improving calcium supplement intake showed that high social support, instead of	(Martin et al., 2017; Nguyen et al., 2019, 2018, 2017; Omotayo et al., 2018b)	Five studies. 3 high quality studies, 1 moderate and 1 low quality studies.

For peer review only

	adherence partners alone, was associated with higher adherence to calcium supplement (OR: 2.10; 95% CI: 1.32, 3.34). Women with high family support reported higher intake of calcium supplements (OR = 2.1).		
5.3	**Counselling facilitates calcium uptake**<br>Quantitative evidence extended qualitative findings where not only counselling, but also starting antenatal contacts at early gestational age, higher number of antenatal contacts, and receiving free calcium supplements were associated with higher calcium intake by women. One paper reports that women were 59 times more likely to consume calcium supplements if they had received them for free.	(Liu et al., 2019; Nguyen et al., 2019, 2017; Thapa et al., 2016)	Four studies. 2 High quality studies, 1 moderate and 1 low quality studies.
**Health care providers' factors**			
**6**	**Healthcare provider knowledge and training**		
6.1	**Varied knowledge about preeclampsia/eclampsia among providers**<br>No data supporting this theme from quantitative evidence.	No data supporting this theme from quantitative evidence.	No data supporting this theme from quantitative evidence.
6.2	**Inadequate training**<br>No data supporting this theme from quantitative evidence.	No data supporting this theme from quantitative evidence.	No data supporting this theme from quantitative evidence.
**7**	**Beliefs on the intervention**		
7.1	**Perceived overmedicalization when prescribing calcium supplements**<br>No data supporting this theme from quantitative evidence.	No data supporting this theme from quantitative evidence.	No data supporting this theme from quantitative evidence.
7.2	**Beliefs about calcium**<br>No data supporting this theme from quantitative evidence.	No data supporting this theme from quantitative evidence.	No data supporting this theme from quantitative evidence.
**8**	**Structural factors**		
8.1	**High workload, inadequate staffing, stock out, and lack of equipment**<br>Quantitative evidence extended qualitative findings by showing that in the context of a comprehensive integrated program including the implementation of job aids, training, guidelines, monitoring, and feedback session for healthcare providers, could overcome barriers in prescribing women with calcium supplements.	(Omotayo et al., 2018a)	1 Moderate study

For peer review only

References

Baxter, J.-A.B., Roth, D.E., Al Mahmud, A., Ahmed, T., Islam, M., Zlotkin, S.H., 2014. Tablets Are Preferred and More Acceptable Than Powdered Prenatal Calcium Supplements among Pregnant Women in Dhaka, Bangladesh. J. Nutr. 144, 1106–1112. https://doi.org/10.3945/jn.113.188524

Ghosh-Jerath, S., Devasenapathy, N., Singh, A., Shankar, A., Zodpey, S., 2015. Ante natal care (ANC) utilization, dietary practices and nutritional outcomes in pregnant and recently delivered women in urban slums of Delhi, India: an exploratory cross-sectional study. Reprod. Health 12, 20. https://doi.org/10.1186/s12978-015-0008-9

Liu, D., Cheng, Y., Dang, S., Wang, D., Zhao, Y., Li, C., Li, S., Lei, F., Qu, P., Mi, B., Zhang, R., Li, J., Zeng, L., Yan, H., 2019. Maternal adherence to micronutrient supplementation before and during pregnancy in Northwest China: a large-scale population-based cross-sectional survey. BMJ Open 9, e028843. https://doi.org/10.1136/bmjopen-2018-028843

Martin, S.L., Omotayo, M.O., Pelto, G.H., Chapleau, G.M., Stoltzfus, R.J., Dickin, K.L., 2017. Adherence-specific social support enhances adherence to calcium supplementation regimens among pregnant women. J. Nutr. 147. https://doi.org/10.3945/jn.116.242503

Nguyen, P.H., Frongillo, E.A., Sanghvi, T., Wable, G., Mahmud, Z., Tran, L.M., Aktar, B., Afsana, K., Alayon, S., Ruel, M.T., Menon, P., 2018. Engagement of Husbands in a Maternal Nutrition Program Substantially Contributed to Greater Intake of Micronutrient Supplements and Dietary Diversity during Pregnancy: Results of a Cluster-Randomized Program Evaluation in Bangladesh. J. Nutr. 148, 1352–1363. https://doi.org/10.1093/jn/nxy090

Nguyen, P.H., Kachwaha, S., Avula, R., Young, M., Tran, L.M., Ghosh, S., Agrawal, R., Escobar-Alegria, J., Patil, S., Menon, P., 2019. Maternal nutrition practices in Uttar Pradesh, India: Role of key influential demand and supply factors. Matern. Child Nutr. 15. https://doi.org/10.1111/mcn.12839

Nguyen, P.H., Sanghvi, T., Kim, S.S., Tran, L.M., Afsana, K., Mahmud, Z., Aktar, B., Menon, P., 2017. Factors influencing maternal nutrition practices in a large scale maternal, newborn and child health program in Bangladesh. PLoS One 12, e0179873. https://doi.org/10.1371/journal.pone.0179873

Omotayo, M.O., Dickin, K.L., Pelletier, D.L., Martin, S.L., Kung'u, J.K., Stoltzfus, R.J., 2018a. Feasibility of integrating calcium and iron-folate supplementation to prevent preeclampsia and anemia in pregnancy in primary healthcare facilities in Kenya. Matern. Child Nutr. 14, e12437. https://doi.org/10.1111/mcn.12437

Omotayo, M.O., Martin, S.L., Stoltzfus, R.J., Ortolano, S.E., Mwanga, E., Dickin, K.L., 2018b. With adaptation, the WHO guidelines on calcium supplementation for prevention of pre-eclampsia are adopted by pregnant women. Matern. Child Nutr. 14, e12521. https://doi.org/10.1111/mcn.12521

Shakya Shrestha, S., Adhikari, R., Tamrakar, S., Shrestha, R., Shrestha, A., n.d. Adherence to Iron, Folic Acid and Calcium Supplement and Factors Affecting it among the Antenatal Care Attending Women in a Tertiary Care Hospital: A Cross Sectional Study. Kathmandu Univ. Med. J. (KUMJ) 18, 186–192.

Thapa, K., Sanghvi, H., Rawlins, B., Karki, Y.B., Regmi, K., Aryal, S., Aryal, Y., Murakami, P., Bhattarai, J., Suhowatsky, S., K., T., H., S., B., R., Y.B., K., K., R., S., A., Y., A., P., M., J., B., S., S., 2016. Coverage, compliance, acceptability and feasibility of a program to prevent pre-eclampsia and eclampsia through calcium supplementation for pregnant women: An operations research study in one district of Nepal. BMC Pregnancy Childbirth. https://doi.org/10.1186/s12884-016-1033-6

For peer review only - http://bmjopen.bmj.com/site/about/guidelines.xhtml

Appendix 7. COM-B Mapping Table

Behavior aimed: calcium supplementation use by women and health providers						
No	COM-B Domain	TDF Domain*	List of factors affecting calcium use	Stakeholders affected (women, health providers, partner)	Evidence Source (Quantitative/Qualitative)	Barriers or facilitators to calcium use
**Women's factors**						
1	Capability	Know	Limited knowledge about preeclampsia/eclampsia	Women	Qualitative	
2	Capability	Know	Counselling on information about preeclampsia and calcium from their providers independent to woman risk	Women	Qualitative	
3	Capability	Know	High knowledge of calcium supplementation	Women	Quantitative	
4	Capability	Know	High education of women	Women	Quantitative	
5	Capability	Know	Symptoms were believed to be linked to evil attacks or nutritional deficiencies	Women	Qualitative	
6	Capability	Know	Inadequate information on preeclampsia/eclampsia and calcium from providers	Women	Qualitative	
7	Capability	Know	Receive adequate information about condition and calcium from their providers	Women	Qualitative	
8	Motivation	Bel Cons	Preeclampsia/eclampsia was not considered as a serious problem by women	Women	Qualitative	
9	Motivation	Bel Cons	Experiences and fears of side effects	Women	Qualitative and quantitative	
10	Motivation	Bel Cons	Assurance of calcium safety	Women	Qualitative	
11	Motivation	Bel Cons	Safety of calcium was not assured	Women	Qualitative	
12	Motivation	Bel Cons	Experienced and expected benefits from taking calcium and folic acid	Women	Qualitative and quantitative	
13	Motivation	Bel Cap	Belief that having essential information would help them making informed decisions	Women	Qualitative	
14	Motivation	Bel Cap	Belief that providers is a reliable source of information	Women	Qualitative	
15	Motivation	Bel Cap	Belief of being able to consume calcium daily	Women	Quantitative	
16	Motivation	Em	Valued information, education and communication (IEC) materials	Women	Qualitative	
17	Motivation	Em	Positive perceptions on calcium tablet characteristics (e.g. size, taste, smell)	Women	Qualitative and quantitative	
18	Motivation	Em	Women felt that 3-4 pills per day at multiple times were too many	Women	Quantitative and qualitative	
19	Motivation	Em	Inconvenience in taking pill daily	Women	Quantitative and qualitative	
20	Motivation	Em	Belief that one combined pill per day could ease burden	Women	Qualitative	
21	Motivation	Em	Feeling confident taking calcium after receiving adequate information from provider	Women	Quantitative	
22	Opportunity	Soc	Discouragement by family, neighbours and community in taking calcium	Women	Qualitative and quantitative	

For peer review only

23	Opportunity	Soc	Stigmatisation of having supplements and posters with HIV	Women	Qualitative
24	Opportunity	Soc	Family support in consuming calcium	Women	Qualitative and quantitative
25	Opportunity	Soc	Positive belief and experiences about 'adherence partners'	Women	Qualitative and quantitative
26	Opportunity	Ev	Conflicting daily routine with taking calcium	Women	Qualitative and quantitative
27	Opportunity	Ev	Food insecurity	Women	Qualitative and quantitative
28	Opportunity	Ev	Early initiation and frequent antenatal visits	Women	Quantitative
29	Opportunity	Ev	Receiving free calcium supplements	Women	Quantitative
30	Opportunity	Ev	Reminder tools distribution, such as home based posters and pill-taking calendars	Women	Qualitative and Quantitative
31	Opportunity	Ev	Universal free calcium distribution through antenatal care	Women	Quantitative
**Providers factors**					
1	Capability	Know	Varied knowledge on pre-eclampsia/eclampsia	Providers	Qualitative
3	Capability	Skills	Incontinuity and non-in-depth training about pre-eclampsia/eclampsia	Providers	Qualitative
2	Capability	Skills	Felt inadequately trained	Providers	Qualitative
4	Capability	Skills	Continuous training to manage the condition and address resistance from community	Providers	Qualitative
5	Motivation	Bel Cons	Positive belief about calcium supplementation benefits	Providers	Qualitative
6	Motivation	Bel Cons	Belief that preeclampsia/eclampsia was not a priority health concern	Providers	Qualitative
7	Motivation	Bel Cons	Perceived over-medicalization to prescribe calcium to low risk women	Women and Providers	Qualitative
8	Motivation	Bel Cons	Fears in generating anxiety on low-risk women	Providers	Qualitative
9	Motivation	Bel Cons	Belief that women should get a chance to receive information regardless risk	Providers	Qualitative
10	Opportunity	Ev	Providers felt that providing calcium would increase workload	Providers	Qualitative
11	Opportunity	Ev	Inadequate number of staff providing care	Providers	Qualitative
12	Opportunity	Ev	Comprehensive integrated program (job aids, training, guidelines, regular supplies)	Providers	Quantitative

Barriers	
Facilitators	

* Know: Knowledge, Phys: Physical skills, Mem: Memory, attention and decision processes, Beh Reg: Behavioural regulation, Em: Emotion, Id: Social/professional role and identity, Bel Cons: Belief about consequences, Bel Cap: Belief about capabilities, Int: Intentions, Opt: Optimisms, Ev: Environmental context and resources, Soc: Social influences

For peer review only

BMJ Open

Factors affecting the implementation of calcium supplementation strategies during pregnancy to prevent pre eclampsia: a mixed-methods systematic review

Journal:	*BMJ Open*
Manuscript ID	bmjopen-2022-070677.R3
Article Type:	Original research
Date Submitted by the Author:	29-Nov-2023
Complete List of Authors:	Cormick, G; Instituto de Efectividad Clinica y Sanitaria, Centro de Investigaciones en Epidemiología y Salud Pública (Consejo Nacional de Investigaciones Científicas y Técnicas- CONICET); Universidad Nacional de La Matanza, Departamento de Salud<br>Moraa, Helen; University of Nairobi<br>Zahroh, Rana; University of Melbourne<br>Allotey, John; Univ Birmingham<br>Rocha, Thaís; University of Birmingham<br>Pena-Rosas, Juan-Pablo; Organisation mondiale de la Sante, Nutrition for Health and Development<br>Qureshi , Zahida ; University of Nairobi, Obstetrics and Gynaecology<br>Hofmeyr, G Justus ; University of the Witwatersrand Faculty of Health Sciences, Effective Care Research Unit<br>Mistry, Hema; University of Birmingham<br>Smits, Luc; Maastricht University<br>Vogel, Joshua; Burnet Institute; World Health Organization, Reproductive Health and Research<br>Palacios , Alfredo ; Institute for Clinical Effectiveness and Health Policy<br>Gwako, George N. ; University of Nairobi, Obstetrics and Gynaecology ;<br>Abalos, E; Univ Western Australia<br>Larbi, Koiwah Koi; ACTION ON PREECLAMPSIA<br>Carroli, Guillermo; CREP<br>Riley, Richard; University of Birmingham<br>Snell, Kym IE; Keele University<br>Thorson, Anna; Human Reproduction Program/World Health Organization (Geneva)<br>Young, Taryn; Stellenbosch University, Centre for Evidence-based Health Care, Division Epidemiology and Biostatistics, Dept. of Global Health<br>Betran, Ana Pilar; World Hlth Org, Reproductive Health and Research<br>Thangaratinam, Shakila; Institute of Metabolism and Systems Research University of Birmingham<br>Bohren, Meghan ; University of Melbourne School of Population and Global Health, Centre for Health Equity
**Primary Subject Heading**:	Obstetrics and gynaecology
Secondary Subject Heading:	Qualitative research, Public health, Health policy, Global health
Keywords:	Hypertension < CARDIOLOGY, OBSTETRICS, PUBLIC HEALTH

SCHOLARONE™
Manuscripts

I, the Submitting Author has the right to grant and does grant on behalf of all authors of the Work (as defined in the below author licence), an exclusive licence and/or a non-exclusive licence for contributions from authors who are: i) UK Crown employees; ii) where BMJ has agreed a CC-BY licence shall apply, and/or iii) in accordance with the terms applicable for US Federal Government officers or employees acting as part of their official duties; on a worldwide, perpetual, irrevocable, royalty-free basis to BMJ Publishing Group Ltd ("BMJ") its licensees and where the relevant Journal is co-owned by BMJ to the co-owners of the Journal, to publish the Work in this journal and any other BMJ products and to exploit all rights, as set out in our licence.

The Submitting Author accepts and understands that any supply made under these terms is made by BMJ to the Submitting Author unless you are acting as an employee on behalf of your employer or a postgraduate student of an affiliated institution which is paying any applicable article publishing charge ("APC") for Open Access articles. Where the Submitting Author wishes to make the Work available on an Open Access basis (and intends to pay the relevant APC), the terms of reuse of such Open Access shall be governed by a Creative Commons licence – details of these licences and which Creative Commons licence will apply to this Work are set out in our licence referred to above.

Other than as permitted in any relevant BMJ Author's Self Archiving Policies, I confirm this Work has not been accepted for publication elsewhere, is not being considered for publication elsewhere and does not duplicate material already published. I confirm all authors consent to publication of this Work and authorise the granting of this licence.

For peer review only

Factors affecting the implementation of calcium supplementation strategies during pregnancy to prevent pre-eclampsia: a mixed-methods systematic review

Gabriela Cormick [1,2,3], Helen Moraa [4], Rana Islamiah Zahroh [5], John Allotey [6], Thais Rocha [6], Juan Pablo Peña-Rosas [7], Zahida Qureshi [8], G Justus Hofmeyr [9], Hema Mistry [10], Luc JM Smits [11], Joshua P. Vogel [12], Alfredo Palacios [13], Gwako George Nyakundi[4], Edgardo Abalos [14], Koiwah Koi-Larbi [15], Guillermo Carroli [16], Richard Riley [17], Kym Snell[17], Anna Thorson [18], Taryn Young [19], Ana Pilar Betran[18], Shakila Thangaratinam [6,17], Meghan A. Bohren[5], for I-CIP Collaborative Network

1. Centro de Investigaciones en Epidemiología y Salud Pública (CIESP-IECS), CONICET, Buenos Aires, Argentina
2. Instituto de Efectividad Clinica y Sanitaria (IECS-CONICET), Buenos Aires, Argentina
3. Departamento de Salud, Universidad Nacional de La Matanza (UNLAM), San Justo 1903, Argentina.
4. University of Nairobi
5. Gender and Women's Health Unit, Nossal Institute for Global Health, School of Population and Global Health, University of Melbourne, Carlton, Victoria, Australia
6. WHO Collaborating Centre for Global Women's Health, Institute of Metabolism and Systems Research, University of Birmingham, Birmingham, UK
7. Department of Nutrition and Food Safety, World Health Organization, Geneva, Switzerland
8. Department of Obstetrics and Gynaecology, University of Nairobi, Nairobi Kenya.
9. Department of Obstetrics and Gynaecology, University of Botswana, Gaborone, Botswana; Effective Care Research Unit, University of the Witwatersrand and Walter Sisulu University, East London, South Africa.
10. Clinical Trials Unit, Warwick Medical School, University of Warwick, Coventry, CV4 7AL, United Kingdom; University Hospitals Coventry and Warwickshire NHS Trust, Coventry, CV2 2DX, United Kingdom
11. Department of Epidemiology, CAPHRI Care and Public Health Research Institute, Maastricht University, Maastricht, The Netherlands
12. Maternal, Child and Adolescent Health Program, Burnet Institute, 85 Commercial Road, Melbourne, 3000, Australia

For peer review only - http://bmjopen.bmj.com/site/about/guidelines.xhtml

13. Department of Health Technology Assessment and Health Economics, Institute for Clinical Effectiveness and Health Policy (IECS), Buenos Aires, Argentina; Centre for Health Economics (CHE), University of York, York, United Kingdom
14. Centro de estudios de Estado y Sociedad, Buenos Aires, Argentina
15. Action on preeclampsia Charity (APEC), Ghana
16. Centro Rosarino de Estudios Perinatales (CREP), Rosario, Argentina
17. Institute of Applied Health Research, University of Birmingham, Birmingham, UK
18. UNDP-UNFPA-UNICEF-WHO-World Bank Special Programme of Research Development and Research Training in Human Reproduction (HRP), Department of Sexual and Reproductive Health and Research, World Health Organization, Geneva, Switzerland.
19. Centre for Evidence-based Health Care, Division of Epidemiology and Biostatistics, Department of Global Health, Faculty of Medicine and Health Sciences, Stellenbosch University, Stellenbosch, South Africa
20. Birmingham Women's and Children's NHS Foundation Trust, Birmingham, UK

Correspondence to:

Gabriela Cormick

gabmick@yahoo.co.uk

For peer review only

Abstract

Objectives Daily calcium supplements are recommended for pregnant women from 20 weeks' gestation to prevent pre-eclampsia in populations with low dietary calcium intake. We aimed to improve understanding of barriers and facilitators for calcium supplement intake during pregnancy to prevent pre-eclampsia.

Design Mixed-method systematic review, with confidence assessed using the GRADE-CERQual approach.

Data sources MEDLINE and EMBASE (via Ovid), CINAHL and Global Health (via EBSCO) and grey literature databases were searched up to Sept 17, 2022.

Eligibility criteria We included primary qualitative, quantitative, and mixed-methods studies reporting implementation or use of calcium supplements during pregnancy, excluding calcium fortification and non-primary studies. No restrictions were imposed on settings, language, or publication date.

Data extraction and synthesis Two independent reviewers extracted data and assessed risk of bias. We analysed the qualitative data using thematic synthesis, and quantitative findings were thematically mapped to qualitative findings. We then mapped the results to behavioural change frameworks to identify barriers and facilitators.

Results Eighteen reports from nine studies were included in this review. Women reported barriers to consuming calcium supplements included limited knowledge about calcium supplements and pre-eclampsia, fears and experiences of side effects, varying preferences for tablets, dosing, working schedules, being away from home and taking other supplements. Receiving information regarding pre-eclampsia and safety of calcium supplement use from reliable sources, alternative dosing options, supplement reminders, early antenatal care, free supplements and support from families and communities were reported as facilitators. Healthcare providers felt that consistent messaging about benefits and risks of calcium, training, and ensuring adequate staffing and calcium supply is available would be able to help them in promoting calcium.

Conclusion Relevant stakeholders should consider the identified barriers and facilitators when formulating interventions and policies on calcium supplement use. These review findings can inform implementation to ensure effective and equitable provision and scale-up of calcium interventions.

Study registration: PROSPERO, CRD42021239143..

For peer review only

For peer review only - http://bmjopen.bmj.com/site/about/guidelines.xhtml

Strengths and limitations of this study

- We adopted a mixed-methods approach, which allowed for inclusion and consolidation of studies with a range of designs.
- The strength of our review lies on behaviour change frameworks mapping, which improved our understanding of how barriers and facilitators influence calcium uptake and potential strategies to address them.
- The transferability of our results may be limited, as studies were mostly from low- and middle-income countries and almost half came from the same project in Kenya and Ethiopia.
- Most included studies engaged women who attended antenatal care, therefore, might not be representative of those that do not reach the health system during pregnancy.

For peer review only

INTRODUCTION

Hypertensive disorders of pregnancy are among the leading causes of maternal and perinatal morbidity and mortality globally.(1) Pre-eclampsia is a hypertensive disorder of pregnancy characterised by hypertension developing after 20 weeks' gestation, combined with proteinuria or other new onset of maternal organ dysfunction, while eclampsia is a severe form of pre-eclampsia characterised by seizures.(2,3) Pre-eclampsia contribute to approximately 14% of the 300,000 maternal deaths worldwide annually.(4) Management of pre-eclampsia requires regular monitoring and evaluation of the woman and her baby to achieve an optimal timing of birth and prevent severe complications.(5) Preventive strategies are essential to reduce the burden of morbidity and mortality, especially in low- and middle-income countries (LMICs) where most complications occur.

The World Health Organization (WHO) recommends 1.5 to 2 grams a day from 20 weeks' gestation for women who are living in populations with low dietary calcium intake, especially those at high risk of developing pre-eclampsia. (6) This is aligned with findings from a systematic review that reported calcium supplements during pregnancy compared to placebo may reduce the risk of pre-eclampsia by 55% (13 trials, 15,730 participants; Relative Risk (RR) 0.45, 95% CI: 0.31 to 0. 65).(7) Moreover, maternal death or severe morbidity was reduced by 20% with calcium supplements (four trials, 9,732 participants; RR 0.80, 95% CI 0.66 to 0.98). (7) The evidence base has since been updated multiple times, with WHO recommendation consistently updated up to 2018.(8–10)

Despite the WHO recommendation, calcium supplement during pregnancy remains low in LMICs and rates of pre-eclampsia are not falling in regions where calcium supplementation is recommended.(10) Practical challenges to implementing WHO recommendations have been documented. For example, women need to take three, spaced tablets to achieve the requisite daily dose, and this needs be separated from timing of intake of other supplements (such as iron) to optimise calcium absorption.(11) In addition, antenatal care services need consistent supplies of calcium tablets, which can be hindered by logistical issues in supplement distribution and storage.(7) We conducted a mixed-methods systematic review aiming to improve understanding of the barriers and facilitators of calcium supplement intake during pregnancy to prevent pre-eclampsia, from the perspectives of women, families, community members, healthcare providers, and policymakers.

For peer review only

METHODS

This mixed-methods review is reported according to Preferred Reporting Items for Systematic Reviews and Meta-Analyses (PRISMA) (Appendix 1) (12), Enhancing Transparency in Reporting the Synthesis of Qualitative Research (ENTREQ) statement (Appendix 2) (13), and based on guidance from Cochrane Effective Practice and Organisation of Care.(14) The protocol was registered on PROSPERO (CRD42021239143).

Topic of interest and types of studies

We included studies that documented perspectives, perceptions and experiences of women who experienced or were at risk of pre-eclampsia and/or received calcium-containing supplements. We also included studies on the views of their partners or families, as well as studies on maternity healthcare providers (e.g., midwives, nurses, doctors) and other relevant stakeholders (e.g., facility managers, policymakers) involved in decisions on calcium supplements in pregnancy. There were no limitations imposed on geographical location or type of health facility. The timeframe for using calcium supplement is during pregnancy, independent of the gestational age.

We included primary qualitative, quantitative, and mixed-methods studies reporting implementation or use of calcium supplements in any presentations including powder, granule, chewable tablet, capsule, liquid filled capsule, tablet, suspension, or powder for suspension. We did not include studies assessing the effects of calcium fortified foods or beverages. We excluded case reports or case series, letters, editorials, commentaries, reviews, study protocols, posters, and conference abstracts or other study sources that did not provide primary data.

Search strategy

We searched MEDLINE and EMBASE via Ovid, CINAHL and Global Health via EBSCO to identify eligible studies from inception to Sept 17, 2022. A search strategy was developed and adapted for each database (Appendix 3), using different terms for calcium and pregnancy. No limitations on publication date or language were imposed. Grey literature searches were also conducted using OpenGrey and Google. We checked reference lists of included studies to identify any relevant record not retrieved in the database search.

Study selection

After removing duplicates in EndNote, records were imported to Covidence for screening.(15) Two of the following authors (GC, GG, APB, RIZ, HM) independently assessed eligibility of each record by comparing titles and abstracts against the eligibility criteria. Full texts of potentially eligible papers were retrieved and assessed, disagreements were resolved through discussion or consulting a third author. Papers emerging from the same study were collated and treated as one data source. Titles and abstracts of papers published in languages other than English, French, and Spanish were translated through open-source software (Google Translate) to assess their eligibility. Had we identified any relevant titles or abstracts

For peer review only - http://bmjopen.bmj.com/site/about/guidelines.xhtml

in languages other than English, French and Spanish, we would have sought formal translation of the full texts from a native speaker.

Data extraction and assessment of methodological limitations

Using a pre-designed form, two reviewers (GC, RIZ) independently extracted data from included studies on study characteristics (setting, sample size, characteristics of participants, objectives), design (data collection and analysis methods), qualitative data (themes, findings, and quotations) and quantitative data (data source, outcome measures, results, measures of compliance or uptake).

All included studies underwent quality appraisal by two authors (GC, HM, RIZ). As the review included quantitative, qualitative and mixed-methods studies, we used the Mixed Methods Appraisal Tool (MMAT), which produces a single quality rating on the basis of: aims, methodology, design, recruitment, data collection, blinding, data analysis, selective reporting, reflexivity, ethical considerations, results, research contribution, and other sources of bias.(16) Appraisal of study quality was used to inform data analysis, and not to exclude studies.

Quality appraisal, analysis and assessing confidence

We conducted a preliminary qualitative synthesis using a thematic analysis approach.(17) Thematic analysis is a valuable method in analysing qualitative data to examine perspectives, preferences, experiences, acceptability, feasibility, and other factors that can influence implementation.(17) The analysis begins with initial readings to build our familiarity with the data. Two reviewers (GC, HM) independently conducted line-by-line coding of findings of two qualitative studies.(18,19) From this we developed the qualitative codebook, which was then used to code all other included studies. Next, we generated analytical themes and interpretations to explore relationships within and across studies. This was achieved by organising codes into a hierarchy and identifying barriers and facilitators between study characteristics and findings or exploring different findings across studies. Once qualitative themes were generated, a summary of qualitative findings was developed. Quantitative findings were then narratively mapped to qualitative themes to explore areas of convergence or divergence. ATLAS.ti was used to manage data analysis.

After the thematic analysis, we mapped the qualitative themes to the Theoretical Domains Framework (TDF) and Capability, Opportunity, and Motivation of Behaviour (COM-B) models.(20,21) TDF and COM-B are interrelated behaviour change models which can guide implementation research and intervention design in understanding barriers and facilitators of intended behaviours. We used TDF and COM-B to explore barriers and facilitators of healthcare providers and women to use calcium supplements during pregnancy using evidence-based behaviours to identify potential behaviour change intervention strategies.

We used the GRADE-CERQual (Grading of Recommendations, Assessment, Development, and Evaluations - Confidence in the Evidence from Reviews of Qualitative research) approach to assess confidence in each qualitative finding. (22) GRADE-CERQual assessed confidence based on four key components: methodological limitations (23), coherence (24), adequacy (25), and

For peer review only - http://bmjopen.bmj.com/site/about/guidelines.xhtml

relevance. (26) After assessing each of the four components, we assessed the overall confidence (22) as high, moderate, low, or very low.

Patient and public involvement

None.

RESULTS

We included 18 papers from 16 studies (Figure 1). Included papers were published in English between 2014 and 2022. Out of 16 studies, 10 were quantitative and came from 11 papers (27–37), four were qualitative (18,19,38,39) and two were mixed-methods and came from three papers (40–42) Detailed study characteristics can be found in Table 1.

Table 1 Characteristics of included studies

Study	Country	Population	Number of participants	Methods	Ref
Qualitative					
Birhanu 2018	Ethiopia	Pregnant women and healthcare providers	20 women, 22 healthcare providers	Phenomology	19
Martin 2017a	Kenya	Pregnant women and healthcare providers	22 women, 20 healthcare providers	Phenomology	38
Martin 2018	Kenya	Healthcare providers, pregnant women, adherence partners	7 healthcare providers, 32 pregnant women, 20 adherence partners	Phenomology	39
Vestering 2019	Netherlands	Healthcare providers and pregnant women	8 healthcare providers, 25 women	Phenomology	18
Mixed methods					
Kachwaha 2022	India	Healthcare providers, supervisors, and facility staffs	~500 healthcare providers and supervisors, and 20 block level staffs	Mixed methods	42
Martin 2017b & Omatoyo 2018a	Kenya and Ethiopia	Pregnant women	85 pregnant women, 50 in Ethiopia, 38 in Kenya	Trials of improved practices	41, 40
Quantitative					
Baxter 2014	Bangladesh	Pregnant women	132	Modified discrete-choice trial	30
Bora 2022	India	Pregnant women	3,097,274	Cross-sectional study	27
Ghosh-Jareth 2015	India	Pregnant women and recently delivered women	184 pregnant women and 160 recently delivered women	Cross-sectional study	29
Liu 2019	China	Women aged from 16 to 49 years who were pregnant between 2010 and 2013 and had specific pregnancy outcomes before the survey	30,027	Cross-sectional study	36
Martin 2017c & Omotayo 2018b	Kenya	Pregnant women	990 pregnant women and 16 facilities	Process evaluation study adopting program impact pathway	33, 28
Nguyen 2017	Bangladesh	Pregnant women and recently delivered women	600 pregnant women and 2000 recently delivered women	Cross-sectional household surveys	34
Nguyen 2018	Bangladesh	Women and husbands	1000 women and 70% of their husbands	Cluster randomised control trial with cross-sectional household surveys	35
Nguyen 2019	India	Pregnant women and recently delivered women	667 pregnant women and 1835 recently delivered women	Cross-sectional household surveys	32
Shakya Shrestha 2020	Nepal	Pregnant women	191	Cross-sectional study	37

For peer review only - http://bmjopen.bmj.com/site/about/guidelines.xhtml

Thapa 2016	Nepal	Pregnant and postpartum women, health facilities, female community health volunteers	1240	Prospective collection and secondary analysis of monitoring data captured by the MOHP	31

Five studies aimed to evaluate the implementation of calcium supplements in pregnancy. (27,30,31,39,40) Seven studies evaluated the incorporation of calcium supplement recommendation to other recommended supplements taken during pregnancy, including aspirin (18), and iron and folic acid supplements. (19,28,33,37,38,41,42) Four studies focused on general nutritional practices during pregnancy (29,32,34,35) and one study on all types of micronutrient supplements used before and during pregnancy. (36) Five studies came from one project, Micronutrient Initiative-Cornell University Calcium (MICa) projects (19,28,33,38–41), and four studies came from Alive & Thrive (A&T) project (32, 34, 35, 42).

The studies were conducted in seven different countries across three regions. In Sub-Saharan Africa, three studies were conducted in Kenya (28, 33, 38,39), one study in Ethiopia (19), and one study in Kenya in Ethiopia (40,41). In Asia, four studies were conducted in India (27, 29, 32, 42), three studies in Bangladesh (30, 34, 35), two studies in Nepal (31,37), and one study in China (36). There is one study conducted in Europe, specifically the Netherlands. (18)

All four qualitative studies involved pregnant women and healthcare providers (18,19,38), while one study also included adherence partners to support and remind women to take medication. (39) One mixed-method study included pregnant women (40,41) and one included healthcare providers and facility staff. (42) Among the ten quantitative studies, eight included pregnant women or women who had recently given birth (27–30,32,33, 34,36,37), one included healthcare providers (31) and one included both women and their husbands. (35)

Results of the critical appraisal of the included studies are available in Appendix 4. The main areas of concern for qualitative studies were an unclear or partial description of reflexivity and limited information regarding ethical considerations. For the quantitative studies, main concerns were regarding the appropriateness of measurement tools, lack of detail regarding nonresponse bias, and insufficient information regarding statistical analysis.

Qualitative and quantitative synthesis

We identified five themes related to factors affecting calcium supplement intake during pregnancy: 1) women's existing knowledge and learning; 2) women's beliefs about calcium supplements; 3) calcium supplement characteristics and dose regimens; 4) challenges due to daily routines and food insecurity; and 5) strategies to improve the use of calcium. We also identified three themes related to factors affecting healthcare providers' prescription of calcium supplements: 1) health provider knowledge and training; 2) their beliefs about calcium supplements; and 3) structural factors on site. Across all themes, there were 19 qualitative findings (Table 2): 11 findings were high confidence, six were moderate confidence

For peer review only

and two were low confidence (Appendix 5: Evidence profile). The quantitative findings which were mapped to qualitative themes can be found in Appendix 6.

Table 2 Summary of qualitative findings

No	Summary of qualitative review findings	Contributing qualitative studies	Overall CERQual assessment	Explanation of overall assessment
**1. Knowledge and learning**				
1.1	**Women's knowledge about pre-eclampsia/eclampsia** Most women had limited knowledge about preeclampsia/eclampsia, and preeclampsia/eclampsia was not typically considered as a serious problem by women. General symptoms of preeclampsia/eclampsia such as swollen feet, severe headache, blurred vision and vomiting were considered normal in pregnancy, while seizures were linked to evil attacks or nutritional deficiencies	(26,27,34)	**High confidence**	Due to no or very minor concerns on coherence, minor concerns on methodological limitations (reflexivity and ethics), moderate concerns on relevance (1 out of 3 studies are indirectly relevant to our review aim and small number of countries), and minor concerns of adequacy (3 out of 6 contributed with 2 thick and 1 thin data)
1.2	**Information provision to women** Women felt they did not receive adequate information during pregnancy from health workers about preeclampsia/eclampsia and calcium supplementation and would like to be given more information regardless of their risk status. Women believed that having this essential information could help them to make informed choices and to actively participate in their care. There were, however, mixed opinions from health workers, where some feared that more information could generate anxiety for women, while others were supportive of providing information to women	(13,34)	**Low confidence**	Minor concerns on methodological limitations (ethics and reflexivity), moderate concerns on coherence (no clear understanding on why some health workers worry in generating anxiety to women while the others are not), moderate concerns on relevance (small number of countries), and serious concerns on adequacy (2 out of 6 contributed with 1 thick and 1 thin data)
1.3	**Learning about calcium supplementation** Women typically learned about calcium, including pre-eclampsia and eclampsia symptoms, from health workers, and considered health workers as the most trusted and reliable source of information. They reported feeling confident about taking calcium after receiving adequate information from their health workers. Women also appreciated receiving information on calcium supplementation and preeclampsia/eclampsia from information, education, and communication (IEC) materials like videos, media and trusted websites	(13,27,34)	**High confidence**	Due to no or very minor concerns on coherence, minor concerns on relevance (small number of countries), minor concerns on methodological limitations (reflexivity and ethics), and minor concerns on adequacy (3 out of 6 contributed, 1 moderate thick and 2 thin data)
**2. Believe about the intervention**				
2.1	**Fears about side effects as barriers to calcium uptake among women** Fears about side effects impact adherence to calcium consumption by women. Women highlighted that assurance of safe use of calcium is a key facilitator to consistent use. However, some women felt safety was not assured by health workers and were told by their families or communities that any pills consumed during pregnancy could be harmful, especially when the intervention was perceived as "experimental"	(11,13,27,34)	**High Confidence**	Due to no or very minor concerns on coherence, minor concerns on relevance (small number of countries), minor concerns on adequacy (small number of studies contributing to the qualitative evidence synthesis), and minor concerns on methodological limitations (reflexivity and ethics)
2.2	**Experiences of side effects** Some women reported experiencing side effects after taking calcium and iron folic acid, which include dizziness, vomiting, nausea, stomach-ache, loss appetite, tiredness, diarrhea, bloating, and burping, yet noted that the side effects subsided with time. Women also reported to keep consuming the medication despite experiencing the side effects	(11,13,27)	**High Confidence**	Due to no or very minor concerns on coherence, minor concerns on methodological limitations (ethics and reflexivity), moderate concerns on relevance (only low and lower middle-income country and small number of countries) and minor concerns on adequacy (3 out of 6 studies contributed with 2 thick and 1 with thin data)
2.3	**Concerns of being stigmatized as HIV patient** Women expressed concerns about being stigmatized as HIV patients if they ingested calcium, which was a reported barrier to use. Some women were afraid of being stigmatized as their community often associated supplement consumption and accompanying reminder posters with HIV	(13,34)	**Moderate Confidence**	Due to no or very minor concerns on methodological limitations, no or very minor concerns on coherence, minor concerns on relevance (small number of countries), and serious concerns on adequacy (2 out of 6 study contributed)
2.4	**Positive perceptions about calcium** Women reported that both their perceptions about expected benefits and previous experiences of taking calcium and iron-folic acid were facilitators of use. Women believed that consuming pills could compensate for sub-optimal nutrition during pregnancy and that consuming nutrient supplementation during pregnancy would help keep the baby safe	(11,13,27,28, 34)	**High Confidence**	Due to no or very minor concerns on coherence, minor concerns on methodological limitations (ethics and reflexivity), minor concerns on relevance (1 study indirectly relevant to review aim and small number of countries) and adequacy (small number of studies contributing to the qualitative evidence synthesis)
**3. Medication characteristics and doses**				
3.1	**Varying preferences about characteristics of calcium tablets** Positive perceptions about the characteristics of the calcium tablet played a role in motivating women to take it. Some women preferred the chewable, sweet-tasting tablets that could be swallowed without water, while others preferred the hard tablets which were smaller in size, had no smell, and	(11,27)	**Moderate confidence**	Due to no or very minor concerns on coherence, minor concerns on methodological limitations (reflexivity), moderate concerns on relevance (all low or lower middle-income countries and small number of countries) and moderate concerns on adequacy (2 out of 6 studies contributed with moderate to thick data)

For peer review only

	needed to be taken with water. Based on individual preference, the taste, smell, size, and convenience were instrumental in uptake of the calcium supplements			
**3.2**	**Medication dosing as a barrier of use**<br>Women described that they could feel overwhelmed with the number of calcium pills they had to take per day, this includes women with comorbidities who need to take additional medications to manage their health condition. Women felt that 3-4 pills per day at multiple times was overly onerous and recommended combining them into one pill could ease the burden	(11,13,27)	**High Confidence**	No or very minor concerns on coherence, minor concerns on methodological limitations (reflexivity), minor concerns on adequacy (3 out of 6 studies contributed with moderate to thick data), and moderate concerns on relevance (small number of countries)
**4**	**Routine and food insecurity**			
**4.1**	**Adherence challenges due to routines**<br>Adherence to calcium consumption was challenging for some women because of conflicting activities in women's daily routine such as consuming other medications, travelling, being away from home and household chores, which make women forgetting to take their medication	(11,13,27)	**High Confidence**	Due to no or very minor concerns with coherence, minor concerns on methodological limitations (reflexivity), moderate concerns on relevance (small number of countries) and moderate concerns on adequacy (small number of studies contributing to qualitative evidence synthesis)
**4.2**	**Food insecurity as a barrier to calcium uptake**<br>Women believed that adequate foo d was necessary to be able to take the supplements, to avoid nausea associated with the supplement and perceived it as a standard practice to eat before consuming any medication. However, women reported that food insecurity was a critical barrier to calcium uptake	(13,27)	**Moderate Confidence**	Due to no or very minor concern with coherence, minor concerns on methodological limitations (reflexivity), moderate concerns on adequacy (2 out 6 studies contributed with 1 thick and 1 thin data), and moderate concerns on relevance (small number of countries)
**5. Strategies to improve use**				
**5.1**	**Implementation of reminders to promote adherence**<br>Women and health workers perceived reminders as beneficial in promoting women's adherence in consuming calcium. Several reminder strategies were deemed to be useful by women and health workers, such as home-based posters, calendars with illustrations and daily reminders, and integrating supplement consumption into the women's daily routine such as at mealtime	(11,13,26,27)	**High Confidence**	Due to no or very minor concerns on methodological limitations, no or very minor concerns on coherence, minor concerns on adequacy (small number of studies contributing to qualitative evidence synthesis), moderate concerns on relevance (due to all studies coming from low income or lower middle-income country and small number of countries)
**5.2**	**Importance of family support and adherence partner implementation**<br>Having family support was instrumental to women in adhering to calcium use and could be leveraged by notifying them on the importance of women's adherence to consumption and appointing someone to be woman's "adherence partner" to help remind woman in taking the supplement. Both women and health workers were positive about adherence partners in providing support in terms of encouraging them to take the supplements, providing food, helping them around the house, emotional support, improving family relationships, and increased partner or husband involvement in pregnancy	(11,13,26,28)	**High Confidence**	No or very minor concerns on methodological limitations, no or very minor concerns on coherence, minor concerns on adequacy (small number of studies contributing to qualitative evidence synthesis), and moderate concerns on relevance (due to all studies coming from low income or lower middle-income country only, 2 of 5 studies has indirectly relevant aim, and small number of countries)
**5.3**	**Counselling facilitates calcium uptake**<br>Both women and health workers and acknowledged that the counselling they received from health workers was a motivator to calcium uptake. Women valued the discussion they have with health workers and felt more confident to take calcium supplements when they received counselling on information of preeclampsia/eclampsia and the benefits of calcium from their health workers	(13,27)	**Moderate Confidence**	No or very minor concerns on methodological limitations, no or very minor concerns on coherence, moderate concerns on relevance (due to all studies coming from low or lower middle-income country and small number of countries) and moderate concerns on adequacy (2 out 6 studies contributed with thick data)
**6. Knowledge and training**				
**6.1**	**Varied knowledge about preeclampsia or eclampsia among health workers**<br>Health workers' knowledge about preeclampsia and eclampsia was variable among health workers, and some health workers felt that preeclampsia or eclampsia is not a priority health concern in their area and reported never having encountered any case	(26,27, 30)	**Moderate Confidence**	Due to no or very minor concerns on methodological limitations and coherence, moderate concerns on relevance (due to all studies coming from low or lower middle-income countries and small number of studies) and moderate concerns on adequacy (2 out 6 studies contributed with thick data)
**6.2**	**Inadequate training to diagnose and treat pre-eclampsia and eclampsia**<br>While some health workers mentioned that training about preeclampsia/eclampsia and calcium supplementation was adequate, others reported that their training lacked depth and continuity, and thus felt unprepared to diagnose and offer information about preeclampsia/eclampsia and calcium to pregnant women. Health workers expressed the needs to have more and continuous training to manage the condition and to address any concerns and resistance from the community	(13,26,27)	**High Confidence**	No or very minor concerns on methodological limitations, coherence, moderate concerns on relevance (due to all studies coming from low income or lower middle-income country and small number of countries) and minor concerns on adequacy (3 out of 6 studies contributed with 2 thick and 1 thin data)
**7. Beliefs on the intervention**				
**7.1**	**Perceived overmedicalization when prescribing calcium supplementation**<br>Both health workers and women perceived that prescribing more pills to general low-risk women during pregnancy was a form of over-medicalization of pregnancy. Some health workers, however, felt that calcium supplementation during pregnancy was a way to prevent further medicalization when complications occurred	(34)	**Low confidence**	No or very minor concerns on coherence, minor concerns on methodological limitations (reflexivity and ethics), serious concerns on relevance (evidence coming from high income country only), serious concerns on adequacy (1 out of 6 study contributed)
**7.2**	**Beliefs that pre-eclampsia is not a serious problem in their settings**<br>Health workers generally had positive beliefs about calcium supplementation and there was optimism that calcium could be delivered through antenatal care health workers. Some facilitators motivating health workers in prescribing calcium supplementation include belief in its prevention value and expected benefits, women liked the calcium supplements, and benefits experienced by women, and perceived lack of knowledge on how to treat preeclampsia which motivated health workers to err towards prevention	(13,27,34)	**High Confidence**	No or very minor concerns on coherence, minor concerns on relevance (small number of countries), minor concerns on methodological limitations (ethics and reflexivity), and minor concerns on adequacy (3 out of 6 studies contributed with 2 thick and 1 thin data)
**8. Structural factors**				
**8.1**	**Increased workload**<br>In general, health workers felt that their workload increased by	(13,27)	**Moderate Confidence**	No or very minor concerns on methodological limitations, no or very minor concerns on coherence, minor concerns

For peer review only - http://bmjopen.bmj.com/site/about/guidelines.xhtml

including calcium supplementation in the services they were providing to pregnant women. Health workers reported inadequate number of staff providing care, yet they needed to provide additional counselling and prescribing to women, especially pregnant women with comorbidities.	on relevance (due to all studies coming from low income or lower middle-income countries and small number of countries) and serious concerns on adequacy (2 out 6 studies contributed)

For peer review only

For peer review only - http://bmjopen.bmj.com/site/about/guidelines.xhtml

Women's knowledge and learning

Women's knowledge about pre-eclampsia

Most women had limited knowledge about pre-eclampsia, and these conditions were not typically considered by women to be a serious problem. Symptoms of pre-eclampsia such as swollen feet, severe headache, blurred vision and vomiting were considered normal signs and symptoms in pregnancy, while seizures were associated with evil attacks or nutritional deficiencies (Finding 1.1 – High confidence). (18,19,38) Women and healthcare providers from Ethiopia and Kenya stated that there was no local language for pre-eclampsia, which made it difficult for healthcare providers to explain the condition to women, and served as a barrier in providing adequate knowledge to women and encouraging them to use calcium supplements. (19,38)

Information provision to women

Women felt they did not receive adequate information during pregnancy from healthcare providers about pre-eclampsia and calcium supplements. They described wanting to be given more information, regardless of their pre-eclampsia risk status. Women believed that having this essential information could help them to make informed choices and actively participate in their care. There were, however, mixed opinions from healthcare providers - some feared that more information could generate anxiety for women, while others were more supportive of providing information (Finding 1.2 – Low confidence). (18,39) Healthcare providers were worried that sharing information about pre-eclampsia, especially with low-risk pregnant women, could lead to anxiety and make an "uncomplicated pregnancy more stressful". (18) Healthcare providers viewed their role as informants, but not as decision-makers for women. They believed that it should be woman's choice to decide whether to consume calcium supplements or not. (18) Women and healthcare providers mentioned that the scope of information provided to women should include symptoms of pre-eclampsia as well as effectiveness, benefits, and safety of calcium-containing supplements. (18)

Learning about calcium supplements

Women typically learned about dietary calcium, including pre-eclampsia symptoms, from healthcare providers. They considered healthcare providers to be the most trusted and reliable source of information and reported feeling confident about taking calcium-containing supplements after receiving adequate information from them. Women also appreciated receiving information on calcium supplements and pre-eclampsia via information, education, and communication (IEC) materials like videos, media campaigns, and trusted websites (Finding 1.3 – High confidence). (18,19,32,34,39) Quantitative evidence supported qualitative findings regarding women's learning on calcium supplements. In the context of an intervention implementing training to healthcare providers to reinforce calcium-related messages to women, women reported that they would take calcium in a future pregnancy and that they would recommend calcium to other pregnant women. (31)

For peer review only

Women were more likely to consume calcium supplements if they had higher knowledge of calcium benefits (Odds Ratio (OR) 11.7, 95% Confidence Interval (CI): 5.97-22.86) and higher general education (OR 2.59, 95% CI: 2.21-3.05). (32) Higher adherence was also reported in those with higher education (OR 1.45, 95% CI: CI 1.31 to 1.60).(36) Higher nutrition knowledge was also associated with taking 6 times more calcium tablets.(32)
One paper, evaluating calcium supplement intake before and after nutritional interventions, showed an association between maternal knowledge of nutrition and higher calcium supplement intake (β ~31.9, 95% CI: 20.9, 43.0), however the paper also highlighted there were still large gaps between knowledge and practices, as the intake of calcium supplement tablets during 6 month was low, 82 ±66 out of the recommended 180 tablets.(34)

Women's beliefs about calcium supplements in pregnancy

Fears about side effects as a barrier to calcium supplements uptake

Women's fears about the side effects of calcium supplements affected their adherence. Women highlighted that assurance of safe use of calcium supplement is a key facilitator to consistent use. However, some women felt safety was not assured by healthcare providers, especially when calcium supplements were perceived as "experimental". Women had also received messages from their families or communities that any pills consumed during pregnancy could be harmful. (Finding 2.1 – High confidence). (18,19,39,40) Quantitative evidence supported qualitative findings as few women reported being discouraged to take supplements by friends (4%) and elder women (3%).(28)

Women's experiences of side effects

Some women reported experiencing side effects after taking calcium and iron-folic acid supplements, such as dizziness, vomiting, nausea, stomach aches, loss of appetite, tiredness, diarrhoea, bloating, and burping, yet noted that side effects subsided with time. Women also reported that they continued taking calcium supplements despite these side effects (Finding 2.2 – High confidence). (19,39,40) Quantitative evidence supported the qualitative findings as some women reported experiencing side effects, usually related to gastrointestinal symptoms. (29–31,37) The reported side effect rates were usually low, 4% of women mentioning nausea or vomiting or constipation in one paper while in another paper 14.9% of women reported that side effects was the reason for missing a dose of IFA or calcium.(30,37) These could cause supplement discontinuation or erratic supplement intake for one to 10 days after side effects were felt in around 5% of women.(37)

Concerns about being stigmatized as HIV patients

Women expressed concerns that if they took calcium supplements, they could be stigmatized as HIV patients, which was a reported barrier to use. Some women were afraid of being stigmatized as their community often associated nutritional supplement intake and accompanying reminder posters, with HIV (Finding 2.3 – Moderate confidence). (18,39)

For peer review only - http://bmjopen.bmj.com/site/about/guidelines.xhtml
For peer review only

Positive perceptions of calcium supplements

Women reported that both their perceptions about expected benefits and previous experiences of taking calcium and iron-folic acid supplements were facilitators of use. Women believed that taking pills could compensate for sub-optimal nutrition during pregnancy, and that supplements during pregnancy would help keep their baby safe (Finding 2.4 – High confidence). (18,19,39–41) Women also reported reduced cravings to consume soil (pica) during pregnancy (a cultural practice or cravings characterised by recurrent ingestion of unusually high amounts of soil, and is related to iron deficiency anaemia, which is common during pregnancy). (39) Furthermore, some women appreciated the emphasis of "prevention" when encouraging supplement intake. (19,41) Quantitative evidence supported the qualitative findings that women's beliefs about the importance of calcium supplements to both woman's and baby's health are associated with calcium supplement intake. (32) Positive beliefs about calcium supplements and self-efficacy were associated with taking calcium supplements (OR 4.6, 95% CI: 2.0 to 10.5) and with taking a higher number of supplements (OR 2.77, 95% CI: 1.68 to 4.57). (32)

Calcium supplement characteristics and regimens

Varying preferences about characteristics of calcium tablets

Positive perceptions about the characteristics of the calcium tablet played a role in motivating women to take it. Some women preferred the chewable, sweet-tasting tablets that could be swallowed without water, while others preferred the hard tablets which were smaller in size, had no smell, and needed to be taken with water. Based on individual preference, the taste, smell, size, and convenience affected calcium supplement use (Finding 3.1 – Moderate confidence). (19,40)

Quantitative evidence supported qualitative findings regarding varying preferences calcium supplement's organoleptic properties. One paper which evaluate the impact of a program to implement calcium supplementation showed that most women (77%) reported preferences for conventional tablets that were easier to take and swallow, while the least preferred vehicle was unflavoured powder, as women dislike the taste.(30) Another paper reported that conventional tablets had an acceptable taste (83.9% of women).(31) Chewable tablets were preferred by most women (74%) in another paper. (40) Some characteristics that women considered while taking the supplements include tablet's flavour, chewable or swallow, taken with water or not, smell, and size.(30,31,40)

Supplement regimen as a barrier to use

Women described that they feel overwhelmed with the number of calcium tablets they had to take each day, especially women with other comorbidities who needed to take additional medications for their health conditions. Women felt that 3-4 pills per day at multiple times was onerous and preferred if they were combined into one pill (Finding 3.2 – High confidence). (19,39,40) In two quantitative studies, women preferred taking fewer tablets per

For peer review only - http://bmjopen.bmj.com/site/about/guidelines.xhtml

day. (30,40) However, those allocated to the study arm that used more frequent doses took more calcium overall. (40)

Daily routines and food insecurity

Adherence challenges due to routines

Adherence to calcium supplements was challenging for some women because of conflicting activities in their daily routine, such as taking other medications or supplements, traveling, being away from home, and household chores, which can lead them to forget to take calcium (Finding 4.1 – High confidence). (19,39,40) In quantitative evidence, women described busy work schedules and not being at home as also contributing to forgetting to consume their calcium supplements. (30,31,37) Forgetting to take calcium supplements was the most frequent reason (52.1%) for not taking IFA or calcium supplements and busy work schedules was inversely associated with adherence to calcium supplementation. (31,37)

Food insecurity as a barrier to calcium uptake

Women believed that adequate food was necessary to take the supplements and to avoid nausea. They perceived it as normal to eat before consuming any medication. However, women reported that food insecurity was a critical barrier to calcium uptake (Finding 4.2 – Moderate confidence). (19,39) Quantitative evidence supported qualitative findings that women with food security, high socio-economic status and living in urban areas are more likely to consume calcium supplements as compared to their counterparts.(34,36) Food security was associated with taking 6 more calcium tablets. (34)

Strategies to improve the use of calcium supplements

Implementation of reminders to promote adherence

Women and healthcare providers perceived reminders as beneficial in promoting women's adherence to calcium supplements. Several reminder strategies were described as useful by women and healthcare providers, such as home-based posters, calendars with illustrations and daily reminders, and integrating calcium supplement intake into women's daily routine, such as mealtimes (Finding 5.1 – High confidence). (19,38–40) Quantitative evidence supported the qualitative findings that distribution of behaviour change materials to women, such as pill-taking calendars, were associated with increased adherence. (40)

Importance of family support and 'adherence partner' implementation

Having family support was instrumental to pregnant women adhering to calcium supplements. This could be leveraged by notifying family members on the importance of calcium, and appointing someone to be an "adherence partner" or "pill buddy" to help remind her to take it. Both women and healthcare providers were positive about adherence partners in providing support in terms of encouraging them to take the supplements, providing food, helping them around the house, providing emotional support, improving family relationships,

For peer review only - http://bmjopen.bmj.com/site/about/guidelines.xhtml

and thereby increasing partner or husband involvement in pregnancy (Finding 5.2 – High confidence). (19,38–41) Women could choose who their adherence partner was, and some opted for their husband, a male or female relative, or their child. Some women reported that the support they received from adherence partners decreased over time (19,39–41), suggesting challenges with sustaining appropriate intake throughout pregnancy.

Quantitative evidence supported the qualitative findings that social support is important in encouraging women to take calcium supplements. (28,32,34,35,40) Women considered involving a husband, partner, or family in education sessions or appointing someone as the "adherence partner" to be an acceptable strategy for promoting adherence. (28,32,34,35) Women often chose their husband (52%), older female relative (23%), children (14%), cousins or other relatives as adherence partners (8%) (28), and were satisfied with the reminders and support received from their adherence partner. (28,32,34) However, a randomised trial assessing adherence partners in improving calcium supplement intake showed that high social support, instead of adherence partners alone, was associated with higher adherence to calcium supplements (OR: 2.10; 95% CI: 1.32, 3.34). (28) Women with high family support reported higher intake of calcium supplements (OR = 2.1).(32)

Counselling facilitates calcium supplements uptake

Both women and healthcare providers acknowledged that counselling women on the benefits of calcium was a motivator to calcium supplement intake. Women valued the discussion they have with healthcare providers and felt more confident to take calcium supplements when they received counselling and information and pre-eclampsia and the benefits of calcium from their healthcare providers (Finding 5.3 – Moderate confidence). (19,39) This was confirmed by healthcare providers who reported that they have seen positive results following counselling women on iron-folic acid supplements and believed that this would be replicated for calcium-containing supplements. (39)

Quantitative evidence extended qualitative findings where not only counselling, but also starting antenatal contacts at early gestational age, higher number of antenatal contacts, and receiving free calcium supplements were associated with higher calcium intake by women. (31,32,34,36) One paper reports that women were 59 times more likely to consume calcium supplements if they had received them for free.(32)

Healthcare provider factors

Healthcare provider knowledge and training

Varied knowledge about pre-eclampsia among healthcare providers

Healthcare providers' knowledge about pre-eclampsia was varied. Some felt that pre-eclampsia is not a priority health concern in their area and reported never having encountered any case (Finding 6.1 – Moderate confidence). (19,38,42)

Inadequate training to diagnose and treat pre-eclampsia

While some healthcare providers mentioned that training about pre-eclampsia and calcium supplements was adequate, others reported that their training lacked depth and continuity, and thus felt unprepared to diagnose it and offer information these conditions and the use of calcium for prevention. Healthcare providers expressed the need to have more and continuous training on managing pre-eclampsia, as well as time to address concerns or resistance from the community (Finding 6.2 – High confidence). (19,38,39) Healthcare providers had positive views of trainings and IEC materials and felt that it helps improve their knowledge. (42) They also valued supervision visits which help them solve problems and increases their accountability. (42) Support was also needed throughout calcium roll-out to ensure challenges can be addressed promptly during implementation. (19)

Beliefs about the intervention

Perceived overmedicalization when prescribing calcium supplements

Both healthcare providers and women perceived that prescribing more tablets to “low-risk” women during pregnancy was a form of over-medicalization of pregnancy. However, some healthcare providers felt that calcium supplements were a way to prevent further medicalization due to pre-eclampsia-related complications (Finding 7.1 – Low confidence). (18)

Beliefs about calcium supplements

Healthcare providers generally had positive beliefs about calcium supplements and there was optimism that calcium could be delivered through antenatal care healthcare providers. Some facilitators motivating healthcare providers to prescribe calcium supplements included their beliefs in its prevention value and expected benefits, that women liked the calcium supplements and experienced benefits from it, and a perceived lack of knowledge on how to treat pre-eclampsia which motivated healthcare providers to side towards prevention (Finding 7.2 – High confidence). (18,19,39)

Structural factors

High workload, inadequate staffing, stock out, and lack of equipment

In general, healthcare providers felt that their workload increased by including calcium supplements in antenatal care for pregnant women. Healthcare providers reported existing inadequate staffing, yet they needed to provide additional counselling and prescription to women, especially pregnant women with comorbidities (Finding 8.1 – Moderate confidence). (19,39) Stock-outs were also reported as critical barriers, often due to logistical issues in the supply chain (e.g., centralization or procurement changes), errors in demand estimation by government staff, and inadequate storage facilities. (42) Importantly, some healthcare providers reported that a lack of equipment to diagnose pre-eclampsia was a barrier to

For peer review only - http://bmjopen.bmj.com/site/about/guidelines.xhtml

calcium implementation .(19) Facility staff members and supervisors reported that utilisation of health information systems to monitor calcium stocks, checklists to provide feedback on counselling, and gaps to address, as well as collaboration with government staff members, could be facilitators of use. (42)

Quantitative evidence extended qualitative findings by showing that in the context of a comprehensive integrated program including the implementation of job aids, training, guidelines, monitoring, and feedback session for healthcare providers, could overcome barriers in prescribing women with calcium supplements. (33)

Mapping to behaviour change models

Through COM-B and TDF mapping (Appendix 7), we identified that the critical domains on facilitators and barriers to improve calcium use among women, which include: knowledge, beliefs about consequences, beliefs about capabilities, emotion, social influences, and environmental context and resources to improve calcium use by women. To encourage calcium prescription by healthcare providers, facilitators and barriers related to knowledge, skills, beliefs about consequences and environmental context domains should be addressed. Figure 2 shows the categorisation of barriers and facilitators across the Capability, Opportunity and Motivation Behaviour. The mapping shows that factors encouraging women's use of and adherence to calcium includes receiving adequate information about pre-eclampsia through counselling with healthcare providers and IEC materials, assurance of calcium safety, receiving preferred characteristics of tablet and doses, family and community support, early and frequent antenatal contacts, free calcium supplements, and reminder tools distribution. Likewise, factors that may encourage healthcare providers to prescribe calcium supplements include continuous training about identification and management of pre-eclampsia and calcium supplements, dissemination of consistent messages, reminders, and ensuring adequate number of human resources, equipment for diagnosing pre-eclampsia, and availability of calcium pills at health facilities.

DISCUSSION

We included 18 papers from 16 studies conducted primarily in LMICs and reporting views of women, adherence partners, and healthcare providers. Our review shows the importance of healthcare providers' knowledge and training about calcium supplements and pre-eclampsia, as women reported providers as the most reliable sources of information and reassurance on safety of calcium and would encourage adherence. Promoting early initiation of antenatal care and consistent messages on pre-eclampsia and calcium supplementation may improve women's use of calcium supplements. Free calcium supplements and options on doses and tablet preferences could help overcome barriers to calcium supplement use for women. Reminder systems and support from family and community may also help increase women´s calcium uptake.

Women play an important role in decision-making about calcium supplement during pregnancy. Our review shows that women's limited knowledge about and fears of side effects, and potential impacts on their baby serve as the critical barriers to calcium

For peer review only - http://bmjopen.bmj.com/site/about/guidelines.xhtml

supplement intake. These barriers about calcium supplementation have been reported on the use of other supplements during pregnancy. For example, studies on factors affecting multiple micronutrient supplementation, iron folic acid, and lipid-based nutrients reported women's knowledge, acceptance, motivation, and attitudes toward the medication play an important role. (43,44) Furthermore, a study on factors affecting use of intervention for preterm management also reported women felt hesitant in consuming the medications to improve labour outcome due to fears about baby's growth and development.(45) This highlights the need to ensure that women are aware on the benefits of the supplement and given assurance on the safety of its use.

As the most trusted informants, healthcare providers knowledge about calcium and pre-eclampsia are important to support women uptake of calcium supplements. Studies reported different levels of knowledge about calcium and pre-eclampsia among providers, with some providers having persistent beliefs that evidence on calcium supplements during pregnancy is still in the experimental phase. Some healthcare providers might be reluctant to talk about pre-eclampsia with the fear to stress low risk women; however, this reluctance should be balanced by the risk of symptoms of pre-eclampsia going unnoticed by women. Reinforcing messages related to improving pregnancy outcomes and similarities to other supplements taken during pregnancy such as folic acid and iron that also help to may facilitate use of calcium supplements. Healthcare providers also need to know about how to assess eligibility for calcium supplements, including how to screen and score women at high-risk and to identify populations with low calcium intake. There is lack of acceptable biomarkers of individual calcium intake and calcium status, which complicates screening individuals.(46) WHO recommendations on calcium supplementation were set for populations with low calcium intake, as dietary assessments are more reliable to identify populations with low calcium intake rather than to identify individuals. (46)

Human resource shortages are a recurrent health system challenge, particularly in LMICs which results in overburdened staff unable to deliver recommended practices. Appropriate staffing, particularly of midwives and nurses who provide most antenatal care services, remains crucial to achieve quality of care. Unfortunately, this is also applicable on the context of other medication delivered during pregnancy. For example, providers reported unavailability of stock, inadequate staffs and equipment as the main barriers in prescribing interventions to pregnant women experiencing preterm labour. Therefore, ensuring availability of diagnostic tools and calcium stocks is critical to ensure appropriate prescription and delivery of care.(45) Where health providers constraints persist, innovative strategies to streamline antenatal care practices may be needed to improve efficiency.(47)

Implications for research, policy, and practice

The TDF and COM-B mapping in our review can be used by researchers and programme managers to inform the development of implementation models to optimise the use of calcium supplements. Assessing the extent to which the barriers and facilitators to calcium prescription and use identified in our review are potential implementation challenges in different contexts can be a useful starting point for formative research to scale-up implementation. Table 3 presents a list of questions derived from our findings and may help programme implementers, policymakers, researchers, and other stakeholders to identify

For peer review only - http://bmjopen.bmj.com/site/about/guidelines.xhtml

and address factors that may affect prescription and use of calcium supplements during pregnancy. Assessing the extent to which the barriers and facilitators identified in our review are potential implementation challenges in different settings is a useful starting point for formative research to scale this intervention.

Table 3 Implications for research, policy, and practice

Domain	List of questions
**Guidelines and protocols**	1. Are guidelines and clinical protocols on pre-eclampsia/eclampsia and calcium supplements during pregnancy consistent between WHO, national, and facility-levels?
**Knowledge and learning**	2. Do women or healthcare providers have scepticism or concerns about adverse effects of calcium supplements during pregnancy that can be addressed?<br>3. Do women and their family members receive education and educational materials about signs of pre-eclampsia/eclampsia early in pregnancy?<br>4. Do women have sufficient time and opportunity to discuss pre-eclampsia/eclampsia with healthcare providers during antenatal care?<br>5. Do women have sufficient time and opportunity to discuss calcium supplementation with healthcare providers during antenatal care, including addressing fears about side effects, managing side effects, safety concerns, and reinforcing positive messaging about expected benefits?<br>6. Have concerns from both women and healthcare providers about calcium supplements as a form of overmedicalisation of pregnancy been addressed in culturally-appropriate ways?<br>7. Have healthcare providers received in-service training on pre-eclampsia/eclampsia prevention and management, including the importance of calcium supplements during pregnancy for prevention?
**Strategies to improve use**	8. Do all relevant cadres of healthcare providers (including midwives and nurses) have authority to prescribe calcium supplements during pregnancy?<br>9. Do women have the opportunity to try different types of calcium tablets to suit their preferences, such as chewable/non-chewable, different tastes, and different size tablets?<br>10. Do women have the opportunity to try different calcium dosing combinations to suit their schedules and preferences?<br>11. For women experiencing or at risk of food insecurity during pregnancy, are there additional social services to support adequate nutrition intake during pregnancy?<br>12. Have different types of reminder systems (e.g., posters for home, calendars, and integration into daily routines) been designed with women and their families to encourage use?<br>13. Has support from family and/or adherence partners been integrated for women, and do family members or adherence partners have the opportunity to attend educational sessions?<br>14. Are stocks of calcium readily available in the antenatal care wards?<br>15. Is there sufficient funding and budget allocation to ensure continuous procurement and distribution of calcium supplements?

Strengths and limitations

Most included studies were from low- and middle-income countries and almost half came from the same project conducted in Kenya and Ethiopia, which may limit transferability of results to other contexts. The results from our review therefore can be used to guide formative research as well as implementation and programme planning in other contexts.

For peer review only - http://bmjopen.bmj.com/site/about/guidelines.xhtml

Most included studies engaged women who attended antenatal care; therefore, might not be representative of those that do not reach the health system during pregnancy. None of the included studies reported the perspectives of policymakers.

Despite these limitations, this is the first systematic review of barriers and facilitators to calcium supplement use during pregnancy. We adopted a mixed methods approach which allowed for inclusion and consolidation of studies with a range of designs. Mapping the review findings to behaviour change models improved understanding of how barriers and facilitators influence calcium uptake, and consequently can be addressed in future interventional or programmatic work.

Conclusion

Our review identified a range of barriers and facilitators affecting calcium supplements during pregnancy to prevent pre-eclampsia. When formulating intervention and policies on calcium supplement use, relevant stakeholders should consider the identified barriers and facilitators to optimise uptake. Findings from this study can inform implementation considerations, to ensure effective and equitable provision and scale-up of calcium interventions.

Funding: This work was funded by the Medical Research Council [grant number MR/T038861/1] and the UNDP-UNFPA-UNICEF-WHO-World Bank Special Programme of Research, Development and Research Training in Human Reproduction (HRP), a cosponsored programme executed by the World Health Organization (WHO). MAB's time is supported by an Australian Research Council Discovery Early Career Researcher Award (DE200100264) and a Dame Kate Campbell Fellowship (University of Melbourne Faculty of Medicine, Dentistry and Health Sciences) and Programa PROINCE-UNLaM-CyTMA SAL-077.

Competing interests: None declared

Ethics: Ethical approval was not required; no primary data were collected.

Patient consent for publication: Not required.

Contributors: All authors have made substantial contributions as outlined below. Conceptualisation: ST, APB, MAB, JA and GC. Data curation: TR, JA, GC, HM and RIZ. Formal analysis: RIZ, GC, HM and MAB. Funding acquisition: ST, APB and JA. Investigation and methodology: RIZ, MAB, GC and HM. Writing – original draft: GC, HM, RIZ and MAB. Writing – review and editing: GC, HM, RIZ, JA, TR, JPP, ZQ, GJH, HM, LS, JPV, AP, GNG, EA, KKL, GC, RR, KS, TA, TY, APB, TS and MAB.

Data availability statement: All data relevant to the study are included in the article or uploaded as supplementary information.

For peer review only - http://bmjopen.bmj.com/site/about/guidelines.xhtml

For peer review only

For peer review only

For peer review only - http://bmjopen.bmj.com/site/about/guidelines.xhtml

References

1. Poon LC, Shennan A, Hyett JA, Kapur A, Hadar E, Divakar H, et al. The International Federation of Gynecology and Obstetrics (FIGO) initiative on pre-eclampsia: A pragmatic guide for first-trimester screening and prevention. Int J Gynecol Obstet. 2019;145(S1).

2. Mol BWJ, Roberts CT, Thangaratinam S, Magee LA, De Groot CJM, Hofmeyr GJ. Pre-eclampsia. In: The Lancet. 2016. p. 999–1011.

3. Gestational Hypertension and Preeclampsia: ACOG Practice Bulletin, Number 222. Obstet Gynecol. 2020;135(6).

4. Say L, Chou D, Gemmill A, Tunçalp Ö, Moller A-B, Daniels J, et al. Global causes of maternal death: a WHO systematic analysis. Lancet Glob Heal [Internet]. 2014 Jun;2(6):e323–33. Available from: https://linkinghub.elsevier.com/retrieve/pii/S2214109X1470227X

5. Steegers EAP, von Dadelszen P, Duvekot JJ, Pijnenborg R. Pre-eclampsia. Lancet [Internet]. 2010 Aug;376(9741):631–44. Available from: https://linkinghub.elsevier.com/retrieve/pii/S0140673610602796

6. Prevention and treatment of pre-eclampsia and eclampsia. World Health. 2011.

7. Hofmeyr GJ, Lawrie TA, Atallah ÁN, Torloni MR. Calcium supplementation during pregnancy for preventing hypertensive disorders and related problems. Vol. 2018, Cochrane Database of Systematic Reviews. 2018.

8. WHO recommendation: Calcium supplementation during pregnancy for the prevention of pre-eclampsia and its complications. Geneva; 2018.

9. WHO recommendation on Calcium supplementation before pregnancy for the prevention of pre-eclampsia and its complications. Geneva; 2020.

10. WHO Recommendations on Antenatal Care for a Positive Pregnancy Experience. Geneva; 2013.

11. Omotayo MO, Dickin KL, O'Brien KO, Neufeld LM, De Regil LM, Stoltzfus RJ. Calcium Supplementation to Prevent Preeclampsia: Translating Guidelines into Practice in Low-Income Countries. Adv Nutr. 2016;7(2):275–8.

12. Page MJ, McKenzie JE, Bossuyt PM, Boutron I, Hoffmann TC, Mulrow CD, et al. The PRISMA 2020 statement: An updated guideline for reporting systematic reviews. Vol. 372, The BMJ. 2021.

13. Tong A, Flemming K, McInnes E, Oliver S, Craig J. Enhancing transparency in reporting the synthesis of qualitative research: ENTREQ. BMC Med Res Methodol. 2012;12.

14. Glenton C, Lewin S, Downe S, Paulsen E, Munabi-Babigumira S, Johansen M, et al. Qualitative Evidence Syntheses Within Cochrane Effective Practice and Organisation of Care: Developing a Template and Guidance. Int J Qual Methods. 2021;20.

For peer review only - http://bmjopen.bmj.com/site/about/guidelines.xhtml

15. Covidence systematic review software. Melbourne, Australia: Veritas Health Innovation;

16. Hong QN, Fàbregues S, Bartlett G, Boardman F, Cargo M, Dagenais P, et al. The Mixed Methods Appraisal Tool (MMAT) version 2018 for information professionals and researchers. Educ Inf. 2018;34(4).

17. Thomas J, Harden A. Methods for the thematic synthesis of qualitative research in systematic reviews. BMC Med Res Methodol [Internet]. 2008 Dec 10;8(1):45. Available from: https://bmcmedresmethodol.biomedcentral.com/articles/10.1186/1471-2288-8-45

18. Vestering A, Bekker MN, Grobbee DE, van der Graaf R, Franx A, Crombag NMT, et al. Views and preferences of medical professionals and pregnant women about a novel primary prevention intervention for hypertensive disorders of pregnancy: a qualitative study. Reprod Health [Internet]. 2019 May 2;16(1):46. Available from: http://www.ncbi.nlm.nih.gov/pubmed/31046778

19. Birhanu Z, Chapleau GM, Ortolano SE, Mamo G, Martin SL, Dickin KL. Ethiopian women's perspectives on antenatal care and iron-folic acid supplementation: Insights for translating global antenatal calcium guidelines into practice. Matern Child Nutr [Internet]. 2018 Feb;14:e12424. Available from: https://onlinelibrary.wiley.com/doi/10.1111/mcn.12424

20. Michie S, van Stralen MM, West R. The behaviour change wheel: A new method for characterising and designing behaviour change interventions. Implement Sci [Internet]. 2011 Dec 23;6(1):42. Available from: http://implementationscience.biomedcentral.com/articles/10.1186/1748-5908-6-42

21. Atkins L, Francis J, Islam R, O'Connor D, Patey A, Ivers N, et al. A guide to using the Theoretical Domains Framework of behaviour change to investigate implementation problems. Implement Sci [Internet]. 2017 Dec 21;12(1):77. Available from: http://implementationscience.biomedcentral.com/articles/10.1186/s13012-017-0605-9

22. Lewin S, Bohren M, Rashidian A, Munthe-Kaas H, Glenton C, Colvin CJ, et al. Applying GRADE-CERQual to qualitative evidence synthesis findings—paper 2: how to make an overall CERQual assessment of confidence and create a Summary of Qualitative Findings table. Implement Sci [Internet]. 2018 Jan 25;13(S1):10. Available from: https://implementationscience.biomedcentral.com/articles/10.1186/s13012-017-0689-2

23. Munthe-Kaas H, Bohren MA, Glenton C, Lewin S, Noyes J, Tunçalp Ö, et al. Applying GRADE-CERQual to qualitative evidence synthesis findings—paper 3: how to assess methodological limitations. Implement Sci [Internet]. 2018 Jan 25;13(S1):9. Available from: https://implementationscience.biomedcentral.com/articles/10.1186/s13012-017-0690-9

24. Colvin CJ, Garside R, Wainwright M, Munthe-Kaas H, Glenton C, Bohren MA, et al. Applying GRADE-CERQual to qualitative evidence synthesis findings—paper 4: how to assess coherence. Implement Sci [Internet]. 2018 Jan 25;13(S1):13. Available from: https://implementationscience.biomedcentral.com/articles/10.1186/s13012-017-0691-8

For peer review only - http://bmjopen.bmj.com/site/about/guidelines.xhtml

25. Glenton C, Carlsen B, Lewin S, Munthe-Kaas H, Colvin CJ, Tunçalp Ö, et al. Applying GRADE-CERQual to qualitative evidence synthesis findings—paper 5: how to assess adequacy of data. Implement Sci [Internet]. 2018 Jan 25;13(S1):14. Available from: https://implementationscience.biomedcentral.com/articles/10.1186/s13012-017-0692-7

26. Noyes J, Booth A, Lewin S, Carlsen B, Glenton C, Colvin CJ, et al. Applying GRADE-CERQual to qualitative evidence synthesis findings–paper 6: how to assess relevance of the data. Implement Sci [Internet]. 2018 Jan 25;13(S1):4. Available from: https://implementationscience.biomedcentral.com/articles/10.1186/s13012-017-0693-6

27. Bora K, Barman B, Pala S, Das A, Doke G, Tripura A. Coverage of antenatal iron-folic acid and calcium distribution during pregnancy and their contextual determinants in the northeastern region of India. Front Nutr [Internet]. 2022 Jul 18;9. Available from: https://www.frontiersin.org/articles/10.3389/fnut.2022.894245/full

28. Martin SL, Omotayo MO, Pelto GH, Chapleau GM, Stoltzfus RJ, Dickin KL. Adherence-specific social support enhances adherence to calcium supplementation regimens among pregnant women. J Nutr. 2017;147(4).

29. Ghosh-Jerath S, Devasenapathy N, Singh A, Shankar A, Zodpey S. Ante natal care (ANC) utilization, dietary practices and nutritional outcomes in pregnant and recently delivered women in urban slums of Delhi, India: an exploratory cross-sectional study. Reprod Health [Internet]. 2015 Dec 20;12(1):20. Available from: https://reproductive-health-journal.biomedcentral.com/articles/10.1186/s12978-015-0008-9

30. Baxter J-AB, Roth DE, Al Mahmud A, Ahmed T, Islam M, Zlotkin SH. Tablets Are Preferred and More Acceptable Than Powdered Prenatal Calcium Supplements among Pregnant Women in Dhaka, Bangladesh. J Nutr [Internet]. 2014 Jul 1;144(7):1106–12. Available from: https://academic.oup.com/jn/article/144/7/1106/4569766

31. Thapa K, Sanghvi H, Rawlins B, Karki YB, Regmi K, Aryal S, et al. Coverage, compliance, acceptability and feasibility of a program to prevent pre-eclampsia and eclampsia through calcium supplementation for pregnant women: An operations research study in one district of Nepal. BMC Pregnancy Childbirth. 2016;

32. Nguyen PH, Kachwaha S, Avula R, Young M, Tran LM, Ghosh S, et al. Maternal nutrition practices in Uttar Pradesh, India: Role of key influential demand and supply factors. Matern Child Nutr [Internet]. 2019 Oct 18;15(4). Available from: https://onlinelibrary.wiley.com/doi/10.1111/mcn.12839

33. Omotayo MO, Dickin KL, Pelletier DL, Martin SL, Kung'u JK, Stoltzfus RJ. Feasibility of integrating calcium and iron-folate supplementation to prevent preeclampsia and anemia in pregnancy in primary healthcare facilities in Kenya. Matern Child Nutr [Internet]. 2018 Feb;14:e12437. Available from: https://onlinelibrary.wiley.com/doi/10.1111/mcn.12437

34. Nguyen PH, Sanghvi T, Kim SS, Tran LM, Afsana K, Mahmud Z, et al. Factors influencing

For peer review only

maternal nutrition practices in a large scale maternal, newborn and child health program in Bangladesh. de Souza RJ, editor. PLoS One [Internet]. 2017 Jul 10;12(7):e0179873. Available from: https://dx.plos.org/10.1371/journal.pone.0179873

35. Nguyen PH, Frongillo EA, Sanghvi T, Wable G, Mahmud Z, Tran LM, et al. Engagement of Husbands in a Maternal Nutrition Program Substantially Contributed to Greater Intake of Micronutrient Supplements and Dietary Diversity during Pregnancy: Results of a Cluster-Randomized Program Evaluation in Bangladesh. J Nutr [Internet]. 2018 Aug 1;148(8):1352–63. Available from: https://academic.oup.com/jn/article/148/8/1352/5040613

36. Liu D, Cheng Y, Dang S, Wang D, Zhao Y, Li C, et al. Maternal adherence to micronutrient supplementation before and during pregnancy in Northwest China: a large-scale population-based cross-sectional survey. BMJ Open [Internet]. 2019 Aug 8;9(8):e028843. Available from: https://bmjopen.bmj.com/lookup/doi/10.1136/bmjopen-2018-028843

37. Shakya Shrestha S, Adhikari R, Tamrakar S, Shrestha R, Shrestha A. Adherence to Iron, Folic Acid and Calcium Supplement and Factors Affecting it among the Antenatal Care Attending Women in a Tertiary Care Hospital: A Cross Sectional Study. Kathmandu Univ Med J (KUMJ) [Internet]. 18(70):186–92. Available from: http://www.ncbi.nlm.nih.gov/pubmed/33594028

38. Martin SL, Seim GL, Wawire S, Chapleau GM, Young SL, Dickin KL. Translating formative research findings into a behaviour change strategy to promote antenatal calcium and iron and folic acid supplementation in western Kenya. Matern Child Nutr [Internet]. 2017 Jan 22;13(1):mcn.12233. Available from: https://onlinelibrary.wiley.com/doi/10.1111/mcn.12233

39. Martin SL, Wawire V, Ombunda H, Li T, Sklar K, Tzehaie H, et al. Integrating Calcium Supplementation into Facility-Based Antenatal Care Services in Western Kenya: A Qualitative Process Evaluation to Identify Implementation Barriers and Facilitators. Curr Dev Nutr [Internet]. 2018 Nov 1;2(11). Available from: https://academic.oup.com/cdn/article/doi/10.1093/cdn/nzy068/5078341

40. Omotayo MO, Martin SL, Stoltzfus RJ, Ortolano SE, Mwanga E, Dickin KL. With adaptation, the WHO guidelines on calcium supplementation for prevention of pre-eclampsia are adopted by pregnant women. Matern Child Nutr [Internet]. 2018 Apr;14(2):e12521. Available from: http://doi.wiley.com/10.1111/mcn.12521

41. Martin SL, Omotayo MO, Chapleau GM, Stoltzfus RJ, Birhanu Z, Ortolano SE, et al. Adherence partners are an acceptable behaviour change strategy to support calcium and iron-folic acid supplementation among pregnant women in Ethiopia and Kenya. Matern Child Nutr [Internet]. 2017 Jul;13(3):e12331. Available from: https://onlinelibrary.wiley.com/doi/10.1111/mcn.12331

42. Kachwaha S, Nguyen PH, Tran LM, Avula R, Young MF, Ghosh S, et al. Specificity Matters: Unpacking Impact Pathways of Individual Interventions within Bundled Packages Helps Interpret the Limited Impacts of a Maternal Nutrition Intervention in India. J Nutr. 2022

For peer review only

Feb;152(2):612–29.

43. Clermont A, Kodish S, Matar Seck A, Salifou A, Rosen J, Grais R, et al. Acceptability and Utilization of Three Nutritional Supplements during Pregnancy: Findings from a Longitudinal, Mixed-Methods Study in Niger. Nutrients [Internet]. 2018 Aug 12;10(8):1073. Available from: http://www.mdpi.com/2072-6643/10/8/1073

44. Garcia-Casal MN, Estevez D, De-Regil LM. Multiple micronutrient supplements in pregnancy: Implementation considerations for integration as part of quality services in routine antenatal care. Objectives, results, and conclusions of the meeting. Matern Child Nutr [Internet]. 2018 Dec;14:e12704. Available from: https://onlinelibrary.wiley.com/doi/10.1111/mcn.12704

45. Zahroh RI, Hazfiarini A, Eddy KE, Vogel JP, Tunçalp Özge, Minckas N, et al. Factors influencing appropriate use of interventions for management of women experiencing preterm birth: A mixed-methods systematic review and narrative synthesis. Stock SJ, editor. PLOS Med [Internet]. 2022 Aug 23;19(8):e1004074. Available from: https://dx.plos.org/10.1371/journal.pmed.1004074

46. (EFSA) EFSA. Scientific Opinion on Dietary Reference Values for calcium. EFSA J. 2015;13(5):1–82.

47. Betrán AP, Bergel E, Griffin S, Melo A, Nguyen MH, Carbonell A, et al. Provision of medical supply kits to improve quality of antenatal care in Mozambique: a stepped-wedge cluster randomised trial. Lancet Glob Heal [Internet]. 2018 Jan;6(1):e57–65. Available from: https://linkinghub.elsevier.com/retrieve/pii/S2214109X17304217

FIGURE TITLES/LEGENDS

Figure 1 PRISMA flow diagram

PRISMA flow chart illustrating the number of records included and excluded at various screening and reviewing steps, leading to final list of records for data extraction and meta-analysis.

Figure 2 Capability, Opportunity and Motivation Model of Behaviour

Categorisation of barriers and facilitators across the Capability, Opportunity and Motivation model of Behaviour

For peer review only

PRISMA 2020 flow diagram for new systematic reviews which included searches of databases, registers, and other sources

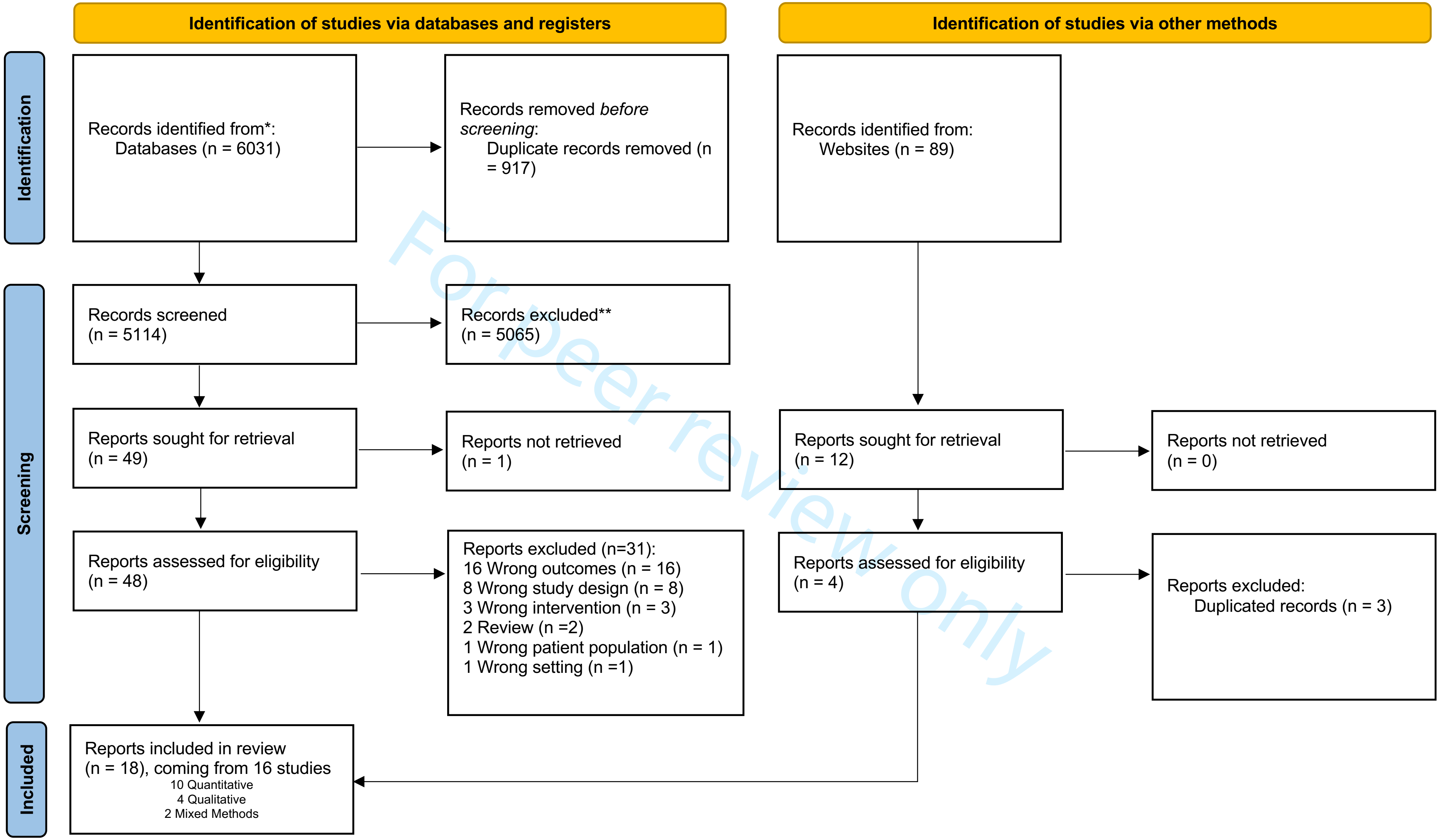

*Consider, if feasible to do so, reporting the number of records identified from each database or register searched (rather than the total number across all databases/registers).
**If automation tools were used, indicate how many records were excluded by a human and how many were excluded by automation tools.

From: Page MJ, McKenzie JE, Bossuyt PM, Boutron I, Hoffmann TC, Mulrow CD, et al. The PRISMA 2020 statement: an updated guideline for reporting systematic reviews. BMJ 2021;372:n71. doi: 10.1136/bmj.n71. For more information, visit: http://www.prisma-statement.org/

For peer review only

Capability in using interventions

Barriers

- Limited knowledge about preeclampsia/eclampsia†
- Symptoms were believed to be linked to evil attacks or nutritional deficiencies†
- Inadequate information on preeclampsia/eclampsia and calcium from providers†
- Varied knowledge on pre-eclampsia/eclampsia ¥
- In continuity and lack of depth training about pre-eclampsia/eclampsia ¥
- Felt inadequately trained ¥

Facilitators

- High knowledge of calcium supplementation†
- High education of women†
- Receive adequate information about condition and calcium from their providers†
- Counselling on information about preeclampsia and calcium from their providers†
- Continuous training to manage the condition and address resistance from community ¥
- Regular supervision visits for troubleshooting ¥

Motivation in using interventions

Barriers

- Pre-eclampsia/eclampsia was not considered as a serious problem†
- Experiences and fears of side effects†
- Safety of calcium was not assured†
- Felt that 3-4 pills per day at multiple times were too many†
- Inconvenience in taking pill daily†
- Perceived over-medicalization†¥
- Belief that preeclampsia/eclampsia was not a priority health concern ¥
- Fears in generating anxiety on low-risk women ¥

Facilitators

- Assurance of calcium safety†
- Experienced and expected benefits from taking calcium and folic acid†
- Valued information, education and communication (IEC) materials†
- Positive perceptions on tablet characteristics (smell, size, taste) †
- Belief that one combined pill per day could ease burden†
- Belief that having information would help making informed decisions†
- Belief that providers is a reliable source of information†
- Belief of being able to consume calcium daily†
- Feeling confident taking calcium after counselling provider†
- Positive belief about calcium supplementation benefits¥
- Belief that women should receive information regardless risk ¥

Opportunity in using interventions

Barriers

- Discouragement by family, neighbors and community in taking calcium†
- Stigmatization of having supplements and posters with HIV†
- Conflicting daily routine with taking calcium†
- Food insecurity†
- Providers felt that providing calcium would increase workload ¥
- Inadequate number of staff providing care ¥
- Stock out of supplements ¥
- Lack of equipment to diagnose ¥

Facilitators

- Family support in consuming calcium†
- Positive belief and experiences about 'adherence partners' †¥
- Early initiation and frequent antenatal visits †
- Universal free calcium distribution through antenatal care †
- Reminder tools distribution, such posters and pill-taking calendars †¥
- Adequate calcium supplement stock and its storage ¥
- Provision of equipment to diagnose pre-eclampsia ¥
- Adequate number of human resources at health facility ¥
- Comprehensive integrated program (job aids, training, guidelines, regular supplies) ¥

Behaviour: Calcium supplementation prescription by providers and use by women

†: woman's factors; ¥: providers factors

For peer review only - http://bmjopen.bmj.com/site/about/guidelines.xhtml

Appendix 1. PRISMA 2020 Main Checklist

Topic	No.	Item	Location where item is reported
**TITLE**			
**Title**	1	Identify the report as a systematic review.	Title
**ABSTRACT**			
**Abstract**	2	See the PRISMA 2020 for Abstracts checklist	Abstract
**INTRODUCTION**			
**Rationale**	3	Describe the rationale for the review in the context of existing knowledge.	Introduction
**Objectives**	4	Provide an explicit statement of the objective(s) or question(s) the review addresses.	Introduction
**METHODS**			
**Eligibility criteria**	5	Specify the inclusion and exclusion criteria for the review and how studies were grouped for the syntheses.	Methods –Topic of interest and type of studies
**Information sources**	6	Specify all databases, registers, websites, organisations, reference lists and other sources searched or consulted to identify studies. Specify the date when each source was last searched or consulted.	Methods – Search methods for identification of studies
**Search strategy**	7	Present the full search strategies for all databases, registers and websites, including any filters and limits used.	Appendix 3
**Selection process**	8	Specify the methods used to decide whether a study met the inclusion criteria of the review, including how many reviewers screened each record and each report retrieved, whether they	Methods – Selection of studies

For peer review only - http://bmjopen.bmj.com/site/about/guidelines.xhtml

		worked independently, and if applicable, details of automation tools used in the process.	
**Data collection process**	9	Specify the methods used to collect data from reports, including how many reviewers collected data from each report, whether they worked independently, any processes for obtaining or confirming data from study investigators, and if applicable, details of automation tools used in the process.	Methods – Data extraction and assessing the methodological limitations
**Data items**	10a	List and define all outcomes for which data were sought. Specify whether all results that were compatible with each outcome domain in each study were sought (e.g. for all measures, time points, analyses), and if not, the methods used to decide which results to collect.	Methods – Data extraction and assessing the methodological limitations
	10b	List and define all other variables for which data were sought (e.g. participant and intervention characteristics, funding sources). Describe any assumptions made about any missing or unclear information.	Methods – Data extraction and assessing the methodological limitations
**Study risk of bias assessment**	11	Specify the methods used to assess risk of bias in the included studies, including details of the tool(s) used, how many reviewers assessed each study and whether they worked independently, and if applicable, details of automation tools used in the process.	Methods – Data extraction and assessing the methodological limitations
**Effect measures**	12	Specify for each outcome the effect measure(s) (e.g. risk ratio, mean difference) used in the synthesis or presentation of results.	Not applicable
**Synthesis methods**	13a	Describe the processes used to decide which studies were eligible for each synthesis (e.g. tabulating the study intervention characteristics and comparing against the planned groups for each synthesis (item 5)).	Not applicable
	13b	Describe any methods required to prepare the data for presentation or synthesis, such as handling of missing summary statistics, or data conversions.	Not applicable
	13c	Describe any methods used to tabulate or visually display results of individual studies and syntheses.	Methods – Quality appraisal, analysis and assessing confidence

For peer review only - http://bmjopen.bmj.com/site/about/guidelines.xhtml

	13d	Describe any methods used to synthesize results and provide a rationale for the choice(s). If meta-analysis was performed, describe the model(s), method(s) to identify the presence and extent of statistical heterogeneity, and software package(s) used.	Methods – Quality appraisal, analysis and assessing confidence
	13e	Describe any methods used to explore possible causes of heterogeneity among study results (e.g. subgroup analysis, meta-regression).	Not Applicable
	13f	Describe any sensitivity analyses conducted to assess robustness of the synthesized results.	Not Applicable
**Reporting bias assessment**	14	Describe any methods used to assess risk of bias due to missing results in a synthesis (arising from reporting biases).	Methods – Quality appraisal, analysis and assessing confidence
**Certainty assessment**	15	Describe any methods used to assess certainty (or confidence) in the body of evidence for an outcome.	Methods – Quality appraisal, analysis and assessing confidence
**RESULTS**			
**Study selection**	16a	Describe the results of the search and selection process, from the number of records identified in the search to the number of studies included in the review, ideally using a flow diagram.	Results and Figure 1. PRISMA flowchart
	16b	Cite studies that might appear to meet the inclusion criteria, but which were excluded, and explain why they were excluded.	PRISMA flowchart
**Study characteristics**	17	Cite each included study and present its characteristics.	Results and Table 1
**Risk of bias in studies**	18	Present assessments of risk of bias for each included study.	Results
**Results of individual studies**	19	For all outcomes, present, for each study: (a) summary statistics for each group (where appropriate) and (b) an effect estimate and its	Appendix 5 and 6

For peer review only

		precision (e.g. confidence/credible interval), ideally using structured tables or plots.	
**Results of syntheses**	20a	For each synthesis, briefly summarise the characteristics and risk of bias among contributing studies.	Results. Summary of qualitative findings and GRADE-CERQual Evidence Profile
	20b	Present results of all statistical syntheses conducted. If meta-analysis was done, present for each the summary estimate and its precision (e.g. confidence/credible interval) and measures of statistical heterogeneity. If comparing groups, describe the direction of the effect.	Not applicable
	20c	Present results of all investigations of possible causes of heterogeneity among study results.	Not applicable
	20d	Present results of all sensitivity analyses conducted to assess the robustness of the synthesized results.	Not applicable
**Reporting biases**	21	Present assessments of risk of bias due to missing results (arising from reporting biases) for each synthesis assessed.	Not applicable
**Certainty of evidence**	22	Present assessments of certainty (or confidence) in the body of evidence for each outcome assessed.	Results– Table 2: Summary of qualitative findings and GRADE-CERQual Evidence Profile
**DISCUSSION**			
**Discussion**	23a	Provide a general interpretation of the results in the context of other evidence.	Discussions, Interpretation
	23b	Discuss any limitations of the evidence included in the review.	Discussions, Strengths and limitations
	23c	Discuss any limitations of the review processes used.	Discussions, Strengths and limitations
	23d	Discuss implications of the results for practice, policy, and future research.	Discussions, Implications for practice and conclusions

For peer review only - http://bmjopen.bmj.com/site/about/guidelines.xhtml

OTHER INFORMATION			
**Registration and protocol**	24a	Provide registration information for the review, including register name and registration number, or state that the review was not registered.	Methods
	24b	Indicate where the review protocol can be accessed, or state that a protocol was not prepared.	Methods
	24c	Describe and explain any amendments to information provided at registration or in the protocol.	Methods
**Support**	25	Describe sources of financial or non-financial support for the review, and the role of the funders or sponsors in the review.	Sources of funding
**Competing interests**	26	Declare any competing interests of review authors.	Competing interests
**Availability of data, code and other materials**	27	Report which of the following are publicly available and where they can be found: template data collection forms; data extracted from included studies; data used for all analyses; analytic code; any other materials used in the review.	Appendix 5 and 6

For peer review only - http://bmjopen.bmj.com/site/about/guidelines.xhtml

Appendix 1.1. PRISMA Abstract Checklist

Topic	No.	Item	Reported?
**TITLE**			
**Title**	1	Identify the report as a systematic review.	Yes
**INTRODUCTION**			
**Objectives**	2	Provide an explicit statement of the main objective(s) or question(s) the review addresses.	Yes
**METHODS**			
**Eligibility criteria**	3	Specify the inclusion and exclusion criteria for the review.	Yes
**Information sources**	4	Specify the information sources (e.g. databases, registers) used to identify studies and the date when each was last searched.	Yes
**Risk of bias**	5	Specify the methods used to assess risk of bias in the included studies.	Yes
**Synthesis of results**	6	Specify the methods used to present and synthesize results.	Yes
**RESULTS**			
**Included studies**	7	Give the total number of included studies and participants and summarise relevant characteristics of studies.	Yes
**Synthesis of results**	8	Present results for main outcomes, preferably indicating the number of included studies and participants for each. If meta-analysis was done, report the summary estimate and confidence/credible interval. If comparing groups, indicate the direction of the effect (i.e. which group is favoured).	Yes
**DISCUSSION**			

For peer review only - http://bmjopen.bmj.com/site/about/guidelines.xhtml

**Limitations of evidence**	9	Provide a brief summary of the limitations of the evidence included in the review (e.g. study risk of bias, inconsistency and imprecision).	Yes
**Interpretation**	10	Provide a general interpretation of the results and important implications.	Yes
**OTHER**			
**Funding**	11	Specify the primary source of funding for the review.	Yes
**Registration**	12	Provide the register name and registration number.	Yes

For peer review only

From: Page MJ, McKenzie JE, Bossuyt PM, Boutron I, Hoffmann TC, Mulrow CD, et al. The PRISMA 2020 statement: an updated guideline for reporting systematic reviews. MetaArXiv. 2020, September 14. DOI: 10.31222/osf.io/v7gm2. For more information, visit: www.prisma-statement.org

Appendix 2. Enhancing transparency in reporting the synthesis of qualitative research: ENTREQ Checklist (Tong, et al., 2012)

Item No.	Guide and Description	Report Location
1. Aim	State the research question the synthesis addresses	Introduction
2. Synthesis methodology	Identify the synthesis methodology or theoretical framework which underpins the synthesis, and describe the rationale for choice of methodology (e.g. meta-ethnography, thematic synthesis, critical interpretive synthesis, grounded theory synthesis, realist synthesis, meta-aggregation, meta-study, framework synthesis)	Methods – _ Data extraction and assessing the methodological limitations
3. Approach to searching	Indicate whether the search was pre-planned (comprehensive search strategies to seek all available studies) or iterative (to seek all available concepts until they theoretical saturation is achieved)	Methods – Search methods for identification of studies
4. Inclusion criteria	Specify the inclusion/exclusion criteria (e.g. in terms of population, language, year limits, type of publication, study type)	Methods – Selection of studies
5. Data sources	Describe the information sources used (e.g. electronic databases (MEDLINE, EMBASE, CINAHL, psycINFO), grey literature databases (digital thesis, policy reports), relevant organisational websites, experts, information specialists, generic web searches (Google Scholar) hand searching, reference lists) and when the searches conducted; provide the rationale for using the data sources	Methods - Search methods for identification of studies
6. Electronic Search strategy	Describe the literature search (e.g. provide electronic search strategies with population terms, clinical or health topic terms, experiential or social phenomena related terms, filters for qualitative research, and search limits)	Appendix 3
7. Study screening methods	Describe the process of study screening and sifting (e.g. title, abstract and full text review, number of independent reviewers who screened studies)	Methods - Search methods for identification of studies
8. Study characteristics	Present the characteristics of the included studies (e.g. year of publication, country, population, number of participants, data collection, methodology, analysis, research questions)	Table 1 - Characteristics of included studies
9. Study selection results	Identify the number of studies screened and provide reasons for study exclusion (e.g. for comprehensive searching, provide numbers of studies screened and reasons for exclusion indicated in a figure/flowchart; for iterative searching describe reasons for study exclusion	Fig 1 - PRISMA flow diagram

For peer review only

	and inclusion based on modifications to the research question and/or contribution to theory development)	
10. Rationale for appraisal	Describe the rationale and approach used to appraise the included studies or selected findings (e.g. assessment of conduct (validity and robustness), assessment of reporting (transparency), assessment of content and utility of the findings)	Methods - Quality appraisal, analysis and assessing confidence and Appendix 4
11. Appraisal items	State the tools, frameworks and criteria used to appraise the studies or selected findings (e.g. Existing tools: CASP, QARI, COREQ, Mays and Pope [25]; reviewer developed tools; describe the domains assessed: research team, study design, data analysis and interpretations, reporting)	Methods - Data extraction and assessing the methodological limitations
12. Appraisal process	Indicate whether the appraisal was conducted independently by more than one reviewer and if consensus was required	Methods - Data extraction and assessing the methodological limitations
13. Appraisal results	Present results of the quality assessment and indicate which articles, if any, were weighted/excluded based on the assessment and give the rationale	Appendix 5
14. Data extraction	Indicate which sections of the primary studies were analysed and how were the data extracted from the primary studies? (e.g. all text under the headings "results /conclusions" were extracted electronically and entered into a computer software)	Methods- Data extraction and assessing the methodological limitations
15. Software	State the computer software used, if any	None
16. Number of reviewers	Identify who was involved in coding and analysis	Methods - Data extraction and assessing the methodological limitations
17. Coding	Describe the process for coding of data (e.g. line by line coding to search for concepts)	Methods - Quality appraisal, analysis and assessing confidence
18. Study comparison	Describe how were comparisons made within and across studies (e.g. subsequent studies were coded into pre-existing concepts, and new concepts were created when deemed necessary)	Methods - Quality appraisal, analysis and assessing confidence

For peer review only

19. Derivation of themes	Explain whether the process of deriving the themes or constructs was inductive or deductive	Methods - Quality appraisal, analysis and assessing confidence
20. Quotations	Provide quotations from the primary studies to illustrate themes/constructs, and identify whether the quotations were participant quotations of the author's interpretation	Results
21. Synthesis output	Present rich, compelling and useful results that go beyond a summary of the primary studies (e.g. new interpretation, models of evidence, conceptual models, analytical framework, development of a new theory or construct)	Discussion

For peer review only

Appendix 3. Search Strategy

Embase (inception to 2021 March 22)

1 exp Calcium/ or Calcium Carbonate/ (305629)
2 (calcium adj3 supplement*).mp. (9456)
3 1 or 2 (307747)
4 Pregnant Women/ or Prenatal Care/ (109074)
5 (pregnan* or prenatal).mp. (1086333)
6 4 or 5 (1086333)
7 3 and 6 (8440)
8 limit 7 to humans (5885)
9 limit 8 to (amphibia or ape or bird or cat or cattle or chicken or dog or "ducks and geese" or fish or "frogs and toads" or goat or guinea pig or "hamsters and gerbils" or horse or monkey or mouse or "pigeons and doves" or "rabbits and hares" or rat or reptile or sheep or swine) (277)
10 8 not 9 (5608)
11 limit 10 to (conference abstract or conference paper or "conference review" or editorial or erratum or letter or note or "review") (2567)
12 10 not 11 (**3041**)

Embase (inception to 2022 August 16) – Search update

1 exp Calcium/ or Calcium Carbonate/ (323807)
2 (calcium adj3 supplement*).mp. (10017)
3 1 or 2 (326035)
4 Pregnant Women/ or Prenatal Care/ (125455)
5 (pregnan* or prenatal).mp. (1161704)
6 4 or 5 (1161704)
7 3 and 6 (9061)
8 limit 7 to humans (6413)
9 limit 8 to (amphibia or ape or bird or cat or cattle or chicken or dog or "ducks and geese" or fish or "frogs and toads" or goat or guinea pig or "hamsters and gerbils" or horse or monkey or mouse or "pigeons and doves" or "rabbits and hares" or rat or reptile or sheep or swine) (303)
10 8 not 9 (6110)
11 limit 10 to (conference abstract or conference paper or "conference review" or editorial or erratum or letter or note or "review") (2744)
12 10 not 11 (3366)
13 limit 12 to yr="2022 - 2023" (**142**)

MEDLINE (1946 to March Week 2 2021)

1 exp Calcium/ or Calcium Carbonate/ (277850)
2 (calcium adj3 supplement*).mp. (5560)

For peer review only

3 1 or 2 (280742)
4 Pregnant Women/ or Prenatal Care/ (36709)
5 (pregnan* or prenatal).mp. (999134)
6 4 or 5 (999134)
7 3 and 6 (6417)
8 limit 7 to humans (3417)
9 limit 8 to animals (618)
10 8 not 9 (2799)
11 limit 10 to ("review articles" and case reports) (17)
12 10 not 11 (**2782 results**)

MEDLINE (inception to August Week 1, 2022) – Search update

1 exp Calcium/ or Calcium Carbonate/ (286238)
2 (calcium adj3 supplement*).mp. (5967)
3 1 or 2 (289314)
4 Pregnant Women/ or Prenatal Care/ (42373)
5 (pregnan* or prenatal).mp. (1068999)
6 4 or 5 (1068999)
7 3 and 6 6605
8 limit 7 to humans (3545)
9 limit 8 to animals (634)
10 8 not 9 (2911)
11 limit 10 to ("review articles" and case reports) (18)
12 10 not 11 (2893)
13 limit 12 to yr="2021 - 2022" (**89**)

CINAHL

CINAHL (inception to March 2021)

(calcium and supplement*) AND (pregnan* or prenatal) AND (preeclamp* or pre-eclamp* or eclamp* or "gestational hypertension" or "maternal hypertension") (**132**)

CINAHL (March 2021 to August 2022) – Search update

(calcium and supplement*) AND (pregnan* or prenatal) AND (preeclamp* or pre-eclamp* or eclamp* or "gestational hypertension" or "maternal hypertension") (**1**)

GLOBAL HEALTH

GLOBAL HEALTH (inception to March 2021)

(calcium and supplement*) AND (pregnan* or prenatal) AND (preeclamp* or pre-eclamp* or eclamp* or "gestational hypertension" or "maternal hypertension") (**158 results**)

GLOBAL HEALTH (2021 to August 2022) – Search update

(calcium and supplement*) AND (pregnan* or prenatal) AND (preeclamp* or pre-eclamp* or eclamp* or "gestational hypertension" or "maternal hypertension") (**158 results**)

For peer review only - http://bmjopen.bmj.com/site/about/guidelines.xhtml

For peer review only

For peer review only - http://bmjopen.bmj.com/site/about/guidelines.xhtml

Appendix 4. Critical Appraisal Table

Qualitative studies

STUDY DETAIL	SCREENING QUESTIONS		1. QUALITATIVE STUDIES							MMAT RATING
**First author**	**S1. Are there clear research questions?**	**S2. Do the collected data allow to address the research questions?**	**1.1. Is the qualitative approach appropriate to answer the research question? (Aim, appropriateness of a qualitative approach)**	**1.2. Are the qualitative data collection methods adequate to address the research question? (recruitment, data collection)**	**1.3. Are the findings adequately derived from the data? (rigor in analysis)**	**1.4. Is the interpretation of results sufficiently substantiated by data? (link from data to findings)**	**1.5. Is there coherence between qualitative data sources, collection, analysis and interpretation? (overall design from start to finish)**	**1.6. Have ethical issues been taken into consideration? (consent, confidentiality, ethics approval)**	**1.7. Is relationship between researcher and participants adequately considered? (interaction and reflection on how research team influences design & implementation)**	
**Vestering 2019**	Yes	Yes	Yes	Yes	Yes	Yes	Yes	Partial	Partial	"Moderate" (minor issues impacting credibility/validity)
**Birhanu 2018**	Yes	Yes	Yes	Yes	Yes	Yes	Yes	Yes	Unclear	"Moderate" (minor issues impacting credibility/validity)
**Martin 2017a**	Yes	Yes	Yes	Yes	Yes	Yes	Yes	Yes	Unclear	"Moderate" (minor issues impacting credibility/validity)
**Martin 2018**	Yes	Yes	Yes	Yes	Yes	Yes	Yes	Yes	Partial	"Moderate" (minor issues impacting credibility/validity)

Quantitative studies

STUDY DETAIL	SCREENING QUESTIONS		3. NON-RANDOMIZED STUDIES					4. QUANTITATIVE DESCRIPTIVE STUDIES					
**First author**	**S1. Are there clear research questions?**	**S2. Do the collected data allow to address the research questions?**	**3.1. Are the participants representative of the target population?**	**3.2. Are measurements appropriate regarding both the outcome and intervention (or exposure)?**	**3.3. Are there complete outcome data?**	**3.4. Are the confounders accounted for in the design and analysis?**	**3.5. During the study period, is the intervention administered (or exposure occurred) as intended?**	**4.1. Is the sampling strategy relevant to address the research question?**	**4.2. Is the sample representative of the target population?**	**4.3. Are the measurements appropriate?**	**4.4. Is the risk of nonresponse bias low?**	**4.5. Is the statistical analysis appropriate to answer the research question?**	**MMAT RATING**
Baxter 2014	Yes	Yes						Yes	Unclear	Yes	No	Yes	"Low" (some issues likely to impact credibility/validity)
Thapa 2016	Yes	Yes	Yes	Yes	Yes	Yes	Yes						"High" (no or very minor significant issues)
Nguyen 2019	Yes	Yes						Yes	Yes	Yes	Unclear	Yes	"Moderate" (minor issues impacting credibility/validity)
Omotayo 2018a & Martin 2017b	Yes	Yes	Yes	Yes	Partial	Yes	Yes						"Moderate" (minor issues impacting credibility/validity)
Nguyen 2017	Yes	Yes						Yes	Yes	Yes	Yes	Yes	"High" (no or very minor significant issues)
Nguyen 2018	Yes	Yes						Yes	Yes	Yes	Yes	Yes	"High" (no or very minor significant issues)

Liu 2019	Yes	Yes						Yes	Yes	Partial	No	Yes	"Low" (some issues likely to impact credibility/validity)
Shakya Shrestha 2020	Yes	Yes						Partial	Unclear	Partial	Yes	Partial	"Very low" (significant issues impacting credibility/validity)
Ghosh-Jerath 2015	Yes	Yes						Yes	Yes	Partial	No	Yes	"Low" (some issues likely to impact credibility/validity)

*Grey shades or empty cells refer to not applicable.

Mixed methods studies

STUDY DETAIL	First author	Omotayo 2018b & Martin 2017c	Kachwaha 2022
SCREENING QUESTIONS	S1. Are there clear research questions?	Yes	Yes
	S2. Do the collected data allow to address the research questions?	Yes	Yes
1. QUALITATIVE STUDIES	1.1. Is the qualitative approach appropriate to answer the research question? (Aim, appropriateness of a qualitative approach)	Yes	Yes
	1.2. Are the qualitative data collection methods adequate to address the research question? (recruitment, data collection)	Yes	Yes
	1.3. Are the findings adequately derived from the data? (rigor in analysis)	Yes	Yes
	1.4. Is the interpretation of results sufficiently substantiated by data? (link from data to findings)	Yes	Yes
	1.5. Is there coherence between qualitative data sources, collection, analysis and interpretation? (overall design from start to finish)	Yes	Yes
	1.6. Have ethical issues been taken into consideration? (consent, confidentiality, ethics approval)	Yes	Yes
	1.7. Is relationship between researcher and participants adequately considered? (interaction and reflection on how research team influences design & implementation)	Unclear	Unclear
	4.1. Is the sampling strategy relevant to address the research question?	Yes	Yes

4. QUANTITATIVE DESCRIPTIVE STUDIES	**4.2. Is the sample representative of the target population?**	Yes	Yes
	**4.3. Are the measurements appropriate?**	Yes	Yes
	**4.4. Is the risk of nonresponse bias low?**	Yes	Partial
	**4.5. Is the statistical analysis appropriate to answer the research question?**	Yes	Yes
5. MIXED METHODS STUDIES	**5.1. Is there an adequate rationale for using a mixed methods design to address the research question?**	Yes	Yes
	**5.2. Are the different components of the study effectively integrated to answer the research question?**	Yes	Yes
	**5.3. Are the outputs of the integration of qualitative and quantitative components adequately interpreted?**	Yes	Yes
	**5.4. Are divergences and inconsistencies between quantitative and qualitative results adequately addressed?**	Yes	Yes
	**5.5. Do the different components of the study adhere to the quality criteria of each tradition of the methods involved?**	Yes	Partial
**MMAT RATING**		"High" (no or very minor significant issues)	"Moderate" (minor issues impacting credibility/validity)

For peer review only

Appendix 5. Evidence profile

No	Summary of qualitative review findings	Contributing qualitative studies	Methodological limitations assessment	Coherence assessment	Relevance assessment	Adequacy assessment	Overall CERQual assessment	Explanation of overall assessment
**Women's factors**								
**1. Women's knowledge and learning**								
**1.1**	**Women's knowledge about pre-eclampsia or eclampsia** Most women had limited knowledge about pre-eclampsia or eclampsia, and these conditions were not typically considered by women to be a serious problem. Symptoms of pre-eclampsia such as swollen feet, severe headache, blurred vision and vomiting were considered normal signs and symptoms in pregnancy, while seizures were associated with evil attacks or nutritional deficiencies.	(Martin 2017b, Birhanu 2018, Vestering 2019)	**Minor concerns:** Three studies with minor issues (reflexivity and ethics).	**No or very minor concerns**	**Moderate concerns:** One study is indirectly relevant to review aim and represented 3 countries (Kenya, Ethiopia, Netherlands), including 1 low-income, 1 lower middle income and 1 high income country. All perspectives came from health providers and women.	**Minor concerns:** 3 out of 6 studies contributed to review finding (2 thick data, 1 thin data).	**High confidence**	Due to no or very minor concerns on coherence, minor concerns on methodological limitations (reflexivity and ethics), moderate concerns on relevance (1 out of 3 studies are indirectly relevant to our review aim and small number of countries), and minor concerns of adequacy (3 out of 6 contributed with 2 thick and 1 thin data).
**1.2**	**Information provision to women** Women felt they did not receive adequate information during pregnancy from healthcare providers about pre-eclampsia or eclampsia and calcium supplements. They described wanting to be given more information, regardless of their pre-eclampsia risk status. Women believed that having this essential information could help them to make	(Vestering 2019, Martin 2018)	**Minor concerns:** Two studies with minor issues (ethics and reflexivity).	**Moderate concerns:** No good understanding why some providers worry in generating anxiety to women	**Moderate concerns:** All studies are directly relevant to review aim and represented 2 countries (Kenya, Netherlands), including 1 lower middle income and 1 high income country. All perspectives came from health providers and women.	**Serious concerns:** 2 out of 6 studies contributed to review finding (1 thick data, 1 thin data).	**Low confidence**	Minor concerns on methodological limitations (ethics and reflexivity), moderate concerns on coherence (no clear understanding on why some providers worry in generating anxiety to women while the others are not), moderate concerns on relevance (small number of countries), and

No	Summary of qualitative review findings	Contributing qualitative studies	Methodological limitations assessment	Coherence assessment	Relevance assessment	Adequacy assessment	Overall CERQual assessment	Explanation of overall assessment
	informed choices and actively participate in their care. There were, however, mixed opinions from healthcare providers - some feared that more information could generate anxiety for women, while others were more supportive of providing information.			while others are not.				serious concerns on adequacy (2 out of 6 contributed with 1 thick and 1 thin data).
1.3	**Learning about calcium supplements** Women typically learned about dietary calcium, including pre-eclampsia and eclampsia symptoms, from healthcare providers. They considered healthcare providers to be the most trusted and reliable source of information and reported feeling confident about taking calcium-containing supplements after receiving adequate information from them. Women also appreciated receiving information on calcium supplements and pre-eclampsia or eclampsia via information, education, and communication (IEC) materials like videos, media, and trusted websites.	(Vestering 2019, Martin 2018, Birhanu 2018)	**Minor concerns:** Three studies with minor issues (reflexivity and ethics).	**No or very minor concerns**	**Minor concerns:** All studies are directly relevant to review aim and represented 3 countries (Kenya, Ethiopia, Netherlands), including 1 low-income, 1 lower middle income and 1 high income country. All perspectives came from health providers and women.	**Minor concerns:** 3 out of 6 studies contributed to findings (2 with moderate-thick data and 1 with thin data.	**High confidence**	Due to no or very minor concerns on coherence, minor concerns on relevance (small number of countries), minor concerns on methodological limitations (reflexivity and ethics), and minor concerns on adequacy (3 out of 6 contributed, 1 moderate thick and 2 thin data).

For peer review only

No	Summary of qualitative review findings	Contributing qualitative studies	Methodological limitations assessment	Coherence assessment	Relevance assessment	Adequacy assessment	Overall CERQual assessment	Explanation of overall assessment
**2. Women's beliefs about calcium supplements in pregnancy**								
**2.1**	**Fears about side-effects as a barrier to calcium supplements uptake** Women's fears about the side effects of calcium supplements affected their adherence. Women highlighted that assurance of safe use of calcium supplement is a key facilitator to consistent use. However, some women felt safety was not assured by healthcare providers, especially when calcium supplements were perceived as "experimental". Women had also received messages from their families or communities that any pills consumed during pregnancy could be harmful.	(Omotayo 2017, Birhanu 2018, Vestering 2019, Martin 2018)	**Minor concerns:** Three out of four studies have minor issues (ethics and reflexivity) and one study with no or very minor issues.	**No or very minor concerns**	**Minor concerns:** All studies are directly relevant to review aim and represented 3 countries (Kenya, Ethiopia, Netherlands), including 1 low-income, 1 lower middle income and 1 high income country. All perspectives came from women.	**Minor concerns** Overall, small number of studies contributing to the qualitative evidence synthesis.	**High Confidence**	Due to no or very minor concerns on coherence, minor concerns on relevance (small number of countries), minor concerns on adequacy (small number of studies contributing to the qualitative evidence synthesis), and minor concerns on methodological limitations (reflexivity and ethics).
**2.2**	**Women's experiences of side-effects** Some women reported experiencing side effects after taking calcium and iron-folic acid supplements, such as dizziness, vomiting, nausea, stomach aches, loss of appetite, tiredness, diarrhoea, bloating, and burping, yet noted that side effects subsided with time. Women also reported that they continued consuming calcium despite	(Omotayo 2017, Martin 2018, Birhanu 2018)	**Minor concerns:** Two out of three studies have minor issues (ethics and reflexivity) and one study with no or very minor issues	**No or very minor concerns**	**Moderate concerns:** All studies are directly relevant to review aim and represented 2 countries (Kenya, Ethiopia), including 1 low-income and 1 lower middle-income country. All perspectives came from health providers and women.	**Minor concerns:** 3 out of 6 studies contributed to the findings (2 thin and 1 with thick data).	**High Confidence**	Due to no or very minor concerns on coherence, minor concerns on methodological limitations (ethics and reflexivity), moderate concerns on relevance (only low and lower middle-income country and small number of countries) and minor concerns on adequacy (3 out of 6 studies

No	Summary of qualitative review findings	Contributing qualitative studies	Methodological limitations assessment	Coherence assessment	Relevance assessment	Adequacy assessment	Overall CERQual assessment	Explanation of overall assessment
	these side effects.							contributed with 2 thick and 1 with thin data).
2.3	**Concerns about being stigmatized as HIV patients** Women expressed concerns that if they ingested calcium supplements, they could be stigmatized as HIV patients, which was a reported barrier to use. Some women were afraid of being stigmatized as their community often associated nutritional supplement consumption and accompanying reminder posters with HIV.	(Martin 2018, Vestering 2019)	**No or very minor concerns**	**No or very minor concerns**	**Moderate concerns:** All studies are directly relevant to review aim and represented 2 countries (Kenya, Netherlands), including 1 lower middle income and 1 high income country. All perspectives came from health providers and women.	**Serious concerns:** 2 out of 6 studies contributed to review finding (1 thick data and 1 thin data).	**Moderate Confidence**	Due to no or very minor concerns on methodological limitations, no or very minor concerns on coherence, minor concerns on relevance (small number of countries), and serious concerns on adequacy (2 out of 6 study contributed).
2.4	**Positive perceptions of calcium supplements** Women reported that both their perceptions about expected benefits and previous experiences of taking calcium and iron-folic acid supplements were facilitators of use. Women believed that consuming pills could compensate for sub-optimal nutrition during pregnancy, and that supplements during pregnancy would help keep their baby safe.	(Vestering 2019, Martin 2018, Martin 2017b, Birhanu 2018, Omotayo 2017)	**Minor concerns:** Four out of five studies have minor issues (ethics and reflexivity) and one study with no or very minor issues.	**No or very minor concerns**	**Minor concerns:** One study is indirectly relevant to review aim and represented 3 countries (Kenya, Ethiopia, Netherlands), including 1 low-income, 1 lower middle income and 1 high income country. All perspectives came from health providers and women.	**Minor concerns** Overall, small number of studies contributing to the qualitative evidence synthesis.	**High Confidence**	Due to no or very minor concerns on coherence, minor concerns on methodological limitations (ethics and reflexivity), minor concerns on relevance (1 study indirectly relevant to review aim and small number of countries) and adequacy (small number of studies contributing to the qualitative evidence synthesis).
**3. Calcium supplement characteristics and regimens**								

For peer review only

No	Summary of qualitative review findings	Contributing qualitative studies	Methodological limitations assessment	Coherence assessment	Relevance assessment	Adequacy assessment	Overall CERQual assessment	Explanation of overall assessment
3.1	**Varying preferences about characteristics of calcium tablets** Positive perceptions about the characteristics of the calcium tablet played a role in motivating women to take it. Some women preferred the chewable, sweet-tasting tablets that could be swallowed without water, while others preferred the hard tablets which were smaller in size, had no smell, and needed to be taken with water. Based on individual preference, the taste, smell, size, and convenience affected calcium supplement use.	(Omotayo 2017, Birhanu 2018)	**Minor concerns:** One study has minor issues (reflexivity) and one study has no or very minor issues.	**No or very minor concerns**	**Moderate concerns:** All studies are directly relevant to review aim and represented 2 countries (Kenya, Ethiopia), including 1 low-income and 1 lower middle-income country. All perspectives came from women.	**Moderate concerns:** 2 out of 6 studies contributed to findings and both had moderate to thick data.	**Moderate confidence**	Due to no or very minor concerns on coherence, minor concerns on methodological limitations (reflexivity), moderate concerns on relevance (all low or lower middle-income countries and small number of countries) and moderate concerns on adequacy (2 out of 6 studies contributed with moderate to thick data).
3.2	**Supplement regimen as a barrier to use** Women described that they feel overwhelmed with the number of calcium tablets they had to take each day, especially women with other comorbidities who needed to take additional medications for their health conditions. Women felt that 3-4 pills per day at multiple times was overly onerous and preferred if they were combined into one pill.	(Martin 2018, Birhanu 2018, Omotayo 2017)	**Minor concerns:** Two studies have minor issues (reflexivity) and one study has no or very minor issues.	**No or very minor concerns**	**Moderate concerns:** All studies are directly relevant to review aim and represented 2 countries (Kenya, Ethiopia), including 1 low-income and 1 lower middle-income country. All perspectives came from health providers and women.	**Minor concerns:** 3 out of 6 studies contributing to findings and all have moderate to thick data.	**High Confidence**	No or very minor concerns on coherence, minor concerns on methodological limitations (reflexivity), minor concerns on adequacy (3 out of 6 studies contributed with moderate to thick data), and moderate concerns on relevance (small number of countries).
**4. Daily routines and food insecurity**								

For peer review only

No	Summary of qualitative review findings	Contributing qualitative studies	Methodological limitations assessment	Coherence assessment	Relevance assessment	Adequacy assessment	Overall CERQual assessment	Explanation of overall assessment
**4.1**	**Adherence challenges due to routines** Adherence to calcium supplements consumption was challenging for some women because of conflicting activities in their daily routine, such as consuming other medications, traveling, being away from home, and household chores, which can lead them to forget to take calcium.	(Martin 2018, Birhanu 2018, Omotayo 2017)	**Minor concerns:** Two out of three studies have minor issues (reflexivity) and one study with no or very minor issues.	**No or very minor concerns**	**Moderate concerns:** All studies are directly relevant to review aim and represented 2 countries (Kenya, Ethiopia), including 1 low-income and 1 lower middle-income country. All perspectives came from health providers and women.	**Minor concerns:** Overall, small number of studies contributing to qualitative evidence synthesis.	**High Confidence**	Due to no or very minor concerns with coherence, minor concerns on methodological limitations (reflexivity), moderate concerns on relevance (small number of countries) and moderate concerns on adequacy (small number of studies contributing to qualitative evidence synthesis).
**4.2**	**Food insecurity as a barrier to calcium uptake** Women believed that adequate food was necessary to be able to take the supplements, to avoid nausea. They perceived it as normal to eat before consuming any medication. However, women reported that food insecurity was a critical barrier to calcium uptake.	(Birhanu 2018, Martin 2018)	**Minor concerns:** Two studies with minor issues (reflexivity).	**No or very minor concerns**	**Moderate concerns:** All studies are directly relevant to review aim and represented 2 countries (Kenya, Ethiopia), including 1 low-income and 1 lower middle-income country. All perspectives came from health providers and women.	**Moderate concerns:** 2 out of 6 studies contributed to findings with 1 thick and 1 thin data.	**Moderate Confidence**	Due to no or very minor concern with coherence, minor concerns on methodological limitations (reflexivity), moderate concerns on adequacy (2 out 6 studies contributed with 1 thick and 1 thin data), and moderate concerns on relevance (small number of countries).
**5. Strategies to improve the use of calcium supplements**								
**5.1**	**Implementation of reminders to promote adherence** Women and healthcare providers perceived reminders as beneficial in promoting women's adherence to consuming calcium supplements.	(Martin 2017a, Martin 2018, Birhanu 2018,	**No or very minor concerns**	**No or very minor concerns**	**Moderate concerns:** One study indirectly relevant to review aim and represented 2 countries (Kenya, Ethiopia), including 1	**Minor concerns:** Overall, small numbers of studies	**High Confidence**	Due to no or very minor concerns on methodological limitations, no or very minor concerns on coherence, minor concerns on adequacy (small number of studies contributing

No	Summary of qualitative review findings	Contributing qualitative studies	Methodological limitations assessment	Coherence assessment	Relevance assessment	Adequacy assessment	Overall CERQual assessment	Explanation of overall assessment
	Several reminder strategies were described as useful by women and healthcare providers, such as home-based posters, calendars with illustrations and daily reminders, and integrating supplement consumption into women's daily routine, such as mealtimes.	Omotayo 2017)			low-income and 1 lower middle-income country. All perspectives came from health providers and women.	contributing to qualitative evidence synthesis.		to qualitative evidence synthesis), moderate concerns on relevance (due to all studies coming from low income or lower middle-income country and small number of countries).
5.2	**Importance of family support and 'adherence partner' implementation** Having family support was instrumental to pregnant women adhering to calcium supplement use. This could be leveraged by notifying family members on the importance of calcium and appointing someone to be an "adherence partner" or "pill buddy" to help remind her to take it. Both women and healthcare providers were positive about adherence partners in providing support in terms of encouraging them to take the supplements, providing food, helping them around the house, providing emotional support, improving family relationships, and	(Martin 2018, Martin 2017b, Birhanu 2018, Martin 2017a, Omotayo 2017)	**No or very minor concerns**	**No or very minor concerns**	**Moderate concerns:** Two out of five studies indirectly relevant to review aim and represented 2 countries (Kenya, Ethiopia), including 1 low-income and 1 lower middle-income country. All perspectives came from health providers, partner and women.	**Minor concerns:** Overall, small number of studies contributing to qualitative evidence synthesis.	**High Confidence**	No or very minor concerns on methodological limitations, no or very minor concerns on coherence, minor concerns on adequacy (small number of studies contributing to qualitative evidence synthesis), and moderate concerns on relevance (due to all studies coming from low income or lower middle-income country only, 2 of 5 studies has indirectly relevant aim, and small number of countries).

For peer review only

No	Summary of qualitative review findings	Contributing qualitative studies	Methodological limitations assessment	Coherence assessment	Relevance assessment	Adequacy assessment	Overall CERQual assessment	Explanation of overall assessment
	thereby increasing partner or husband involvement in pregnancy.							
5.3	**Counselling facilitates calcium supplements uptake** Both women and healthcare providers acknowledged that counselling women on the benefits of calcium was a motivator to calcium supplements uptake. Women valued the discussion they have with healthcare providers and felt more confident to take calcium supplements when they received counselling and information and pre-eclampsia or eclampsia and the benefits of calcium from their healthcare providers.	(Birhanu 2018, Martin 2018)	**No or very minor concerns**	**No or very minor concerns**	**Moderate concerns:** All studies are directly relevant to review aim and represented 2 countries (Kenya, Ethiopia), including 1 low-income and 1 lower middle-income country. All perspectives came from health providers and women.	**Moderate concerns:** 2 out of 6 studies contributing to findings with all thick data.	**Moderate Confidence**	No or very minor concerns on methodological limitations, no or very minor concerns on coherence, moderate concerns on relevance (due to all studies coming from low or lower middle-income country and small number of countries) and moderate concerns on adequacy (2 out 6 studies contributed with thick data).
**Health care providers' factors**								
**6. Healthcare provider knowledge and training**								
6.1	**Varied knowledge about pre-eclampsia or eclampsia among healthcare providers** Healthcare providers' knowledge about pre-eclampsia and eclampsia was varied. Some felt that pre-eclampsia and eclampsia is not a priority health concern in their area	(Birhanu 2018, Martin 2017a, Kachwaha 2022)	**No or very minor concerns**	**No or very minor concerns**	**Moderate concerns:** One study is indirectly relevant to review aim and represented 2 countries (Kenya, Ethiopia), including 1 low-income and 1 lower middle-income country.	**Moderate concerns:** 2 out of 6 studies contributed to findings with thick data.	**Moderate Confidence**	Due to no or very minor concerns on methodological limitations and coherence, moderate concerns on relevance (due to all studies coming from low or lower middle-income countries and small number of studies) and

For peer review only

No	Summary of qualitative review findings	Contributing qualitative studies	Methodological limitations assessment	Coherence assessment	Relevance assessment	Adequacy assessment	Overall CERQual assessment	Explanation of overall assessment
	and reported never having encountered any case.				All perspectives came from health providers.			moderate concerns on adequacy (2 out 6 studies contributed with thick data).
6.2	**Inadequate training to diagnose and treat pre-eclampsia and eclampsia** While some healthcare providers mentioned that training about pre-eclampsia or eclampsia and calcium supplements was adequate, others reported that their training lacked depth and continuity, and thus felt unprepared to diagnose it and offer information these conditions and the use of calcium for prevention. Healthcare providers expressed the need to have more and continuous training on managing pre-eclampsia, as well as time to address concerns or resistance from the community.	(Martin 2018, Birhanu 2018, Martin 2017a)	**No or very minor concerns**	**No or very minor concerns**	**Moderate concerns:** One study is indirectly relevant to review aim and represented 2 countries (Kenya, Ethiopia), including 1 low-income and 1 lower middle-income country. All perspectives came from health providers.	**Minor concerns:** 3 out of 6 studies contributed with 2 thick and 1 thin data.	**High Confidence**	No or very minor concerns on methodological limitations, coherence, moderate concerns on relevance (due to all studies coming from low income or lower middle-income country and small number of countries) and minor concerns on adequacy (3 out of 6 studies contributed with 2 thick and 1 thin data).
**7. Beliefs about the intervention**								
7.1	**Perceived overmedicalization when prescribing calcium supplements** Both healthcare providers and women perceived that prescribing more tablets to “low-risk” women during pregnancy was a form of over-	(Vestering 2019)	**Minor concerns:** One study with minor issues (reflexivity and ethics).	**No or very minor concerns**	**Serious concerns:** One study is directly relevant to review aim and represented 1 country (Netherlands), which is high income country. All perspectives	**Serious concerns:** 1 out of 6 study contributed to findings	**Low confidence**	No or very minor concerns on coherence, minor concerns on methodological limitations (reflexivity and ethics), serious concerns on relevance (evidence coming from high income country only), serious

For peer review only

No	Summary of qualitative review findings	Contributing qualitative studies	Methodological limitations assessment	Coherence assessment	Relevance assessment	Adequacy assessment	Overall CERQual assessment	Explanation of overall assessment
	medicalization of pregnancy. However, some healthcare providers felt that calcium supplements were a way to prevent further medicalization due to pre-eclampsia-related complications.				came from health providers and women.	with thick data.		concerns on adequacy (1 out of 6 study contributed).
7.2	**Beliefs about calcium supplements** Healthcare providers generally had positive beliefs about calcium supplements and there was optimism that calcium could be delivered through antenatal care healthcare providers. Some facilitators motivating healthcare providers to prescribe calcium supplements included their beliefs in its prevention value and expected benefits, that women liked the calcium supplements and experienced benefits from it, and a perceived lack of knowledge on how to treat pre-eclampsia which motivated healthcare providers to side towards prevention.	(Martin 2018, Vestering 2019, Birhanu 2018)	**Minor concerns:** Two studies with minor issues (ethics and reflexivity).	**No or very minor concerns**	**Minor concerns:** All studies are directly relevant to review aim and represented 3 countries (Kenya, Ethiopia, Netherlands), including 1 low-income, 1 lower middle income and 1 high income country. All perspectives came from health providers.	**Minor concerns:** 3 out of 6 studies contributed with 2 thick and 1 thin data.	**High Confidence**	No or very minor concerns on coherence, minor concerns on relevance (small number of countries), minor concerns on methodological limitations (ethics and reflexivity), and minor concerns on adequacy (3 out of 6 studies contributed with 2 thick and 1 thin data).
**8. Structural factors**								

For peer review only

No	Summary of qualitative review findings	Contributing qualitative studies	Methodological limitations assessment	Coherence assessment	Relevance assessment	Adequacy assessment	Overall CERQual assessment	Explanation of overall assessment
**8.1**	**High workload, inadequate staffing, stock out, and lack of equipment** In general, healthcare providers felt that their workload increased by including calcium supplements in antenatal care for pregnant women. Healthcare providers reported existing inadequate staffing, yet they needed to provide additional counselling and prescription to women, especially pregnant women with comorbidities.	(Martin 2018, Birhanu 2018)	**No or very minor concerns**	**No or very minor concerns**	**Moderate concerns:** All studies are directly relevant to review aim and represented 2 countries (Kenya, Ethiopia), including 1 low-income and 1 lower middle-income country. All perspectives came from health providers.	**Serious concerns:** 2 out 6 studies contributed to findings (1 thick, 1 thin data).	**Moderate Confidence**	No or very minor concerns on methodological limitations, no or very minor concerns on coherence, minor concerns on relevance (due to all studies coming from low income or lower middle-income countries and small number of countries) and serious concerns on adequacy (2 out 6 studies contributed).

References

Birhanu Z, Chapleau GM, Ortolano SE, Mamo G, Martin SL, Dickin KL. Ethiopian women's perspectives on antenatal care and iron-folic acid supplementation: Insights for translating global antenatal calcium guidelines into practice. Matern Child Nutr [Internet]. 2018 Feb;14:e12424. Available from: https://onlinelibrary.wiley.com/doi/10.1111/mcn.12424

Kachwaha S, Nguyen PH, Tran LM, Avula R, Young MF, Ghosh S, et al. Specificity Matters: Unpacking Impact Pathways of Individual Interventions within Bundled Packages Helps Interpret the Limited Impacts of a Maternal Nutrition Intervention in India. J Nutr. 2022 Feb;152(2):612–29.

Martin SL, Seim GL, Wawire S, Chapleau GM, Young SL, Dickin KL. Translating formative research findings into a behaviour change strategy to promote antenatal calcium and iron and folic acid supplementation in western Kenya. Matern Child Nutr [Internet]. 2017 Jan 22;13(1):mcn.12233. Available from: https://onlinelibrary.wiley.com/doi/10.1111/mcn.12233 Martin 2017a 38

Martin SL, Omotayo MO, Chapleau GM, Stoltzfus RJ, Birhanu Z, Ortolano SE, et al. Adherence partners are an acceptable behaviour change strategy to support calcium and iron-folic acid supplementation among pregnant women in Ethiopia and Kenya. Matern Child Nutr [Internet]. 2017 Jul;13(3):e12331. Available from: https://onlinelibrary.wiley.com/doi/10.1111/mcn.12331 Martin 2017b 41

Martin SL, Wawire V, Ombunda H, Li T, Sklar K, Tzehaie H, et al. Integrating Calcium Supplementation into Facility-Based Antenatal Care Services in Western Kenya: A Qualitative Process Evaluation to Identify Implementation Barriers and Facilitators. Curr Dev Nutr [Internet]. 2018 Nov 1;2(11). Available from: https://academic.oup.com/cdn/article/doi/10.1093/cdn/nzy068/5078341 39

Omotayo MO, Martin SL, Stoltzfus RJ, Ortolano SE, Mwanga E, Dickin KL. With adaptation, the WHO guidelines on calcium supplementation for prevention of pre-eclampsia are adopted by pregnant women. Matern Child Nutr [Internet]. 2018 Apr;14(2):e12521. Available from: http://doi.wiley.com/10.1111/mcn.12521

Vestering A, Bekker MN, Grobbee DE, van der Graaf R, Franx A, Crombag NMT, et al. Views and preferences of medical professionals and pregnant women about a novel primary prevention intervention for hypertensive disorders of pregnancy: a qualitative study. Reprod Health [Internet]. 2019 May 2;16(1):46. Available from: http://www.ncbi.nlm.nih.gov/pubmed/31046778

For peer review only

Appendix 6. Summary of Quantitative Findings

#	Summary of Quantitative review findings	Contributing quantitative studies	Quality ratings
**Women's factors**			
**1**	**Knowledge and learning**		
1.1	**Women's knowledge about pre-eclampsia or eclampsia**<br>No data supporting this theme from quantitative evidence.	No data supporting this theme from quantitative evidence.	No data supporting this theme from quantitative evidence.
1.2	**Information provision to women**<br>No data supporting this theme from quantitative evidence.	No data supporting this theme from quantitative evidence.	No data supporting this theme from quantitative evidence.
1.3	**Learning about calcium supplementation**<br>Quantitative evidence supported qualitative findings regarding women's learning on calcium supplements. In the context of an intervention implementing training to healthcare providers to reinforce calcium-related messages to women, women reported that they would take calcium in a future pregnancy and that they would recommend calcium to other pregnant women. Women were more likely to consume calcium supplements if they had higher knowledge of calcium benefits (Odds Ratio (OR) 11.7, 95% Confidence Interval (CI): 5.97-22.86) and higher general education (OR 2.59, 95% CI: 2.21-3.05). Higher adherence was also reported in those with higher education (OR 1.45, 95% CI: CI 1.31 to 1.60).(Liu et al., 2019) Higher nutrition knowledge was also associated with taking 6 times more calcium tablets. One paper, evaluating calcium supplement intake before and after nutritional interventions, showed an association between maternal knowledge of nutrition and higher calcium supplement intake (β ~31.9, 95% CI: 20.9, 43.0), however the paper also highlighted there were still large gaps between knowledge and practices, as the intake of calcium supplement tablets during 6 month was low, 82 ±66 out of the recommended 180 tablets.	(Liu et al., 2019; Nguyen et al., 2019, 2017; Thapa et al., 2016)	Four studies. 1 high quality, 1 low quality and 2 moderate quality studies.

For peer review only

**2**	**Believe about the intervention**		
2.1	**Fears about side-effects as barriers to calcium uptake among women**<br>Quantitative evidence supported qualitative findings as few women reported being discouraged to take the supplements by friends (4%) and elder women (3%).	(Martin et al., 2017)	1 Low quality study.
2.2	**Experiences of side effects**<br>Quantitative evidence supported the qualitative findings as some women reported experiencing side effects, usually related to gastrointestinal symptoms. The reported side-effects rates were usually low, 4% of women mentioning nausea or vomiting or constipation in one paper while in another paper 14.9% of women reported that side effects was the reason for missing a dose of IFA or calcium. These could cause supplement discontinuation or erratic supplement intake for one to 10 days after side effects were felt in around 5% of women.	(Baxter et al., 2014; Ghosh-Jerath et al., 2015; Shakya Shrestha et al., n.d.; Thapa et al., 2016)	Four studies. 1 high quality, 2 low quality and 1 very low quality study.
2.3	**Concerns of being stigmatized as HIV patient**<br>No data supporting this theme from quantitative evidence.	No data supporting this theme from quantitative evidence.	No data supporting this theme from quantitative evidence.
2.4	**Positive perceptions about calcium**<br>Quantitative evidence supported the qualitative findings that women's beliefs about the importance of calcium supplements to both the woman's and baby's health and on being able to consume it daily are associated with consuming calcium supplements. Positive beliefs about calcium supplements and self-efficacy were associated with taking calcium supplements (OR 4.6, 95% CI: 2.0 to 10.5) and with taking a higher number of supplements (OR 2.77, 95% CI: 1.68 to 4.57).	(Nguyen et al., 2019)	1 Moderate quality study.
**3**	**Calcium supplement characteristics and regimens**		
3.1	**Varying preferences about characteristics of calcium tablets**<br>Quantitative evidence supported qualitative findings regarding varying preferences calcium supplement's organoleptic properties. One paper which evaluate the impact of a program to implement calcium supplementation showed that most women (77%) reported preferences for conventional tablets that were easier to take and swallow, while the least preferred vehicle was unflavoured powder, as women dislike the taste.(Baxter et al., 2014) Another paper reported that conventional tablets had an acceptable taste (83.9% of women). Chewable tablets	(Baxter et al., 2014; Omotayo et al., 2018b; Thapa et al., 2016)	Three studies. 1 Low quality and 2 high quality studies.

	were preferred by most women (74%) in another paper. Some characteristics that women considered while taking the supplements include tablet's flavour, chewable or swallow, taken with water or not, smell, and size.		
3.2	**Adherence challenges due to routines** In two quantitative studies, women preferred to take fewer tablets per day. However, in one study, women who were allocated to the study arm that used more frequent doses took more calcium overall.	(Baxter et al., 2014; Omotayo et al., 2018b, 2018a)	Three studies. 1 High quality, 1 moderate and 1 low quality studies.
**4**	**Daily routines and food insecurity**		
4.1	**Adherence challenges due to routines** In quantitative evidence, women described busy work schedules and not being at home as also contributing to forgetting to consume their calcium supplements.	(Baxter et al., 2014; Shakya Shrestha et al., n.d.; Thapa et al., 2016)	Three studies. 1 High quality, 1 low and 1 very low-quality studies.
4.2	**Food insecurity as a barrier to calcium uptake** Quantitative evidence supported qualitative findings that women with food security, high socio-economic status and living in urban areas are more likely to consume calcium supplements as compared to their counterparts. Food security was associated with consumption of 6 more calcium tablets.	(Liu et al., 2019; Nguyen et al., 2017)	Two studies. 1 High quality and 1 low quality studies.
**5**	**Strategies to improve use**		
5.1	**Implementation of reminders to promote adherence** Quantitative evidence supported the qualitative findings that distribution of behaviour change materials to women, such as pill-taking calendars, were associated with increased adherence	(Omotayo et al., 2018b)	1 High quality study.
5.2	**Importance of family support and adherence partner implementation** Quantitative evidence supported the qualitative findings that social support is important in encouraging women to consume calcium supplements. Women considered involving a husband, partner, or family in education sessions or appointing someone as the "adherence partner" to be an acceptable strategy for promoting adherence. Women often chose their husband (52%), older female relative (23%), children (14%), cousins or other relatives as adherence partners (8%), and were satisfied with the reminders and support received from their adherence partner. However, a randomised trial assessing adherence partners in improving calcium supplement intake showed that high social support, instead of	(Martin et al., 2017; Nguyen et al., 2019, 2018, 2017; Omotayo et al., 2018b)	Five studies. 3 high quality studies, 1 moderate and 1 low quality studies.

For peer review only

	adherence partners alone, was associated with higher adherence to calcium supplement (OR: 2.10; 95% CI: 1.32, 3.34). Women with high family support reported higher intake of calcium supplements (OR = 2.1).		
5.3	**Counselling facilitates calcium uptake**<br>Quantitative evidence extended qualitative findings where not only counselling, but also starting antenatal contacts at early gestational age, higher number of antenatal contacts, and receiving free calcium supplements were associated with higher calcium intake by women. One paper reports that women were 59 times more likely to consume calcium supplements if they had received them for free.	(Liu et al., 2019; Nguyen et al., 2019, 2017; Thapa et al., 2016)	Four studies. 2 High quality studies, 1 moderate and 1 low quality studies.
**Health care providers' factors**			
**6**	**Healthcare provider knowledge and training**		
6.1	**Varied knowledge about preeclampsia/eclampsia among providers**<br>No data supporting this theme from quantitative evidence.	No data supporting this theme from quantitative evidence.	No data supporting this theme from quantitative evidence.
6.2	**Inadequate training**<br>No data supporting this theme from quantitative evidence.	No data supporting this theme from quantitative evidence.	No data supporting this theme from quantitative evidence.
**7**	**Beliefs on the intervention**		
7.1	**Perceived overmedicalization when prescribing calcium supplements**<br>No data supporting this theme from quantitative evidence.	No data supporting this theme from quantitative evidence.	No data supporting this theme from quantitative evidence.
7.2	**Beliefs about calcium**<br>No data supporting this theme from quantitative evidence.	No data supporting this theme from quantitative evidence.	No data supporting this theme from quantitative evidence.
**8**	**Structural factors**		
8.1	**High workload, inadequate staffing, stock out, and lack of equipment**<br>Quantitative evidence extended qualitative findings by showing that in the context of a comprehensive integrated program including the implementation of job aids, training, guidelines, monitoring, and feedback session for healthcare providers, could overcome barriers in prescribing women with calcium supplements.	(Omotayo et al., 2018a)	1 Moderate study

References

Baxter, J.-A.B., Roth, D.E., Al Mahmud, A., Ahmed, T., Islam, M., Zlotkin, S.H., 2014. Tablets Are Preferred and More Acceptable Than Powdered Prenatal Calcium Supplements among Pregnant Women in Dhaka, Bangladesh. J. Nutr. 144, 1106–1112. https://doi.org/10.3945/jn.113.188524

Ghosh-Jerath, S., Devasenapathy, N., Singh, A., Shankar, A., Zodpey, S., 2015. Ante natal care (ANC) utilization, dietary practices and nutritional outcomes in pregnant and recently delivered women in urban slums of Delhi, India: an exploratory cross-sectional study. Reprod. Health 12, 20. https://doi.org/10.1186/s12978-015-0008-9

Liu, D., Cheng, Y., Dang, S., Wang, D., Zhao, Y., Li, C., Li, S., Lei, F., Qu, P., Mi, B., Zhang, R., Li, J., Zeng, L., Yan, H., 2019. Maternal adherence to micronutrient supplementation before and during pregnancy in Northwest China: a large-scale population-based cross-sectional survey. BMJ Open 9, e028843. https://doi.org/10.1136/bmjopen-2018-028843

Martin, S.L., Omotayo, M.O., Pelto, G.H., Chapleau, G.M., Stoltzfus, R.J., Dickin, K.L., 2017. Adherence-specific social support enhances adherence to calcium supplementation regimens among pregnant women. J. Nutr. 147. https://doi.org/10.3945/jn.116.242503

Nguyen, P.H., Frongillo, E.A., Sanghvi, T., Wable, G., Mahmud, Z., Tran, L.M., Aktar, B., Afsana, K., Alayon, S., Ruel, M.T., Menon, P., 2018. Engagement of Husbands in a Maternal Nutrition Program Substantially Contributed to Greater Intake of Micronutrient Supplements and Dietary Diversity during Pregnancy: Results of a Cluster-Randomized Program Evaluation in Bangladesh. J. Nutr. 148, 1352–1363. https://doi.org/10.1093/jn/nxy090

Nguyen, P.H., Kachwaha, S., Avula, R., Young, M., Tran, L.M., Ghosh, S., Agrawal, R., Escobar-Alegria, J., Patil, S., Menon, P., 2019. Maternal nutrition practices in Uttar Pradesh, India: Role of key influential demand and supply factors. Matern. Child Nutr. 15. https://doi.org/10.1111/mcn.12839

Nguyen, P.H., Sanghvi, T., Kim, S.S., Tran, L.M., Afsana, K., Mahmud, Z., Aktar, B., Menon, P., 2017. Factors influencing maternal nutrition practices in a large scale maternal, newborn and child health program in Bangladesh. PLoS One 12, e0179873. https://doi.org/10.1371/journal.pone.0179873

Omotayo, M.O., Dickin, K.L., Pelletier, D.L., Martin, S.L., Kung'u, J.K., Stoltzfus, R.J., 2018a. Feasibility of integrating calcium and iron-folate supplementation to prevent preeclampsia and anemia in pregnancy in primary healthcare facilities in Kenya. Matern. Child Nutr. 14, e12437. https://doi.org/10.1111/mcn.12437

Omotayo, M.O., Martin, S.L., Stoltzfus, R.J., Ortolano, S.E., Mwanga, E., Dickin, K.L., 2018b. With adaptation, the WHO guidelines on calcium supplementation for prevention of pre-eclampsia are adopted by pregnant women. Matern. Child Nutr. 14, e12521. https://doi.org/10.1111/mcn.12521

Shakya Shrestha, S., Adhikari, R., Tamrakar, S., Shrestha, R., Shrestha, A., n.d. Adherence to Iron, Folic Acid and Calcium Supplement and Factors Affecting it among the Antenatal Care Attending Women in a Tertiary Care Hospital: A Cross Sectional Study. Kathmandu Univ. Med. J. (KUMJ) 18, 186–192.

Thapa, K., Sanghvi, H., Rawlins, B., Karki, Y.B., Regmi, K., Aryal, S., Aryal, Y., Murakami, P., Bhattarai, J., Suhowatsky, S., K., T., H., S., B., R., Y.B., K., K., R., S., A., Y., A., P., M., J., B., S., S., 2016. Coverage, compliance, acceptability and feasibility of a program to prevent pre-eclampsia and eclampsia through calcium supplementation for pregnant women: An operations research study in one district of Nepal. BMC Pregnancy Childbirth. https://doi.org/10.1186/s12884-016-1033-6

Appendix 7. COM-B Mapping Table

Behavior aimed: calcium supplementation use by women and health providers						
No	COM-B Domain	TDF Domain*	List of factors affecting calcium use	Stakeholders affected (women, health providers, partner)	Evidence Source (Quantitative/Qualitative)	Barriers or facilitators to calcium use
**Women's factors**						
1	Capability	Know	Limited knowledge about preeclampsia/eclampsia	Women	Qualitative	
2	Capability	Know	Counselling on information about preeclampsia and calcium from their providers independent to woman risk	Women	Qualitative	
3	Capability	Know	High knowledge of calcium supplementation	Women	Quantitative	
4	Capability	Know	High education of women	Women	Quantitative	
5	Capability	Know	Symptoms were believed to be linked to evil attacks or nutritional deficiencies	Women	Qualitative	
6	Capability	Know	Inadequate information on preeclampsia/eclampsia and calcium from providers	Women	Qualitative	
7	Capability	Know	Receive adequate information about condition and calcium from their providers	Women	Qualitative	
8	Motivation	Bel Cons	Preeclampsia/eclampsia was not considered as a serious problem by women	Women	Qualitative	
9	Motivation	Bel Cons	Experiences and fears of side effects	Women	Qualitative and quantitative	
10	Motivation	Bel Cons	Assurance of calcium safety	Women	Qualitative	
11	Motivation	Bel Cons	Safety of calcium was not assured	Women	Qualitative	
12	Motivation	Bel Cons	Experienced and expected benefits from taking calcium and folic acid	Women	Qualitative and quantitative	
13	Motivation	Bel Cap	Belief that having essential information would help them making informed decisions	Women	Qualitative	
14	Motivation	Bel Cap	Belief that providers is a reliable source of information	Women	Qualitative	
15	Motivation	Bel Cap	Belief of being able to consume calcium daily	Women	Quantitative	
16	Motivation	Em	Valued information, education and communication (IEC) materials	Women	Qualitative	
17	Motivation	Em	Positive perceptions on calcium tablet characteristics (e.g. size, taste, smell)	Women	Qualitative and quantitative	
18	Motivation	Em	Women felt that 3-4 pills per day at multiple times were too many	Women	Quantitative and qualitative	
19	Motivation	Em	Inconvenience in taking pill daily	Women	Quantitative and qualitative	
20	Motivation	Em	Belief that one combined pill per day could ease burden	Women	Qualitative	
21	Motivation	Em	Feeling confident taking calcium after receiving adequate information from provider	Women	Quantitative	
22	Opportunity	Soc	Discouragement by family, neighbours and community in taking calcium	Women	Qualitative and quantitative	

For peer review only

23	Opportunity	Soc	Stigmatisation of having supplements and posters with HIV	Women	Qualitative	
24	Opportunity	Soc	Family support in consuming calcium	Women	Qualitative and quantitative	
25	Opportunity	Soc	Positive belief and experiences about 'adherence partners'	Women	Qualitative and quantitative	
26	Opportunity	Ev	Conflicting daily routine with taking calcium	Women	Qualitative and quantitative	
27	Opportunity	Ev	Food insecurity	Women	Qualitative and quantitative	
28	Opportunity	Ev	Early initiation and frequent antenatal visits	Women	Quantitative	
29	Opportunity	Ev	Receiving free calcium supplements	Women	Quantitative	
30	Opportunity	Ev	Reminder tools distribution, such as home based posters and pill-taking calendars	Women	Qualitative and Quantitative	
31	Opportunity	Ev	Universal free calcium distribution through antenatal care	Women	Quantitative	
**Providers factors**						
1	Capability	Know	Varied knowledge on pre-eclampsia/eclampsia	Providers	Qualitative	
3	Capability	Skills	Incontinuity and non-in-depth training about pre-eclampsia/eclampsia	Providers	Qualitative	
2	Capability	Skills	Felt inadequately trained	Providers	Qualitative	
4	Capability	Skills	Continuous training to manage the condition and address resistance from community	Providers	Qualitative	
5	Motivation	Bel Cons	Positive belief about calcium supplementation benefits	Providers	Qualitative	
6	Motivation	Bel Cons	Belief that preeclampsia/eclampsia was not a priority health concern	Providers	Qualitative	
7	Motivation	Bel Cons	Perceived over-medicalization to prescribe calcium to low risk women	Women and Providers	Qualitative	
8	Motivation	Bel Cons	Fears in generating anxiety on low-risk women	Providers	Qualitative	
9	Motivation	Bel Cons	Belief that women should get a chance to receive information regardless risk	Providers	Qualitative	
10	Opportunity	Ev	Providers felt that providing calcium would increase workload	Providers	Qualitative	
11	Opportunity	Ev	Inadequate number of staff providing care	Providers	Qualitative	
12	Opportunity	Ev	Comprehensive integrated program (job aids, training, guidelines, regular supplies)	Providers	Quantitative	

Barriers	
Facilitators	

* Know: Knowledge, Phys: Physical skills, Mem: Memory, attention and decision processes, Beh Reg: Behavioural regulation, Em: Emotion, Id: Social/professional role and identity, Bel Cons: Belief about consequences, Bel Cap: Belief about capabilities, Int: Intentions, Opt: Optimisms, Ev: Environmental context and resources, Soc: Social influences

For peer review only
